# Multi-omic characterization of pediatric ARDS via nasal brushings

**DOI:** 10.1186/s12931-022-02098-3

**Published:** 2022-07-09

**Authors:** James G. Williams, Rashika Joshi, David Haslam, Nadir Yehya, Rhonda L. Jones, Aditi Paranjpe, Mario Pujato, Krishna M. Roskin, Patrick M. Lahni, Hector R. Wong, Brian M. Varisco

**Affiliations:** 1 Critical Care Medicine, Cincinnati Children’s Hospital Medical Center, 3333 Burnet Avenue, MLC 7006, Cincinnati, OH 45229 USA; 2 Infectious Diseases, Cincinnati Children’s Hospital Medical Center, Cincinnati, OH USA; 3 University of Cincinnati College of Medicine, Cincinnati, OH USA; 4 Critical Care Medicine, Children’s Hospital of Philadelphia, Philadelphia, PA USA; 5 Perlman School of Medicine, University of Philadelphia, Philadelphia, PA USA; 6 Biomedical Informatics, Cincinnati Children’s Hospital Medical Center, Cincinnati, OH USA; 7 Production Informatics, AstraZeneca Oncology Division, Gaithersburg, MD USA

**Keywords:** Transcriptomics, Subclassification, Acute lung injury, Pediatric acute respiratory distress syndrome

## Abstract

**Rationale:**

While nasal brushing transcriptomics can identify disease subtypes in chronic pulmonary diseases, it is unknown whether this is true in pediatric acute respiratory distress syndrome (PARDS).

**Objectives:**

Determine whether nasal transcriptomics and methylomics can identify clinically meaningful PARDS subgroups that reflect important pathobiological processes.

**Methods:**

Nasal brushings and serum were collected on days 1, 3, 7, and 14 from control and PARDS subjects from two centers. PARDS duration was the primary endpoint.

**Measurements and main results:**

Twenty-four control and 39 PARDS subjects were enrolled. Two nasal methylation patterns were identified. Compared to Methyl Subgroup 1, Subgroup 2 had hypomethylation of inflammatory genes and was enriched for immunocompromised subjects. Four transcriptomic patterns were identified with temporal patterns indicating injury, repair, and regeneration. Over time, both inflammatory (Subgroup B) and cell injury (Subgroup D) patterns transitioned to repair (Subgroup A) and eventually homeostasis (Subgroup C). When control specimens were included, they were largely Subgroup C. In comparison with 17 serum biomarkers, the nasal transcriptome was more predictive of prolonged PARDS. Subjects with initial Transcriptomic Subgroup B or D assignment had median PARDS duration of 8 days compared to 2 in A or C (p = 0.02). For predicting PARDS duration ≥ 3 days, nasal transcriptomics was more sensitive and serum biomarkers more specific.

**Conclusions:**

PARDS nasal transcriptome may reflect distal lung injury, repair, and regeneration. A combined nasal PCR and serum biomarker assay could be useful for predictive and diagnostic enrichment.

*Trial registration* Clinicaltrials.gov NCT03539783 May 29, 2018.

**Supplementary Information:**

The online version contains supplementary material available at 10.1186/s12931-022-02098-3.

## Introduction

In the pediatric intensive care unit (PICU), pediatric acute respiratory distress syndrome (PARDS) is a leading source of morbidity and mortality [1]. Despite decades of research and many large, multicenter, randomized clinical trials in ARDS, the only consensus therapies are supportive: the use of low tidal volume ventilation and employing a restrictive fluid strategy [2, 3]. A criticism of many studies in ARDS has been failure to account for etiologic, biologic, and physiologic differences [4]. Recent work has suggested the presence of a hyperinflammatory ARDS subgroup. In latent group analysis of a clinical trial of the FACTT trial [3] subjects with greater inflammation were more likely to survive with a fluid liberal strategy, than subjects without this subphenotype [5]. Similarly, hyperinflammatory ARDS patients had lower mortality with higher positive end expiatory pressure (PEEP) levels [6]. Similar findings were recently shown in PARDS patients [7].Thus, inflammatory serum biomarkers may differentiate ARDS subphenotypes that confer greater mortality risk and might be helpful in directing therapy.

However, there is likely a limit to the extent to which serum assays accurately reflect lung pathology. While peripheral blood gene expression profiling in pediatric sepsis had identified important subgroups that correlate with outcome [8, 9], there is ~ 40% concordance and ~ 20% discordance of gene expression between lung and peripheral leukocytes [10]. RAGE, ANG2 and inflammatory cytokines are increased in multiple non-lung conditions and likely lack specificity. Bronchial and nasal gene expression profiling was highly diagnostic for the presence of lung cancer in smokers [11], and for corticosteroid sensitivity in asthma [12], and nasal transcriptomic profiling can differentiate between COPD and non-COPD in smokers [13]. Differences in nasal DNA methylation have been established in pediatric and adult asthma [14, 15]. Recent functional data demonstrated increased nasal potential difference in at-risk adults who go onto develop ARDS [16]. This, the nasal epithelium may provide a window into distal lung epithelial processes.

Given these studies, we hypothesized that multi-omic characterization of PARDS nasal brushings and matched serum could be used to define and provide pathobiological insights into PARDS. Our principal aim was to test whether changes in the nasal transcriptome during PARDS correlated with PARDS course and reflected potentially meaningful biological processes. Our secondary aim was to compare these transcriptomic changes with the nasal methylome and serum biomarkers previously reported important in PARDS. To accomplish this, we assessed transcriptomic and methylomic profiles form nasal brushings and compared these to time-matched serum biomarker profiles. We found four patterns of gene expression that corresponded to injury (two patterns), repair/regeneration, and homeostasis and that PARDS duration was greater in subjects with the first two compared to the latter two patterns. Complimentary studies of the epigenome suggested the presence of risk loci related to inflammatory genes. Lastly, predictive modeling suggested that serum biomarkers and nasal transcriptomics provided different and complementary information.

## Materials and methods

### Human subjects

Research was conducted under approval from the Cincinnati Children’ Hospital and Children’s Hospital of Philadelphia Institutional Review Boards (2017-1345 and 20-017205 respectively) and registered with clinicaltrials.gov (NCT#03539783).

### Subject eligibility

Patients admitted to the PICU < 18 years of age who were invasively mechanically ventilated and meeting consensus PARDS criteria [1] or who were admitted to the PICU for a non-pulmonary reason and with expected hospitalization ≥ 7 days were eligible for enrollment. Patients with limited resuscitation orders, that required chronic mechanical ventilation, with a baseline oxygen requirement (≥ 2 L per minute for PARDS, any for Control) or with high-risk of nasal bleeding were excluded. Informed written consent was obtained from the patient’s parent or guardian with patient assent as appropriate.

### Study design

Consented patients had nasal brushings and serum collected on the day of enrollment (day 1), and on study days 3, 7, and 14 if they were still admitted. No blood was collected if it could not be drawn from a vascular access device or from a clinically indicated venipuncture, and a patient, family, or provider could refuse brushing without removal from the study.

### Nasal brushing

Nasal inferior turbinate brushings were collected, and brush heads were cut and preserved in RNAProtect buffer (Qiagen) at – 80 °C.

### Bronchial brushing

In subjects undergoing clinically indicated bronchoscopy in the CCHMC PICU bronchial brushing was performed in a healthy-appearing distal bronchus with concomitant nasal brushing.

### RNA and DNA extraction and sequencing

Brushes were thawed on ice with 1:100 β-mercaptoethanol to reduce mucus disulfide bond. The extract was passed through a QIAShredder and AllPrep Columns (Qiagen) with extraction of first DNA and then RNA (after DNAse treatment). Barcoded libraries were created using New England Biosystems Single Cell/Low Input RNA kit per manufacturer instructions. For each specimen, ten million, 150 base pair, paired-end read were obtained using a NovaSeq 6000. Eluted DNA was sheared using a Covaris M220 device and methylated cytosines converted to thymidines using the NEBNext Enzymatic MethylSeq kit with barcoding. Each methylated DNA library was sequenced at 20X genomic coverage using a NovaSeq 6000.

### Serum protein quantification and predictive statistic comparisons

Serum samples were analyzed in duplicate for Angiopoietin2 (ANG2), Granzyme B (GrB), Intercellular Adhesion Molecule1 (ICAM1), Interferon-γ (IFNγ), Interleukin-6 (IL6), IL8, IL10, IL17, IL18, Surfactant Protein D, Tumor Necrosis Factorα (TNF-α), TNF Receptor Soluble Factor 1A (TNFSF1A), and RAGE using R&D Systems reagents and a Luminex 200 instrument.

### Bioinformatics

RNA reads were aligned to GRCh38 and count matrices generated using STAR. Human differential gene expression was calculated using DESeq2 and other packages described in the Additional file 1. Clustering was performed using Ward’s Minimum Variance Method and clusters created by iterative comparison of distances using the hclust function. For metagenomic analysis, bacterial and viral RNA sequences were aligned using Kraken2 and normalized reads assessed using vegan. DNA sequences were aligned to GRCh38 and methylated and non-methylated coverage matrices generated using Bismark. Coverage files were analyzed using methylKit and other packages described in the Additional file 1. ToppGene was used for gene set enrichment analysis (GSEA). For genes and microbial species, a false discovery rate of < 0.1 was considered significant.

### Statistical analysis

Non-parametric continuous variables were compared using Wilcoxon rank sum test using a Dunn test with Bonferroni correction for post hoc comparisons. Categorical variables were compared by fisher exact test using the R statistical package and rstatix. Finalfit was used for logistic regression, and receiver operator characteristic curves were generated and analyzed using pROC. p-values < 0.05 were considered significant.

### Data availability

Datasets are publicly available as GSE192364 and GSE192926. Other data is freely available by contacting the corresponding author.

## Results

### Subject enrollment and cohort characteristics

We enrolled 24 control and 39 PARDS subjects from April 1, 2018 to June 30, 2021. Subject characteristics are presented in Table 1, and Additional file 12: Table S1 provides patient-specific data and describes assays conducted on each specimen, and Additional file 2: Fig. S1 summarizes the analyzed specimens.

**Table 1:** Subject demographics

		PARDS (n = 39)	Control (n = 23)	p-value
Age	Median (IQR)	6.8 (3.5 to 10.3)	6.1 (1.3 to 11.2)	0.471
Sex	Female	17 (43.6)	9 (39.1)	0.794
	Male	22 (56.4)	14 (60.9)	
Race	Non-White	12 (30.8)	3 (13.0)	0.138
	White	27 (69.2)	20 (87.0)	
Genetic Synd. or D.D	False	18 (46.2)	20 (87.0)	0.003
	True	21 (53.8)	3 (13.0)	
Baseline lung disease	False	26 (66.7)	14 (60.9)	0.784
	True	13 (33.3)	9 (39.1)	
Baseline kidney or liver disease	False	34 (87.2)	21 (91.3)	1
	True	5 (12.8)	2 (8.7)	
Immunocompromised	False	27 (69.2)	22 (95.7)	0.021
	True	12 (30.8)	1 (4.3)	
Highest PELOD2	Median (IQR)	13.0 (11.0 to 15.5)	8.0 (6.5 to 10.0)	< 0.001
Highest PARDS category	None	1 (2.6)^#^	22 (95.8)	< 0.001
	Mild	10 (25.6)	1 (4.2)^##^	
	Moderate	16 (41.0)	0 (0.0)	
	Severe	12 (30.8)	0 (0.0)	
Viral infection	False	20 (51.3)	23 (100)	< 0.001
	True	19 (48.7)		
Any corticosteroids	No	15 (38.5)	16 (69.6)	0.034
	Yes	24 (61.5)	7 (30.4)	
Ventilator free days	Median (IQR)	19.4 (13.0 to 22.1)	0.0 (0.0 to 20.6)	0.016
PICU days	Median (IQR)	17.0 (10.0 to 29.5)	5.0 (2.0 to 7.5)	< 0.001
Hospital days	Median (IQR)	29.0 (14.0 to 60.0)	14.0 (5.0 to 19.0)	< 0.001
Mortality	Died	7 (17.9)		0.04
	Survived	32 (82.1)	23 (100.0)	

^*^Categorical variables compared by Fisher Exact Test

^**^Continuous variables compared by Wilcoxon Rank Sum Test

^#^One PARDS subject was consented and enrolled but brushing was delayed and no longer met critiera

^##^One control subject developed PARDS after enrollment

### Overall approach

For methylomic and transcriptomic analyses, we first compared nasal and bronchial data, identified subgroups within PARDS, and performed gene set enrichment analysis and multivariate analyses of these subgroups, and lastly compared these subgroups to control specimens. We then compared how well nasal transcriptomics predicted PARDS course compared to serum biomarkers. Lastly, we compared assessed which gene expression changes had coordinate changes in the epigenome.

### The methylation patterns of nasal and bronchial epithelial cells do not differ by collection site

To test whether the epigenetic state of the upper and lower conducing airways were similar, we compared DNA methylation of paired nasal and bronchial specimens. We analyzed 8 paired brushings from 3 PARDS and 1 control subjects. By site, 0.2% percent of regions were differentially methylated (DMRs) between the upper and lower airways with 48% corresponding to annotated CgP islands or shores, and 9% promoters (24 genes, Additional file 13: Table S2, Additional file 3: Fig. S2A, B). By k-means clustering, specimens were more similar by subject than by collection site (Additional file 3: Fig. S2C) indicating similarity of the nasal and bronchial methylomes.

### A subset of PARDS patients have hypomethylation of promoters related to inflammation

**Fig. 1:**
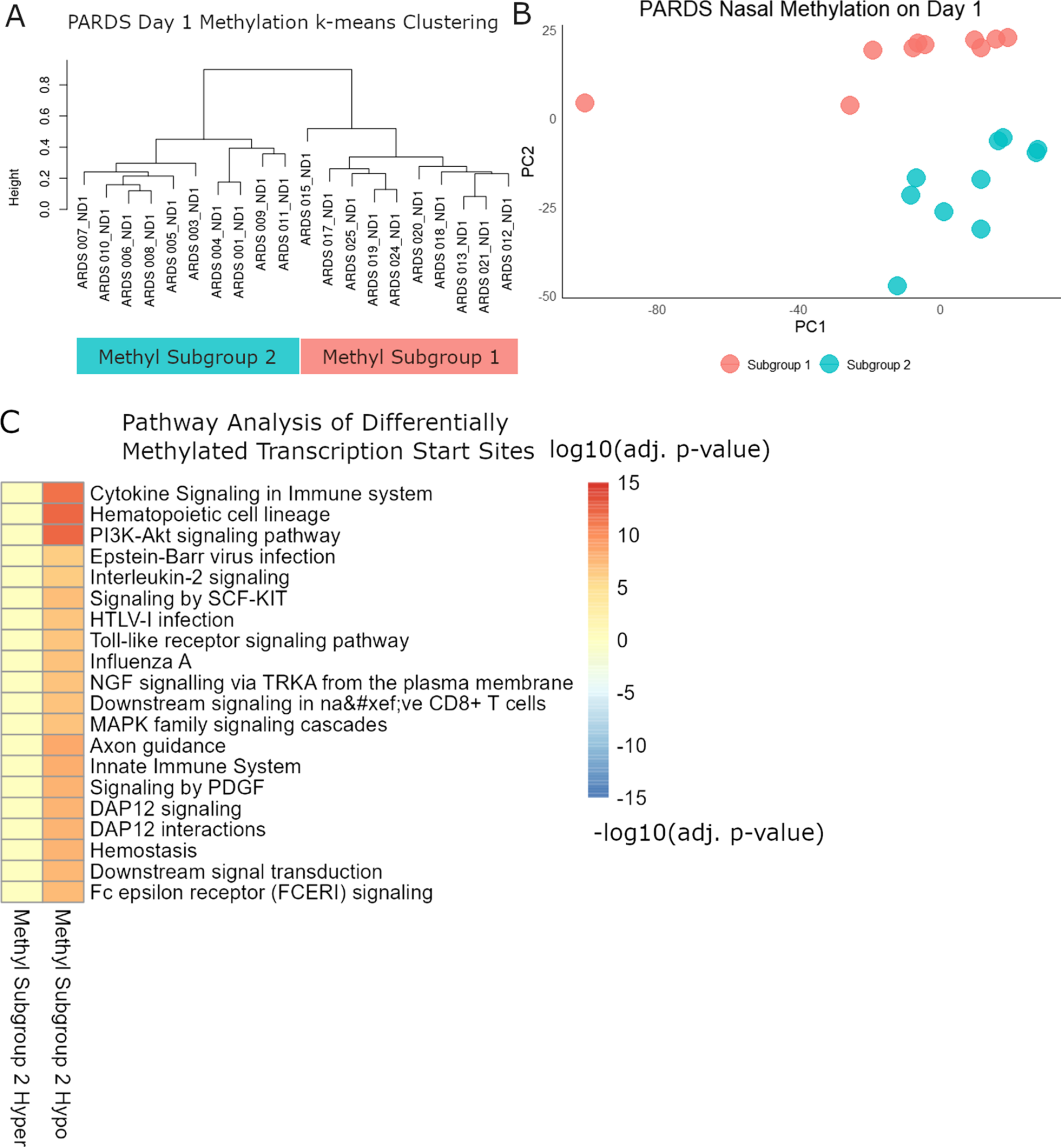
Nasal methylomic patterns on day 1 specimens of control and PARDS subjects. **A** The day 1 DNA methylation of 20 PARDS subjects was compared by k-means clustering. Two clusters were apparent with nine PARDS specimens were classified as Methyl Subgroup 2 and 11 PARDS specimens classified as Methyl Subgroup 1. **B** Principal component plot showing clustering of the two Methyl Subgroups. **C** By gene set enrichment analysis, hypomethylated genes in Methyl Subgroup 2 were largely related to inflammation-related genes

To characterize patterns of methylation within PARDS subjects, we performed analysis of 68 specimens from 20 PARDS subjects. Limiting this analysis to day 1 specimens (20 specimens), two patterns were evident by k-means clustering and principal component analysis (Fig. 1A, B). We labeled these two groups as Methyl Subgroup 1 (11 subjects) and Methyl Subgroup 2 (9 subjects) and compared the differentially methylated transcription start sites. Compared to Subgroup 1, Subgroup 2 had hypermethylation of 422 genes and hypomethylation of 1,258 genes (Additional file 13: Table S2). Gene set enrichment analysis for hypermethylated genes in Subgroup 2 compared to Subgroup 1 found few significant pathways or processes, but pathway analysis was highly significant for hypomethylated genes, and these pathways were related to inflammation (Fig. 1C). In comparing the clinical characteristics and outcomes of the two groups, Subgroup 2 was over half immunocompromised subjects and included all subject deaths and had longer hospitalization. Interesting negative findings between the two groups were no difference in illness or PARDS severity, no difference in frequency of viral infection, and similar ventilator free and PICU days (Table 2).

**Table 2:** Methyl subgroup comparisons (PARDS only)

		Subgroup 1 (n = 12)	Subgroup 2 (n = 21)	p-value
		(n = 11)	(n = 9)	
Age	Median (IQR)	5.2 (3.5 to 9.9)	6.8 (3.0 to 10.3)	0.79
Sex	Female	2 (18.2)	4 (44.4)	0.336
	Male	9 (81.8)	5 (55.6)	
Race	White	11 (100.0)	6 (66.7)	0.074
	Non-White		3 (33.3)	
Genetic Synd. or D.D	False	7 (63.6)	2 (22.2)	0.092
	True	4 (36.4)	7 (77.8)	
Baseline lung disease	False	4 (36.4)	7 (77.8)	0.092
	True	7 (63.6)	2 (22.2)	
Immunocompromised	False	11 (100.0)	4 (44.4)	0.008
	True		5 (55.6)	
Highest PELOD2	Median (IQR)	11.0 (10.5 to 12.0)	12.0 (11.0 to 13.0)	0.227
Highest PARDS category	None	1 (9.1)	0 (0.0)	1
	Mild	2 (18.2)	2 (22.2)	
	Moderate	6 (54.5)	5 (55.6)	
	Severe	2 (18.2)	2 (22.2)	
Viral infection	False	4 (36.4)	5 (55.6)	0.653
	True	7 (63.6)	4 (44.4)	
Any corticosteroids	No	3 (27.3)	5 (55.6)	0.362
	Yes	8 (72.7)	4 (44.4)	
Ventilator free days	Median (IQR)	20.4 (13.0 to 24.0)	17.9 (0.0 to 18.9)	0.159
PICU days	Median (IQR)	15.0 (10.0 to 23.5)	17.0 (15.0 to 90.0)	0.285
Hospital days	Mean (SD)	31.5 (28.3)	87.6 (75.2)	0.034
Mortality	Survived	11 (100.0)	6 (66.7)	0.074
	Died		3 (33.3)	

^*^Categorical variables compared by Fisher Exact Test

^**^Continuous variables compared by Wilcoxon Rank Sum Test

### Methylation changes over time and in comparison to control

**Fig. 2:**
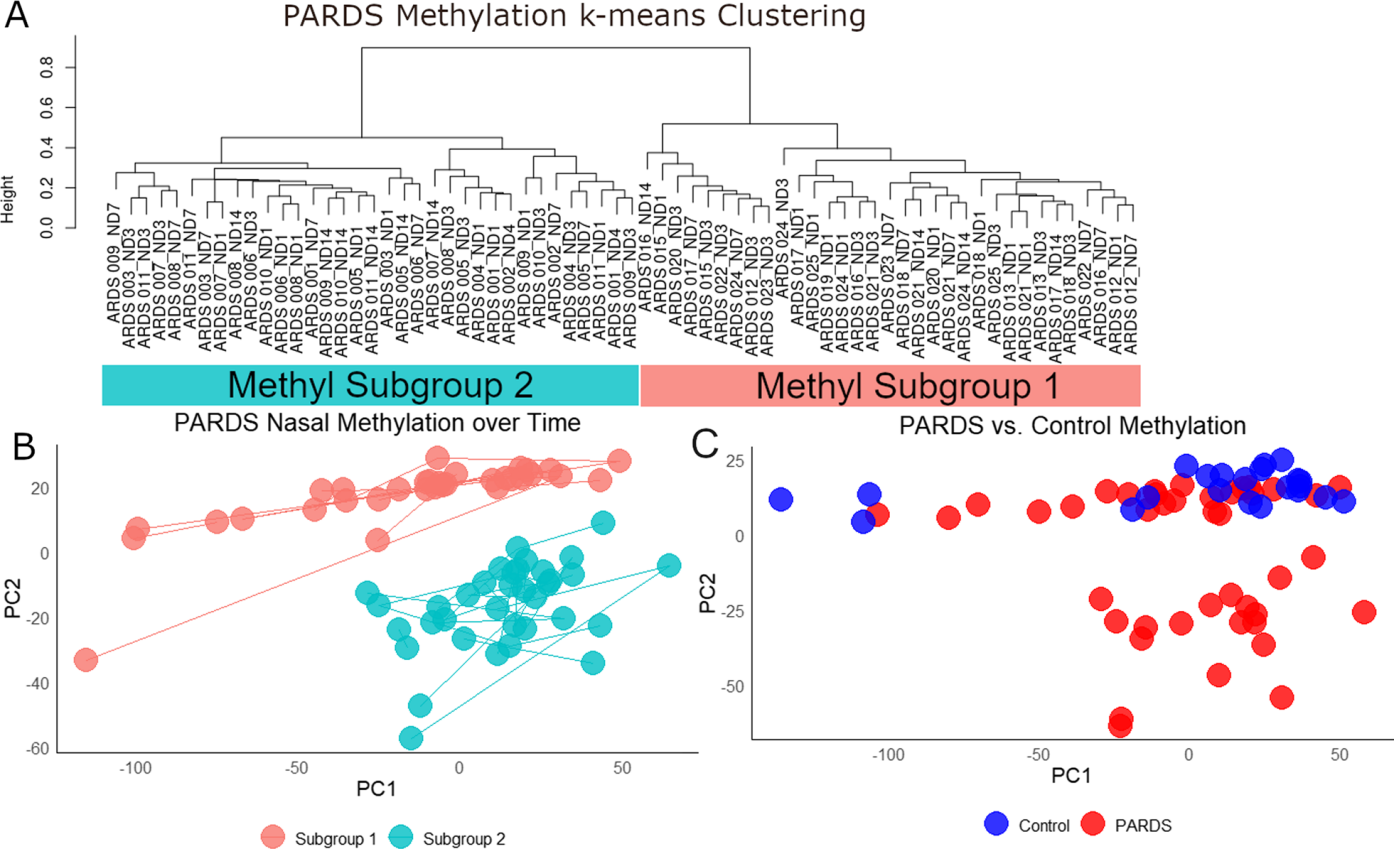
Changes in nasal methylome over time in PARDS and control subjects. **A** In assessing methylation and longitudinal methylation changes in PARDS subjects with at least two usable specimens, we found that all day 1 specimens were identically classified as previous and that the two-group model still described the data well. **B** Principal component plot of PARDS nasal specimens with lines connecting sequential specimens. There was no change in subgroup assignment for any subject despite PARDS resolution in most. **C** Re-analysis and re-clustering of methylomic data of control and PARDS subjects identified two clusters and revealed that all control specimens clustered with a single subset of PARDS specimens

DNA methylation is considered a relatively stable epigenetic feature. We performed methylomic analysis on nasal brushings from control and PARDS subjects in which we had at least two specimens to assess Methylomic Subgroup stability. There were 9 control and 21 PARDS subjects with two or more evaluable specimens (70 total). Methyl Subgroup classification was consistent in all samples over time (Fig. 2A, B). We then sought to identify similarities and differences of these patterns with control subjects. Control subjects were admitted to the PICU without evidence of lung disease and with expected hospitalization stay of at least 7 days (Additional file 12: Table S1). All control subjects had a similar pattern of methylation (Fig. 2C). These data show that a subset of PARDS patients have methylation patterns associated with poor outcomes and that some of these methylation changes are also present in the nasal epithelium of patients without PARDS.

### mRNA-seq data filtering, normalization and batch correction

One hundred forty-two nasal specimens from 56 subjects were sequenced, aligned, and normalized with removal of mitochondrial and ribosomal transcripts. Transcripts without at least five reads in half of specimens, and specimens without at least 100,000 reads and 5000 unique transcripts were excluded from further analysis. Expression values of 10,846 transcripts in 129 specimens from 37 PARDS and 15 control subjects were normalized by batch prior to analysis (Additional file 4: Fig. S3A–C). Analyzing PARDS specimens suggested that six or seven principal components best characterized the data, and covariate analysis for PARDS severity, comorbidity, infectious etiology, direct lung injury, race, sex, and age only identified age as being significantly correlated with any component and this only explaining 0.6% of dataset variance (Additional file 4: Fig. S3D, E).

### Nasal and bronchial transcriptomes are dynamic

In comparing the transcriptomes of the nasal and bronchial epithelium, twelve specimens from five PARDS and one control subject were evaluable. By k-means clustering, principal component analysis, and Euclidean distance, specimens tended to cluster more closely by subject than by collection site (Additional file 5: Fig. S4). As in healthy adults [17], upper and lower conducting airway gene expression is similar in PARDS.

### Four transcriptional patterns in the nasal epithelium of PARDS subjects

**Fig. 3:**
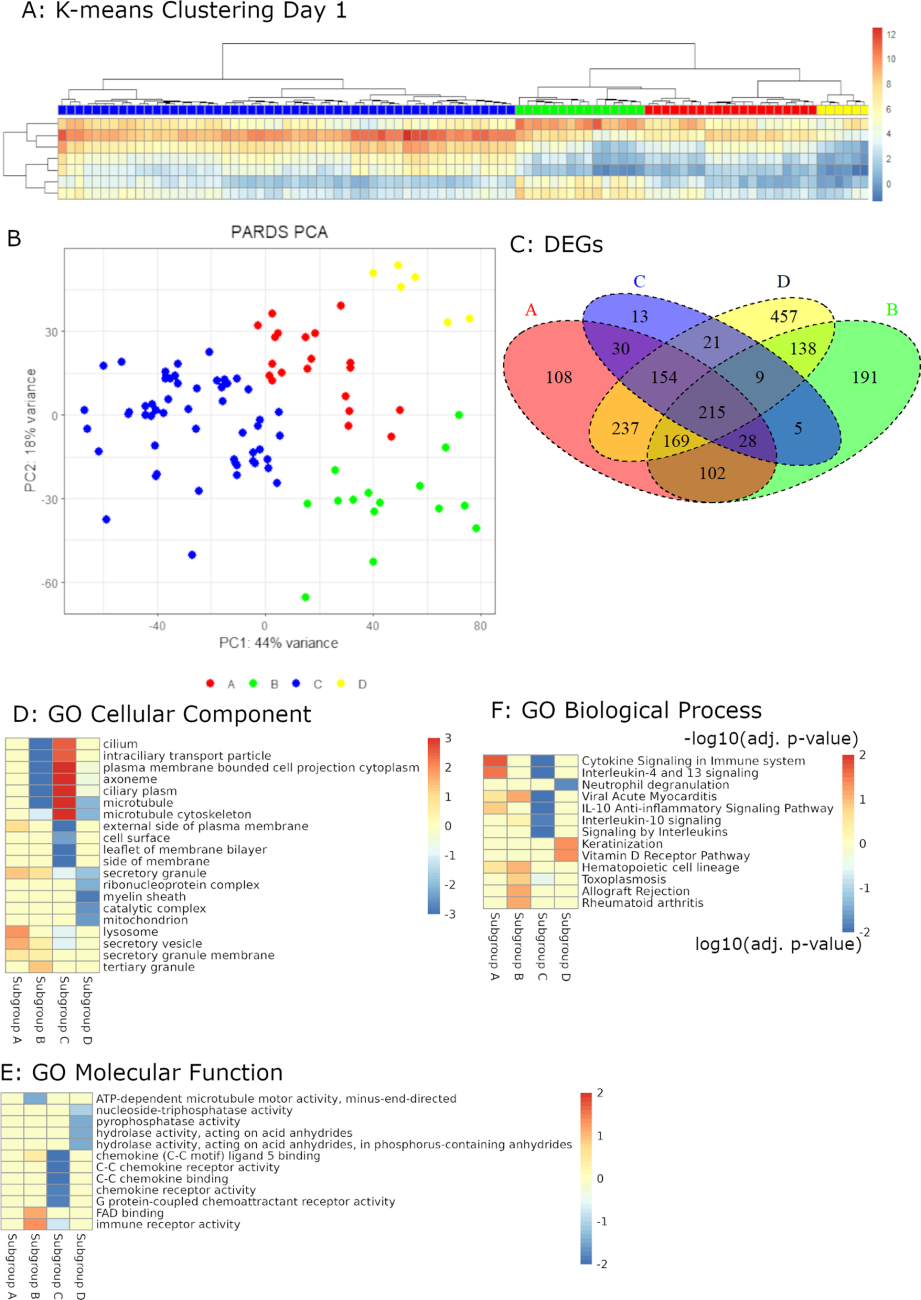
Nasal transcriptomic classification in PARDS subjects. **A** Nasal transcriptomic data from 39 PARDS subjects were clustered by k-means identifying four subgroups, Nasal Transcriptomic Subgroups A, B, C, and D. The eight rows are summarized values for the eight modules indicated by dataset analysis (Additional file 4: Fig. S3). **B** Principal component analysis showed clustering of these four subgroups in both Principal Component 1 (PC1) which largely corresponded to epithelial cell function and PC2 which largely corresponded to inflammation. **C** Venn diagram summarizing the number of differentially expressed genes (DEGs, fold change ≥ 2, adjusted p-value ≤ 0.1) for specimens in each Subgroup compared to specimens not in that Subgroup. **D** Gene set enrichment analysis (GSEA) for each of these comparisons showed an increase in cilia-related genes in Subgroup C, **E** a reduction in chemokine and cytokine signaling in Subgroup C, **F** increased innate immune signaling in Subgroup B, increased interleukin-4 and 13 signaling in Subgroup A, and increased epithelialization-related processes in Subgroup D

We performed k-means clustering of each evaluable specimen and identified four subgroups within our dataset which we termed A, B, C, and D (Fig. 3A, B). Principal component 1 genes were largely related to ciliary cell function and Interleukin-4 (IL-4) signaling, and principal component 2 genes were largely related to inflammation and chemokine signaling ( Additional file 14: Table S3, Additional file 15: Table S4). We defined a differentially expressed gene (DEG) as one with twofold changed expression with an adjusted p-value of less than 0.1. Subgroups A, B, C, and D contained 1375; 834; 841 and 2038 DEGs and had substantial DEG overlap (Fig. 3C, Additional file 16: Table S5, Additional file 6: Fig. S5). GSEA showed Subgroup A enriched for IL-4, IL-10, and IL-13 signaling. Subgroup B had downregulation of ciliary function-related processes and upregulation of innate immune ones. Subgroup C had upregulation of these same ciliary processes and downregulation of IL-4, IL-10, and IL-13 signaling. Subgroup D had downregulation of both microtubule and innate immune processes and upregulation of processes related to epithelial integrity (Fig. 3D–F). In evaluating the abundance of cell-specific mRNAs, Subgroup A and B both had reduced abundance of ciliated cell mRNAs and Subgroup B had increased myeloid cell mRNAs. Subgroup C had a greater abundance of ciliated cell mRNAs compared to all others, and Subgroup D had reduction epithelial stem cell mRNAs (secretory and basal cells, Table 3). Considering the dataset holistically, subgroups A, B, and D all had some element of epithelial cell dysfunction with the degree of this dysfunction and associated inflammation differentiating the subgroups while subgroup C had preserved epithelial cell function. The only notable difference in clinical characteristics between the subgroups was a trend towards fewer ventilator free days in Subgroup B (Table 4).

**Table 3:** PARDS transcriptomic subgroup cell-specific mRNAs

	A	B	C	D
Up		Secretory cell M1 macrophage Neutrophil	Ciliated cell	
Down	Ciliated cell	Ciliated cell	Neutrophil NK-cell Cytotoxic T-cell	Effector T-cell Basal cell Secretory cell

**Table 4:** Initial nasal transcriptomic subgroup comparisons

	Levels	A (n = 9)	B (n = 7)	C (n = 17)	D (n = 5)	p-value
Age	Median (IQR)	5.2 (4.8 to 11.0)	9.0 (3.2 to 10.3)	7.9 (4.4 to 8.6)	5.3 (5.1 to 8.0)	0.96
Sex	Female	5 (55.6)	3 (42.9)	7 (41.2)	2 (40.0)	0.96
	Male	4 (44.4)	4 (57.1)	10 (58.8)	3 (60.0)	
Race	Non-White	1 (11.1)	3 (42.9)	6 (35.3)	1 (20.0)	0.528
	White	8 (88.9)	4 (57.1)	11 (64.7)	4 (80.0)	
Genetic Synd. or D.D	False	6 (66.7)	1 (14.3)	6 (35.3)	2 (40.0)	0.20
	True	3 (33.3)	6 (85.7)	11 (64.7)	3 (60.0)	
Baseline lung disease	False	7 (77.8)	4 (57.1)	11 (64.7)	4 (80.0)	0.82
	True	2 (22.2)	3 (42.9)	6 (35.3)	1 (20.0)	
Immunocompromised	False	6 (66.7)	4 (57.1)	12 (70.6)	3 (60.0)	0.96
	True	3 (33.3)	3 (42.9)	5 (29.4)	2 (40.0)	
Highest PELOD2	Median (IQR)	12.0 (11.0 to 15.0)	12.0 (11.0 to 14.5)	12.0 (10.0 to 14.0)	15.0 (14.0 to 15.0)	0.71
Highest PARDS category	None	1 (11.1)	0 (0.0)	0 (0.0)	1 (20.0)	0.66
	Mild	2 (22.2)	0 (0.0)	5 (29.4)	1 (20.0)	
	Moderate	4 (44.4)	4 (57.1)	8 (47.1)	2 (40.0)	
	Severe	2 (22.2)	3 (42.9)	4 (23.5)	1 (20.0)	
Viral infection	False	4 (44.4)	3 (42.9)	11 (64.7)	2 (40.0)	0.64
	True	5 (55.6)	4 (57.1)	6 (35.3)	3 (60.0)	
Any corticosteroids	No	3 (33.3)	2 (28.6)	7 (41.2)	3 (60.0)	0.769
	Yes	6 (66.7)	5 (71.4)	10 (58.8)	2 (40.0)	
Ventilator free days	Median (IQR)	21.8 (19.4 to 24.5)	13.0 (0.0 to 18.1)	18.9 (12.9 to 21.3)	17.9 (0.0 to 22.4)	0.082
PICU days	Median (IQR)	20.0 (15.0 to 29.0)	25.0 (17.5 to 61.5)	15.0 (10.0 to 22.0)	8.0 (7.0 to 24.0)	0.294
Hospital days	Median (IQR)	45.0 (28.0 to 103.0)	31.0 (25.0 to 107.5)	20.0 (13.0 to 45.0)	13.0 (12.0 to 56.0)	0.182
Mortality	Died	2 (22.2)	2 (28.6)	2 (11.8)	1 (20.0)	0.774
	Survived	7 (77.8)	5 (71.4)	15 (88.2)	4 (80.0)	

**Fig. 4:**
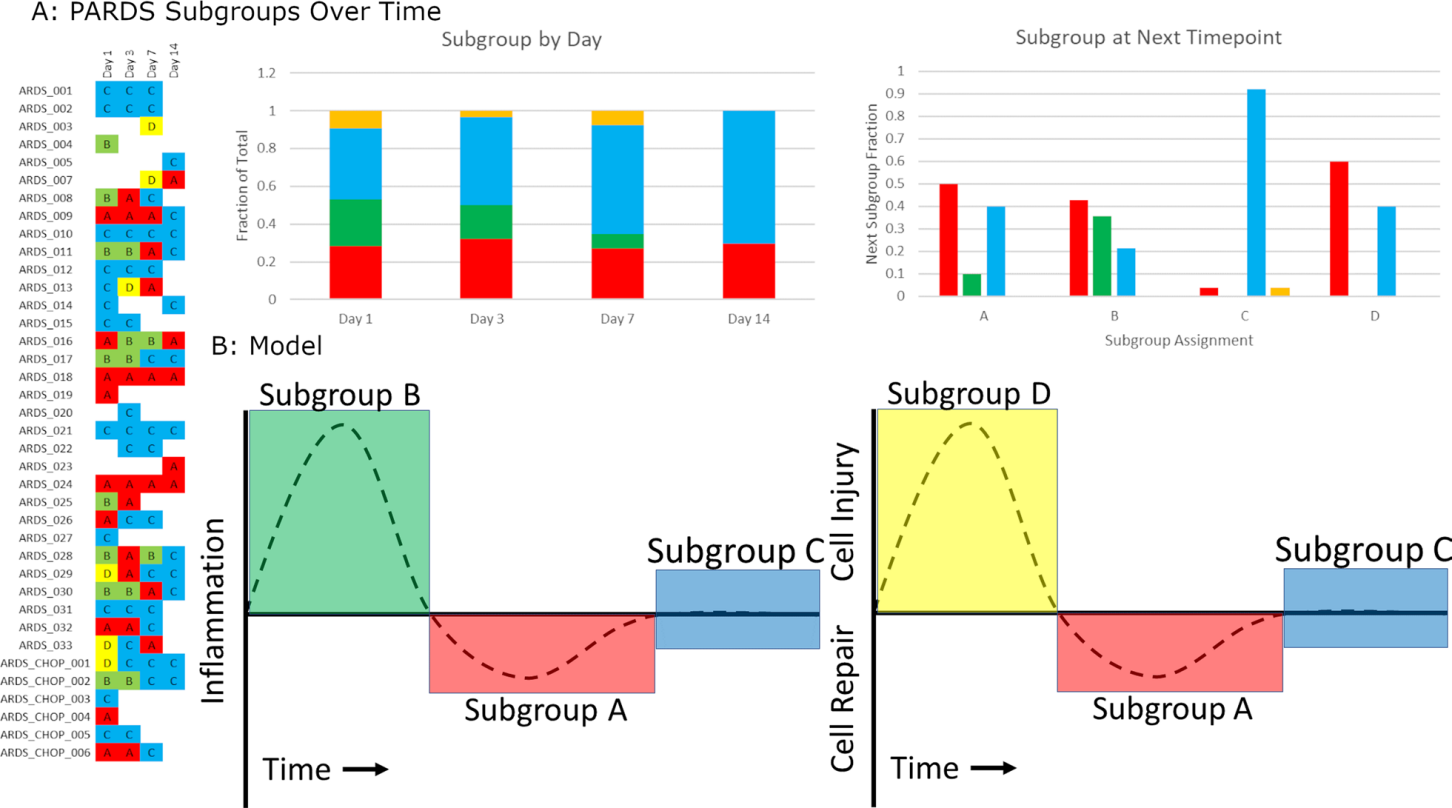
Changes in PARDS nasal transcriptome over time. **A** Subgroup assignment by subject was mapped over time. Subgroup B specimens were more common at days 1 and 3, and Subgroup C became increasing prevalent over time. In comparing the next subgroup assignment based on the current one, Subgroup C was stable while B and D transitioned to A. **B** A Model of how nasal transcriptomic subgroups transition over the course of injury, repair, and regeneration

In evaluating how PARDS Subgroups changed over time, Subgroups B and D were restricted to earlier time points with the proportion of specimens classified as Subgroup C increasing over time. Although the time intervals between specimens was not consistent, Subgroups B and D tended to transition to Subgroups A and C, and Subgroup C specimens remained consistent (Fig. 4A). Taken together, these data support a model in which Subgroups B and D represent two different modes of injury. Subgroup B is characterized by innate immune activation and ciliary dysfunction, and Subgroup D is characterized by epithelial dysfunction without inflammation. Both Subgroups transition to Subgroup A which is anti-inflammatory with increased mRNA levels of cytokines important in epithelial repair and differentiation (IL-4, IL-10, and IL-13). Subgroup C is homeostatic with restored epithelial function (Fig. 4B).

### Comparison of PARDS and control nasal transcriptomes

We reanalyzed the PARDS specimens in conjunction with 27 control specimens from 16 control subjects. Three control subjects developed lung injury (a new oxygen requirement) and one developed PARDS over the course of the study. This last subject clustered with Subgroup B, and 1, 3, and 3 specimens of the lung injury control subjects clustered with A, B, and C respectively. Among control subjects who did not develop lung injury, 3, 4, 11, and 1 clustered with A, B, C, or D respectively (Additional file 7: Fig. S6). These data suggest that the nasal transcriptome reflects lung injury in non-PARDS subjects and that the homeostatic state of the nasal epithelium is Subgroup C.

### Microbiome differences between control and two PARDS subgroups

Although we did not identify any differences in viral infection between Methylomic or Transcriptomic Subgroups (Tables 2, 4) the presence of respiratory viruses might influence the nasal transcriptome. We first visualized day 1 and all PARDS nasal specimens by infectious agent (Additional file 8: Fig. S7A, B). For this analysis, we used a shotgun metagenomic approach in which read sequences are aligned to viral and bacterial genomes. In day 1 Subgroup A, B, C, and D specimens, 67%, 42%, 24%, and 40% had a diagnosed viral infection respectively; however, in analyzing all time points, 47%, 36%, 27%, and 33% had a diagnosed viral infection—consistent with dataset analysis showing that infectious agent did not explain a significant portion of data set variation (Additional file 4: Fig. S3). For more comprehensive assessment of the microbiome, we performed shotgun metagenomics with comparison by Transcriptomic Subgroup and PARDS severity. Microbial diversity was increased in nasal brushings of PARDS subjects, not different between Transcriptomic Subgroups, and greater in moderate PARDS compared to severe or no PARDS (Additional file 8: Fig. S7C, D). There were no differences in the fraction of bacterial or viral reads between Transcriptomic Subgroups (Additional file 8: Fig. S7E, F). The dissimilarity of control vs. Transcriptomic Subgroups was significant (p < 0.001) but limited to seven bacterial an no viral species (Additional file 17: Table S6, Additional file 18: Table S7), and there were no significant microbiome differences by PARDS Severity. Neither bacteria nor specific viruses influence PARDS Transcriptomic Subgroup.

### Nasal transcriptomic subgroup predicts prolonged PARDS better than serum biomarkers

**Fig. 5:**
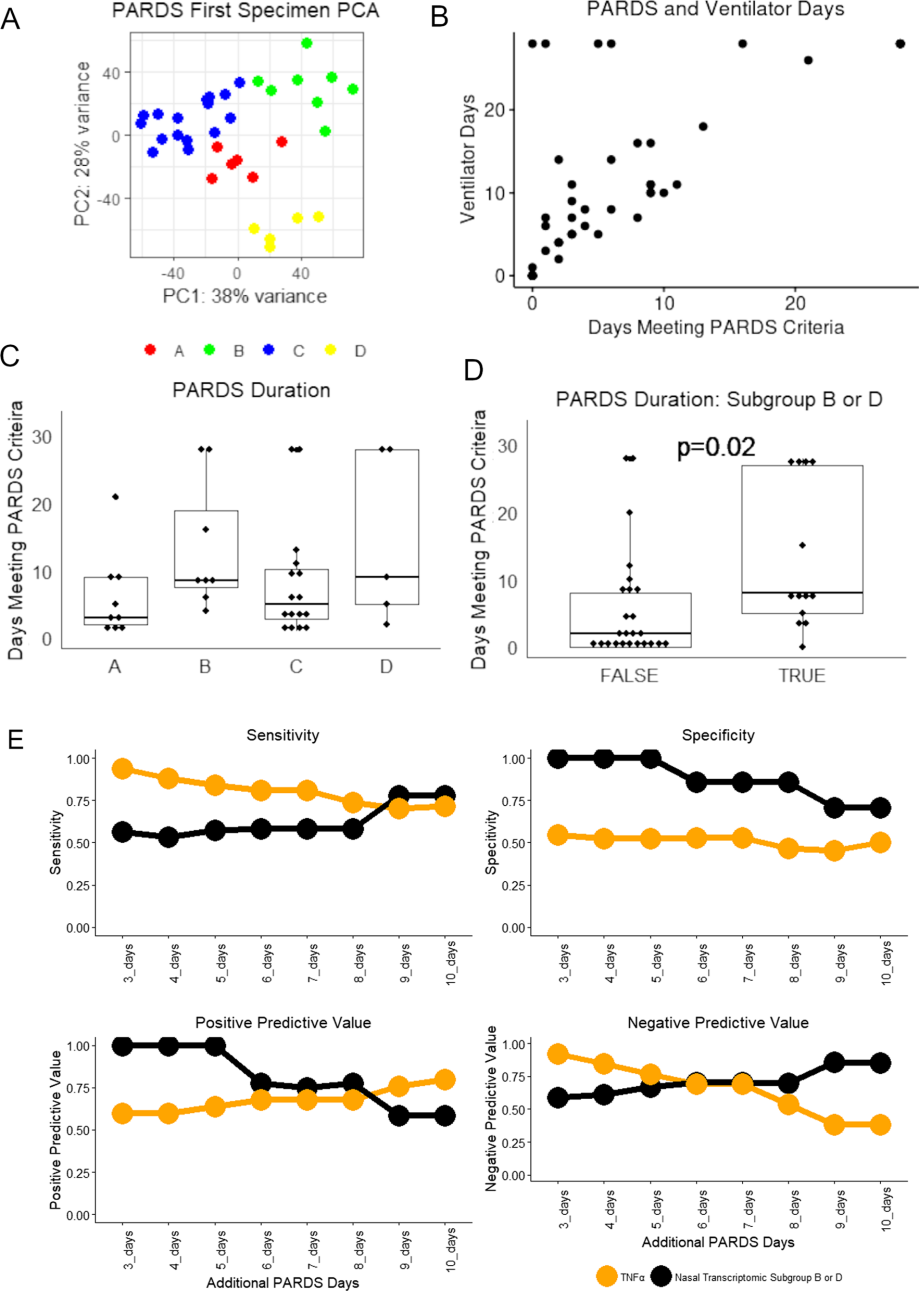
Nasal transcriptomics to predict PARDS duration. **A** Principal component plot of the nasal transcriptomes of the first specimens from PARDS subjects showing consistent classification. **B** A scatter plot of these PARDS subjects comparing PARDS duration to number of days with invasive mechanical ventilation. Many PARDS subjects remained intubated despite PARDS resolution. **C** PARDS duration tended to be longer in subjects with initial Transcriptomic Subgroup B or D assignment (Kruskal–Wallis p = 0.1). **D** Subjects with either Subgroup B or D initially had a median of 7-days longer PARDS duration. Comparison by Kruskal–Wallis shown on plot. **E** In comparing test characteristics for serum biomarkers vs. nasal transcriptomic subgroup to predict continued PARDS at 3 to 10 days, initial sensitivity and negative predictive value were high but diminished over time whereas for serum biomarkers, the reverse was true. TNFα is shown for illustrative purposes. Predictive comparisons for the other fourteen serum biomarkers can seen in Additional file 10: Fig. S9

If our inflammation and epithelial cell injury model were correct, then we would expect longer PARDS duration in Subgroups B and D. Limiting our analysis to the first available specimen (Fig. 5A), we used days meeting PARDS criteria as a primary endpoint because a sizable number of subjects remained intubated after PARDS resolution (Fig. 5B) making ventilator free days a less-reliable measure of lung injury resolution. We found that the number of days meeting PARDS criteria was greater in Nasal Transcriptomic Subgroups B&D compared to Subgroups A & C (Fig. 5C, D, median 8 vs. 2 days, p = 0.02). Thus, subjects with greater inflammation and more epithelial cell dysfunction had longer PARDS duration.

There is much interest in serum biomarker panels for ARDS and PARDS subclassification. We quantified serum levels of seventeen serum biomarkers obtained at the time of nasal brushing. None of the seventeen assayed biomarkers had a significant association with Nasal Transcriptomic Subgroup (Additional file 9: Fig. S8). To compare the ability of serum biomarkers and nasal transcriptomic subgroups to predict continued PARDS, the optimal predictive threshold for each at each time point was determined by receiver operator characteristic, and sensitivity, specificity, positive predictive value, and negative predictive value were determined for each biomarker and Nasal Transcriptomic Subgroup B or D (Fig. 5E, Additional file 10: Fig. S9). The sensitivity of Nasal Transcriptomic Subgroup B or D to predict continued PARDS at up to 7 days was as good or better than any of the assayed serum biomarkers, although there was some diminishment over time. The specificity of Subgroup B or D with regards to continued PARDS was poor. The positive predictive value of Transcriptomic Subgroup was poor for continued PARDS at day 3 (60%), but gradually improved to 80% by day 10 while the positive predictive value of all serum biomarkers diminished over time. In contrast, the negative predictive value of Transcriptomic Subgroup at 3 days was very good at 92% but diminished to 38% in predicting continued PARDS at 10 days while biomarker negative predictive value improved for longer-duration PARDS. These data indicate that serum biomarkers and nasal transcriptomics yield different information with low serum biomarkers being specific for the rapid resolution of PARDS but high serum biomarkers lacking sensitivity for continued PARDS up to 1 week later. In contrast, transcriptomics lacks specificity but has good sensitivity for continued PARDS up to 7 days and is more predictive of prolonged PARDS than serum biomarkers.

### Functional epigenetic modules in the nasal epithelium of PARDS subjects

**Fig. 6:**
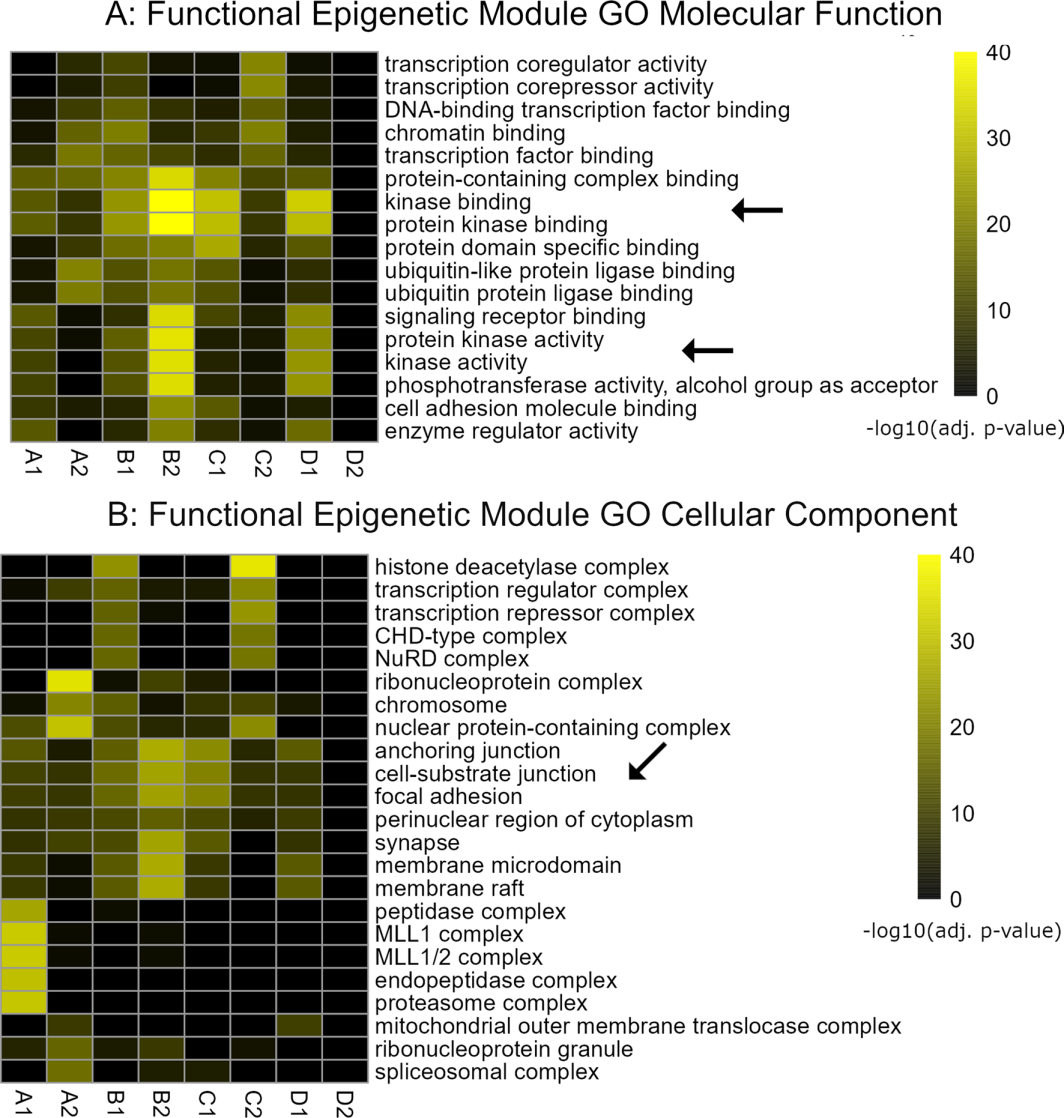
Functional epigenetic modules in PARDS. **A** We compared genes that had coordinate hypomethylation and increased expression or vice versa and combined each Methyl and Nasal Transcriptomic Subgroup to make a combined subgroup. Gene sets with coordinate epigenetic and transcriptional changes were termed functional epigenetic modules and used in gene set enrichment analysis. Combined subgroup B2 had changes in genes related to downstream inflammatory signaling. **B** Genes that encode for cell–cell and cell–matrix interface proteins had coordinate changes in methylation and gene expression in multiple combined subgroups

We compared transcriptomic and methylomic data to identify functions that were coordinately controlled at the epigenetic and transcriptional levels and performed functional enrichment for genes with increased mRNA and reduced methylated DNA or reduced mRNA and increased methylated DNA by Nasal Transcriptomic and Methylomic Subgroups. In this analysis, genes with coordinate upregulation of expression and reduced methylation are categorized as upregulated and genes with downregulation of expression and increased methylation are categorized as downregulated. The genes for each combined subgroup analysis are listed in Additional file 19: Table S8. Gene sets that show coordinated expression and methylation are referred to as functional epigenetic modules, and gene set enrichment analysis of these coordinately regulated genes is provided in Fig. 6 and Additional file 11: Fig. S10. Key findings include hypomethylation and increased expression of genes related to inflammation in combined subgroup B2 and regulation of focal adhesion and cell–matrix interaction genes in several combined subgroups.

## Discussion

Nasal transcriptomics and methylomics have been shown to reflect lung disease subtypes in asthma and lung cancer, but this study is the first to demonstrate similar capability in an acute condition like PARDS. We found that a subset of PARDS patients had hypomethylation of inflammation-related genes and that the nasal transcriptome changed in ways that would be expected during epithelial cell injury, repair, and regeneration. Subjects with either inflammation and epithelial cell dysfunction (Subgroup B) or loss of epithelial stem cell genes (Subgroup D) had longer PARDS duration.

The nasal transcriptome provides different information than is available from serum biomarkers. There was a subset of PARDS patients not identified by nasal transcriptomics. These patients had relatively short PARDS duration. Nasal transcriptomics was a better predictor of longer PARDS course than serum biomarkers consistent with the time needed for epithelial repair and regeneration. Our findings mirror the hyperinflammatory, compensatory hypoinflammatory, and resolution phases of sepsis. Further interrogation of the individual roles of inflammatory cell subtypes should be undertaken. If our findings are validated in follow-up studies, then perhaps a limited PCR panel could be developed to identify subjects at risk for prolonged PARDS for either clinical purposes or for study screening.

Our finding of four nasal transcriptomic subgroups was based on several factors and is open to criticism. First, as seen in principal component plots and reflected in our model, these groups represent a spectrum of biological processes and definitive classification is difficult. Second, our classification scheme was influenced by our post hoc observation that control subjects were largely Subgroup C, but several control subjects that developed lung injury were Subgroup B. Third, we were influenced by the genes driving principal components 1 and 2 with PC1 being largely epithelial homeostasis and repair genes and PC2 being innate inflammation genes. If one were to assign subgroup C as “normal” and A, B, and D as “abnormal” the inflammatory signal would be lost which seemed inappropriate for disease like PARDS. Fourth, we were struck by the upregulation and downregulation of cell-specific mRNAs in each group that correlated with known patterns of injury, repair, and regeneration. Whether this classification scheme is correct needs to be tested in a validation study.

Our findings regarding DNA methylation risk loci that were associated with PARDS could explain the spectrum of lung injury severity among individuals experiencing a similar insult and perhaps some factor of underlying illness that contributes to this predisposition. In comparing the expected transcriptional changes with these differentially methylated regions, genes related to inflammation and epithelial function are consistent with the role of inflammatory cells and epithelial cell dysfunction in PARDS. A prospective study of at-risk patients will be needed to test whether these differentially methylated regions represent true PARDS risk loci.

We found no association of different viral or bacterial species with different PARDS subgroups. This could be due to a relatively small number of specimens compared to the large number of pathogenic viruses and bacteria, but it could also be due to common injury-response pathways that any pathogen-specific signal was diluted by other samples at similar points in the progression through injury, repair, and regeneration. If validated prospectively, these genes might be targeted to prevent lung injury progression.

We note several limitations of this study. First, the overall number of subjects is small, but the longitudinal nature of the analysis somewhat mitigates this limitation. Second, our injury-repair-regeneration model is based solely on mRNA and not procedures such as flow cytometry. Third, although we performed correlative analysis with a small number of concurrent bronchial specimens, we did not have specimens from the alveolar compartment and can only report associations of nasal transcriptomic patterns with clinical measures of lung injury severity. Performing studies that directly compare gene expression of alveolar and conducing airway epithelial cells will be needed to quantify the degree of similarity between these two compartments. Fourth, most patients had longitudinal brushings in the same nares due to presence of a nasogastric tube. It is possible that some element of repair/regeneration signals in PARDS subjects was from the procedure itself. However, this was absent in control subjects who also had longitudinal brushings. Fifth, while control subjects were critically ill, there were substantial differences in terms of underlying genetic disorders or developmental delay and immunocompromised state. Sixth, we chose critically ill subjects without lung disease as our control subjects. We did this to try to control for the effects of critical illness itself, but it may be that critical illness induces changes in the respiratory epithelium that would not be present in patients outside the hospital. Lastly and perhaps most importantly, these findings need to be validated in a second cohort of PARDS patients and should also be tested in adult with ARDS.

## Conclusion

Nasal transcriptomic profiling in PARDS likely reflects injury, repair, and regenerative processes of the distal lung and can identify patients likely to experience a prolonged PARDS course.

## Supplementary Information

**Additional file 1**. Online supplemental methods.

**Additional file 2: Fig. S1.** Specimen Distribution by Day and Group. (A) Venn diagram of the number of evaluable RNA (red), DNA (blue) and serum (yellow) specimens for PARDS subjects on day 1. (B) The same analysis for PARDS day 3, (C) PARDS day 7, (D) PARDS day 14, (E) Control day 1, (F) Control day 3, (G) Control day 7, and (H) Control day 14.

**Additional file 3: Fig. S2.** Nasal and Bronchial Methylation Data. (A) In comparing the DNA of matched nasal and bronchial specimens, differentially methylated regions (DMRs) largely corresponded regions in or near CpG islands and (B) transcriptionally important regions of the genome. (C) Comparisons of tracheal and nasal methylation showed that except in one case, matching nasal and bronchial specimens were in the same cluster. (D) In comparing the methylation pattern of Methyl Subgroup 1 nasal specimens to Methyl Subgroup 2, Manhattan plots showed that Methyl Subgroup 2 had hypomethylation of the centromeric regions chromosomes 5, 7, 10, and 17 compared to Methyl Subgroup 2. Genes with significantly different methylation did not have adjusted p-values of less than 10–25. (E) Compared to Subgroup 1, Subgroup 2 had hypermethylation of centromeric regions of chromosomes 1 and 16.

**Additional file 4: Fig. S3.** Processing of mRNA Data (A) While there were twenty specimens that were excluded for having less than 100,000 reads, the only distribution difference between batches in read count was more counts in standard RNA-seq specimens. No specimen with less than 5,000 unique transcripts had > 100,000 reads. (B) Principal component plot showing batch effects. (C) Principal component plot after batch normalization. (D) Scree plot of dataset structure showing that seven or eight principal components best described the dataset structure of PARDS nasal specimens. Bronchial and control specimens were excluded from this analysis. (E) Eigenvalue correlation plot showing the contribution of the noted variables with each of the first eight principal components. Color scale is for r2 value. ** p < 0.01.

**Additional file 5: Fig. S4.** Comparison of Nasal and Bronchial Transcriptomes. (A) In principal component analysis of paired nasal and bronchial specimens, there was no clear clustering by either subject or collection site. (B) A Euclidean distance plot also demonstrated no clear associations by site or subject. Connecting lines show paired nasal and bronchial specimens. (C) K-means clustering plot showing the same data with yellow boxes signifying bronchial specimens and purple nasal.

**Additional file 6: Fig. S5.** Volcano Plots of Differentially Expressed Genes. Nasal Transcriptomic Subgroups A, B, C, and D volcano plots of genes with increased or decreased mRNA abundance with highlighting of several inflammatory and epithelial function-related genes.

**Additional file 7: Fig. S6.** Comparison of PARDS Nasal Transcriptomic Subgroups with Controls. (A) PARDS and control specimens were re-processed together with similar clustering of Subgroup A, B, C, and D specimens. While most control subjects did not develop lung injury, one developed mild ARDS and several developed lung injury (defined as a new oxygen requirement of > 24 h). Control specimens were largely clustered with Subgroup C and specimens from subjects who developed ARDS or lung injury were clustered with B or A. (B) A k-means clustering tree of control and PARDS specimens showed that subgroups B and D remained largely consistent but some of the similarities between groups A and C were diminished.

**Additional file 8: Fig. S7.** Metagenomic Assessment of PARDS Nasal Transcriptomic Subgroups. (A) There was no consistent pattern of viral or bacterial infection with PARDS Nasal Transcriptomic Subgroup in combined analysis. (B) Nor was there any clear association when limiting analysis to initial specimens. (C) In metagenomic analysis, Shannon diversity index values identified increased diversity of specimens collected at a time of moderate PARDS compared to both severe and no PARDS. These comparisons were not significant when analyzed by collection day. (D) Microbial diversity either tended or was significantly elevated in PARDS subgroups compared to control, but again was not significant when analyzed by collection day. Comparison is by Wilcoxon rank sum test. (E) There were no differences in the percentage of reads mapping to bacterial or (F) viral genomes by Transcriptomic Subgroup when analyzed as a group or by day.

**Additional file 9: Fig. S8.** Serum Biomarkers by PARDS Nasal Transcriptomic Subgroup. After quantification of 17 ARDS- and PARDS-associated serum biomarkers, there were no significant differences in levels by Transcriptomic subgroup when analyzed by Kruskal–Wallis test.

**Additional file 10: Fig. S9.** Test characteristics of Initial Nasal Transcriptomic Subgroup vs. Seventeen Serum Biomarkers for Predicting Continued PARDS at Different Days. (A) The sensitivity of Nasal Transcriptomic Subgroup B or D for predicting continued PARDS at days 3–5 was high but diminished over time. This was in contrast to all of the other serum biomarkers which showed the opposite pattern. (B) The specificity of Nasal Transcriptomic Subgroup B or D for continued PARDS was low and inferior to all serum biomarkers assayed. (C) The positive predictive value of Nasal Transcriptomic Subgroup was poor for predicting short-term but good for predicting long-term continued PARDS. Again, this was the opposite of serum biomarkers. (D) The negative predictive value of initial Transcriptomic subgroup was good for early but poor for later PARDS.

**Additional file 11: Fig. S10.** Additional Functional Epigenetic Module Information. (A) Gene set enrichment analysis of genes with coordinate changes in methylation and expression identified specific biological processes, (B) gene families, (C) and pathways that may be regulated at the epigenetic level.

**Additional file 12: Table S1.** Specimen Log.

**Additional file 13: Table S2.** Differentially Methylated Transcription Start Sites Bronchial vs. Nasal.

**Additional file 14: Table S3.** Differentially Methylated Transcription Start Sites Subgroup 1 vs Subgroup 2.

**Additional file 15: Table S4.** Principal Component Genes.

**Additional file 16: Table S5.** Principal Component GO Molecular Function.

**Additional file 17: Table S6.** Differentially Expressed Genes.

**Additional file 18: Table S7.** Differentially Abundant Microbial Species Summary.

**Additional file 19: Table S8.** Differentially Abundant Microbial Species.

## Data Availability

Datasets are available on Geodatasets as GSE192364 and GSE192926. Other data are available by contacting the corresponding author.
