# Supplementary material for: Multi-omic characterization of pediatric ARDS via nasal brushings
Source: Respir Res. 2022 Jul 9;23:181. doi: 10.1186/s12931-022-02098-3 (PMC9270778; doi:10.1186/s12931-022-02098-3)
Supplement: Supplementary file 1 — Additional file 1. Online supplemental methods. [file 12931_2022_2098_MOESM1_ESM.docx]

Multi-omic Characterization of Pediatric ARDS via Nasal Brushings-Supplement

James Garrett Williams MD^1^, Rashika Joshi MD^1^, David Haslam^2,3^, Nadir Yehya^4,5^, Rhonda L. Jones RN^1^, Aditi Paranjpe MS^6^, Mario Pujato PhD^7^, Krishna M. Roskin PhD^3,6^, Patrick M. Lahni MS^1^, Hector R. Wong MD^1,3^ and Brian M. Varisco MD^1,3^*

## Author details

1 Critical Care Medicine, Cincinnati Children’s Hospital Medical Center

2 Infectious Diseases, Cincinnati Children’s Hospital Medical Center

3 University of Cincinnati College of Medicine

4 Critical Care Medicine, Children’s Hospital of Philadelphia

5 Perlman School of Medicine, University of Philadelphia

6 Biomedical Informatics, Cincinnati Children’s Hospital Medical Center

7 Production Informatics, AstraZeneca Oncology Division

*Correspondence:

Brian Varisco MD, Cincinnati Children’s Hospital Medical Center

3333 Burnet Avenue, MLC 7006

Cincinnati, OH 45229

[brian.varisco@cchmc.org](mailto:brian.varisco@cchmc.org)

## Author Contributions

1-Substantial contributions to the conception or design of the work

2-Acquisition, analysis, or interpretation of data for the work

3-Drafting the work or revising it critically for important intellectual content

4-Final approval of the version to be published

5-Agreement to be accountable for all aspects of the work in ensuring that questions related to the accuracy or integrity of any part of the work are appropriately investigated and resolved.

JGW-1,2,3,4,5 RJ-2,3,4,5 NY-1,3,4,5 RLJ-1,3,4,5 AP-1,3,4,5

MP-2,3,4,5 KR-2,3,4,5 PML-1,3,4,5 HRW-1,3,4,5 BMV-1,2,3,4,5

## Financial Support

2020 SCCM Discovery Award, Society of Critical Care Medicine’s Research Discovery, the Critical Care Research Network (Varisco)

2019 Center for Pediatric Genomics Pilot Grant (Varisco)

R01HL141229 (Varisco)

## Disclosures / Conflict of Interest

The authors declare no conflicts of interest.

## Key Words

Transcriptomics, Subclassification, Acute Lung Injury, Pediatric Acute Respiratory Distress Syndrome

# ONLINE SUPPLEMENTAL METHODS

## Bioinformatic analysis of RNA

RNA reads were aligned to GRCh38 and count matrices generated using STAR v2.6.7a [1]. After removal of ribosomal and mitochondrial genes, variance stabilizing transformation, batch normalization, and centering, differential gene expression was assayed using DESeq2 v1.34.0 [2], limma v3.50-0 [3], PCAtools v2.6.0 [4], EnhancedVolcano v1.12.0 [5], jackstraw v1.3.1 [6], and VennDiagram v1.7.1 [7]. Gene set enrichment analysis was performed using ToppGene [8] and top terms visualized using pheatmap v1.0.12 [9].

## Bioinformatic Analysis of methylated DNA and Comparison with mRNA-Seq Data

DNA sequences were aligned to GRCh38 and methylated and non-methylated loci counted using Bismark v0.22.3 [10]. Coverage files were analyzed and assigned to gene features using methylKit v1.20.0 [11] and ToppGene was used for gene set enrichment analysis. Methylation and gene expression matrixes were analyzed by the FEM package v3.15.0 [12] to generate lists of genes with coordinated differences in methylation and gene expression. For each Methyl Subgroup and Transcriptomic Subgroup combination, the matrices were compared against those of specimens with those exclusive of that combination (e.g. the methylation and gene expression matrixes of samples in A1 were compared against those of B2, C2, and D2). These gene lists were used for gene set enrichment analysis with ToppGene.

## Metagenomic Analysis

Using a shotgun metagenomic approach bacterial and viral RNA sequences were aligned bacterial and viral genomes using Kraken2 v2.1.2 [13] and normalized reads quantified using vegan v2.5.7 [14]. Shannon bacterial diversity indices were determined for each specimen and compared between Subgroups and over time. Specific species with differential abundance between control and PARDS Transcriptomic Subgroups were determined using Wilcoxon testing after filtering for species with at least 100 unique reads.

## Serum Protein Quantification and Predictive Statistic Comparisons

Serum samples were analyzed in duplicate for Angiopoietin2 (ANG2), Granzyme B (GrB), Intercellular Adhesion Molecule1 (ICAM1), Interferon-γ (IFNγ), Interleukin-6 (IL6), IL8, IL10, IL17, IL18, Surfactant Protein D, Tumor Necrosis Factorα (TNF-α), TNF Receptor Soluble Factor 1A (TNFSF1A), and RAGE using R&D Systems reagents and a Luminex 200 instrument. Optimized thresholds for continued to meet PARDS criteria at days 3 to 10 were determined using pROC [15] with determination of sensitivity, specificity, positive predictive value, and negative predictive value based on that threshold.

# ONLINE DATA SUPPLEMENT

1: R-code for mRNA-Seq analysis

2: R-code for methyl-Seq analysis

3: R-code for functional epigenetic modules analysis

4: R-code for metagenomic analysis

# Supplemental Tables

Supplemental Table 1: Specimen Log

Supplemental Table 2: Differentially Methylated Transcription Start Sites Bronchial vs. Nasal

Supplemental Table 3: Differentially Methylated Transcription Start Sites Subgroup 1 vs Subgroup 2

Supplemental Table 4: Principal Component Genes

Supplemental Table 5: Principal Component GO Molecular Function

Supplemental Table 6: Differentially Expressed Genes

Supplemental Table 7: Differentially Abundant Microbial Species Summary

Supplemental Table 8: Differentially Abundant Microbial Species

Supplemental Table 9: Functional Epigenetic Module Genes

# SUPPLEMENTAL FIGURE LEGENDS

## Supplemental Figure 1: Specimen Distribution by Day and Group

1. Venn diagram of the number of evaluable RNA (red), DNA (blue) and serum (yellow) specimens for PARDS subjects on day 1.
2. The same analysis for PARDS day 3,
3. PARDS day 7,
4. PARDS day 14,
5. Control day 1,
6. Control day 3,
7. Control day 7, and
8. Control day 14

## Supplemental Figure 2: Nasal and Bronchial Methylation Data

1. In comparing the DNA of matched nasal and bronchial specimens, differentially methylated regions (DMRs) largely corresponded regions in or near CpG islands and
2. transcriptionally important regions of the genome.
3. Comparisons of tracheal and nasal methylation showed that except in one case, matching nasal and bronchial specimens were in the same cluster.
4. In comparing the methylation pattern of Methyl Subgroup 1 nasal specimens to Methyl Subgroup 2, Manhattan plots showed that Methyl Subgroup 2 had hypomethylation of the centromeric regions chromosomes 5, 7, 10, and 17 compared to Methyl Subgroup 2. Genes with significantly different methylation did not have adjusted p-values of less than 10^-25^.
5. Compared to Subgroup 1, Subgroup 2 had hypermethylation of centromeric regions of chromosomes 1 and 16.

## Supplemental Figure 3: Processing of mRNA Data

1. While there were twenty specimens that were excluded for having less than 100,000 reads, the only distribution difference between batches in read count was more counts in standard RNA-seq specimens. No specimen with less than 5,000 unique transcripts had >100,000 reads.
2. Principal component plot showing batch effects.
3. Principal component plot after batch normalization.
4. Scree plot of dataset structure showing that seven or eight principal components best described the dataset structure of PARDS nasal specimens. Bronchial and control specimens were excluded from this analysis.
5. Eigenvalue correlation plot showing the contribution of the noted variables with each of the first eight principal components. Color scale is for r^2^ value. ** p<0.01.

## Supplemental Figure 4: Comparison of Nasal and Bronchial Transcriptomes

1. In principal component analysis of paired nasal and bronchial specimens, there was no clear clustering by either subject or collection site.
2. A Euclidean distance plot also demonstrated no clear associations by site or subject. Connecting lines show paired nasal and bronchial specimens.
3. K-means clustering plot showing the same data with yellow boxes signifying bronchial specimens and purple nasal.

## Supplemental Figure 5: Volcano Plots of Differentially Expressed Genes

Nasal Transcriptomic Subgroups A, B, C, and D volcano plots of genes with increased or decreased mRNA abundance with highlighting of several inflammatory and epithelial function-related genes.

## Supplemental Figure 6: Comparison of PARDS Nasal Transcriptomic Subgroups with Controls

1. PARDS and control specimens were re-processed together with similar clustering of Subgroup A, B, C, and D specimens. While most control subjects did not develop lung injury, one developed mild ARDS and several developed lung injury (defined as a new oxygen requirement of > 24 hours). Control specimens were largely clustered with Subgroup C and specimens from subjects who developed ARDS or lung injury were clustered with B or A.
2. A k-means clustering tree of control and PARDS specimens showed that subgroups B and D remained largely consistent but some of the similarities between groups A and C were diminished.

## Supplemental Figure 7: Metagenomic Assessment of PARDS Nasal Transcriptomic Subgroups

1. There was no consistent pattern of viral or bacterial infection with PARDS Nasal Transcriptomic Subgroup in combined analysis.
2. Nor was there any clear association when limiting analysis to initial specimens.
3. In metagenomic analysis, Shannon diversity index values identified increased diversity of specimens collected at a time of moderate PARDS compared to both severe and no PARDS. These comparisons were not significant when analyzed by collection day.
4. Microbial diversity either tended or was significantly elevated in PARDS subgroups compared to control, but again was not significant when analyzed by collection day. Comparison is by Wilcoxon rank sum test.
5. There were no differences in the percentage of reads mapping to bacterial or
6. viral genomes by Transcriptomic Subgroup when analyzed as a group or by day.

## Supplemental Figure 8: Serum Biomarkers by PARDS Nasal Transcriptomic Subgroup

After quantification of 17 ARDS- and PARDS-associated serum biomarkers, there were no significant differences in levels by Transcriptomic subgroup when analyzed by Kruskal-Wallis test.

## Supplemental Figure 9: Test characteristics of Initial Nasal Transcriptomic Subgroup vs. Seventeen Serum Biomarkers for Predicting Continued PARDS at Different Days

1. The sensitivity of Nasal Transcriptomic Subgroup B or D for predicting continued PARDS at days 3-5 was high but diminished over time. This was in contrast to all of the other serum biomarkers which showed the opposite pattern.
2. The specificity of Nasal Transcriptomic Subgroup B or D for continued PARDS was low and inferior to all serum biomarkers assayed.
3. The positive predictive value of Nasal Transcriptomic Subgroup was poor for predicting short-term but good for predicting long-term continued PARDS. Again, this was the opposite of serum biomarkers.
4. The negative predictive value of initial Transcriptomic subgroup was good for early but poor for later PARDS

## Supplemental Figure 10: Additional Functional Epigenetic Module Information

1. Gene set enrichment analysis of genes with coordinate changes in methylation and expression identified specific biological processes,
2. gene families,
3. and pathways that may be regulated at the epigenetic level.

# REFERENCES

1. Dobin A, Davis CA, Schlesinger F, Drenkow J, Zaleski C, Jha S, et al. STAR: ultrafast universal RNA-seq aligner. Bioinformatics. 2013;29:15–21.

2. Love MI, Huber W, Anders S. Moderated estimation of fold change and dispersion for RNA-seq data with DESeq2. Genome Biology. 2014;15:550.

3. Ritchie ME, Phipson B, Wu D, Hu Y, Law CW, Shi W, et al. limma powers differential expression analyses for RNA-sequencing and microarray studies. Nucleic Acids Res. 2015;43:e47.

4. Blighe K, Lun, Aaron. PCAtools: everything Principal Components Analysis [Internet]. 2020 [cited 2020 Aug 4]. Available from: https://github.com/kevinblighe/PCAtools

5. Blighe K. EnhancedVolcano: Publication-ready volcano plots with enhanced colouring and labeling [Internet]. 2019. Available from: https://github.com/kevinblighe/EnhancedVolcano

6. Neo Christopher Chung, John D. Storey, Wei Hao. jackstraw: Statistical Inference for Unsupervised Learning in jackstraw: Statistical Inference for Unsupervised Learning [Internet]. [cited 2020 Aug 4]. Available from: https://rdrr.io/cran/jackstraw/man/jackstraw.html

7. Chen H. VennDiagram: Generate High-Resolution Venn and Euler Plots [Internet]. 2018 [cited 2020 Jun 18]. Available from: https://CRAN.R-project.org/package=VennDiagram

8. Chen J, Bardes EE, Aronow BJ, Jegga AG. ToppGene Suite for gene list enrichment analysis and candidate gene prioritization. Nucleic Acids Research. 2009;37:W305-11.

9. Kolde R. pheatmap: Pretty Heatmaps [Internet]. 2019 [cited 2020 Apr 28]. Available from: https://CRAN.R-project.org/package=pheatmap

10. Krueger F, Andrews SR. Bismark: a flexible aligner and methylation caller for Bisulfite-Seq applications. Bioinformatics. 2011;27:1571–2.

11. Akalin A, Kormaksson M, Li S, Garrett-Bakelman FE, Figueroa ME, Melnick A, et al. methylKit: a comprehensive R package for the analysis of genome-wide DNA methylation profiles. Genome Biol. 2012;13:R87.

12. Jiao Y, Widschwendter M, Teschendorff AE. A systems-level integrative framework for genome-wide DNA methylation and gene expression data identifies differential gene expression modules under epigenetic control. Bioinformatics. 2014;30:2360–6.

13. Wood D, Lu J, Langmead B. Improved metagenomic analysis with Kraken 2 | Genome Biology | Full Text. Genome Biology [Internet]. 2019 [cited 2021 Jun 25];20. Available from: https://genomebiology.biomedcentral.com/articles/10.1186/s13059-019-1891-0

14. Oksanen J, Blanchet FG, Friendly M, Kindt R, Legendre P, McGlinn D, et al. vegan community ecology package version 2.5-7 November 2020 [Internet]. 2020. Available from: http://CRAN.Rproject.org/pachage=vegan

15. Robin X, Turck N, Hainard A, Tiberti N, Lisacek F, Sanchez J-C, et al. pROC: an open-source package for R and S+ to analyze and compare ROC curves. BMC Bioinformatics. 2011;12:77.
