# Supplementary figures and images for: Multi-omic characterization of pediatric ARDS via nasal brushings
Source: Respir Res. 2022 Jul 9;23:181. doi: 10.1186/s12931-022-02098-3 (PMC9270778; doi:10.1186/s12931-022-02098-3)

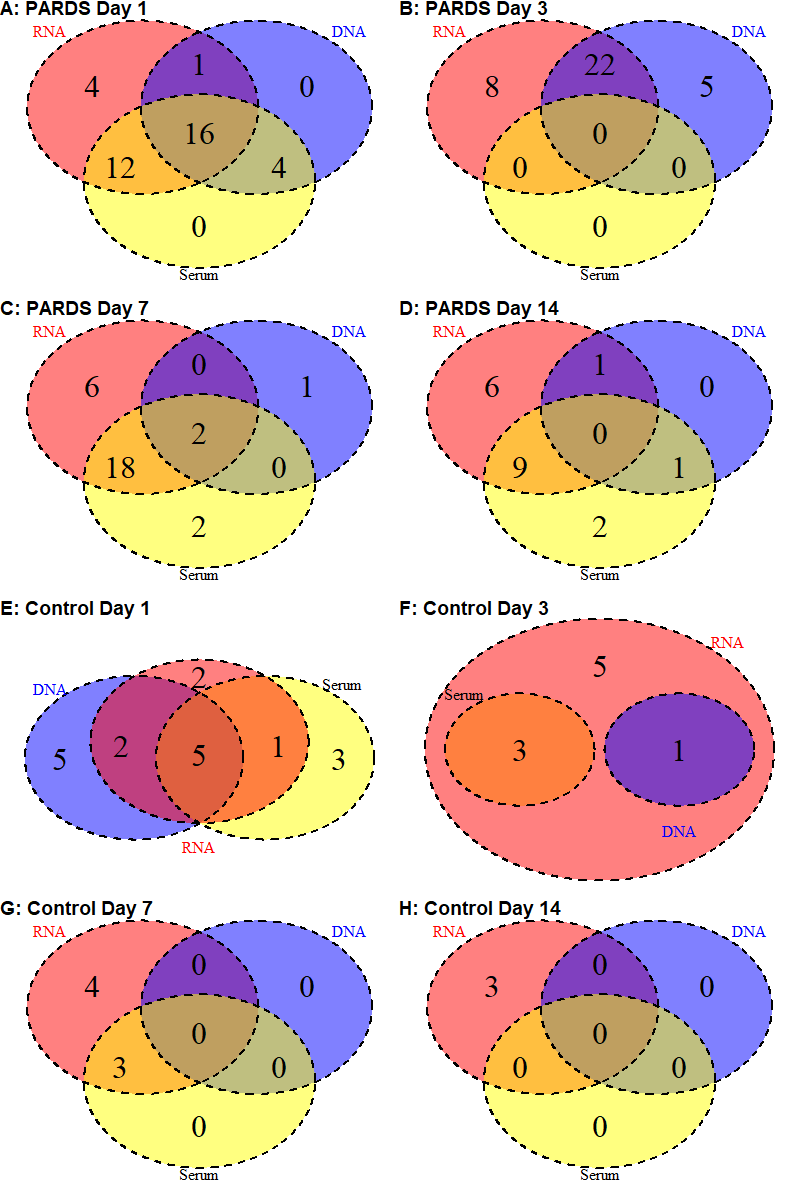

Supplement: Supplementary file 2 — Additional file 2: Fig. S1. Specimen Distribution by Day and Group. (A) Venn diagram of the number of evaluable RNA (red), DNA (blue) and serum (yellow) specimens for PARDS subjects on day 1. (B) The same analysis for PARDS day 3, (C) PARDS day 7, (D) PARDS day 14, (E) Control day 1, (F) Control day 3, (G) Control day 7, and (H) Control day 14. [file 12931_2022_2098_MOESM2_ESM.png]

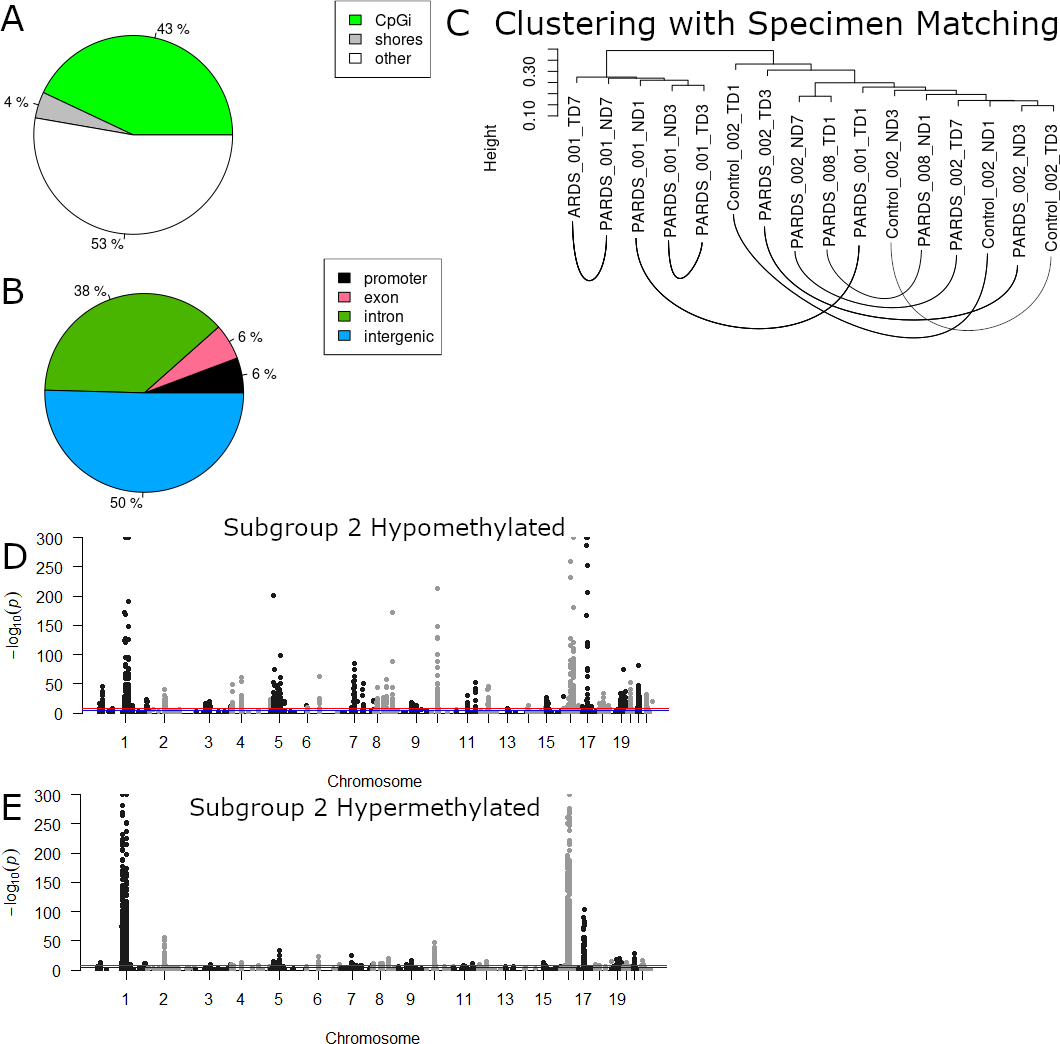

Supplement: Supplementary file 3 — Additional file 3: Fig. S2. Nasal and Bronchial Methylation Data. (A) In comparing the DNA of matched nasal and bronchial specimens, differentially methylated regions (DMRs) largely corresponded regions in or near CpG islands and (B) transcriptionally important regions of the genome. (C) Comparisons of tracheal and nasal methylation showed that except in one case, matching nasal and bronchial specimens were in the same cluster. (D) In comparing the methylation pattern of Methyl Subgroup 1 nasal specimens to Methyl Subgroup 2, Manhattan plots showed that Methyl Subgroup 2 had hypomethylation of the centromeric regions chromosomes 5, 7, 10, and 17 compared to Methyl Subgroup 2. Genes with significantly different methylation did not have adjusted p-values of less than 10–25. (E) Compared to Subgroup 1, Subgroup 2 had hypermethylation of centromeric regions of chromosomes 1 and 16. [file 12931_2022_2098_MOESM3_ESM.png]

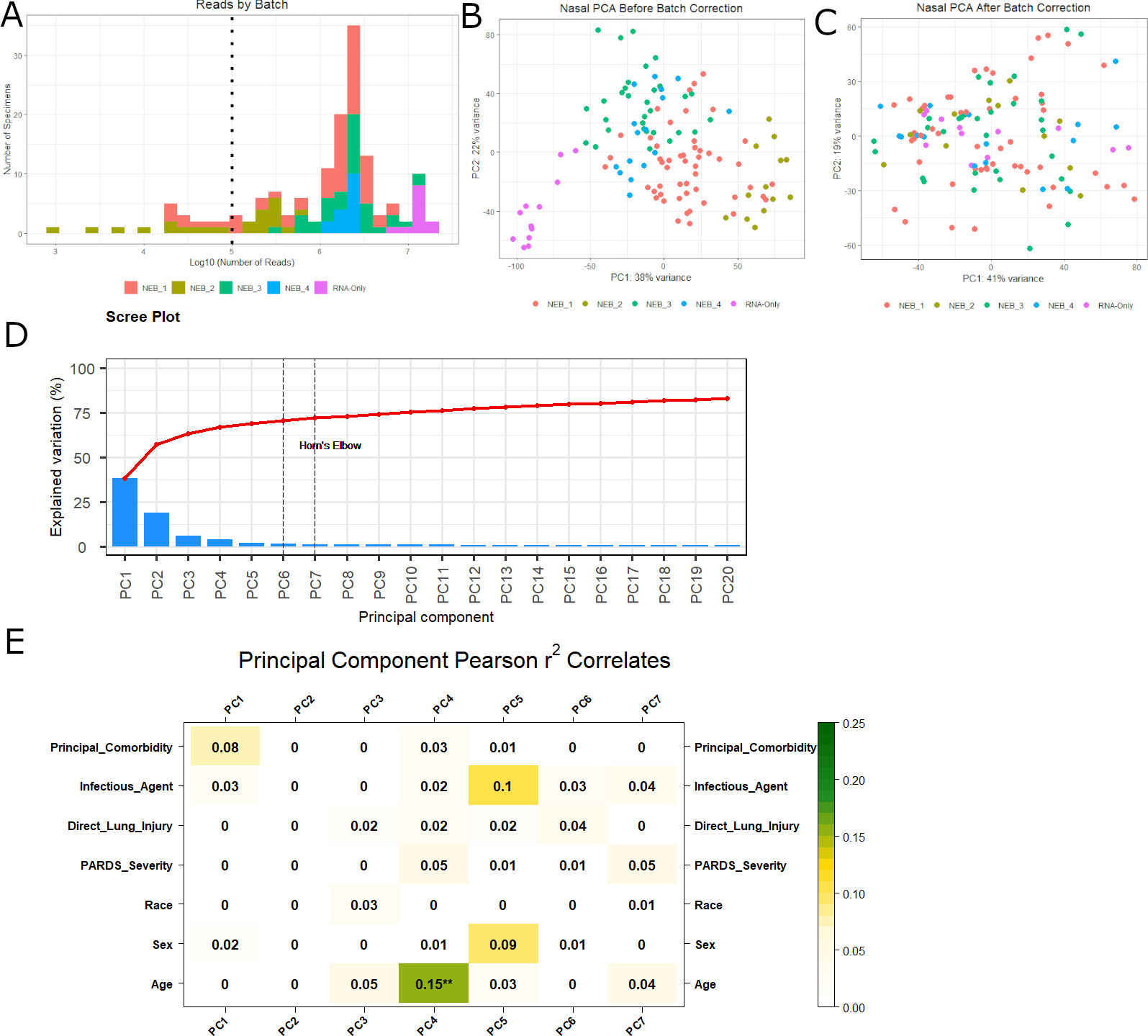

Supplement: Supplementary file 4 — Additional file 4: Fig. S3. Processing of mRNA Data (A) While there were twenty specimens that were excluded for having less than 100,000 reads, the only distribution difference between batches in read count was more counts in standard RNA-seq specimens. No specimen with less than 5,000 unique transcripts had > 100,000 reads. (B) Principal component plot showing batch effects. (C) Principal component plot after batch normalization. (D) Scree plot of dataset structure showing that seven or eight principal components best described the dataset structure of PARDS nasal specimens. Bronchial and control specimens were excluded from this analysis. (E) Eigenvalue correlation plot showing the contribution of the noted variables with each of the first eight principal components. Color scale is for r2 value. ** p < 0.01. [file 12931_2022_2098_MOESM4_ESM.png]

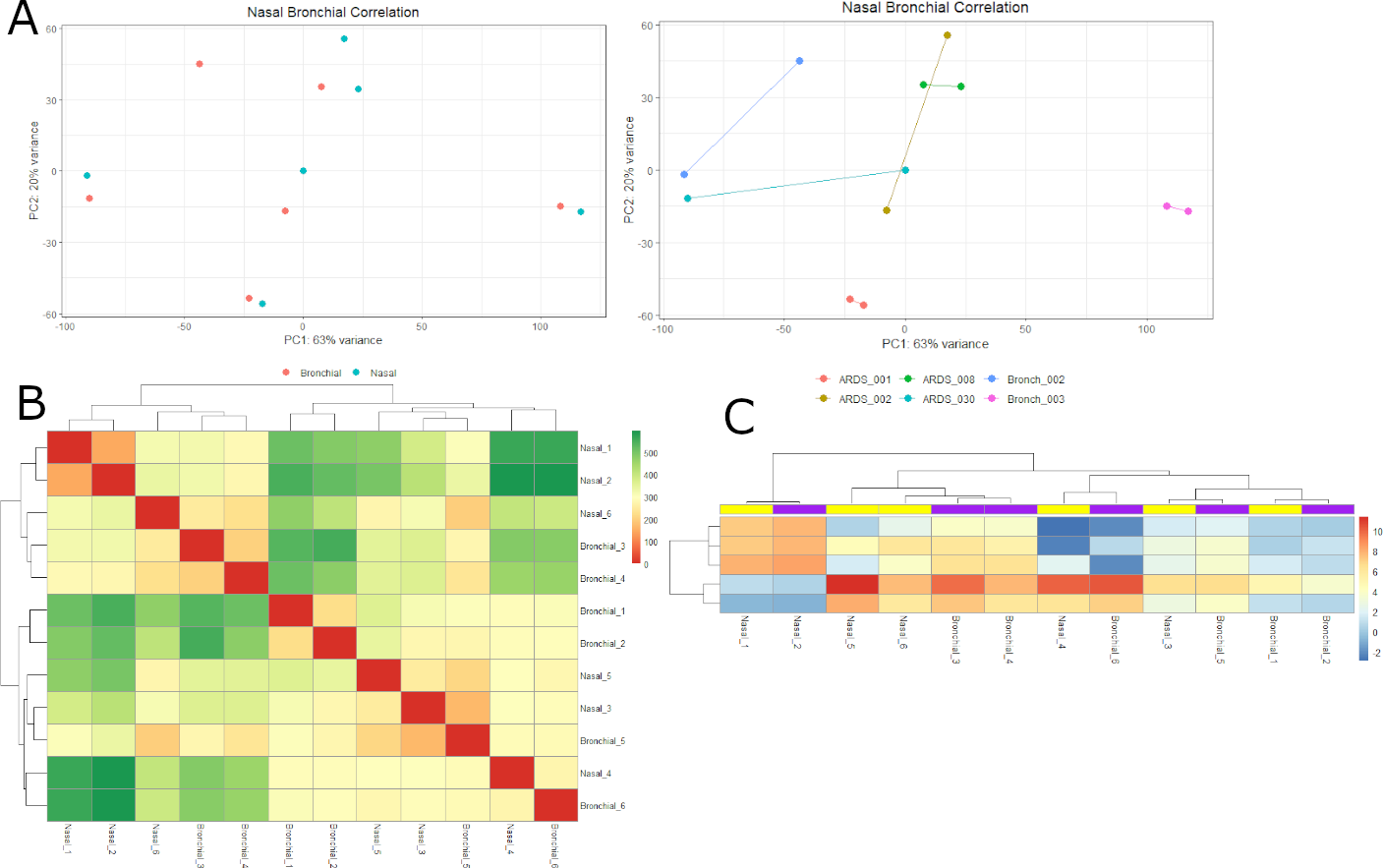

Supplement: Supplementary file 5 — Additional file 5: Fig. S4. Comparison of Nasal and Bronchial Transcriptomes. (A) In principal component analysis of paired nasal and bronchial specimens, there was no clear clustering by either subject or collection site. (B) A Euclidean distance plot also demonstrated no clear associations by site or subject. Connecting lines show paired nasal and bronchial specimens. (C) K-means clustering plot showing the same data with yellow boxes signifying bronchial specimens and purple nasal. [file 12931_2022_2098_MOESM5_ESM.png]

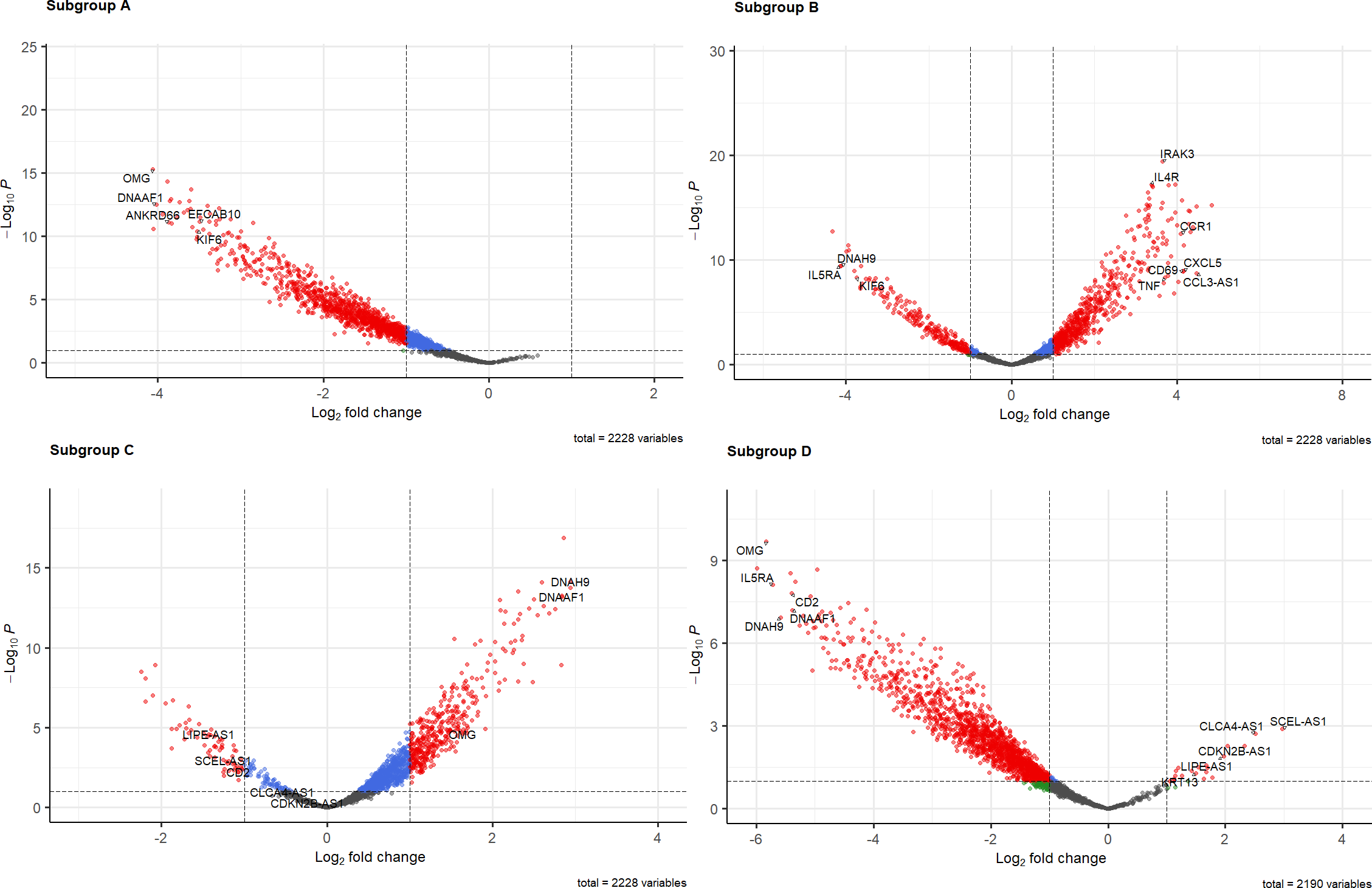

Supplement: Supplementary file 6 — Additional file 6: Fig. S5. Volcano Plots of Differentially Expressed Genes. Nasal Transcriptomic Subgroups A, B, C, and D volcano plots of genes with increased or decreased mRNA abundance with highlighting of several inflammatory and epithelial function-related genes. [file 12931_2022_2098_MOESM6_ESM.png]

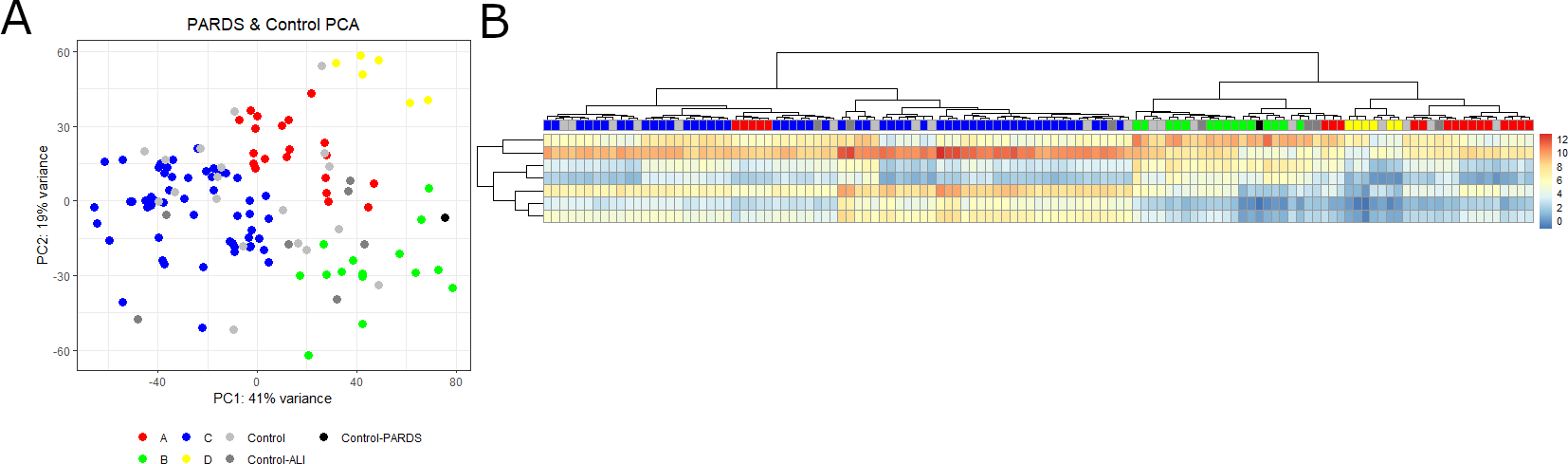

Supplement: Supplementary file 7 — Additional file 7: Fig. S6. Comparison of PARDS Nasal Transcriptomic Subgroups with Controls. (A) PARDS and control specimens were re-processed together with similar clustering of Subgroup A, B, C, and D specimens. While most control subjects did not develop lung injury, one developed mild ARDS and several developed lung injury (defined as a new oxygen requirement of > 24 h). Control specimens were largely clustered with Subgroup C and specimens from subjects who developed ARDS or lung injury were clustered with B or A. (B) A k-means clustering tree of control and PARDS specimens showed that subgroups B and D remained largely consistent but some of the similarities between groups A and C were diminished. [file 12931_2022_2098_MOESM7_ESM.png]

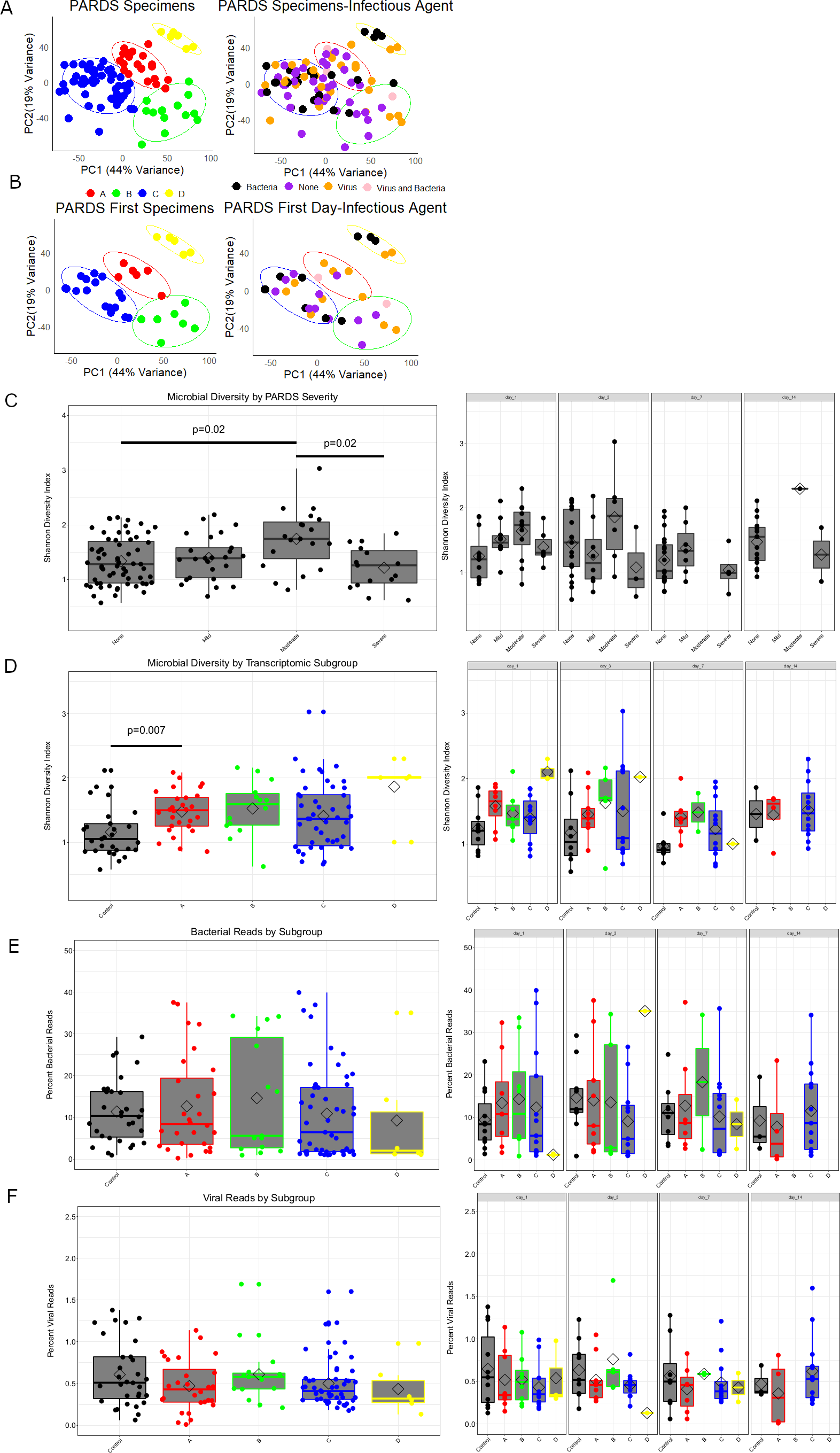

Supplement: Supplementary file 8 — Additional file 8: Fig. S7. Metagenomic Assessment of PARDS Nasal Transcriptomic Subgroups. (A) There was no consistent pattern of viral or bacterial infection with PARDS Nasal Transcriptomic Subgroup in combined analysis. (B) Nor was there any clear association when limiting analysis to initial specimens. (C) In metagenomic analysis, Shannon diversity index values identified increased diversity of specimens collected at a time of moderate PARDS compared to both severe and no PARDS. These comparisons were not significant when analyzed by collection day. (D) Microbial diversity either tended or was significantly elevated in PARDS subgroups compared to control, but again was not significant when analyzed by collection day. Comparison is by Wilcoxon rank sum test. (E) There were no differences in the percentage of reads mapping to bacterial or (F) viral genomes by Transcriptomic Subgroup when analyzed as a group or by day. [file 12931_2022_2098_MOESM8_ESM.png]

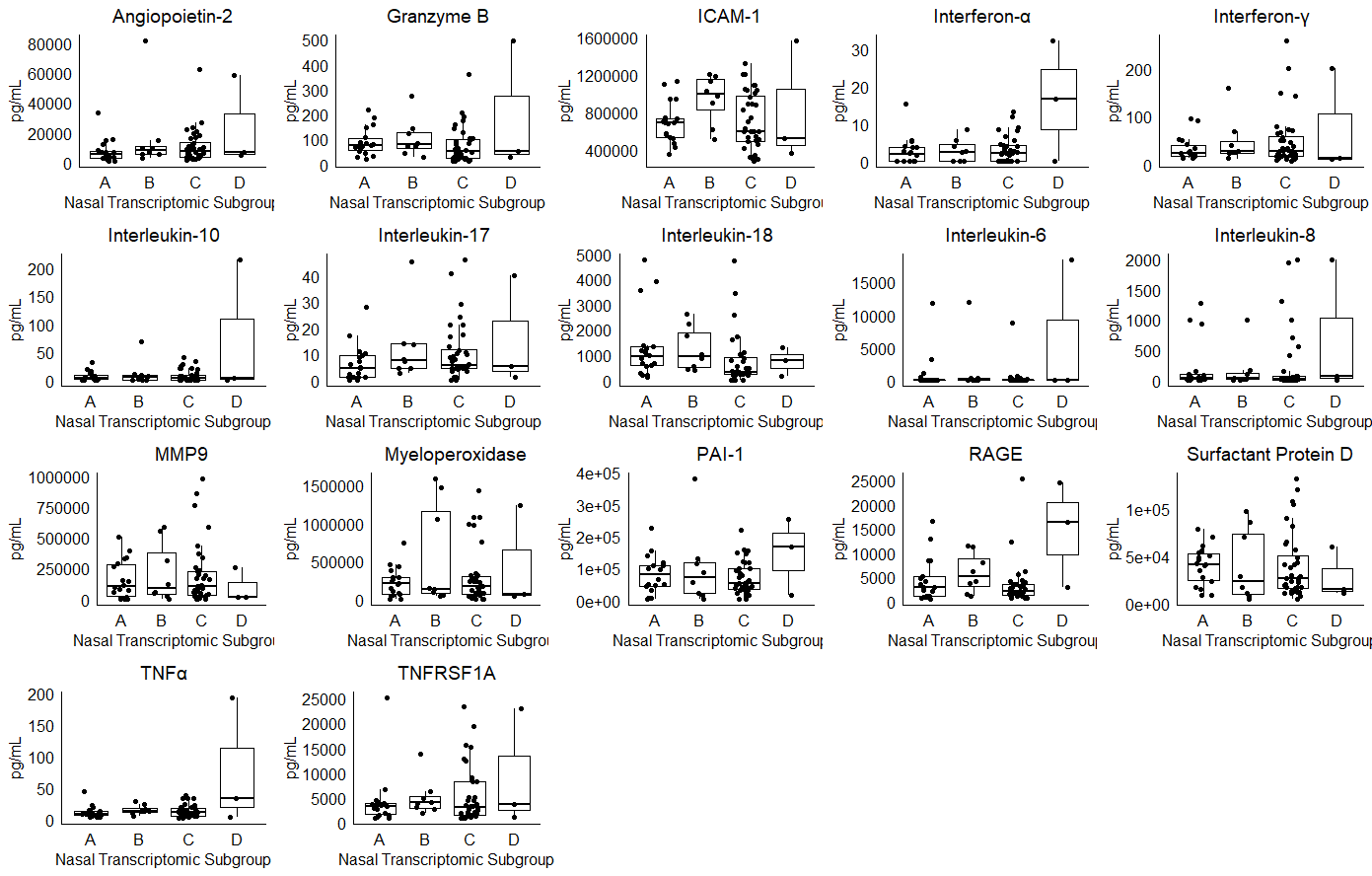

Supplement: Supplementary file 9 — Additional file 9: Fig. S8. Serum Biomarkers by PARDS Nasal Transcriptomic Subgroup. After quantification of 17 ARDS- and PARDS-associated serum biomarkers, there were no significant differences in levels by Transcriptomic subgroup when analyzed by Kruskal–Wallis test. [file 12931_2022_2098_MOESM9_ESM.png]

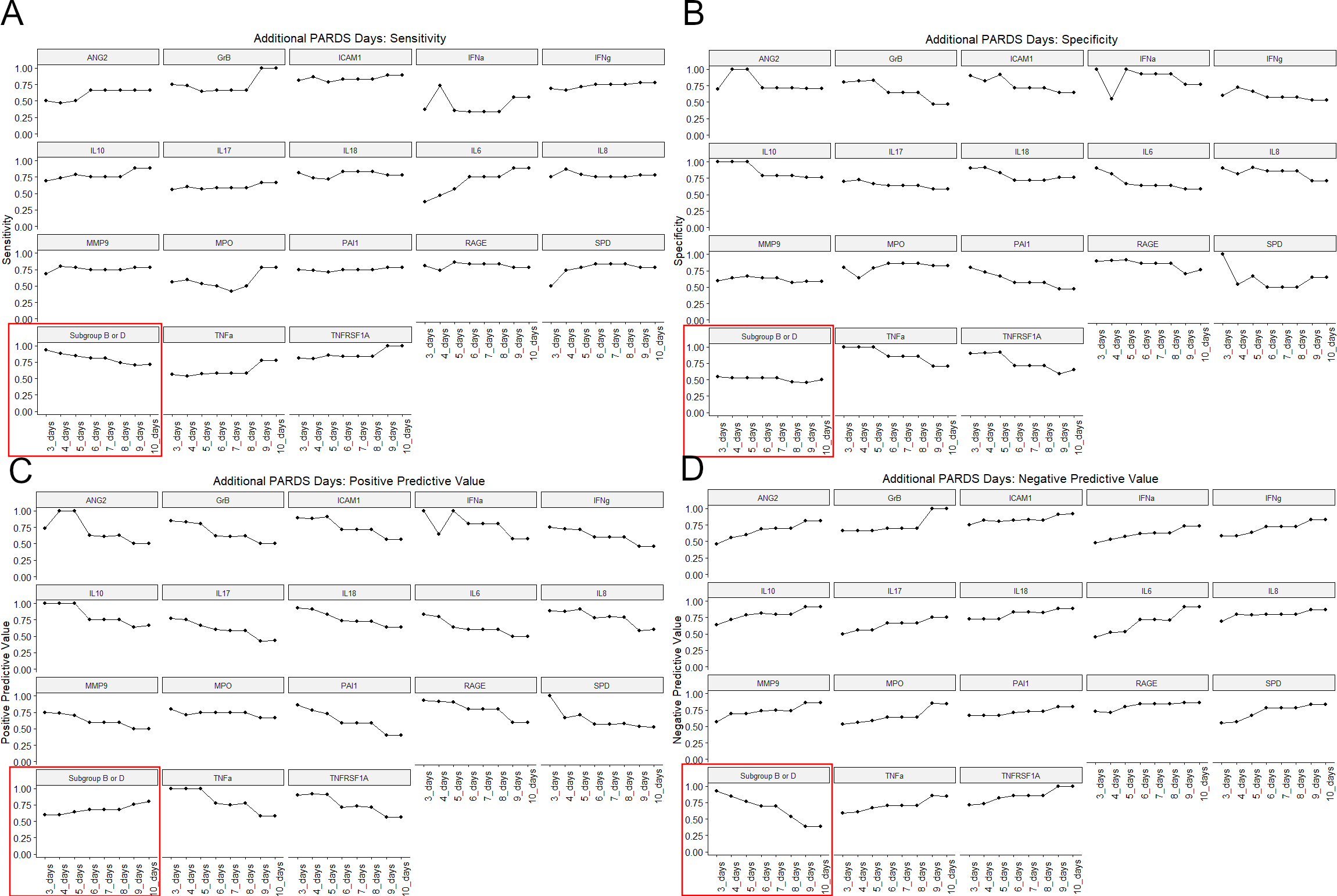

Supplement: Supplementary file 10 — Additional file 10: Fig. S9. Test characteristics of Initial Nasal Transcriptomic Subgroup vs. Seventeen Serum Biomarkers for Predicting Continued PARDS at Different Days. (A) The sensitivity of Nasal Transcriptomic Subgroup B or D for predicting continued PARDS at days 3–5 was high but diminished over time. This was in contrast to all of the other serum biomarkers which showed the opposite pattern. (B) The specificity of Nasal Transcriptomic Subgroup B or D for continued PARDS was low and inferior to all serum biomarkers assayed. (C) The positive predictive value of Nasal Transcriptomic Subgroup was poor for predicting short-term but good for predicting long-term continued PARDS. Again, this was the opposite of serum biomarkers. (D) The negative predictive value of initial Transcriptomic subgroup was good for early but poor for later PARDS. [file 12931_2022_2098_MOESM10_ESM.png]

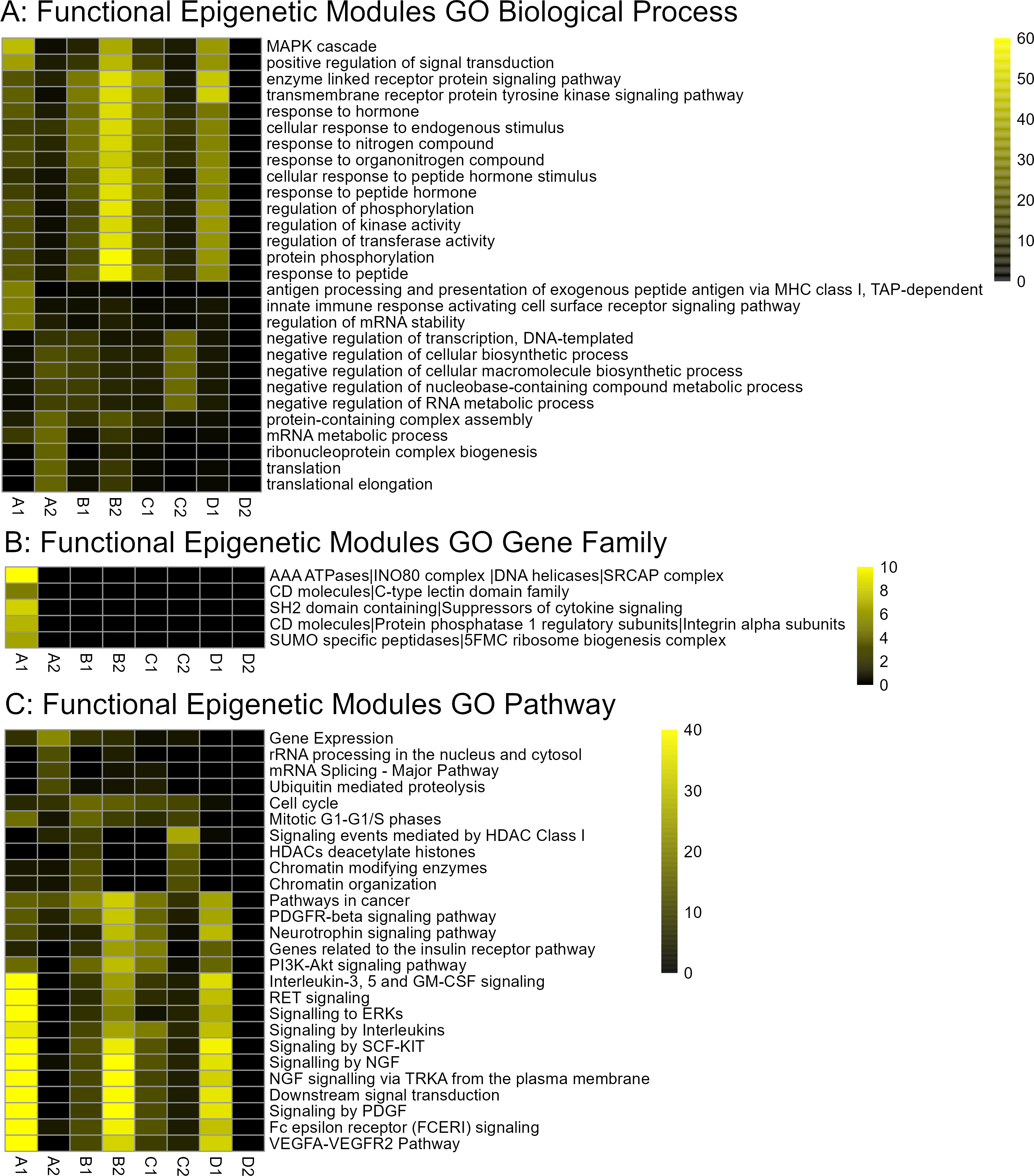

Supplement: Supplementary file 11 — Additional file 11: Fig. S10. Additional Functional Epigenetic Module Information. (A) Gene set enrichment analysis of genes with coordinate changes in methylation and expression identified specific biological processes, (B) gene families, (C) and pathways that may be regulated at the epigenetic level. [file 12931_2022_2098_MOESM11_ESM.png]
