# Supplementary material for: Multi-omic characterization of pediatric ARDS via nasal brushings
Source: Respir Res. 2022 Jul 9;23:181. doi: 10.1186/s12931-022-02098-3 (PMC9270778; doi:10.1186/s12931-022-02098-3)
Supplement: Supplementary file 12 — Additional file 12: Table S1. Specimen Log. [file 12931_2022_2098_MOESM12_ESM.pdf]

Supplemental Table 1: Specimen Log

Sample	Source	mRNA Seq	Methyl Seq	Serum	Underlying Dx	Acute Diagnosis
ARDS_001_ND1	Nasal	RNA-Only	A_1	Yes	Pinealoblastoma	Status epilepticus with aspiration
ARDS_001_TD1	Bronchial	None	D_41	Yes	Pinealoblastoma	Status epilepticus with aspiration
ARDS_001_ND3	Nasal	RNA-Only	None	Yes	Pinealoblastoma	Status epilepticus with aspiration
ARDS_001_TD3	Bronchial	None	C_25	Yes	Pinealoblastoma	Status epilepticus with aspiration
ARDS_001_ND7	Nasal	RNA-Only	B_17	Yes	Pinealoblastoma	Status epilepticus with aspiration
ARDS_001_TD7	Bronchial	NEB_1	C_33	No	Pinealoblastoma	Status epilepticus with aspiration
ARDS_002_ND1	Nasal	RNA-Only	None	Yes	Nephrotic syndrome post kidney transplant	Refractory shock
ARDS_002_TD1_NEB	Bronchial	NEB_1	G_73	No	Nephrotic syndrome post kidney transplant	Refractory shock
ARDS_002_ND3	Nasal	RNA-Only	None	Yes	Nephrotic syndrome post kidney transplant	Refractory shock
ARDS_002_TD3	Bronchial	None	G_81	No	Nephrotic syndrome post kidney transplant	Refractory shock
ARDS_002_ND7	Nasal	RNA-Only	None	Yes	Nephrotic syndrome post kidney transplant	Refractory shock
ARDS_002_TD7	Bronchial	None	H_89	Yes	Nephrotic syndrome post kidney transplant	Refractory shock
ARDS_003_ND1	Nasal	None	A_2	Yes	Asthma	Pneumonia/Sepsis
ARDS_003_ND3	Nasal	None	None	Yes	Asthma	Pneumonia/Sepsis
ARDS_003_ND7	Nasal	NEB_1	None	No	Asthma	Pneumonia/Sepsis
ARDS_004_ND1	Nasal	RNA-Only	C_26	Yes	BMT with BO post lung transplant	Pneumonia-Viral&Bacterial
ARDS_004_ND3	Nasal	None	None	Yes	BMT with BO post lung transplant	Pneumonia-Viral&Bacterial
ARDS_004_ND7	Nasal	None	None	Yes	BMT with BO post lung transplant	Pneumonia-Viral&Bacterial
ARDS_004_ND14	Nasal	None	None	Yes	BMT with BO post lung transplant	Pneumonia-Viral&Bacterial
ARDS_005_ND1	Nasal	None	E_59	Yes	BPD/Trach/Vent	Pneumonia-Viral
ARDS_005_ND3	Nasal	None	None	Yes	BPD/Trach/Vent	Pneumonia-Viral
ARDS_005_ND7	Nasal	None	None	Yes	BPD/Trach/Vent	Pneumonia-Viral
ARDS_005_ND14	Nasal	RNA-Only	None	Yes	BPD/Trach/Vent	Pneumonia-Viral
ARDS_006_ND1	Nasal	None	H_90	Yes	Congenital CMV	Influenza A
ARDS_006_ND3	Nasal	None	None	Yes	Congenital CMV	Influenza A
ARDS_007_ND1	Nasal	None	B_19	Yes	Aplastic anemia post BMT	Coronovirus (non-COVID) infection
ARDS_007_ND3	Nasal	None	None	Yes	Aplastic anemia post BMT	Coronovirus (non-COVID) infection
ARDS_007_ND7	Nasal	NEB_1	None	No	Aplastic anemia post BMT	Coronovirus (non-COVID) infection
ARDS_007_ND14	Nasal	NEB_1	None	Yes	Aplastic anemia post BMT	Coronovirus (non-COVID) infection
ARDS_008_ND1	Nasal	NEB_1	H_91	Yes	Aplastic anemia/MDS/BMT	DAD/IPS
ARDS_008_TD1	Bronchial	NEB_1	E_52	Yes	Aplastic anemia/MDS/BMT	DAD/IPS
ARDS_008_ND3	Nasal	NEB_1	None	Yes	Aplastic anemia/MDS/BMT	DAD/IPS
ARDS_008_ND7	Nasal	NEB_1	None	Yes	Aplastic anemia/MDS/BMT	DAD/IPS
ARDS_008_ND14	Nasal	None	None	Yes	Aplastic anemia/MDS/BMT	DAD/IPS
ARDS_009_ND1	Nasal	NEB_1	A_4	Yes	ALL post BMT	Sepsis- Adenovirus & Corynebacterium
ARDS_009_ND3	Nasal	NEB_1	None	Yes	ALL post BMT	Sepsis- Adenovirus & Corynebacterium
ARDS_009_ND7	Nasal	NEB_1	C_28	No	ALL post BMT	Sepsis- Adenovirus & Corynebacterium
ARDS_009_ND14	Nasal	NEB_1	None	Yes	ALL post BMT	Sepsis- Adenovirus & Corynebacterium
ARDS_010_ND1	Nasal	NEB_1	C_36	Yes	Alpha Thalasemia	Trauma/TBI
ARDS_010_ND3	Nasal	NEB_1	None	Yes	Alpha Thalasemia	Trauma/TBI
ARDS_010_ND7	Nasal	NEB_1	None	Yes	Alpha Thalasemia	Trauma/TBI
ARDS_010_ND14	Nasal	NEB_1	D_44	No	Alpha Thalasemia	Trauma/TBI
ARDS_011_ND1	Nasal	NEB_1	F_68	Yes	T21, Pulm Htn, HIE, Trach/Vent Dep. Short Gut. TPN Dep	Adenovirus, H. flu.
ARDS_011_ND3	Nasal	NEB_1	None	No	T21, Pulm Htn, HIE, Trach/Vent Dep. Short Gut. TPN Dep	Adenovirus, H. flu.
ARDS_011_ND7	Nasal	NEB_1	None	Yes	T21, Pulm Htn, HIE, Trach/Vent Dep. Short Gut. TPN Dep	Adenovirus, H. flu.
ARDS_011_ND14	Nasal	NEB_1	None	Yes	T21, Pulm Htn, HIE, Trach/Vent Dep. Short Gut. TPN Dep	Adenovirus, H. flu.
ARDS_012_ND1	Nasal	NEB_1	A_5	Yes	Chromosomal Abnormality and CLD	Pseudomanas Pneumonia
ARDS_012_ND3	Nasal	NEB_1	None	Yes	Chromosomal Abnormality and CLD	Pseudomanas Pneumonia
ARDS_012_ND7	Nasal	NEB_1	None	Yes	Chromosomal Abnormality and CLD	Pseudomanas Pneumonia
ARDS_013_ND1	Nasal	NEB_1	C_29	Yes	SMA II	Aspiration Pneumonia
ARDS_013_ND3	Nasal	NEB_1	None	Yes	SMA II	Aspiration Pneumonia
ARDS_013_ND7	Nasal	NEB_1	None	Yes	SMA II	Aspiration Pneumonia
ARDS_014_ND1	Nasal	NEB_1	None	Yes	Airway Hemangioma	Aspiration of Blood
ARDS_014_ND14	Nasal	NEB_1	None	No	Airway Hemangioma	Aspiration of Blood
ARDS_015_ND1	Nasal	NEB_1	F_61	Yes	Chromosomal Abnormality	Picornavirus
ARDS_015_ND3	Nasal	NEB_1	None	No	Chromosomal Abnormality	Picornavirus
ARDS_016_ND1	Nasal	NEB_1	None	Yes	DD from HSV meningitis	Sepsis
ARDS_016_ND14	Nasal	NEB_1	None	Yes	DD from HSV meningitis	Sepsis
ARDS_016_ND3	Nasal	NEB_1	None	Yes	DD from HSV meningitis	Sepsis
ARDS_016_ND7	Nasal	NEB_1	None	Yes	DD from HSV meningitis	Sepsis
ARDS_017_ND1	Nasal	NEB_1	B_14	No	Rett Like Syndrome	Respiratory Viral Infection
ARDS_017_ND14	Nasal	NEB_1	None	Yes	Rett Like Syndrome	Respiratory Viral Infection
ARDS_017_ND3	Nasal	NEB_1	None	Yes	Rett Like Syndrome	Respiratory Viral Infection
ARDS_017_ND7	Nasal	NEB_1	None	No	Rett Like Syndrome	Respiratory Viral Infection
ARDS_018_ND1	Nasal	NEB_1	D_46	Yes	Septo-Optic Dysplasia	Sepsis- RSV, Pseudomonas, Steno, MSSA
ARDS_018_ND14	Nasal	NEB_2	None	Yes	Septo-Optic Dysplasia	Sepsis- RSV, Pseudomonas, Steno, MSSA
ARDS_018_ND3	Nasal	NEB_1	None	Yes	Septo-Optic Dysplasia	Sepsis- RSV, Pseudomonas, Steno, MSSA
ARDS_018_ND7	Nasal	NEB_2	None	Yes	Septo-Optic Dysplasia	Sepsis- RSV, Pseudomonas, Steno, MSSA
ARDS_019_ND1	Nasal	NEB_1	G_78	Yes	CLD	RSV
ARDS_020_ND1	Nasal	NEB_2	H_86	Yes	None	Croup and Viral Pneumonia
ARDS_020_ND3	Nasal	NEB_2	None	Yes	None	Croup and Viral Pneumonia
ARDS_021_ND1	Nasal	NEB_2	A_7	Yes	Lissencephaly	Septic Shock
ARDS_021_ND14	Nasal	NEB_2	None	Yes	Lissencephaly	Septic Shock
ARDS_021_ND3	Nasal	NEB_2	None	Yes	Lissencephaly	Septic Shock
ARDS_021_ND7	Nasal	NEB_2	None	Yes	Lissencephaly	Septic Shock
ARDS_022_ND3	Nasal	NEB_2	None	Yes	Hypoxic Ischemic Encephalopathy	Viral pneumonia
ARDS_022_ND7	Nasal	NEB_2	None	Yes	Hypoxic Ischemic Encephalopathy	Viral pneumonia
ARDS_023_ND1	Nasal	NEB_2	None	Yes	Riley Day Syndrome and Bronchiectasis	Influenza B
ARDS_023_ND14	Nasal	NEB_2	None	No	Riley Day Syndrome and Bronchiectasis	Influenza B
ARDS_023_ND3	Nasal	NEB_2	None	Yes	Riley Day Syndrome and Bronchiectasis	Influenza B
ARDS_023_ND7	Nasal	NEB_2	None	Yes	Riley Day Syndrome and Bronchiectasis	Influenza B
ARDS_024_ND1	Nasal	NEB_2	H_87	No	Holoprosencephaly with Trach/Vent	Cardiac arrest
ARDS_024_ND14	Nasal	NEB_2	None	No	Holoprosencephaly with Trach/Vent	Cardiac arrest
ARDS_024_ND3	Nasal	NEB_2	None	No	Holoprosencephaly with Trach/Vent	Cardiac arrest
ARDS_024_ND7	Nasal	NEB_2	None	Yes	Holoprosencephaly with Trach/Vent	Cardiac arrest
ARDS_025_ND1	Nasal	NEB_2	B_24	Yes	Epilepsy and Hydrocephalus	Septic Shock
ARDS_025_ND3	Nasal	NEB_2	None	Yes	Epilepsy and Hydrocephalus	Septic Shock
ARDS_026_ND1	Nasal	NEB_3	None	No	BMT	Multifactorial
ARDS_026_ND3	Nasal	NEB_3	None	Yes	BMT	Multifactorial
ARDS_026_ND7	Nasal	NEB_3	None	Yes	BMT	Multifactorial
ARDS_027_ND1	Nasal	NEB_3	None	Yes	AML s/p BMT	Multifactorial
ARDS_027_ND3	Nasal	None	None	Yes	AML s/p BMT	Multifactorial
ARDS_028_ND1	Nasal	NEB_3	None	No	Genetic Syndrome	Viral pneumonia
ARDS_028_ND14	Nasal	NEB_3	None	No	Genetic Syndrome	Viral pneumonia
ARDS_028_ND3	Nasal	NEB_3	None	Yes	Genetic Syndrome	Viral pneumonia
ARDS_028_ND7	Nasal	NEB_3	None	Yes	Genetic Syndrome	Viral pneumonia
ARDS_029_ND1	Nasal	NEB_3	None	Yes	None	Septic Shock - Meningococcus
ARDS_029_ND14	Nasal	NEB_3	None	Yes	None	Septic Shock - Meningococcus
ARDS_029_ND3	Nasal	NEB_3	None	Yes	None	Septic Shock - Meningococcus
ARDS_029_ND7	Nasal	NEB_3	None	Yes	None	Septic Shock - Meningococcus
ARDS_030_ND1	Nasal	NEB_3	None	Yes	History of prematurity and baseline congenital or acquired heart disease	Pnuemonia
ARDS_030_ND14	Nasal	NEB_3	None	Yes	History of prematurity and baseline congenital or acquired heart disease	Pnuemonia
ARDS_030_ND3_1	Nasal	NEB_3	None	Yes	History of prematurity and baseline congenital or acquired heart disease	Pnuemonia
ARDS_030_ND7	Nasal	NEB_3	None	Yes	History of prematurity and baseline congenital or acquired heart disease	Pnuemonia
ARDS_031_ND1	Nasal	NEB_3	None	Yes	Baseline congenital or acquired heart disease	Bacteial Pneumonia - Pseudomonas
ARDS_031_ND3	Nasal	NEB_3	None	Yes	Baseline congenital or acquired heart disease	Bacteial Pneumonia - Pseudomonas
ARDS_031_ND7	Nasal	NEB_3	None	No	Baseline congenital or acquired heart disease	Bacteial Pneumonia - Pseudomonas
ARDS_032_ND1	Nasal	NEB_3	None	No	None	Trauma
ARDS_032_ND3	Nasal	NEB_3	None	No	None	Trauma
ARDS_032_ND7	Nasal	NEB_3	None	No	None	Trauma

ARDS_033_ND1	Nasal	NEB_3	None	No	History of prematurity and baseline neurodevelomental delay	Non-pulmonary sepsis
ARDS_033_ND3	Nasal	NEB_3	None	Yes	History of prematurity and baseline neurodevelomental delay	Non-pulmonary sepsis
ARDS_033_ND7	Nasal	NEB_3	None	Yes	History of prematurity and baseline neurodevelomental delay	Non-pulmonary sepsis
CHOP_ARDS_001_ND1	Nasal	NEB_4	None	Yes	BMT	Viral pneumonia-Adenovirus
CHOP_ARDS_001_ND3	Nasal	NEB_4	None	No	BMT	Viral pneumonia-Adenovirus
CHOP_ARDS_001_ND	Nasal	NEB_4	None	No	BMT	Viral pneumonia-Adenovirus
CHOP_ARDS_001_ND14	Nasal	NEB_4	None	No	BMT	Viral pneumonia-Adenovirus
CHOP_ARDS_002_ND1	Nasal	NEB_4	None	Yes	Genetic Syndrome	Bacteial Pneumonia - Pseudomonas
CHOP_ARDS_002_ND3	Nasal	NEB_4	None	No	Genetic Syndrome	Bacteial Pneumonia - Pseudomonas
CHOP_ARDS_002_ND	Nasal	NEB_4	None	No	Genetic Syndrome	Bacteial Pneumonia - Pseudomonas
CHOP_ARDS_002_ND14	Nasal	NEB_4	None	No	Genetic Syndrome	Bacteial Pneumonia - Pseudomonas
CHOP_ARDS_003_ND1	Nasal	NEB_4	None	Yes	BMT	Sepsis-Pseudomonas aeruginosa; Serratia marcescens
CHOP_ARDS_004_ND1	Nasal	NEB_4	None	No	Genetic Syndrome	Bacterial Pneumonia - Methicillin Resistant Staphylococcus aureus
CHOP_ARDS_005_ND1	Nasal	NEB_4	None	Yes	Oncologic	Fungal Pneumonia - Saprochaete clavata
CHOP_ARDS_005_ND3	Nasal	NEB_4	None	No	Oncologic	Fungal Pneumonia - Saprochaete clavata
CHOP_ARDS_006_ND1	Nasal	NEB_4	None	Yes	Neurodevelopmental Dela	Viral Pneumonia - COVID19
CHOP_ARDS_006_ND3	Nasal	NEB_4	None	No	Neurodevelopmental Dela	Viral Pneumonia - COVID19
CHOP_ARDS_006_ND7	Nasal	NEB_4	None	No	Neurodevelopmental Dela	Viral Pneumonia - COVID19
Control_001_ND1	Nasal	RNA-Only	None	Yes	None	MVC/TBI
Control_001_TD1	Bronchial	None	D_48	Yes	None	MVC/TBI
Control_002_ND1	Nasal	RNA-Only	E_57	Yes	Laryngeal Cleft	Pneumonia-Viral&Bacterial
Control_002_TD1	Bronchial	None	F_64	Yes	Laryngeal Cleft	Pneumonia-Viral&Bacterial
Control_002_ND3	Nasal	RNA-Only	F_72	No	Laryngeal Cleft	Pneumonia-Viral&Bacterial
Control_003_ND1	Nasal	NEB_1	H_88	Yes	HUS/AKI	PRES
Control_004_ND1	Nasal	None	F_63	No	Central Hypoventilation Syndrome with trach/vent dependence	Tracheal stoma revision
Control_005_ND1	Nasal	None	E_56	No	Chiari Malformation Type 1	Posterior fossa decompression
Control_006_ND1	Nasal	None	E_54	No	Desmoplastic infantile ganglioma and Hydrocephalus	Shunt malfunction
Control_007_ND1	Nasal	None	None	Yes	NAT with intractable epilepsy	s/p Craniotomy with functional hemispherotomy
Control_009_ND1	Nasal	None	J_B2	No	Tracheo-bronchomalacia	post tracheostomy
Control_009_ND14	Nasal	RNA-Only	None	No	Tracheo-bronchomalacia	post tracheostomy
Control_010_ND3	Nasal	RNA-Only	None	No	Refractory Epilepsy	Brain tumur resection
Control_011_ND1	Nasal	None	J_B12	No	None	Infantile botulism
Control_012_ND1	Nasal	NEB_1	J_B6	No	Cephelocele	Cephalocele Closure
Control_013_ND1	Nasal	NEB_1	K_B7	Yes	MMC, hydrocephalus, neurogenic bladder	GU Reconstruction
Control_013_ND3	Nasal	NEB_1	None	No	MMC, hydrocephalus, neurogenic bladder	GU Reconstruction
Control_15_ND1	Nasal	None	None	Yes	TEF and esophageal atresia	Esophageal dilation
Control_015_ND7	Nasal	NEB_1	None	No	TEF and esophageal atresia	Esophageal dilation
Control_017_ND1	Nasal	NEB_1	None	No	Perianal Fistula	PSARP
Control_017_ND3	Nasal	NEB_1	None	No	Perianal Fistula	PSARP
Control_017_ND7	Nasal	NEB_1	None	No	Perianal Fistula	PSARP
Control_018_ND1	Nasal	NEB_1	None	No	Subglottic Stenosis	Laryngotracheoplasty
Control_018_ND3	Nasal	NEB_1	None	No	Subglottic Stenosis	Laryngotracheoplasty
Control_018_ND7	Nasal	NEB_1	None	No	Subglottic Stenosis	Laryngotracheoplasty
Control_019_ND1	Nasal	NEB_1	K_C12	Yes	Subglottic Stenosis	Laryngotracheoplasty
Control_019_ND3	Nasal	NEB_1	None	Yes	Subglottic Stenosis	Laryngotracheoplasty
Control_019_ND7	Nasal	NEB_1	None	Yes	Subglottic Stenosis	Laryngotracheoplasty
Control_020_ND1	Nasal	NEB_1	L_C7	Yes	Chronic Pancreatitis	TPIAT
Control_020_ND14	Nasal	NEB_1	None	No	Chronic Pancreatitis	TPIAT
Control_020_ND3	Nasal	NEB_1	None	Yes	Chronic Pancreatitis	TPIAT
Control_020_ND7	Nasal	NEB_1	None	Yes	Chronic Pancreatitis	TPIAT
Control_021_ND1	Nasal	NEB_1	L_D3	Yes	ESRD	Kidney Transplant
Control_021_ND3	Nasal	NEB_1	None	No	ESRD	Kidney Transplant
Control_021_ND7	Nasal	NEB_1	None	No	ESRD	Kidney Transplant
Control_022_ND1	Nasal	NEB_2	L_D6	Yes	Chronic Pancreatitis	TPIAT
Control_022_ND3	Nasal	NEB_2	None	Yes	Chronic Pancreatitis	TPIAT
Control_022_ND7	Nasal	NEB_2	None	Yes	Chronic Pancreatitis	TPIAT
Control_023_ND1	Nasal	None	None	Yes	Brain Tumor	Obstructive Hydrocephalus
Control_023_ND14	Nasal	NEB_2	None	No	Brain Tumor	Obstructive Hydrocephalus

DAD=Diffuse Alveolar Damage, GU=Genitourinary, IPS=Idiopathic Pulmonary Syndrome, MSSA=Methcillin Susceptible Staph aureus, MVC=Motor Vehicle Collision, PRES=Posterior Reversible Encephalopathy Syndrome, PSARP=Posterior Sagittal Anorectoplasty,
