# Supplementary material for: Multi-omic characterization of pediatric ARDS via nasal brushings
Source: Respir Res. 2022 Jul 9;23:181. doi: 10.1186/s12931-022-02098-3 (PMC9270778; doi:10.1186/s12931-022-02098-3)
Supplement: Supplementary file 13 — Additional file 13: Table S2. Differentially Methylated Transcription Start Sites Bronchial vs. Nasal. [file 12931_2022_2098_MOESM13_ESM.pdf]

Supplemental Table 2: Differentially Methylated Transcription Start Sites Subgroup 1 vs. Subgroup 2

Methyl Subgroup 2 Hypomethylated	Methyl Subgroup 2 Hypermethylated
THAP12 (THAP domain containing 12)	PKN1 (protein kinase N1)
PRPS2 (phosphoribosyl pyrophosphate synthetase 2)	MAPK13 (mitogen-activated protein kinase 13)
RNASEL (ribonuclease L)	MAP2K1 (mitogen-activated protein kinase kinase 1)
RPL9 (ribosomal protein L9)	PSMA1 (proteasome 20S subunit alpha 1)
SECTM1 (secreted and transmembrane 1)	CEACAM5 (CEA cell adhesion molecule 5)
SLC18A3 (solute carrier family 18 member A3)	RGS1 (regulator of G protein signaling 1)
SSRP1 (structure specific recognition protein 1)	RNASE3 (ribonuclease A family member 3)
SSX2 (SSX family member 2)	RPL7A (ribosomal protein L7a)
DDOST (dolichyl-diphosphooligosaccharide--protein glycosyltransferase non-catalytic	AP2A2 (adaptor related protein complex 2 subunit alpha 2)
DSCAM (DS cell adhesion molecule)	LYST (lysosomal trafficking regulator)
H2BC17 (H2B clustered histone 17)	MSMO1 (methylsterol monooxygenase 1)
CCT7 (chaperonin containing TCP1 subunit 7)	CCL8 (C-C motif chemokine ligand 8)
HSPH1 (heat shock protein family H (Hsp110) member 1)	SETMAR (SET domain and mariner transposase fusion gene)
STARD10 (StAR related lipid transfer domain containing 10)	SGCG (sarcoglycan gamma)
HHLA2 (HERV-H LTR-associating 2)	SLC18A1 (solute carrier family 18 member A1)
LTA4H (leukotriene A4 hydrolase)	SLC18A2 (solute carrier family 18 member A2)
NFKBIE (NFKB inhibitor epsilon)	HLTF (helicase like transcription factor)
PGM1 (phosphoglucomutase 1)	CNGA2 (cyclic nucleotide gated channel subunit alpha 2)
CXCR5 (C-X-C motif chemokine receptor 5)	SMARCD2 (SWI/SNF related, matrix associated, actin dependent regulator of
IL6 (interleukin 6)	SIGLEC1 (sialic acid binding Ig like lectin 1)
KNG1 (kininogen 1)	SOX3 (SRY-box transcription factor 3)
LECT2 (leukocyte cell derived chemotaxin 2)	SSRP1 (structure specific recognition protein 1)
MGAT5 (alpha-1,6-mannosylglycoprotein 6-beta-N-acetylglucosaminyltransferase)	SST (somatostatin)
MT1JP (metallothionein 1J, pseudogene)	SSTR1 (somatostatin receptor 1)
MT1M (metallothionein 1M)	SSTR2 (somatostatin receptor 2)
MT1L (metallothionein 1L, pseudogene)	SSTR3 (somatostatin receptor 3)
NDUFS2 (NADH:ubiquinone oxidoreductase core subunit S2)	SSTR5 (somatostatin receptor 5)
PFKFB1 (6-phosphofructo-2-kinase/fructose-2,6-biphosphatase 1)	SSX1 (SSX family member 1)
PPP1R8 (protein phosphatase 1 regulatory subunit 8)	SSX2 (SSX family member 2)
NPY4R (neuropeptide Y receptor Y4)	SSX5 (SSX family member 5)
MAPK10 (mitogen-activated protein kinase 10)	SSX4 (SSX family member 4)
FHL1 (four and a half LIM domains 1)	SS18 (SS18 subunit of BAF chromatin remodeling complex)
PSMB9 (proteasome 20S subunit beta 9)	ST13 (ST13 Hsp70 interacting protein)
GCNT1 (glucosaminyl (N-acetyl) transferase 1)	STAT4 (signal transducer and activator of transcription 4)
SPRR1A (small proline rich protein 1A)	STAT5A (signal transducer and activator of transcription 5A)
SRD5A2 (steroid 5 alpha-reductase 2)	SYK (spleen associated tyrosine kinase)
TLE3 (TLE family member 3, transcriptional corepressor)	THBS2 (thrombospondin 2)
HSF1 (heat shock transcription factor 1)	TJP1 (tight junction protein 1)
UBA1 (ubiquitin like modifier activating enzyme 1)	TMOD1 (tropomodulin 1)
TNC (tenascin C)	DAB1 (DAB adaptor protein 1)
UGDH (UDP-glucose 6-dehydrogenase)	TXK (TXK tyrosine kinase)
PRICKLE3 (prickle planar cell polarity protein 3)	VHL (von Hippel-Lindau tumor suppressor)
CD1B (CD1b molecule)	DPEP1 (dipeptidase 1)
CNGA2 (cyclic nucleotide gated channel subunit alpha 2)	ELAVL3 (ELAV like RNA binding protein 3)
NEFH (neurofilament heavy chain)	ENO2 (enolase 2)
ORC5 (origin recognition complex subunit 5)	AKAP17A (A-kinase anchoring protein 17A)
SLC22A18 (solute carrier family 22 member 18)	H3C8 (H3 clustered histone 8)
OXTR (oxytocin receptor)	OR1D5 (olfactory receptor family 1 subfamily D member 5)
PAX5 (paired box 5)	BCAR3 (BCAR3 adaptor protein, NSP family member)
PROP1 (PROP paired-like homeobox 1)	GALR3 (galanin receptor 3)
SCT (secretin)	FCGR1A (Fc gamma receptor Ia)
SLC10A1 (solute carrier family 10 member 1)	CASK (calcium/calmodulin dependent serine protein kinase)
MAP3K7 (mitogen-activated protein kinase kinase kinase 7)	FBP2 (fructose-bisphosphatase 2)
SLC25A5 (solute carrier family 25 member 5)	APBB2 (amyloid beta precursor protein binding family B member 2)
UQCRH (ubiquinol-cytochrome c reductase hinge protein)	AP1M1 (adaptor related protein complex 1 subunit mu 1)
VSNL1 (visinin like 1)	SGCE (sarcoglycan epsilon)
CPZ (carboxypeptidase Z)	FMO2 (flavin containing dimethylaniline monoxygenase 2)
BLZF1 (basic leucine zipper nuclear factor 1)	PHOX2B (paired like homeobox 2B)
EIF3B (eukaryotic translation initiation factor 3 subunit B)	RTL8C (retrotransposon Gag like 8C)
IDH1 (isocitrate dehydrogenase (NADP(+)) 1)	SYT7 (synaptotagmin 7)
DDR1 (discoidin domain receptor tyrosine kinase 1)	INA (internexin neuronal intermediate filament protein alpha)
LY6E (lymphocyte antigen 6 family member E)	FRZB (frizzled related protein)
MLF1 (myeloid leukemia factor 1)	GPR55 (G protein-coupled receptor 55)
CPT1B (carnitine palmitoyltransferase 1B)	B4GALT1 (beta-1,4-galactosyltransferase 1)
MAPK14 (mitogen-activated protein kinase 14)	STOML1 (stomatin like 1)
RPS27A (ribosomal protein S27a)	ARHGAP29 (Rho GTPase activating protein 29)
SCN1A (sodium voltage-gated channel alpha subunit 1)	GPLD1 (glycosylphosphatidylinositol specific phospholipase D1)
SNAPC2 (small nuclear RNA activating complex polypeptide 2)	GPM6B (glycoprotein M6B)
SNRPD3 (small nuclear ribonucleoprotein D3 polypeptide)	ATF4 (activating transcription factor 4)
SRP72 (signal recognition particle 72)	HDC (histidine decarboxylase)

SSR3 (signal sequence receptor subunit 3)
STIM1 (stromal interaction molecule 1)
THBS3 (thrombospondin 3)
UPP1 (uridine phosphorylase 1)
XPNPEP1 (X-prolyl aminopeptidase 1)
PSCA (prostate stem cell antigen)
GPR68 (G protein-coupled receptor 68)
BNIP2 (BCL2 interacting protein 2)
SHROOM2 (shroom family member 2)
ECHS1 (enoyl-CoA hydratase, short chain 1)
ETFDH (electron transfer flavoprotein dehydrogenase)
C3 (complement C3)
FGF10 (fibroblast growth factor 10)
FOXO3 (forkhead box O3)
FLNB (filamin B)
GRIN2C (glutamate ionotropic receptor NMDA type subunit 2C)
HCFC1 (host cell factor C1)
HLA-DMA (major histocompatibility complex, class II, DM alpha)
EFNA4 (ephrin A4)
EFNA5 (ephrin A5)
EFNB1 (ephrin B1)
MEGF6 (multiple EGF like domains 6)
EGFR (epidermal growth factor receptor)
EIF2B1 (eukaryotic translation initiation factor 2B subunit alpha)
F12 (coagulation factor XII)
GRIK3 (glutamate ionotropic receptor kainate type subunit 3)
APOA4 (apolipoprotein A4)
LAMA4 (laminin subunit alpha 4)
LAMA5 (laminin subunit alpha 5)
CST6 (cystatin E/M)
ME2 (malic enzyme 2)
MGMT (O-6-methylguanine-DNA methyltransferase)
KITLG (KIT ligand)
P2RX1 (purinergic receptor P2X 1)
P2RX3 (purinergic receptor P2X 3)
P2RX7 (purinergic receptor P2X 7)
P2RY1 (purinergic receptor P2Y1)
P2RY4 (pyrimidinergic receptor P2Y4)
P4HB (prolyl 4-hydroxylase subunit beta)
STOM (stomatin)
EPHA4 (EPH receptor A4)
EPHA5 (EPH receptor A5)
EPHB3 (EPH receptor B3)
ASPH (aspartate beta-hydroxylase)
EPS8 (epidermal growth factor receptor pathway substrate 8)
ESD (esterase D)
HMGCS1 (3-hydroxy-3-methylglutaryl-CoA synthase 1)
MTHFD1 (methylenetetrahydrofolate dehydrogenase, cyclohydrolase and
PAK3 (p21 (RAC1) activated kinase 3)
PEX14 (peroxisomal biogenesis factor 14)
F7 (coagulation factor VII)
FKBP2 (FKBP prolyl isomerase 2)
BPI (bactericidal permeability increasing protein)
GTF2H1 (general transcription factor IIH subunit 1)
GTF3C2 (general transcription factor IIIC subunit 2)
HOXB5 (homeobox B5)
HOXB6 (homeobox B6)
HOXC5 (homeobox C5)
HRG (histidine rich glycoprotein)
HSBP1 (heat shock factor binding protein 1)
IBSP (integrin binding sialoprotein)
IL13 (interleukin 13)
LSP1 (lymphocyte specific protein 1)
MAGEA3 (MAGE family member A3)
MAGEA4 (MAGE family member A4)
MANBA (mannosidase beta)
MMP14 (matrix metallopeptidase 14)
NDUFA4 (NDUFA4 mitochondrial complex associated)
NDUFV2 (NADH:ubiquinone oxidoreductase core subunit V2)
NEDD9 (neural precursor cell expressed, developmentally down-regulated 9)
COL5A1 (collagen type V alpha 1 chain)
PCDH1 (protocadherin 1)
PSG3 (pregnancy specific beta-1-glycoprotein 3)
HNF4A (hepatocyte nuclear factor 4 alpha)
IFITM2 (interferon induced transmembrane protein 2)
IGF2BP1 (insulin like growth factor 2 mRNA binding protein 1)
HSPH1 (heat shock protein family H (Hsp110) member 1)
STARD10 (StAR related lipid transfer domain containing 10)
FRS3 (fibroblast growth factor receptor substrate 3)
PPP1R13L (protein phosphatase 1 regulatory subunit 13 like)
SLC27A3 (solute carrier family 27 member 3)
SPINK5 (serine peptidase inhibitor Kazal type 5)
KDELR3 (KDEL endoplasmic reticulum protein retention receptor 3)
MOS (MOS proto-oncogene, serine/threonine kinase)
MTAP (methylthioadenosine phosphorylase)
MTIF2 (mitochondrial translational initiation factor 2)
MT-ND3 (NADH dehydrogenase subunit 3)
NDUFC1 (NADH:ubiquinone oxidoreductase subunit C1)
NUCB2 (nucleobindin 2)
OCRL (OCRL inositol polyphosphate-5-phosphatase)
CBLB (Cbl proto-oncogene B)
PFKFB1 (6-phosphofructo-2-kinase/fructose-2,6-biphosphatase 1)
PGM3 (phosphoglucomutase 3)
PHF2 (PHD finger protein 2)
CD40LG (CD40 ligand)
IFNA16 (interferon alpha 16)
IFNA17 (interferon alpha 17)
IFNB1 (interferon beta 1)
IFNG (interferon gamma)
IGFBP1 (insulin like growth factor binding protein 1)
IGFBP2 (insulin like growth factor binding protein 2)
IGFBP3 (insulin like growth factor binding protein 3)
IK (IK cytokine)
IL1RN (interleukin 1 receptor antagonist)
KCNA4 (potassium voltage-gated channel subfamily A member 4)
KIF3C (kinesin family member 3C)
KIF11 (kinesin family member 11)
CALCR (calcitonin receptor)
JMJD7-PLA2G4B (JMJD7-PLA2G4B readthrough)
CD8A (CD8a molecule)
ADCY7 (adenylate cyclase 7)
LMO2 (LIM domain only 2)
COX15 (cytochrome c oxidase assembly homolog COX15)
MITF (melanocyte inducing transcription factor)
MOCS1 (molybdenum cofactor synthesis 1)
MT1JP (metallothionein 1J, pseudogene)
MT1M (metallothionein 1M)
MT1L (metallothionein 1L, pseudogene)
NCAN (neurocan)
MUTYH (mutY DNA glycosylase)
CCN2 (cellular communication network factor 2)
MYCN (MYCN proto-oncogene, bHLH transcription factor)
MYF5 (myogenic factor 5)
NAP1L1 (nucleosome assembly protein 1 like 1)
NDUFA7 (NADH:ubiquinone oxidoreductase subunit A7)
CYP2B6 (cytochrome P450 family 2 subfamily B member 6)
NEK3 (NIMA related kinase 3)
CYP2D6 (cytochrome P450 family 2 subfamily D member 6)
NFIL3 (nuclear factor, interleukin 3 regulated)
NFRKB (nuclear factor related to kappaB binding protein)
NINJ1 (ninjurin 1)
NOS1 (nitric oxide synthase 1)
NTRK2 (neurotrophic receptor tyrosine kinase 2)
ODF1 (outer dense fiber of sperm tails 1)
DPP4 (dipeptidyl peptidase 4)
AMH (anti-Mullerian hormone)
ANPEP (alanyl aminopeptidase, membrane)
PIK3R1 (phosphoinositide-3-kinase regulatory subunit 1)
FHIT (fragile histidine triad diadenosine triphosphatase)
PSPN (persephin)
FKBP4 (FKBP prolyl isomerase 4)
PSG11 (pregnancy specific beta-1-glycoprotein 11)
PSMC2 (proteasome 26S subunit, ATPase 2)
PSMC6 (proteasome 26S subunit, ATPase 6)
PTGFR (prostaglandin F receptor)
PTS (6-pyruvoyltetrahydropterin synthase)

RBBP6 (RB binding protein 6, ubiquitin ligase)
AREG (amphiregulin)
GLP1R (glucagon like peptide 1 receptor)
IGFBP7 (insulin like growth factor binding protein 7)
CA11 (carbonic anhydrase 11)
LTB (lymphotoxin beta)
CYP4F3 (cytochrome P450 family 4 subfamily F member 3)
LY6H (lymphocyte antigen 6 family member H)
MSR1 (macrophage scavenger receptor 1)
MYH11 (myosin heavy chain 11)
PDE1A (phosphodiesterase 1A)
SERPINB4 (serpin family B member 4)
SLC6A8 (solute carrier family 6 member 8)
E2F3 (E2F transcription factor 3)
TCN1 (transcobalamin 1)
TGFB1 (transforming growth factor beta 1)
ZNF12 (zinc finger protein 12)
EMX1 (empty spiracles homeobox 1)
CASQ2 (calsequestrin 2)
FGF13 (fibroblast growth factor 13)
FUCA1 (alpha-L-fucosidase 1)
GABRD (gamma-aminobutyric acid type A receptor subunit delta)
GLRA1 (glycine receptor alpha 1)
ANXA1 (annexin A1)
GPR21 (G protein-coupled receptor 21)
NR3C1 (nuclear receptor subfamily 3 group C member 1)
APOF (apolipoprotein F)
GATA3 (GATA binding protein 3)
FFAR2 (free fatty acid receptor 2)
H1-4 (H1.4 linker histone, cluster member)
IL2RB (interleukin 2 receptor subunit beta)
FADS1 (fatty acid desaturase 1)
MEF2A (myocyte enhancer factor 2A)
MFAP4 (microfibril associated protein 4)
MOG (myelin oligodendrocyte glycoprotein)
MOV10 (Mov10 RISC complex RNA helicase)

CEACAM4 (CEA cell adhesion molecule 4)

HOOK1 (hook microtubule tethering protein 1)
PEG3 (paternally expressed 3)
PITX3 (paired like homeodomain 3)
RPL22 (ribosomal protein L22)
RPLP0 (ribosomal protein lateral stalk subunit P0)
COL13A1 (collagen type XIII alpha 1 chain)
SDCBP (syndecan binding protein)
HSPA13 (heat shock protein family A (Hsp70) member 13)
TAF11 (TATA-box binding protein associated factor 11)
PPP2R1B (protein phosphatase 2 scaffold subunit Abeta)
ALDH2 (aldehyde dehydrogenase 2 family member)
PTPRA (protein tyrosine phosphatase receptor type A)
PCYT2 (phosphate cytidylyltransferase 2, ethanolamine)
RPS4X (ribosomal protein S4 X-linked)
RPS27 (ribosomal protein S27)
SCN8A (sodium voltage-gated channel alpha subunit 8)
E2F4 (E2F transcription factor 4)
SMO (smoothened, frizzled class receptor)
ASIC2 (acid sensing ion channel subunit 2)
ELOB (elongin B)
TGM3 (transglutaminase 3)
TKT (transketolase)
TP53 (tumor protein p53)
ST8SIA4 (ST8 alpha-N-acetyl-neuraminide alpha-2,8-sialyltransferase 4)
CHAF1B (chromatin assembly factor 1 subunit B)
H2AC4 (H2A clustered histone 4)
CBX4 (chromobox 4)
AGPS (alkylglycerone phosphate synthase)
PIAS1 (protein inhibitor of activated STAT 1)
HNMT (histamine N-methyltransferase)
SLC29A2 (solute carrier family 29 member 2)
EIF3D (eukaryotic translation initiation factor 3 subunit D)
EIF3F (eukaryotic translation initiation factor 3 subunit F)
ADAM15 (ADAM metallopeptidase domain 15)

ARSA (arylsulfatase A)
ASIC2 (acid sensing ion channel subunit 2)
FUT1 (fucosyltransferase 1 (H blood group))
ATP1A2 (ATPase Na+/K+ transporting subunit alpha 2)
ATP1A3 (ATPase Na+/K+ transporting subunit alpha 3)
CCL20 (C-C motif chemokine ligand 20)
SELE (selectin E)
SLC16A1 (solute carrier family 16 member 1)
SP100 (SP100 nuclear antigen)
SPRR1B (small proline rich protein 1B)
STC1 (stanniocalcin 1)
TBXT (T-box transcription factor T)
HBA1 (hemoglobin subunit alpha 1)
TBXAS1 (thromboxane A synthase 1)
HDAC1 (histone deacetylase 1)
CFHR2 (complement factor H related 2)
U2AF1 (U2 small nuclear RNA auxiliary factor 1)
UBA1 (ubiquitin like modifier activating enzyme 1)
UBE2D1 (ubiquitin conjugating enzyme E2 D1)
UBE2E2 (ubiquitin conjugating enzyme E2 E2)
UCP2 (uncoupling protein 2)
UVRAG (UV radiation resistance associated)
VIP (vasoactive intestinal peptide)
WRN (WRN RecQ like helicase)
H2AZ1 (H2A.Z variant histone 1)
ICAM3 (intercellular adhesion molecule 3)
ID3 (inhibitor of DNA binding 3, HLH protein)
BCAT2 (branched chain amino acid transaminase 2)
IL1RAP (interleukin 1 receptor accessory protein)
KCNA2 (potassium voltage-gated channel subfamily A member 2)
KCNJ13 (potassium inwardly rectifying channel subfamily J member 13)
KIF5B (kinesin family member 5B)
KRT19 (keratin 19)
KRT84 (keratin 84)
C6 (complement C6)
LAMC2 (laminin subunit gamma 2)

LHCGR (luteinizing hormone/choriogonadotropin receptor)

ADCY5 (adenylate cyclase 5)
MAP6 (microtubule associated protein 6)
MARK1 (microtubule affinity regulating kinase 1)
MMP9 (matrix metallopeptidase 9)
MMP10 (matrix metallopeptidase 10)
CNR1 (cannabinoid receptor 1)
MYBL1 (MYB proto-oncogene like 1)
AKT1 (AKT serine/threonine kinase 1)
MYF6 (myogenic factor 6)
CLDN7 (claudin 7)
CPN2 (carboxypeptidase N subunit 2)
ORC5 (origin recognition complex subunit 5)
SLC22A18 (solute carrier family 22 member 18)
PAK1 (p21 (RAC1) activated kinase 1)
PEX12 (peroxisomal biogenesis factor 12)
PPP1R1A (protein phosphatase 1 regulatory inhibitor subunit 1A)
PRPSAP2 (phosphoribosyl pyrophosphate synthetase associated protein 2)
PSMD2 (proteasome 26S subunit ubiquitin receptor, non-ATPase 2)
RPL35A (ribosomal protein L35a)
SGK1 (serum/glucocorticoid regulated kinase 1)
FBXW4 (F-box and WD repeat domain containing 4)
SLC5A2 (solute carrier family 5 member 2)
SLC6A4 (solute carrier family 6 member 4)
SLC22A4 (solute carrier family 22 member 4)
SNAPC2 (small nuclear RNA activating complex polypeptide 2)
ENO3 (enolase 3)
SNX1 (sorting nexin 1)
SPI1 (Spi-1 proto-oncogene)
SPTAN1 (spectrin alpha, non-erythrocytic 1)
AMPD2 (adenosine monophosphate deaminase 2)
SRP19 (signal recognition particle 19)
TRIM21 (tripartite motif containing 21)
SYT5 (synaptotagmin 5)
TACC1 (transforming acidic coiled-coil containing protein 1)

CFLAR (CASP8 and FADD like apoptosis regulator)
ATP6V0E1 (ATPase H+ transporting V0 subunit e1)
PGLYRP1 (peptidoglycan recognition protein 1)
C4BPB (complement component 4 binding protein beta)
LDLR (low density lipoprotein receptor)
MPP1 (MAGUK p55 scaffold protein 1)
SCML2 (Scm polycomb group protein like 2)
BAIAP2 (BAR/IMD domain containing adaptor protein 2)
CDH5 (cadherin 5)
PRSS21 (serine protease 21)
TRIOBP (TRIO and F-actin binding protein)
RER1 (retention in endoplasmic reticulum sorting receptor 1)
PDK2 (pyruvate dehydrogenase kinase 2)
DDX42 (DEAD-box helicase 42)
CKS2 (CDC28 protein kinase regulatory subunit 2)
DEFA5 (defensin alpha 5)
RND3 (Rho family GTPase 3)
DHPS (deoxyhypusine synthase)
DLG2 (discs large MAGUK scaffold protein 2)
ASS1 (argininosuccinate synthase 1)
EIF4A1 (eukaryotic translation initiation factor 4A1)
FAH (fumarylacetoacetate hydrolase)
FANCF (FA complementation group F)
FBLN1 (fibulin 1)
FASN (fatty acid synthase)
BCL9 (BCL9 transcription coactivator)
ADAM8 (ADAM metallopeptidase domain 8)
PARP1 (poly(ADP-ribose) polymerase 1)
HK3 (hexokinase 3)
CDH15 (cadherin 15)
IFI16 (interferon gamma inducible protein 16)
IKBKB (inhibitor of nuclear factor kappa B kinase subunit beta)
IRF5 (interferon regulatory factor 5)
CALB2 (calbindin 2)
CCNE1 (cyclin E1)
ADCY7 (adenylate cyclase 7)
CD9 (CD9 molecule)
CD14 (CD14 molecule)
CD86 (CD86 molecule)
CD36 (CD36 molecule)
SCARB1 (scavenger receptor class B member 1)
ADCY8 (adenylate cyclase 8)
ADD1 (adducin 1)
AAMP (angio associated migratory cell protein)
CD40LG (CD40 ligand)
ADD2 (adducin 2)
CDKN3 (cyclin dependent kinase inhibitor 3)
CRYBG1 (crystallin beta-gamma domain containing 1)
ARHGDIA (Rho GDP dissociation inhibitor alpha)
CSNK1A1 (casein kinase 1 alpha 1)
DLG3 (discs large MAGUK scaffold protein 3)
CD38 (CD38 molecule)
CD40 (CD40 molecule)
CD59 (CD59 molecule (CD59 blood group))
CD69 (CD69 molecule)
CLTA (clathrin light chain A)
DAB1 (DAB adaptor protein 1)
ATP4A (ATPase H+/K+ transporting subunit alpha)
ERBB3 (erb-b2 receptor tyrosine kinase 3)
CXCL2 (C-X-C motif chemokine ligand 2)
HLA-B (major histocompatibility complex, class I, B)
NDUFC1 (NADH:ubiquinone oxidoreductase subunit C1)
NFYA (nuclear transcription factor Y subunit alpha)
CDK16 (cyclin dependent kinase 16)
FCER1A (Fc epsilon receptor Ia)
KLF10 (Kruppel like factor 10)
GSTT1 (glutathione S-transferase theta 1)
MTERF1 (mitochondrial transcription termination factor 1)
DLEU2 (deleted in lymphocytic leukemia 2)
FCGBP (Fc gamma binding protein)
LDHA (lactate dehydrogenase A)
SLC6A5 (solute carrier family 6 member 5)
SCAMP1 (secretory carrier membrane protein 1)
TBCC (tubulin folding cofactor C)
TBX15 (T-box transcription factor 15)
ACSL4 (acyl-CoA synthetase long chain family member 4)
FGF2 (fibroblast growth factor 2)
FKBP3 (FKBP prolyl isomerase 3)
FOXF1 (forkhead box F1)
TLE4 (TLE family member 4, transcriptional corepressor)
ACRV1 (acrosomal vesicle protein 1)
TRAF1 (TNF receptor associated factor 1)
TRPC3 (transient receptor potential cation channel subfamily C member 3)
TRPC6 (transient receptor potential cation channel subfamily C member 6)
FUT4 (fucosyltransferase 4)
GBP1 (guanylate binding protein 1)
SLC14A2 (solute carrier family 14 member 2)
MKKS (MKKS centrosomal shuttling protein)
GPR25 (G protein-coupled receptor 25)
TKTL1 (transketolase like 1)
OGT (O-linked N-acetylglucosamine (GlcNAc) transferase)
COPS3 (COP9 signalosome subunit 3)
PDXK (pyridoxal kinase)
STK16 (serine/threonine kinase 16)
PLPP3 (phospholipid phosphatase 3)
ASMTL (acetylserotonin O-methyltransferase like)
IRS2 (insulin receptor substrate 2)
EIF3A (eukaryotic translation initiation factor 3 subunit A)
EIF3F (eukaryotic translation initiation factor 3 subunit F)
RIPK1 (receptor interacting serine/threonine kinase 1)
HUS1 (HUS1 checkpoint clamp component)
ZFP36L1 (ZFP36 ring finger protein like 1)
BTC (betacellulin)
BTD (biotinidase)
ID4 (inhibitor of DNA binding 4, HLH protein)
IDE (insulin degrading enzyme)
IDI1 (isopentenyl-diphosphate delta isomerase 1)
IDUA (alpha-L-iduronidase)
CACNA2D1 (calcium voltage-gated channel auxiliary subunit alpha2delta 1)
IKBKB (inhibitor of nuclear factor kappa B kinase subunit beta)
KCND3 (potassium voltage-gated channel subfamily D member 3)
CD86 (CD86 molecule)
SMAD6 (SMAD family member 6)
MAK (male germ cell associated kinase)
DAP (death associated protein)
CRISP1 (cysteine rich secretory protein 1)
CKMT1B (creatine kinase, mitochondrial 1B)
ABCA3 (ATP binding cassette subfamily A member 3)
COL8A2 (collagen type VIII alpha 2 chain)
CRX (cone-rod homeobox)
MYBL2 (MYB proto-oncogene like 2)
CSH2 (chorionic somatomammotropin hormone 2)
NME3 (NME/NM23 nucleoside diphosphate kinase 3)
OLR1 (oxidized low density lipoprotein receptor 1)
PAFAH1B3 (platelet activating factor acetylhydrolase 1b catalytic subunit 3)
PLEK (pleckstrin)
RPS14 (ribosomal protein S14)
EPHB3 (EPH receptor B3)
ERBB2 (erb-b2 receptor tyrosine kinase 2)
ACADSB (acyl-CoA dehydrogenase short/branched chain)
SNTA1 (syntrophin alpha 1)
STIM1 (stromal interaction molecule 1)
ATP7A (ATPase copper transporting alpha)
TRPM2 (transient receptor potential cation channel subfamily M member 2)
VDAC2 (voltage dependent anion channel 2)
WNT3 (Wnt family member 3)
GYG1 (glycogenin 1)
HADHB (hydroxyacyl-CoA dehydrogenase trifunctional multienzyme complex subunit
ZNF14 (zinc finger protein 14)
ZNF175 (zinc finger protein 175)
HPR (haptoglobin-related protein)
IFT88 (intraflagellar transport 88)
CLTCL1 (clathrin heavy chain like 1)
AOAH (acyloxyacyl hydrolase)
CRK (CRK proto-oncogene, adaptor protein)
KLK3 (kallikrein related peptidase 3)

CIR1 (corepressor interacting with RBPJ, CIR1)
SLC25A44 (solute carrier family 25 member 44)
RAB11FIP3 (RAB11 family interacting protein 3)
IL4 (interleukin 4)
JAG2 (jagged canonical Notch ligand 2)
ADRA1A (adrenoceptor alpha 1A)
MYCN (MYCN proto-oncogene, bHLH transcription factor)
NAP1L4 (nucleosome assembly protein 1 like 4)
FCGRT (Fc gamma receptor and transporter)
ARL2 (ADP ribosylation factor like GTPase 2)
POU3F3 (POU class 3 homeobox 3)
GALNS (galactosamine (N-acetyl)-6-sulfatase)
GGT1 (gamma-glutamyltransferase 1)
RFNG (RFNG O-fucosylpeptide 3-beta-N-acetylglucosaminyltransferase)
RIT2 (Ras like without CAAX 2)
GUSB (glucuronidase beta)
SEC13 (SEC13 homolog, nuclear pore and COPII coat complex component)
SLC5A4 (solute carrier family 5 member 4)
BID (BH3 interacting domain death agonist)
SOX4 (SRY-box transcription factor 4)
GYS1 (glycogen synthase 1)
HBM (hemoglobin subunit mu)
HMGCR (3-hydroxy-3-methylglutaryl-CoA reductase)
ADARB1 (adenosine deaminase RNA specific B1)
HMX2 (H6 family homeobox 2)
HNRNPL (heterogeneous nuclear ribonucleoprotein L)
BCS1L (BCS1 homolog, ubiquinol-cytochrome c reductase complex chaperone)
BGLAP (bone gamma-carboxyglutamate protein)
HSPG2 (heparan sulfate proteoglycan 2)
IFNGR2 (interferon gamma receptor 2)
ITGB7 (integrin subunit beta 7)
ALDH3B1 (aldehyde dehydrogenase 3 family member B1)
CHAD (chondroadherin)
CSH2 (chorionic somatomammotropin hormone 2)
PPARG (peroxisome proliferator activated receptor gamma)
PTPN6 (protein tyrosine phosphatase non-receptor type 6)
RAB5C (RAB5C, member RAS oncogene family)
RAD51B (RAD51 paralog B)
RPS18 (ribosomal protein S18)
RTN2 (reticulon 2)
SPRR2F (small proline rich protein 2F)
HOXD10 (homeobox D10)
HOXD12 (homeobox D12)
IDUA (alpha-L-iduronidase)
USF1 (upstream transcription factor 1)
KCNJ11 (potassium inwardly rectifying channel subfamily J member 11)
MGST2 (microsomal glutathione S-transferase 2)
NRL (neural retina leucine zipper)
SERPINA4 (serpin family A member 4)
PIK3CA (phosphatidylinositol-4,5-bisphosphate 3-kinase catalytic subunit alpha)
NIPSNAP2 (nipsnap homolog 2)
ARRB1 (arrestin beta 1)
SLC3A1 (solute carrier family 3 member 1)
SLC6A11 (solute carrier family 6 member 11)
ATP1A1 (ATPase Na+/K+ transporting subunit alpha 1)
SCGB1A1 (secretoglobin family 1A member 1)
GSTA1 (glutathione S-transferase alpha 1)
YES1 (YES proto-oncogene 1, Src family tyrosine kinase)
ZNF7 (zinc finger protein 7)
ZNF41 (zinc finger protein 41)
HMBS (hydroxymethylbilane synthase)
HMGB2 (high mobility group box 2)
USF2 (upstream transcription factor 2, c-fos interacting)
CXCR2 (C-X-C motif chemokine receptor 2)
INSIG1 (insulin induced gene 1)
IER2 (immediate early response 2)
CD52 (CD52 molecule)
COX4I1 (cytochrome c oxidase subunit 4I1)
MT-CO2 (cytochrome c oxidase subunit II)
MTCP1 (mature T cell proliferation 1)
DHX8 (DEAH-box helicase 8)
PCYT1A (phosphate cytidylyltransferase 1A, choline)
PFKM (phosphofructokinase, muscle)
ASGR1 (asialoglycoprotein receptor 1)
CYP3A7 (cytochrome P450 family 3 subfamily A member 7)
ECI1 (enoyl-CoA delta isomerase 1)
DLG4 (discs large MAGUK scaffold protein 4)
DNASE1 (deoxyribonuclease 1)
DRD3 (dopamine receptor D3)
CXCR5 (C-X-C motif chemokine receptor 5)
EIF2D (eukaryotic translation initiation factor 2D)
BRAF (B-Raf proto-oncogene, serine/threonine kinase)
FES (FES proto-oncogene, tyrosine kinase)
FOXG1 (forkhead box G1)
CASP8 (caspase 8)
FLNB (filamin B)
GABRG2 (gamma-aminobutyric acid type A receptor subunit gamma2)
ALB (albumin)
GLI3 (GLI family zinc finger 3)
CHI3L2 (chitinase 3 like 2)
GNL1 (G protein nucleolar 1 (putative))
GRK4 (G protein-coupled receptor kinase 4)
GRIK4 (glutamate ionotropic receptor kainate type subunit 4)
GSTM1 (glutathione S-transferase mu 1)
GSTM3 (glutathione S-transferase mu 3)
GSTT1 (glutathione S-transferase theta 1)
COL10A1 (collagen type X alpha 1 chain)
HCRTR1 (hypocretin receptor 1)
HLA-DMA (major histocompatibility complex, class II, DM alpha)
HLA-DRB5 (major histocompatibility complex, class II, DR beta 5)
EFNA4 (ephrin A4)
EFNA5 (ephrin A5)
EFNB1 (ephrin B1)
CELSR2 (cadherin EGF LAG seven-pass G-type receptor 2)
MEGF6 (multiple EGF like domains 6)
MEGF9 (multiple EGF like domains 9)
MEGF8 (multiple EGF like domains 8)
EGFR (epidermal growth factor receptor)
EGR2 (early growth response 2)
EGR4 (early growth response 4)
EIF1AX (eukaryotic translation initiation factor 1A X-linked)
EIF2B1 (eukaryotic translation initiation factor 2B subunit alpha)
EPHB2 (EPH receptor B2)
FOXM1 (forkhead box M1)
GBX2 (gastrulation brain homeobox 2)
GFRA2 (GDNF family receptor alpha 2)
C5AR1 (complement C5a receptor 1)
GOLGA1 (golgin A1)
GZMH (granzyme H)
HSD17B10 (hydroxysteroid 17-beta dehydrogenase 10)
CD47 (CD47 molecule)
FOXA3 (forkhead box A3)
IFNA10 (interferon alpha 10)
COL11A1 (collagen type XI alpha 1 chain)
KIR2DS5 (killer cell immunoglobulin like receptor, two Ig domains and short
CRABP2 (cellular retinoic acid binding protein 2)
APOE (apolipoprotein E)
LYZ (lysozyme)
CSTF2 (cleavage stimulation factor subunit 2)
ARSL (arylsulfatase L)
MCM3 (minichromosome maintenance complex component 3)
DDC (dopa decarboxylase)
MGMT (O-6-methylguanine-DNA methyltransferase)
KITLG (KIT ligand)
CITED1 (Cbp/p300 interacting transactivator with Glu/Asp rich carboxy-terminal
DLX3 (distal-less homeobox 3)
DMD (dystrophin)
DUSP4 (dual specificity phosphatase 4)
NFYC (nuclear transcription factor Y subunit gamma)
NPTX1 (neuronal pentraxin 1)
NTF4 (neurotrophin 4)
NTSR1 (neurotensin receptor 1)
P2RX1 (purinergic receptor P2X 1)
P2RX3 (purinergic receptor P2X 3)
P2RX7 (purinergic receptor P2X 7)
P2RY1 (purinergic receptor P2Y1)

PMP2 (peripheral myelin protein 2)
ETV1 (ETS variant transcription factor 1)
RABGGTA (Rab geranylgeranyltransferase subunit alpha)
RGS13 (regulator of G protein signaling 13)
SH3BGR (SH3 domain binding glutamate rich protein)
SLC7A1 (solute carrier family 7 member 1)
SLC7A4 (solute carrier family 7 member 4)
GCLM (glutamate-cysteine ligase modifier subunit)
THPO (thrombopoietin)
GPX4 (glutathione peroxidase 4)
VPREB1 (V-set pre-B cell surrogate light chain 1)
WNT5A (Wnt family member 5A)
GRIK1 (glutamate ionotropic receptor kainate type subunit 1)
GRIN2D (glutamate ionotropic receptor NMDA type subunit 2D)
GSK3A (glycogen synthase kinase 3 alpha)
GSS (glutathione synthetase)
GUCY2D (guanylate cyclase 2D, retinal)
ATP6AP1 (ATPase H+ transporting accessory protein 1)
HDC (histidine decarboxylase)
HEXB (hexosaminidase subunit beta)
HOXA6 (homeobox A6)
ADCY9 (adenylate cyclase 9)
CBFA2T3 (CBFA2/RUNX1 partner transcriptional co-repressor 3)
CCKAR (cholecystokinin A receptor)
CKM (creatine kinase, M-type)
LSS (lanosterol synthase)
MEF2D (myocyte enhancer factor 2D)
CREBL2 (cAMP responsive element binding protein like 2)
NGF (nerve growth factor)
GPR143 (G protein-coupled receptor 143)
PTK2B (protein tyrosine kinase 2 beta)
FGF11 (fibroblast growth factor 11)
FGF14 (fibroblast growth factor 14)
FGFR3 (fibroblast growth factor receptor 3)
FGG (fibrinogen gamma chain)
POR (cytochrome p450 oxidoreductase)
HBA1 (hemoglobin subunit alpha 1)
ALCAM (activated leukocyte cell adhesion molecule)
CENPA (centromere protein A)
KCNF1 (potassium voltage-gated channel modifier subfamily F member 1)
KCNK2 (potassium two pore domain channel subfamily K member 2)
KCNN3 (potassium calcium-activated channel subfamily N member 3)
KCNN4 (potassium calcium-activated channel subfamily N member 4)
KCNQ3 (potassium voltage-gated channel subfamily Q member 3)
KCNS1 (potassium voltage-gated channel modifier subfamily S member 1)
KRAS (KRAS proto-oncogene, GTPase)
KRT2 (keratin 2)
KRT8 (keratin 8)
LCK (LCK proto-oncogene, Src family tyrosine kinase)
LCN1 (lipocalin 1)
MRE11 (MRE11 homolog, double strand break repair nuclease)
MVK (mevalonate kinase)
MYH4 (myosin heavy chain 4)
OPRL1 (opioid related nociceptin receptor 1)
REG3A (regenerating family member 3 alpha)
PLAGL2 (PLAG1 like zinc finger 2)
POU4F1 (POU class 4 homeobox 1)
PPP2R5E (protein phosphatase 2 regulatory subunit B'epsilon)
S100A7 (S100 calcium binding protein A7)
SCNN1G (sodium channel epithelial 1 subunit gamma)
CBLIF (cobalamin binding intrinsic factor)
SRY (sex determining region Y)
P2RY4 (pyrimidinergic receptor P2Y4)
PABPC3 (poly(A) binding protein cytoplasmic 3)
STOM (stomatin)
EPHA1 (EPH receptor A1)
EPHA4 (EPH receptor A4)
EPHA5 (EPH receptor A5)
ASPA (aspartoacylase)
EPHB1 (EPH receptor B1)
CLN8 (CLN8 transmembrane ER and ERGIC protein)
EPO (erythropoietin)
EPRS1 (glutamyl-prolyl-tRNA synthetase 1)
EPS8 (epidermal growth factor receptor pathway substrate 8)
EPS15 (epidermal growth factor receptor pathway substrate 15)
ESD (esterase D)
ESR1 (estrogen receptor 1)
FABP2 (fatty acid binding protein 2)
FABP3 (fatty acid binding protein 3)
FANCF (FA complementation group F)
EFEMP1 (EGF containing fibulin extracellular matrix protein 1)
FOXE1 (forkhead box E1)
AVP (arginine vasopressin)
ACTB (actin beta)
FMO5 (flavin containing dimethylaniline monoxygenase 5)
FMOD (fibromodulin)
FPR2 (formyl peptide receptor 2)
BMP4 (bone morphogenetic protein 4)
LDLRAD4 (low density lipoprotein receptor class A domain containing 4)
GLUD2 (glutamate dehydrogenase 2)
GNG7 (G protein subunit gamma 7)
GRIN2D (glutamate ionotropic receptor NMDA type subunit 2D)
CD247 (CD247 molecule)
CD5 (CD5 molecule)
CETN1 (centrin 1)
CHI3L1 (chitinase 3 like 1)
MBNL1 (muscleblind like splicing regulator 1)
MOG (myelin oligodendrocyte glycoprotein)
MPL (MPL proto-oncogene, thrombopoietin receptor)
MTNR1A (melatonin receptor 1A)
SEPTIN1 (septin 1)
NELL1 (neural EGFL like 1)
NEK1 (NIMA related kinase 1)
NPPB (natriuretic peptide B)
YBX1 (Y-box binding protein 1)
AQP4 (aquaporin 4)
PALM (paralemmin)
PHC1 (polyhomeotic homolog 1)
EIF4EBP1 (eukaryotic translation initiation factor 4E binding protein 1)
PDK2 (pyruvate dehydrogenase kinase 2)
PEG3 (paternally expressed 3)
PFDN2 (prefoldin subunit 2)
PFN2 (profilin 2)
PGD (phosphogluconate dehydrogenase)
DMTN (dematin actin binding protein)
DARS1 (aspartyl-tRNA synthetase 1)
AKR1C2 (aldo-keto reductase family 1 member C2)
DHFR (dihydrofolate reductase)
APOA4 (apolipoprotein A4)
TYMP (thymidine phosphorylase)
GBP3 (guanylate binding protein 3)
FFAR2 (free fatty acid receptor 2)
GPR42 (G protein-coupled receptor 42)
GRK6 (G protein-coupled receptor kinase 6)
MKNK2 (MAPK interacting serine/threonine kinase 2)
BPHL (biphenyl hydrolase like)
BPI (bactericidal permeability increasing protein)
GRB10 (growth factor receptor bound protein 10)
GRB14 (growth factor receptor bound protein 14)
ZFP36L2 (ZFP36 ring finger protein like 2)
RAPGEF1 (Rap guanine nucleotide exchange factor 1)
GRIA1 (glutamate ionotropic receptor AMPA type subunit 1)
GRIK2 (glutamate ionotropic receptor kainate type subunit 2)
ADA (adenosine deaminase)
GUCY1B2 (guanylate cyclase 1 soluble subunit beta 2 (pseudogene))

GTF3C1 (general transcription factor IIIC subunit 1)
GUCA2B (guanylate cyclase activator 2B)
S100G (S100 calcium binding protein G)
HOXB5 (homeobox B5)
HOXB6 (homeobox B6)
CALCA (calcitonin related polypeptide alpha)
CAMK2A (calcium/calmodulin dependent protein kinase II alpha)
HRAS (HRas proto-oncogene, GTPase)
HRC (histidine rich calcium binding protein)
HRG (histidine rich glycoprotein)
CD1C (CD1c molecule)
IL1A (interleukin 1 alpha)
PARP4 (poly(ADP-ribose) polymerase family member 4)
SCARB2 (scavenger receptor class B member 2)
IMPA1 (inositol monophosphatase 1)
KCNH1 (potassium voltage-gated channel subfamily H member 1)
KCNJ5 (potassium inwardly rectifying channel subfamily J member 5)
KCNJ6 (potassium inwardly rectifying channel subfamily J member 6)
LRCH4 (leucine rich repeats and calponin homology domain containing 4)
MAGEA4 (MAGE family member A4)
MANBA (mannosidase beta)
CDH5 (cadherin 5)
CHML (CHM like Rab escort protein)
CLCN6 (chloride voltage-gated channel 6)
NF2 (NF2, moesin-ezrin-radixin like (MERLIN) tumor suppressor)
PCBD1 (pterin-4 alpha-carbinolamine dehydratase 1)
PFKFB3 (6-phosphofructo-2-kinase/fructose-2,6-biphosphatase 3)
COX8A (cytochrome c oxidase subunit 8A)
PGC (progastricsin)
CPD (carboxypeptidase D)
EXOSC10 (exosome component 10)
PMS2 (PMS1 homolog 2, mismatch repair system component)
PRRX1 (paired related homeobox 1)
CSNK1G3 (casein kinase 1 gamma 3)
PPT1 (palmitoyl-protein thioesterase 1)
PRB3 (proline rich protein BstNI subfamily 3)
PRB4 (proline rich protein BstNI subfamily 4)
CTRB1 (chymotrypsinogen B1)
CTRL (chymotrypsin like)
MAP2K7 (mitogen-activated protein kinase kinase 7)
CTSV (cathepsin V)
AMELY (amelogenin Y-linked)
PTGDR (prostaglandin D2 receptor)
PTGDS (prostaglandin D2 synthase)
PTH (parathyroid hormone)
PVR (PVR cell adhesion molecule)
RBBP6 (RB binding protein 6, ubiquitin ligase)
RFC4 (replication factor C subunit 4)
RPS11 (ribosomal protein S11)
CLIP1 (CAP-Gly domain containing linker protein 1)
ARF3 (ADP ribosylation factor 3)
GATM (glycine amidinotransferase)
ATP1B1 (ATPase Na+/K+ transporting subunit beta 1)
ATP2A3 (ATPase sarcoplasmic/endoplasmic reticulum Ca2+ transporting 3)
GLS (glutaminase)
GNS (glucosamine (N-acetyl)-6-sulfatase)
IGSF3 (immunoglobulin superfamily member 3)
IFNA6 (interferon alpha 6)
BDNF (brain derived neurotrophic factor)
PRDM1 (PR/SET domain 1)
JCHAIN (joining chain of multimeric IgA and IgM)
IL1R1 (interleukin 1 receptor type 1)
IL3RA (interleukin 3 receptor subunit alpha)
IL6ST (interleukin 6 cytokine family signal transducer)
CXCL8 (C-X-C motif chemokine ligand 8)
BUB1B (BUB1 mitotic checkpoint serine/threonine kinase B)
JUN (Jun proto-oncogene, AP-1 transcription factor subunit)
JUND (JunD proto-oncogene, AP-1 transcription factor subunit)
KCNJ15 (potassium inwardly rectifying channel subfamily J member 15)
LSAMP (limbic system associated membrane protein)
LSP1 (lymphocyte specific protein 1)
LTA4H (leukotriene A4 hydrolase)
LTB (lymphotoxin beta)

CYP4F3 (cytochrome P450 family 4 subfamily F member 3)
LTBP1 (latent transforming growth factor beta binding protein 1)
LTK (leukocyte receptor tyrosine kinase)
LY6E (lymphocyte antigen 6 family member E)
LY6H (lymphocyte antigen 6 family member H)
CD1D (CD1d molecule)
CD19 (CD19 molecule)
MAF (MAF bZIP transcription factor)
MAGEB3 (MAGE family member B3)
CDR1 (cerebellar degeneration related protein 1)
MSH3 (mutS homolog 3)
CEACAM8 (CEA cell adhesion molecule 8)
MYH10 (myosin heavy chain 10)
MYH9 (myosin heavy chain 9)
MYO7B (myosin VIIB)
NDUFB1 (NADH:ubiquinone oxidoreductase subunit B1)
AHSG (alpha 2-HS glycoprotein)
PCK1 (phosphoenolpyruvate carboxykinase 1)
PDE6D (phosphodiesterase 6D)
PITX2 (paired like homeodomain 2)
PMCHL2 (pro-melanin concentrating hormone like 2 (pseudogene))
PNLIPRP1 (pancreatic lipase related protein 1)
ALDOB (aldolase, fructose-bisphosphate B)
RAD9A (RAD9 checkpoint clamp component A)
CPA3 (carboxypeptidase A3)
MOK (MOK protein kinase)
RTKN (rhotekin)
DAD1 (defender against cell death 1)
S100A9 (S100 calcium binding protein A9)
SALL2 (spalt like transcription factor 2)
SCN4B (sodium voltage-gated channel beta subunit 4)
GCG (glucagon)
SCNN1A (sodium channel epithelial 1 subunit alpha)
TIMM8A (translocase of inner mitochondrial membrane 8A)
SLC1A4 (solute carrier family 1 member 4)
SLC12A3 (solute carrier family 12 member 3)
DPH1 (diphthamide biosynthesis 1)
SOX12 (SRY-box transcription factor 12)
SRD5A1 (steroid 5 alpha-reductase 1)
E2F5 (E2F transcription factor 5)
SYCP1 (synaptonemal complex protein 1)
APOC2 (apolipoprotein C2)
CNTN2 (contactin 2)
TBL1X (transducin beta like 1 X-linked)
EPHX2 (epoxide hydrolase 2)
WARS1 (tryptophanyl-tRNA synthetase 1)
WEE1 (WEE1 G2 checkpoint kinase)
ZNF3 (zinc finger protein 3)
FECH (ferrochelatase)
ZSCAN9 (zinc finger and SCAN domain containing 9)
ZNF202 (zinc finger protein 202)
ZNF226 (zinc finger protein 226)
AOC2 (amine oxidase copper containing 2)
DCK (deoxycytidine kinase)
ACP1 (acid phosphatase 1)
DLG1 (discs large MAGUK scaffold protein 1)
DPAGT1 (dolichyl-phosphate N-acetylglucosaminephosphotransferase 1)
AZU1 (azurocidin 1)
B2M (beta-2-microglobulin)
PLIN2 (perilipin 2)
BMPR1A (bone morphogenetic protein receptor type 1A)
ADGRE1 (adhesion G protein-coupled receptor E1)
ADH6 (alcohol dehydrogenase 6 (class V))
CAST (calpastatin)
F10 (coagulation factor X)
AFM (afamin)
FNTA (farnesyltransferase, CAAX box, alpha)
FPR3 (formyl peptide receptor 3)
FUS (FUS RNA binding protein)
CLN3 (CLN3 lysosomal/endosomal transmembrane protein, battenin)
COL6A1 (collagen type VI alpha 1 chain)
NPBWR1 (neuropeptides B and W receptor 1)
CSF3 (colony stimulating factor 3)

GRM4 (glutamate metabotropic receptor 4)
CXCL2 (C-X-C motif chemokine ligand 2)
DIO2 (iodothyronine deiodinase 2)
FCGR3B (Fc gamma receptor IIIb)
GATA4 (GATA binding protein 4)
PAFAH2 (platelet activating factor acetylhydrolase 2)
SSU72 (SSU72 homolog, RNA polymerase II CTD phosphatase)
HAL (histidine ammonia-lyase)
HK3 (hexokinase 3)
IFNA13 (interferon alpha 13)
IL2RB (interleukin 2 receptor subunit beta)
IL2RG (interleukin 2 receptor subunit gamma)
IL10 (interleukin 10)
IRF7 (interferon regulatory factor 7)
ITGAD (integrin subunit alpha D)
KCNC3 (potassium voltage-gated channel subfamily C member 3)
KIF5A (kinesin family member 5A)
SMAD3 (SMAD family member 3)
MAX (MYC associated factor X)
MEF2A (myocyte enhancer factor 2A)
MFAP3 (microfibril associated protein 3)
ADORA3 (adenosine A3 receptor)
ALDH6A1 (aldehyde dehydrogenase 6 family member A1)
SEPTIN7 (septin 7)
NPR1 (natriuretic peptide receptor 1)
CDH17 (cadherin 17)
OR1D2 (olfactory receptor family 1 subfamily D member 2)
PI3 (peptidase inhibitor 3)
SERPINE2 (serpin family E member 2)
PSEN2 (presenilin 2)
RARRES1 (retinoic acid receptor responder 1)
REL (REL proto-oncogene, NF-kB subunit)
PRPH2 (peripherin 2)
RNY1 (RNA, Ro60-associated Y1)
RP1 (RP1 axonemal microtubule associated)
SERPINB4 (serpin family B member 4)
CCL18 (C-C motif chemokine ligand 18)
SFTPD (surfactant protein D)
STXBP1 (syntaxin binding protein 1)
CREBL2 (cAMP responsive element binding protein like 2)
TNFAIP3 (TNF alpha induced protein 3)
UBE2G1 (ubiquitin conjugating enzyme E2 G1)
XPNPEP1 (X-prolyl aminopeptidase 1)
ZNF185 (zinc finger protein 185 with LIM domain)
ZNF215 (zinc finger protein 215)
MLLT10 (MLLT10 histone lysine methyltransferase DOT1L cofactor)
PTP4A2 (protein tyrosine phosphatase 4A2)
POU1F1 (POU class 1 homeobox 1)
PPP1R10 (protein phosphatase 1 regulatory subunit 10)
PPP2R1B (protein phosphatase 2 scaffold subunit Abeta)
PPP2R2A (protein phosphatase 2 regulatory subunit Balpha)
PPP2R2B (protein phosphatase 2 regulatory subunit Bbeta)
PPP2R5A (protein phosphatase 2 regulatory subunit B'alpha)
PPP3CC (protein phosphatase 3 catalytic subunit gamma)
PSMA3 (proteasome 20S subunit alpha 3)
PSMD12 (proteasome 26S subunit, non-ATPase 12)
PTPRA (protein tyrosine phosphatase receptor type A)
CRYM (crystallin mu)
RAB4A (RAB4A, member RAS oncogene family)
RAD23B (RAD23 homolog B, nucleotide excision repair protein)
RAD51 (RAD51 recombinase)
TEX28 (testis expressed 28)
ARID4A (AT-rich interaction domain 4A)
RFC1 (replication factor C subunit 1)
ALAS1 (5'-aminolevulinate synthase 1)
RPGR (retinitis pigmentosa GTPase regulator)
CCL15 (C-C motif chemokine ligand 15)
CCL17 (C-C motif chemokine ligand 17)
SFPQ (splicing factor proline and glutamine rich)
SFRP5 (secreted frizzled related protein 5)
E2F4 (E2F transcription factor 4)
SLC1A7 (solute carrier family 1 member 7)
SLC2A3 (solute carrier family 2 member 3)

EMD (emerin)
SOD1 (superoxide dismutase 1)
SPRR2B (small proline rich protein 2B)
SPRR2E (small proline rich protein 2E)
NUDT2 (nudix hydrolase 2)
ETV2 (ETS variant transcription factor 2)
SSTR4 (somatostatin receptor 4)
TAT (tyrosine aminotransferase)
ELOB (elongin B)
ZNF12 (zinc finger protein 12)
GALK1 (galactokinase 1)
ZNF84 (zinc finger protein 84)
SLBP (stem-loop binding protein)
ST8SIA4 (ST8 alpha-N-acetyl-neuraminide alpha-2,8-sialyltransferase 4)
GDF1 (growth differentiation factor 1)
PSCA (prostate stem cell antigen)
TCL1A (TCL1 family AKT coactivator A)
GPC1 (glypican 1)
SYN3 (synapsin III)
GSK3B (glycogen synthase kinase 3 beta)
H2AC13 (H2A clustered histone 13)
HGF (hepatocyte growth factor)
CDC42BPA (CDC42 binding protein kinase alpha)
PPFIBP2 (PPFIA binding protein 2)
ENC1 (ectodermal-neural cortex 1)
BCAS1 (brain enriched myelin associated protein 1)
HNF4G (hepatocyte nuclear factor 4 gamma)
CDC14B (cell division cycle 14B)
FAM193A (family with sequence similarity 193 member A)
HOXA13 (homeobox A13)
EIF3C (eukaryotic translation initiation factor 3 subunit C)
S1PR4 (sphingosine-1-phosphate receptor 4)
HSPA5 (heat shock protein family A (Hsp70) member 5)
INPP4B (inositol polyphosphate-4-phosphatase type II B)
FOXN2 (forkhead box N2)
HTN1 (histatin 1)
MCM3AP (minichromosome maintenance complex component 3 associated protein)
ATP6V0E1 (ATPase H+ transporting V0 subunit e1)
PGLYRP1 (peptidoglycan recognition protein 1)
PKMYT1 (protein kinase, membrane associated tyrosine/threonine 1)
IDS (iduronate 2-sulfatase)
IL18 (interleukin 18)
SLC22A13 (solute carrier family 22 member 13)
KLRC2 (killer cell lectin like receptor C2)
KPNA5 (karyopherin subunit alpha 5)
CIR1 (corepressor interacting with RBPJ, CIR1)
IGDCC3 (immunoglobulin superfamily DCC subclass member 3)
RAB36 (RAB36, member RAS oncogene family)
USP15 (ubiquitin specific peptidase 15)
RAD50 (RAD50 double strand break repair protein)
PREB (prolactin regulatory element binding)
RASGRP1 (RAS guanyl releasing protein 1)
PLXNC1 (plexin C1)
ZNF256 (zinc finger protein 256)
MSX1 (msh homeobox 1)
SF3B4 (splicing factor 3b subunit 4)
MYO5B (myosin VB)
BAIAP2 (BAR/IMD domain containing adaptor protein 2)
NDUFB10 (NADH:ubiquinone oxidoreductase subunit B10)
NDUFV2 (NADH:ubiquinone oxidoreductase core subunit V2)
B3GNT2 (UDP-GlcNAc:betaGal beta-1,3-N-acetylglucosaminyltransferase 2)
DLL3 (delta like canonical Notch ligand 3)
VAX1 (ventral anterior homeobox 1)
DUSP14 (dual specificity phosphatase 14)
HSF2BP (heat shock transcription factor 2 binding protein)
CHRND (cholinergic receptor nicotinic delta subunit)
PHYH (phytanoyl-CoA 2-hydroxylase)
PLRG1 (pleiotropic regulator 1)
POLR2L (RNA polymerase II, I and III subunit L)
ACKR2 (atypical chemokine receptor 2)
AMY1B (amylase alpha 1B)
CYP11B2 (cytochrome P450 family 11 subfamily B member 2)
CYP24A1 (cytochrome P450 family 24 subfamily A member 1)

DEFA1 (defensin alpha 1)
ASMT (acetylserotonin O-methyltransferase)
EEF1G (eukaryotic translation elongation factor 1 gamma)
EIF5 (eukaryotic translation initiation factor 5)
ACSL3 (acyl-CoA synthetase long chain family member 3)
FANCB (FA complementation group B)
FAP (fibroblast activation protein alpha)
FBLN1 (fibulin 1)
FASN (fatty acid synthase)
FAT1 (FAT atypical cadherin 1)
BCL9 (BCL9 transcription coactivator)
FAU (FAU ubiquitin like and ribosomal protein S30 fusion)
FCER2 (Fc epsilon receptor II)
ADCY2 (adenylate cyclase 2)
GAPDH (glyceraldehyde-3-phosphate dehydrogenase)
GFI1 (growth factor independent 1 transcriptional repressor)
CASP5 (caspase 5)
GPT (glutamic--pyruvic transaminase)
GRN (granulin precursor)
HCFC1 (host cell factor C1)
HNRNPF (heterogeneous nuclear ribonucleoprotein F)
CS (citrate synthase)
HOXC8 (homeobox C8)
HOXC10 (homeobox C10)
CDK2 (cyclin dependent kinase 2)
ADRA2C (adrenoceptor alpha 2C)
ADSL (adenylosuccinate lyase)
CCN1 (cellular communication network factor 1)
IL7R (interleukin 7 receptor)
IRAK2 (interleukin 1 receptor associated kinase 2)
IRF2 (interferon regulatory factor 2)
CHRM3 (cholinergic receptor muscarinic 3)
IRS1 (insulin receptor substrate 1)
IVL (involucrin)
CLC (Charcot-Leyden crystal galectin)
KCNJ9 (potassium inwardly rectifying channel subfamily J member 9)
KEL (Kell metallo-endopeptidase (Kell blood group))
KIR2DL1 (killer cell immunoglobulin like receptor, two Ig domains and long cytoplasmic
LRP3 (LDL receptor related protein 3)
CCNE1 (cyclin E1)
CCNF (cyclin F)
CCNT2 (cyclin T2)
CD3E (CD3 epsilon subunit of T-cell receptor complex)
CD8B (CD8b molecule)
CHRM4 (cholinergic receptor muscarinic 4)
CD14 (CD14 molecule)
CD22 (CD22 molecule)
HAND1 (heart and neural crest derivatives expressed 1)
SIGLEC6 (sialic acid binding Ig like lectin 6)
CD34 (CD34 molecule)
CD36 (CD36 molecule)
SCARB1 (scavenger receptor class B member 1)
RPH3AL (rabphilin 3A like (without C2 domains))
ADCY8 (adenylate cyclase 8)
AAMP (angio associated migratory cell protein)
AANAT (aralkylamine N-acetyltransferase)
ENTPD3 (ectonucleoside triphosphate diphosphohydrolase 3)
ADD2 (adducin 2)
CD72 (CD72 molecule)
CDK3 (cyclin dependent kinase 3)
CDK6 (cyclin dependent kinase 6)
ALK (ALK receptor tyrosine kinase)
CCR1 (C-C motif chemokine receptor 1)
CRYGS (crystallin gamma S)
PMS2P4 (PMS1 homolog 2, mismatch repair system component pseudogene 4)
PPID (peptidylprolyl isomerase D)
CD55 (CD55 molecule (Cromer blood group))
AKR1C1 (aldo-keto reductase family 1 member C1)
GSDME (gasdermin E)
DOCK1 (dedicator of cytokinesis 1)
DOCK2 (dedicator of cytokinesis 2)
DST (dystonin)
E2F2 (E2F transcription factor 2)

ECH1 (enoyl-CoA hydratase 1)
ECM1 (extracellular matrix protein 1)
CD38 (CD38 molecule)
ENTPD1 (ectonucleoside triphosphate diphosphohydrolase 1)
CRP (C-reactive protein)
CD40 (CD40 molecule)
IER2 (immediate early response 2)
CD44 (CD44 molecule (Indian blood group))
NCOR1 (nuclear receptor corepressor 1)
CD48 (CD48 molecule)
CD58 (CD58 molecule)
CD59 (CD59 molecule (CD59 blood group))
CD69 (CD69 molecule)
CD52 (CD52 molecule)
HLCS (holocarboxylase synthetase)
COL5A1 (collagen type V alpha 1 chain)
CPT2 (carnitine palmitoyltransferase 2)
CRYGB (crystallin gamma B)
AQP5 (aquaporin 5)
CSNK1D (casein kinase 1 delta)
CYP2A13 (cytochrome P450 family 2 subfamily A member 13)
ARCN1 (archain 1)
ASL (argininosuccinate lyase)
GET3 (guided entry of tail-anchored proteins factor 3, ATPase)
ATP6V1B1 (ATPase H+ transporting V1 subunit B1)
BCL2L1 (BCL2 like 1)
ETS1 (ETS proto-oncogene 1, transcription factor)
EWSR1 (EWS RNA binding protein 1)
FABP7 (fatty acid binding protein 7)
FUT2 (fucosyltransferase 2)
GABRA3 (gamma-aminobutyric acid type A receptor subunit alpha3)
ARHGAP35 (Rho GTPase activating protein 35)
ADH1C (alcohol dehydrogenase 1C (class I), gamma polypeptide)
CCIN (calicin)
HLA-DPB1 (major histocompatibility complex, class II, DP beta 1)
HLA-DRB4 (major histocompatibility complex, class II, DR beta 4)
CD37 (CD37 molecule)
RAB32 (RAB32, member RAS oncogene family)
COL4A6 (collagen type IV alpha 6 chain)
NFIA (nuclear factor I A)
NFIC (nuclear factor I C)
NFYB (nuclear transcription factor Y subunit beta)
NOS3 (nitric oxide synthase 3)
CRYBA2 (crystallin beta A2)
P2RX5 (purinergic receptor P2X 5)
CNTN3 (contactin 3)
PCDH7 (protocadherin 7)
PEX7 (peroxisomal biogenesis factor 7)
ARF5 (ADP ribosylation factor 5)
MAP2K6 (mitogen-activated protein kinase kinase 6)
EEF1B2 (eukaryotic translation elongation factor 1 beta 2)
SEMA3F (semaphorin 3F)
SNAPC1 (small nuclear RNA activating complex polypeptide 1)
SPP1 (secreted phosphoprotein 1)
ATP1B2 (ATPase Na+/K+ transporting subunit beta 2)
NR2F2 (nuclear receptor subfamily 2 group F member 2)
G6PD (glucose-6-phosphate dehydrogenase)
KLF10 (Kruppel like factor 10)
GALT (galactose-1-phosphate uridylyltransferase)
TNFRSF1B (TNF receptor superfamily member 1B)
VASP (vasodilator stimulated phosphoprotein)
HCLS1 (hematopoietic cell-specific Lyn substrate 1)
DEK (DEK proto-oncogene)
HMGB2 (high mobility group box 2)
FZD3 (frizzled class receptor 3)
SLC7A5 (solute carrier family 7 member 5)
HSPB1 (heat shock protein family B (small) member 1)
PLA2G10 (phospholipase A2 group X)
IFNA5 (interferon alpha 5)
DYRK3 (dual specificity tyrosine phosphorylation regulated kinase 3)
KHSRP (KH-type splicing regulatory protein)
RGS20 (regulator of G protein signaling 20)
STX11 (syntaxin 11)

IRF5 (interferon regulatory factor 5)
CAPS (calcyphosine)
FPGT (fucose-1-phosphate guanylyltransferase)
ACVRL1 (activin A receptor like type 1)
PEX11A (peroxisomal biogenesis factor 11 alpha)
DLEU2 (deleted in lymphocytic leukemia 2)
MTMR3 (myotubularin related protein 3)
CDK5R2 (cyclin dependent kinase 5 regulatory subunit 2)
LCK (LCK proto-oncogene, Src family tyrosine kinase)
KALRN (kalirin RhoGEF kinase)
LMO7 (LIM domain 7)
NBR1 (NBR1 autophagy cargo receptor)
SNORD30 (small nucleolar RNA, C/D box 30)
TNFRSF8 (TNF receptor superfamily member 8)
CD68 (CD68 molecule)
CDYL (chromodomain Y like)
RAB8A (RAB8A, member RAS oncogene family)
MEOX2 (mesenchyme homeobox 2)
ONECUT2 (one cut homeobox 2)
NPEPPS (aminopeptidase puromycin sensitive)
GMFG (glia maturation factor gamma)
PTGES (prostaglandin E synthase)
TP53I11 (tumor protein p53 inducible protein 11)
TP53I3 (tumor protein p53 inducible protein 3)
FAM53B (family with sequence similarity 53 member B)
IMPA2 (inositol monophosphatase 2)
IRF4 (interferon regulatory factor 4)
CAV3 (caveolin 3)
ITGB4 (integrin subunit beta 4)
LGI1 (leucine rich glioma inactivated 1)
PSMF1 (proteasome inhibitor subunit 1)
ADGRE5 (adhesion G protein-coupled receptor E5)
KPNA4 (karyopherin subunit alpha 4)
CDKN3 (cyclin dependent kinase inhibitor 3)
KRTAP5-9 (keratin associated protein 5-9)
KRT31 (keratin 31)
KRT33A (keratin 33A)
KRT35 (keratin 35)
LAMA3 (laminin subunit alpha 3)
LAMB2 (laminin subunit beta 2)
STMN1 (stathmin 1)
CHD3 (chromodomain helicase DNA binding protein 3)
TACSTD2 (tumor associated calcium signal transducer 2)
CCR4 (C-C motif chemokine receptor 4)
LTB4R (leukotriene B4 receptor)
CD46 (CD46 molecule)
AGA (aspartylglucosaminidase)
MECP2 (methyl-CpG binding protein 2)
DHX15 (DEAH-box helicase 15)
MT2A (metallothionein 2A)
DHCR7 (7-dehydrocholesterol reductase)
MYH7 (myosin heavy chain 7)
NFATC3 (nuclear factor of activated T cells 3)
NHS (NHS actin remodeling regulator)
BIRC2 (baculoviral IAP repeat containing 2)
SERPINB5 (serpin family B member 5)
FXYD1 (FXYD domain containing ion transport regulator 1)
FDPS (farnesyl diphosphate synthase)
FGFR1 (fibroblast growth factor receptor 1)
PPP1R2 (protein phosphatase 1 regulatory inhibitor subunit 2)
MAP2K2 (mitogen-activated protein kinase kinase 2)
PTPN14 (protein tyrosine phosphatase non-receptor type 14)
PTPRB (protein tyrosine phosphatase receptor type B)
REN (renin)
RFPL1 (ret finger protein like 1)
HLA-L (major histocompatibility complex, class I, L (pseudogene))
HOXA6 (homeobox A6)
HOXC5 (homeobox C5)
SCML1 (Scm polycomb group protein like 1)
SHB (SH2 domain containing adaptor protein B)
PCDH20 (protocadherin 20)
SLC6A6 (solute carrier family 6 member 6)
SOX4 (SRY-box transcription factor 4)

SP3 (Sp3 transcription factor)
GYPB (glycophorin B (MNS blood group))
H3-3B (H3.3 histone B)
HAGH (hydroxyacylglutathione hydrolase)
HBM (hemoglobin subunit mu)
HLA-DRA (major histocompatibility complex, class II, DR alpha)
HLA-DRB3 (major histocompatibility complex, class II, DR beta 3)
BAG1 (BAG cochaperone 1)
HMBS (hydroxymethylbilane synthase)
HMX1 (H6 family homeobox 1)
HMX2 (H6 family homeobox 2)
HPCAL1 (hippocalcin like 1)
HSD11B2 (hydroxysteroid 11-beta dehydrogenase 2)
HSPD1 (heat shock protein family D (Hsp60) member 1)
BLVRB (biliverdin reductase B)
ADORA1 (adenosine A1 receptor)
IBSP (integrin binding sialoprotein)
CFI (complement factor I)
IFNAR1 (interferon alpha and beta receptor subunit 1)
ITGB7 (integrin subunit beta 7)
IPO5 (importin 5)
LGALS2 (galectin 2)
LTF (lactotransferrin)
MARCKS (myristoylated alanine rich protein kinase C substrate)
CLTB (clathrin light chain B)
CNGA4 (cyclic nucleotide gated channel subunit alpha 4)
SLC25A5 (solute carrier family 25 member 5)
MT1G (metallothionein 1G)
MYB (MYB proto-oncogene, transcription factor)
AREG (amphiregulin)
NAGA (alpha-N-acetylgalactosaminidase)
ARHGAP1 (Rho GTPase activating protein 1)
NIT1 (nitrilase 1)
EPOR (erythropoietin receptor)
ERCC2 (ERCC excision repair 2, TFIIH core complex helicase subunit)
ESR2 (estrogen receptor 2)
PEBP1 (phosphatidylethanolamine binding protein 1)
PITX1 (paired like homeodomain 1)
PLAGL2 (PLAG1 like zinc finger 2)
FPGS (folylpolyglutamate synthase)
FAM215A (family with sequence similarity 215 member A)
PLG (plasminogen)
PSMB1 (proteasome 20S subunit beta 1)
PSMB5 (proteasome 20S subunit beta 5)
GALNT2 (polypeptide N-acetylgalactosaminyltransferase 2)
NIPSNAP2 (nipsnap homolog 2)
GCH1 (GTP cyclohydrolase 1)
GGT1 (gamma-glutamyltransferase 1)
RLN1 (relaxin 1)
S100A8 (S100 calcium binding protein A8)
GPR15 (G protein-coupled receptor 15)
SCN1B (sodium voltage-gated channel beta subunit 1)
SCN2B (sodium voltage-gated channel beta subunit 2)
SCN3A (sodium voltage-gated channel alpha subunit 3)
SEMG2 (semenogelin 2)
GRIK3 (glutamate ionotropic receptor kainate type subunit 3)
SIM1 (SIM bHLH transcription factor 1)
SLC6A12 (solute carrier family 6 member 12)
SLC8A1 (solute carrier family 8 member A1)
SUMO3 (small ubiquitin like modifier 3)
SNRPA (small nuclear ribonucleoprotein polypeptide A)
SNTB1 (syntrophin beta 1)
HOXC12 (homeobox C12)
HOXC11 (homeobox C11)
HOXD10 (homeobox D10)
HOXD11 (homeobox D11)
HOXD12 (homeobox D12)
CEACAM1 (CEA cell adhesion molecule 1)
OPN1SW (opsin 1, short wave sensitive)
DNAJB1 (DnaJ heat shock protein family (Hsp40) member B1)
BST2 (bone marrow stromal cell antigen 2)
IFI16 (interferon gamma inducible protein 16)
IFNA8 (interferon alpha 8)

IMPDH1 (inosine monophosphate dehydrogenase 1)
INS (insulin)
CAD (carbamoyl-phosphate synthetase 2, aspartate transcarbamylase, and
CAMLG (calcium modulating ligand)
JAK2 (Janus kinase 2)
CASP9 (caspase 9)
KCNA5 (potassium voltage-gated channel subfamily A member 5)
KLKB1 (kallikrein B1)
MMP1 (matrix metallopeptidase 1)
TBC1D25 (TBC1 domain family member 25)
CDK17 (cyclin dependent kinase 17)
DUSP10 (dual specificity phosphatase 10)
PGGT1B (protein geranylgeranyltransferase type I subunit beta)
SERPINB13 (serpin family B member 13)
PIN1 (peptidylprolyl cis/trans isomerase, NIMA-interacting 1)
AEBP1 (AE binding protein 1)
PPP2R5C (protein phosphatase 2 regulatory subunit B'gamma)
AS3MT (arsenite methyltransferase)
PTMA (prothymosin alpha)
RPN2 (ribophorin II)
SORT1 (sortilin 1)
S100A3 (S100 calcium binding protein A3)
EEF1D (eukaryotic translation elongation factor 1 delta)
SLC6A7 (solute carrier family 6 member 7)
SLC11A2 (solute carrier family 11 member 2)
TAF6 (TATA-box binding protein associated factor 6)
ATP5F1E (ATP synthase F1 subunit epsilon)
HNF1B (HNF1 homeobox B)
TLL1 (tolloid like 1)
GCNT2 (glucosaminyl (N-acetyl) transferase 2 (I blood group))
TPD52L1 (TPD52 like 1)
CNBP (CCHC-type zinc finger nucleic acid binding protein)
ZNF10 (zinc finger protein 10)
ZNF182 (zinc finger protein 182)
ZSCAN20 (zinc finger and SCAN domain containing 20)
HARS1 (histidyl-tRNA synthetase 1)
HLA-B (major histocompatibility complex, class I, B)
HLA-DOA (major histocompatibility complex, class II, DO alpha)
HLA-DQB1 (major histocompatibility complex, class II, DQ beta 1)
VEZF1 (vascular endothelial zinc finger 1)
BSND (barttin CLCNK type accessory subunit beta)
DAP3 (death associated protein 3)
HPCA (hippocalcin)
HAS2 (hyaluronan synthase 2)
HNRNPA1 (heterogeneous nuclear ribonucleoprotein A1)
SPTBN1 (spectrin beta, non-erythrocytic 1)
HTR2C (5-hydroxytryptamine receptor 2C)
VPS51 (VPS51 subunit of GARP complex)
IHH (Indian hedgehog signaling molecule)
ACAN (aggrecan)
PAPSS1 (3'-phosphoadenosine 5'-phosphosulfate synthase 1)
INSL4 (insulin like 4)
CD70 (CD70 molecule)
KHK (ketohexokinase)
CDX4 (caudal type homeobox 4)
LCP2 (lymphocyte cytosolic protein 2)
CIDEA (cell death inducing DFFA like effector a)
MAGEA9 (MAGE family member A9)
MAS1 (MAS1 proto-oncogene, G protein-coupled receptor)
MID1 (midline 1)
CTSK (cathepsin K)
MSMB (microseminoprotein beta)
MTCP1 (mature T cell proliferation 1)
NDUFS3 (NADH:ubiquinone oxidoreductase core subunit S3)
NFKB1 (nuclear factor kappa B subunit 1)
ANGPT2 (angiopoietin 2)
GRIN1 (glutamate ionotropic receptor NMDA type subunit 1)
NFKBIA (NFKB inhibitor alpha)
CNOT2 (CCR4-NOT transcription complex subunit 2)
DGKA (diacylglycerol kinase alpha)
CNOT3 (CCR4-NOT transcription complex subunit 3)
DGKB (diacylglycerol kinase beta)
DDX10 (DEAD-box helicase 10)

PRDX1 (peroxiredoxin 1)
PAH (phenylalanine hydroxylase)
DLX1 (distal-less homeobox 1)
PENK (proenkephalin)
EDN2 (endothelin 2)
EMP3 (epithelial membrane protein 3)
ETV1 (ETS variant transcription factor 1)
ETV3 (ETS variant transcription factor 3)
RAB27B (RAB27B, member RAS oncogene family)
RABGGTA (Rab geranylgeranyltransferase subunit alpha)
RABGGTB (Rab geranylgeranyltransferase subunit beta)
RAC3 (Rac family small GTPase 3)
ARHGDIG (Rho GDP dissociation inhibitor gamma)
FGF10 (fibroblast growth factor 10)
RPL23A (ribosomal protein L23a)
RPS8 (ribosomal protein S8)
FSHR (follicle stimulating hormone receptor)
GABRD (gamma-aminobutyric acid type A receptor subunit delta)
SLC6A11 (solute carrier family 6 member 11)
STX5 (syntaxin 5)
GNGT1 (G protein subunit gamma transducin 1)
GNGT2 (G protein subunit gamma transducin 2)
SULT1A1 (sulfotransferase family 1A member 1)
TAF12 (TATA-box binding protein associated factor 12)
TARBP1 (TAR (HIV-1) RNA binding protein 1)
ZEB1 (zinc finger E-box binding homeobox 1)
GP1BA (glycoprotein Ib platelet subunit alpha)
THPO (thrombopoietin)
GRIK5 (glutamate ionotropic receptor kainate type subunit 5)
GRINA (glutamate ionotropic receptor NMDA type subunit associated protein 1)
BCYRN1 (brain cytoplasmic RNA 1)
GTF2B (general transcription factor IIB)
TNFSF4 (TNF superfamily member 4)
COL14A1 (collagen type XIV alpha 1 chain)
VRK2 (VRK serine/threonine kinase 2)
WNT10B (Wnt family member 10B)
MCHR1 (melanin concentrating hormone receptor 1)
GRIN2C (glutamate ionotropic receptor NMDA type subunit 2C)
C1GALT1C1 (C1GALT1 specific chaperone 1)
STXBP6 (syntaxin binding protein 6)
GRM6 (glutamate metabotropic receptor 6)
GTF2E1 (general transcription factor IIE subunit 1)
GTF2H1 (general transcription factor IIH subunit 1)
GTF3C2 (general transcription factor IIIC subunit 2)
GUCA1B (guanylate cyclase activator 1B)
GUCY2C (guanylate cyclase 2C)
GYPC (glycophorin C (Gerbich blood group))
GYPE (glycophorin E (MNS blood group))
H1-3 (H1.3 linker histone, cluster member)
H2AX (H2A.X variant histone)
HFE (homeostatic iron regulator)
BCAT1 (branched chain amino acid transaminase 1)
BCKDHA (branched chain keto acid dehydrogenase E1 subunit alpha)
HTR1D (5-hydroxytryptamine receptor 1D)
ITGAL (integrin subunit alpha L)
IVD (isovaleryl-CoA dehydrogenase)
KCNA3 (potassium voltage-gated channel subfamily A member 3)
CKM (creatine kinase, M-type)
MAN1A1 (mannosidase alpha class 1A member 1)
MAN2A2 (mannosidase alpha class 2A member 2)
MAOB (monoamine oxidase B)
MAZ (MYC associated zinc finger protein)
MDFI (MyoD family inhibitor)
CNN1 (calponin 1)
MEIS2 (Meis homeobox 2)
FOXO4 (forkhead box O4)
MMP2 (matrix metallopeptidase 2)
CRIP2 (cysteine rich protein 2)
MT-ATP6 (ATP synthase F0 subunit 6)
MX1 (MX dynamin like GTPase 1)
CYP4A11 (cytochrome P450 family 4 subfamily A member 11)
NDUFA8 (NADH:ubiquinone oxidoreductase subunit A8)
DES (desmin)

NDUFA9 (NADH:ubiquinone oxidoreductase subunit A9)
NEFM (neurofilament medium chain)
NMBR (neuromedin B receptor)
OAS1 (2'-5'-oligoadenylate synthetase 1)
E2F1 (E2F transcription factor 1)
ORC2 (origin recognition complex subunit 2)
PAX1 (paired box 1)
PBX1 (PBX homeobox 1)
PDK3 (pyruvate dehydrogenase kinase 3)
EXT2 (exostosin glycosyltransferase 2)
PHEX (phosphate regulating endopeptidase homolog X-linked)
PIK3CD (phosphatidylinositol-4,5-bisphosphate 3-kinase catalytic subunit delta)
PIK3CG (phosphatidylinositol-4,5-bisphosphate 3-kinase catalytic subunit gamma)
PMP22 (peripheral myelin protein 22)
POU4F3 (POU class 4 homeobox 3)
BLOC1S1 (biogenesis of lysosomal organelles complex 1 subunit 1)
PPP1CA (protein phosphatase 1 catalytic subunit alpha)
DPPA4 (developmental pluripotency associated 4)
PPP2R2C (protein phosphatase 2 regulatory subunit Bgamma)
PPP2R5B (protein phosphatase 2 regulatory subunit B'beta)
GJA3 (gap junction protein alpha 3)
PRKCA (protein kinase C alpha)
PSMC1 (proteasome 26S subunit, ATPase 1)
GNAT2 (G protein subunit alpha transducin 2)
RBM4 (RNA binding motif protein 4)
GJB1 (gap junction protein beta 1)
BLMH (bleomycin hydrolase)
C4A (complement C4A (Rodgers blood group))
GSN (gelsolin)
HOOK2 (hook microtubule tethering protein 2)
GUCY2D (guanylate cyclase 2D, retinal)
CASP6 (caspase 6)
NMI (N-myc and STAT interactor)
AKR1B1 (aldo-keto reductase family 1 member B)
HPGD (15-hydroxyprostaglandin dehydrogenase)
HTR4 (5-hydroxytryptamine receptor 4)
IGFBP5 (insulin like growth factor binding protein 5)
PDLIM5 (PDZ and LIM domain 5)
IL13 (interleukin 13)
ITPKA (inositol-trisphosphate 3-kinase A)
MAPRE2 (microtubule associated protein RP/EB family member 2)
ITPR3 (inositol 1,4,5-trisphosphate receptor type 3)
JAG2 (jagged canonical Notch ligand 2)
KCNF1 (potassium voltage-gated channel modifier subfamily F member 1)
KCNG1 (potassium voltage-gated channel modifier subfamily G member 1)
CHEK1 (checkpoint kinase 1)
KCNJ1 (potassium inwardly rectifying channel subfamily J member 1)
KCNJ8 (potassium inwardly rectifying channel subfamily J member 8)
KCNN2 (potassium calcium-activated channel subfamily N member 2)
KCNN4 (potassium calcium-activated channel subfamily N member 4)
KCNQ3 (potassium voltage-gated channel subfamily Q member 3)
KCNS1 (potassium voltage-gated channel modifier subfamily S member 1)
KLK1 (kallikrein 1)
KNG1 (kininogen 1)
KIFC1 (kinesin family member C1)
KRAS (KRAS proto-oncogene, GTPase)
KRT10 (keratin 10)
KRT14 (keratin 14)
LAMB1 (laminin subunit beta 1)
RPSA (ribosomal protein SA)
LCN1 (lipocalin 1)
LEPR (leptin receptor)
LGALS3 (galectin 3)
CLCN4 (chloride voltage-gated channel 4)
LIPC (lipase C, hepatic type)
COX7A1 (cytochrome c oxidase subunit 7A1)
MAN2B1 (mannosidase alpha class 2B member 1)
MB (myoglobin)
MBD1 (methyl-CpG binding domain protein 1)
CSNK2A1 (casein kinase 2 alpha 1)
CYP2C19 (cytochrome P450 family 2 subfamily C member 19)
DGKG (diacylglycerol kinase gamma)
NFIX (nuclear factor I X)

LIG4 (DNA ligase 4)
NOTCH4 (notch receptor 4)
ARSD (arylsulfatase D)
PAX4 (paired box 4)
ETFA (electron transfer flavoprotein subunit alpha)
PKNOX1 (PBX/knotted 1 homeobox 1)
POLE2 (DNA polymerase epsilon 2, accessory subunit)
FOXO3 (forkhead box O3)
RAP1A (RAP1A, member of RAS oncogene family)
RASA2 (RAS p21 protein activator 2)
RPS20 (ribosomal protein S20)
TPGS2 (tubulin polyglutamylase complex subunit 2)
RTN1 (reticulon 1)
SCNN1G (sodium channel epithelial 1 subunit gamma)
GJA8 (gap junction protein alpha 8)
SRY (sex determining region Y)
ACP3 (acid phosphatase 3)
GLB1 (galactosidase beta 1)
KRIT1 (KRIT1 ankyrin repeat containing)
CSF2RA (colony stimulating factor 2 receptor subunit alpha)
SCGB2A1 (secretoglobin family 2A member 1)
MMP16 (matrix metallopeptidase 16)
