# Supplementary material for: Multi-omic characterization of pediatric ARDS via nasal brushings
Source: Respir Res. 2022 Jul 9;23:181. doi: 10.1186/s12931-022-02098-3 (PMC9270778; doi:10.1186/s12931-022-02098-3)
Supplement: Supplementary file 14 — Additional file 14: Table S3. Differentially Methylated Transcription Start Sites Subgroup 1 vs Subgroup 2. [file 12931_2022_2098_MOESM14_ESM.pdf]

Supplemental Table 3: Principal Component Genes

PC1

LINC02166 (long intergenic non-protein coding RNA 2166)
GLT8D1 (glycosyltransferase 8 domain containing 1)
UMODL1-AS1 (UMODL1 antisense RNA 1)
ELN-AS1 (ELN antisense RNA 1)
ZMYND10-AS1 (ZMYND10 antisense RNA 1)
SIAE (sialic acid acetylesterase)
DNAAF3-AS1 (DNAAF3 antisense RNA 1)
DNAH12 (dynein axonemal heavy chain 12)
MAP1A (microtubule associated protein 1A)
ATPAF2 (ATP synthase mitochondrial F1 complex assembly factor 2)
WDR86-AS1 (WDR86 antisense RNA 1)
LINC01513 (long intergenic non-protein coding RNA 1513)
MARCHF10 (membrane associated ring-CH-type finger 10)
IL5RA (interleukin 5 receptor subunit alpha)
ENKUR (enkurin, TRPC channel interacting protein)
LINC00683 (long intergenic non-protein coding RNA 683)
DNAAF1 (dynein axonemal assembly factor 1)
C5AR1 (complement C5a receptor 1)
CCDC33 (coiled-coil domain containing 33)
OMG (oligodendrocyte myelin glycoprotein)
LINC01765 (long intergenic non-protein coding RNA 1765)
MED15P9 (mediator complex subunit 15 pseudogene 9)
AQP4-AS1 (AQP4 antisense RNA 1)
EFCAB10 (EF-hand calcium binding domain 10)
DTHD1 (death domain containing 1)
RNF40 (ring finger protein 40)
C22orf15 (chromosome 22 open reading frame 15)
CASC2 (cancer susceptibility 2)
ANKRD44-AS1 (ANKRD44 antisense RNA 1)
DNAH3 (dynein axonemal heavy chain 3)
ERCC1 (ERCC excision repair 1, endonuclease non-catalytic subunit)
HYDIN2 (HYDIN axonemal central pair apparatus protein 2 (pseudogene))
CCDC187 (coiled-coil domain containing 187)
MUC12 (mucin 12, cell surface associated)
LINC02345 (long intergenic non-protein coding RNA 2345)

PC2

MARS1 (methionyl-tRNA synthetase 1)
NCF4-AS1 (NCF4 antisense RNA 1)
SOCS3 (suppressor of cytokine signaling 3)
METTL9 (methyltransferase like 9)
IRAK3 (interleukin 1 receptor associated kinase 3)
NCF1C (neutrophil cytosolic factor 1C pseudogene)
RHOH (ras homolog family member H)
CXCR2 (C-X-C motif chemokine receptor 2)
C5orf58 (chromosome 5 open reading frame 58)
CCR1 (C-C motif chemokine receptor 1)
MNDA (myeloid cell nuclear differentiation antigen)
IFI30 (IFI30 lysosomal thiol reductase)
CSF3R (colony stimulating factor 3 receptor)
NAMPT (nicotinamide phosphoribosyltransferase)
FAM177B (family with sequence similarity 177 member B)
TYMP (thymidine phosphorylase)
WAKMAR2 (wound and keratinocyte migration associated lncRNA 2)
FNIP1 (folliculin interacting protein 1)
PARP10 (poly(ADP-ribose) polymerase family member 10)
SORL1 (sortilin related receptor 1)
SIGLEC10-AS1 (SIGLEC10 antisense RNA 1)
MAP3K20-AS1 (MAP3K20 antisense RNA 1)
BTBD19 (BTB domain containing 19)
LINC00528 (long intergenic non-protein coding RNA 528)
PPP1R15A (protein phosphatase 1 regulatory subunit 15A)
SOD2 (superoxide dismutase 2)
CCR5AS (CCR5 antisense RNA)
IFITM3P1 (IFITM3 pseudogene 1)
NF1 (neurofibromin 1)
CD69 (CD69 molecule)
ALDH1A2 (aldehyde dehydrogenase 1 family member A2)
GNA15-DT (GNA15 divergent transcript)
IGF2R (insulin like growth factor 2 receptor)
PTK2B (protein tyrosine kinase 2 beta)
MX2 (MX dynamin like GTPase 2)
VPS9D1-AS1 (VPS9D1 antisense RNA 1)
FCGR1CP (Fc fragment of IgG receptor Ic, pseudogene)
PLA2G6 (phospholipase A2 group VI)
LINC01271 (long intergenic non-protein coding RNA 1271)
GBP5 (guanylate binding protein 5)
DENND5A (DENN domain containing 5A)
CYREN (cell cycle regulator of NHEJ)
MGAM (maltase-glucoamylase)
STXBP2 (syntaxin binding protein 2)
TNF (tumor necrosis factor)
PPCDC (phosphopantothenoylcysteine decarboxylase)
