# Supplementary material for: Multi-omic characterization of pediatric ARDS via nasal brushings
Source: Respir Res. 2022 Jul 9;23:181. doi: 10.1186/s12931-022-02098-3 (PMC9270778; doi:10.1186/s12931-022-02098-3)
Supplement: Supplementary file 15 — Additional file 15: Table S4. Principal Component Genes. [file 12931_2022_2098_MOESM15_ESM.pdf]

Supplemental Table 4: Principal Component GO Molecular Function

PC1

ATP-dependent microtubule motor activity, minus-end-
dynein light intermediate chain binding
dynein intermediate chain binding
ATP-dependent microtubule motor activity
microtubule motor activity
motor activity
sialate O-acetylesterase activity
sialate 9-O-acetylesterase activity
sialate 4-O-acetylesterase activity
complement component C5a binding
complement component C5a receptor activity
interleukin-5 binding
interleukin-5 receptor activity
3' overhang single-stranded DNA endodeoxyribonuclease
acetylesterase activity
opsonin receptor activity
short-chain carboxylesterase activity

PC2

pentosyltransferase activity
cytokine receptor activity
C-C chemokine receptor activity
C-C chemokine binding
G protein-coupled chemoattractant receptor activity
chemokine receptor activity
nicotinamide phosphoribosyltransferase activity
phosphopantothenoylcysteine decarboxylase activity
glucan 1,4-alpha-glucosidase activity
pyrimidine-nucleoside phosphorylase activity
immune receptor activity
chemokine binding
GTPase activity
retinoid binding
isoprenoid binding
chemokine (C-C motif) ligand 7 binding
methionine-tRNA ligase activity
thymidine phosphorylase activity
interleukin-8 receptor activity
granulocyte colony-stimulating factor binding
cytokine binding
maltose alpha-glucosidase activity
nicotinate-nucleotide diphosphorylase (carboxylating) activity
interleukin-8 binding
3-phosphoinositide-dependent protein kinase binding
syntaxin-3 binding
insulin-like growth factor-activated receptor activity
