# Supplementary material for: Multi-omic characterization of pediatric ARDS via nasal brushings
Source: Respir Res. 2022 Jul 9;23:181. doi: 10.1186/s12931-022-02098-3 (PMC9270778; doi:10.1186/s12931-022-02098-3)
Supplement: Supplementary file 16 — Additional file 16: Table S5. Principal Component GO Molecular Function. [file 12931_2022_2098_MOESM16_ESM.pdf]

Supplemental Table 5: Differentially Expressed Genes

Subgroup A

Down

- TTLL10-AS1 (TTLL10 antisense RNA 1)
- TTLL10 (tubulin tyrosine ligase like 10)
- INTS11 (integrator complex subunit 11)
- CPTP (ceramide-1-phosphate transfer protein)
- MORN1 (MORN repeat containing 1)
- PLCH2 (phospholipase C eta 2)
- TNFRSF14-AS1 (TNFRSF14 antisense RNA 1)
- TTC34 (tetratricopeptide repeat domain 34)
- LINC01134 (long intergenic non-protein coding RNA 1134)
- VAMP3 (vesicle associated membrane protein 3)
- RERE (arginine-glutamic acid dipeptide repeats)
- MIR34AHG (MIR34A host gene)
- EXOSC10 (exosome component 10)
- FHAD1-AS1 (FHAD1 antisense RNA 1)
- NBPF1 (NBPF member 1)
- EMC1-AS1 (EMC1 antisense RNA 1)
- CDC42-AS1 (CDC42 antisense RNA 1)
- KDM1A (lysine demethylase 1A)
- SRSF10 (serine and arginine rich splicing factor 10)
- NCMAP-DT (NCMAP divergent transcript)
- RSRP1 (arginine and serine rich protein 1)
- TMEM50A (transmembrane protein 50A)
- HMGN2 (high mobility group nucleosomal binding domain
- NUDC (nuclear distribution C, dynein complex regulator)
- COL16A1 (collagen type XVI alpha 1 chain)
- EIF3I (eukaryotic translation initiation factor 3 subunit I)
- ZBTB8OS (zinc finger and BTB domain containing 8 opposite
- KIAA0319L (KIAA0319 like)
- TEKT2 (tektin 2)
- CSF3R (colony stimulating factor 3 receptor)
- GNL2 (G protein nucleolar 2)
- EBNA1BP2 (EBNA1 binding protein 2)
- KDM4A-AS1 (KDM4A antisense RNA 1)
- DMAP1 (DNA methyltransferase 1 associated protein 1)
- BTBD19 (BTB domain containing 19)
- NASP (nuclear autoantigenic sperm protein)
- NSUN4 (NOP2/Sun RNA methyltransferase 4)
- ATPAF1 (ATP synthase mitochondrial F1 complex assembly
- CDKN2C (cyclin dependent kinase inhibitor 2C)
- TTC39A-AS1 (TTC39A antisense RNA 1)
- HSPB11 (heat shock protein family B (small) member 11)
- SPATA1 (spermatogenesis associated 1)
- CCN1 (cellular communication network factor 1)
- ODF2L (outer dense fiber of sperm tails 2 like)
- GBP5 (guanylate binding protein 5)
- DR1 (down-regulator of transcription 1)
- F3 (coagulation factor III, tissue factor)
- SLC35A3 (solute carrier family 35 member A3)
- EXTL2 (exostosin like glycosyltransferase 2)
- STXBP3 (syntaxin binding protein 3)
- TMEM167B-DT (TMEM167B divergent transcript)
- SARS1 (seryl-tRNA synthetase 1)
- CEPT1 (choline/ethanolamine phosphotransferase 1)
- DENND2D (DENN domain containing 2D)
- ATP5PB (ATP synthase peripheral stalk-membrane subunit
- TRIM33 (tripartite motif containing 33)
- NRAS (NRAS proto-oncogene, GTPase)
- LINC01765 (long intergenic non-protein coding RNA 1765)
- LINC00623 (long intergenic non-protein coding RNA 623)
- NBPF10 (NBPF member 10)
- ADAMTSL4-AS2 (ADAMTSL4 antisense RNA 2)
- CERS2 (ceramide synthase 2)
- MLLT11 (MLLT11 transcription factor 7 cofactor)
- C2CD4D-AS1 (C2CD4D and THEM5 antisense RNA 1)
- S100A11 (S100 calcium binding protein A11)
- CHTOP (chromatin target of PRMT1)
- ADAR (adenosine deaminase RNA specific)
- DCST1-AS1 (DCST1 antisense RNA 1)
- MNDA (myeloid cell nuclear differentiation antigen)
- LINC01133 (long intergenic non-protein coding RNA 1133)
- VAMP4 (vesicle associated membrane protein 4)
- PRDX6 (peroxiredoxin 6)
- MRPS14 (mitochondrial ribosomal protein S14)
- GLUL (glutamate-ammonia ligase)
- RO60 (Ro60, Y RNA binding protein)
- ELF3-AS1 (ELF3 antisense RNA 1)
- BLACAT1 (bladder cancer associated transcript 1)
- RAB29 (RAB29, member RAS oncogene family)
- LINC00467 (long intergenic non-protein coding RNA 467)
- ESRRG (estrogen related receptor gamma)
- SPATA17-AS1 (SPATA17 antisense RNA 1)
- BROX (BRO1 domain and CAAX motif containing)
- FAM177B (family with sequence similarity 177 member B)
- CAPN2 (calpain 2)
- TMEM63A (transmembrane protein 63A)
- ACBD3-AS1 (ACBD3 antisense RNA 1)
- TRIM11 (tripartite motif containing 11)
- AGT (angiotensinogen)
- COA6-AS1 (COA6 antisense RNA 1)
- TARBP1 (TAR (HIV-1) RNA binding protein 1)
- LINC01132 (long intergenic non-protein coding RNA 1132)
- EDARADD (EDAR associated death domain)
- SDCCAG8 (SHH signaling and ciliogenesis regulator
- COX20 (cytochrome c oxidase assembly factor COX20)
- CALML3-AS1 (CALML3 antisense RNA 1)
- CDC123 (cell division cycle 123)
- RNU6-6P (RNA, U6 small nuclear 6, pseudogene)
- BEND7 (BEN domain containing 7)
- SUV39H2-DT (SUV39H2 divergent transcript)
- RPP38-DT (RPP38 divergent transcript)
- NMT2 (N-myristoyltransferase 2)
- VIM-AS1 (VIM antisense RNA 1)
- ENKUR (enkurin, TRPC channel interacting protein)
- KIF5B (kinesin family member 5B)
- EPC1 (enhancer of polycomb homolog 1)
- ZNF248 (zinc finger protein 248)
- ZNF33A (zinc finger protein 33A)
- ZNF33B (zinc finger protein 33B)
- ZNF32-AS1 (ZNF32 antisense RNA 1)
- RASSF4 (Ras association domain family member 4)
- ZNF22-AS1 (ZNF22 antisense RNA 1)
- TIMM23B (translocase of inner mitochondrial membrane 23
- SGMS1-AS1 (SGMS1 antisense RNA 1)
- JMJD1C (jumonji domain containing 1C)
- REEP3 (receptor accessory protein 3)
- DNAJC9-AS1 (DNAJC9 antisense RNA 1)
- CFAP70 (cilia and flagella associated protein 70)
- PPP3CB (protein phosphatase 3 catalytic subunit beta)
- NUTM2B-AS1 (NUTM2B antisense RNA 1)
- ANXA11 (annexin A11)
- IFIT3 (interferon induced protein with tetratricopeptide
- PDLIM1 (PDZ and LIM domain 1)
- DNMBP (dynamin binding protein)
- DNMBP-AS1 (DNMBP antisense RNA 1)
- C10orf95 (chromosome 10 open reading frame 95)
- CFAP58-DT (CFAP58 divergent transcript)
- PDCD4-AS1 (PDCD4 antisense RNA 1)
- ZDHHC6 (zinc finger DHHC-type palmitoyltransferase 6)
- VTI1A (vesicle transport through interaction with t-SNAREs
- CASP7 (caspase 7)
- PLEKHS1 (pleckstrin homology domain containing S1)
- HSPA12A (heat shock protein family A (Hsp70) member
- WDR11 (WD repeat domain 11)
- MUC5B-AS1 (MUC5B antisense RNA 1)
- NAP1L4 (nucleosome assembly protein 1 like 4)
- TRIM22 (tripartite motif containing 22)
- WEE1 (WEE1 G2 checkpoint kinase)
- TEAD1 (TEA domain transcription factor 1)
- GTF2H1 (general transcription factor IIH subunit 1)
- ANO3 (anoctamin 3)
- TCP11L1 (t-complex 11 like 1)
- LINC00294 (long intergenic non-protein coding RNA 294)
- CD59 (CD59 molecule (CD59 blood group))
- FBXO3 (F-box protein 3)
- CD44 (CD44 molecule (Indian blood group))
- CD44-AS1 (CD44 antisense RNA 1)
- PRDM11 (PR/SET domain 11)
- PHF21A (PHD finger protein 21A)
- DGKZ (diacylglycerol kinase zeta)
- FNBP4 (formin binding protein 4)
- CD6 (CD6 molecule)
- CYB561A3 (cytochrome b561 family member A3)
- TMEM258 (transmembrane protein 258)
- FADS1 (fatty acid desaturase 1)
- GPR137 (G protein-coupled receptor 137)
- ESRRA (estrogen related receptor alpha)
- MAP3K11 (mitogen-activated protein kinase kinase kinase
- RBM4B (RNA binding motif protein 4B)
- ANKRD13D (ankyrin repeat domain 13D)
- RAD9A (RAD9 checkpoint clamp component A)
- ALDH3B1 (aldehyde dehydrogenase 3 family member B1)
- TCIRG1 (T cell immune regulator 1, ATPase H+ transporting
- ARAP1-AS1 (ARAP1 antisense RNA 1)
- ATG16L2 (autophagy related 16 like 2)
- PCF11 (PCF11 cleavage and polyadenylation factor subunit)
- PCF11-AS1 (PCF11 antisense RNA 1)
- PRSS23 (serine protease 23)
- ARHGAP42-AS1 (ARHGAP42 antisense RNA 1)
- CASP4 (caspase 4)
- EXPH5 (exophilin 5)
- COLCA1 (colorectal cancer associated 1)
- IL18 (interleukin 18)
- PAFAH1B2 (platelet activating factor acetylhydrolase 1b
- TMPRSS4 (transmembrane serine protease 4)
- CENATAC-DT (CENATAC divergent transcript)
- TRIM29 (tripartite motif containing 29)
- SORL1 (sortilin related receptor 1)
- SIAE (sialic acid acetylesterase)

- GSEC (G-quadruplex forming sequence containing lncRNA)

- CRACR2A (calcium release activated channel regulator 2A)
- AKAP3 (A-kinase anchoring protein 3)
- MIR200CHG (MIR200C and MIR141 host gene)
- CD69 (CD69 molecule)
- YBX3 (Y-box binding protein 3)
- CDKN1B (cyclin dependent kinase inhibitor 1B)
- PLBD1-AS1 (PLBD1 antisense RNA 1)
- H2AJ (H2A.J histone)
- ETFRF1 (electron transfer flavoprotein regulatory factor 1)
- FGFR1OP2 (FGFR1 oncogene partner 2)
- PTHLH (parathyroid hormone like hormone)
- FAR2 (fatty acyl-CoA reductase 2)
- LINC02422 (long intergenic non-protein coding RNA 2422)
- PKP2 (plakophilin 2)
- IRAK4 (interleukin 1 receptor associated kinase 4)
- SCAF11 (SR-related CTD associated factor 11)
- HDAC7 (histone deacetylase 7)
- FKBP11 (FKBP prolyl isomerase 11)
- TUBA1C (tubulin alpha 1c)
- NR4A1AS (NR4A1 antisense RNA)
- KRT8 (keratin 8)
- PCBP2 (poly(rC) binding protein 2)
- CBX5 (chromobox 5)
- MYL6 (myosin light chain 6)
- PTGES3 (prostaglandin E synthase 3)

Up

- CDSN (corneodesmosin)

Subgroup B

Down

- TTLL10-AS1 (TTLL10 antisense RNA 1)
- TTLL10 (tubulin tyrosine ligase like 10)
- MORN1 (MORN repeat containing 1)
- TTC34 (tetratricopeptide repeat domain 34)
- RERE (arginine-glutamic acid dipeptide repeats)
- EXOSC10 (exosome component 10)
- FHAD1-AS1 (FHAD1 antisense RNA 1)
- HMGN2 (high mobility group nucleosomal binding domain
- COL16A1 (collagen type XVI alpha 1 chain)
- TEKT2 (tektin 2)
- EBNA1BP2 (EBNA1 binding protein 2)
- LINC01144 (long intergenic non-protein coding RNA 1144)
- TTC39A-AS1 (TTC39A antisense RNA 1)
- SLC35A3 (solute carrier family 35 member A3)
- LINC01765 (long intergenic non-protein coding RNA 1765)
- C2CD4D-AS1 (C2CD4D and THEM5 antisense RNA 1)
- LINC00467 (long intergenic non-protein coding RNA 467)
- SPATA17-AS1 (SPATA17 antisense RNA 1)
- ACBD3-AS1 (ACBD3 antisense RNA 1)
- LINC01132 (long intergenic non-protein coding RNA 1132)
- EDARADD (EDAR associated death domain)
- BEND7 (BEN domain containing 7)
- ENKUR (enkurin, TRPC channel interacting protein)
- CFAP70 (cilia and flagella associated protein 70)
- PLEKHS1 (pleckstrin homology domain containing S1)
- HSPA12A (heat shock protein family A (Hsp70) member
- CASC2 (cancer susceptibility 2)
- MUC5B-AS1 (MUC5B antisense RNA 1)
- STIM1-AS1 (STIM1 antisense RNA 1)
- NUCB2 (nucleobindin 2)
- CSRP3-AS1 (CSRP3 and E2F8 antisense RNA 1)
- CD6 (CD6 molecule)
- RAD9A (RAD9 checkpoint clamp component A)
- PRSS23 (serine protease 23)
- EXPH5 (exophilin 5)
- COLCA1 (colorectal cancer associated 1)
- SIAE (sialic acid acetylesterase)
- CRACR2A (calcium release activated channel regulator 2A)
- AKAP3 (A-kinase anchoring protein 3)
- TUBA1C (tubulin alpha 1c)
- KRT8 (keratin 8)
- LRRIQ1 (leucine rich repeats and IQ motif containing 1)
- IQCD (IQ motif containing D)
- NRAV (negative regulator of antiviral response)
- CFAP251 (cilia and flagella associated protein 251)
- DNAH10 (dynein axonemal heavy chain 10)
- DNAH10OS (dynein axonemal heavy chain 10 opposite
- LINC00332 (long intergenic non-protein coding RNA 332)
- VWA8-AS1 (VWA8 antisense RNA 1 (head to head))
- SUCLA2-AS1 (SUCLA2 antisense RNA 1)
- NEK5 (NIMA related kinase 5)
- FGF14-AS2 (FGF14 antisense RNA 2)
- ZFHX2 (zinc finger homeobox 2)
- SLC25A21-AS1 (SLC25A21 antisense RNA 1)
- SALRNA1 (senescence associated long non-coding RNA 1)
- PTGR2 (prostaglandin reductase 2)
- FOXN3-AS1 (FOXN3 antisense RNA 1)
- PAPOLA-DT (PAPOLA divergent transcript)
- CLBA1 (clathrin binding box of aftiphilin containing 1)
- NDN (necdin, MAGE family member)
- LINC02345 (long intergenic non-protein coding RNA 2345)
- MAP1A (microtubule associated protein 1A)
- TEX9 (testis expressed 9)
- MYO1E (myosin IE)
- CCDC33 (coiled-coil domain containing 33)
- WDR93 (WD repeat domain 93)
- ABAT (4-aminobutyrate aminotransferase)
- LINC02185 (long intergenic non-protein coding RNA 2185)
- DNAH3 (dynein axonemal heavy chain 3)
- RNF40 (ring finger protein 40)
- BCL7C (BAF chromatin remodeling complex subunit BCL7C)
- FTO (FTO alpha-ketoglutarate dependent dioxygenase)
- DNAAF1 (dynein axonemal assembly factor 1)
- CIBAR2 (CBY1 interacting BAR domain containing 2)
- LINC02166 (long intergenic non-protein coding RNA 2166)
- NAA38 (N-alpha-acetyltransferase 38, NatC auxiliary
- DNAH9 (dynein axonemal heavy chain 9)
- DRC3 (dynein regulatory complex subunit 3)
- ATPAF2 (ATP synthase mitochondrial F1 complex assembly
- B9D1 (B9 domain containing 1)
- ERVE-1 (endogenous retrovirus group E member 1)
- OMG (oligodendrocyte myelin glycoprotein)
- TADA2A (transcriptional adaptor 2A)
- ANKFN1 (ankyrin repeat and fibronectin type III domain
- AKAP1-DT (AKAP1 divergent transcript)
- LINC02875 (long intergenic non-protein coding RNA 2875)
- MARCHF10 (membrane associated ring-CH-type finger 10)
- PRR29-AS1 (PRR29 antisense RNA 1)
- ICAM2 (intercellular adhesion molecule 2)
- BAIAP2-DT (BAIAP2 divergent transcript)
- BAIAP2 (BAR/IMD domain containing adaptor protein 2)
- MAFG-DT (MAFG divergent transcript)
- TTC39C-AS1 (TTC39C antisense RNA 1)
- KCTD1 (potassium channel tetramerization domain
- AQP4-AS1 (AQP4 antisense RNA 1)
- LINC01908 (long intergenic non-protein coding RNA 1908)
- ELAC1 (elaC ribonuclease Z 1)
- TXNL1 (thioredoxin like 1)
- NEDD4L (NEDD4 like E3 ubiquitin protein ligase)
- KDSR-DT (KDSR divergent transcript)
- LINC00683 (long intergenic non-protein coding RNA 683)
- PARD6G-AS1 (PARD6G antisense RNA 1)
- C3 (complement C3)
- PRKCSH (protein kinase C substrate 80K-H)
- ZNF440 (zinc finger protein 440)
- NFIX (nuclear factor I X)
- NWD1 (NACHT and WD repeat domain containing 1)
- DPY19L3-DT (DPY19L3 divergent transcript)
- CEP89 (centrosomal protein 89)
- CYP2F1 (cytochrome P450 family 2 subfamily F member 1)
- ERCC1 (ERCC excision repair 1, endonuclease non-catalytic
- C5AR1 (complement C5a receptor 1)
- ZNF473 (zinc finger protein 473)
- ZNF677 (zinc finger protein 677)
- DNAAF3-AS1 (DNAAF3 antisense RNA 1)
- FAM166C (family with sequence similarity 166 member C)
- LINC01833 (long intergenic non-protein coding RNA 1833)
- SIX3-AS1 (SIX3 antisense RNA 1)
- CALM2 (calmodulin 2)
- NEURL3 (neuralized E3 ubiquitin protein ligase 3)
- CRACDL (CRACD like)
- TSGA10 (testis specific 10)
- TBC1D8-AS1 (TBC1D8 antisense RNA 1)
- CCDC138 (coiled-coil domain containing 138)
- NPHP1 (nephrocystin 1)
- TTC21B-AS1 (TTC21B antisense RNA 1)
- AGPS (alkylglycerone phosphate synthase)
- ANKRD44-AS1 (ANKRD44 antisense RNA 1)
- IGFBP2 (insulin like growth factor binding protein 2)
- NORAD (non-coding RNA activated by DNA damage)
- VAPB (VAMP associated protein B and C)
- UCKL1-AS1 (UCKL1 antisense RNA 1)
- LINC01436 (long intergenic non-protein coding RNA 1436)
- UMODL1-AS1 (UMODL1 antisense RNA 1)
- NDUFV3 (NADH:ubiquinone oxidoreductase subunit V3)
- TFIP11-DT (TFIP11 divergent transcript)
- RNF215 (ring finger protein 215)
- EP300-AS1 (EP300 antisense RNA 1)
- SYN2 (synapsin II)
- KIF9-AS1 (KIF9 antisense RNA 1)
- ZMYND10-AS1 (ZMYND10 antisense RNA 1)
- GLT8D1 (glycosyltransferase 8 domain containing 1)
- DNAH12 (dynein axonemal heavy chain 12)
- FAM107A (family with sequence similarity 107 member A)
- DCBLD2 (discoidin, CUB and LCCL domain containing 2)
- CD200R1L-AS1 (CD200R1L antisense RNA 1)
- QTRT2 (queuine tRNA-ribosyltransferase accessory subunit
- SNORA7B (small nucleolar RNA, H/ACA box 7B)
- EFCAB12 (EF-hand calcium binding domain 12)
- ANKUB1 (ankyrin repeat and ubiquitin domain containing
- TMEM212 (transmembrane protein 212)
- LINC02031 (long intergenic non-protein coding RNA 2031)
- ABCC5 (ATP binding cassette subfamily C member 5)
- PCYT1A (phosphate cytidylyltransferase 1A, choline)
- SPON2 (spondin 2)
- TADA2B (transcriptional adaptor 2B)
- PCDH7 (protocadherin 7)
- DTHD1 (death domain containing 1)
- UGDH (UDP-glucose 6-dehydrogenase)
- UGDH-AS1 (UGDH antisense RNA 1)
- LINC02265 (long intergenic non-protein coding RNA 2265)
- SGCB (sarcoglycan beta)
- SULT1E1 (sulfotransferase family 1E member 1)
- BMPR1B-DT (BMPR1B divergent transcript)
- CXXC4 (CXXC finger protein 4)
- SETD7 (SET domain containing 7, histone lysine
- TPPP (tubulin polymerization promoting protein)
- SEMA5A (semaphorin 5A)
- LINC01513 (long intergenic non-protein coding RNA 1513)
- TTC23L-AS1 (TTC23L antisense RNA 1)
- LIFR (LIF receptor subunit alpha)
- BTF3-DT (BTF3 divergent transcript)
- LINC01843 (long intergenic non-protein coding RNA 1843)
- IK (IK cytokine)
- FABP6-AS1 (FABP6 antisense RNA 1)
- HCG14 (HLA complex group 14)
- SAPCD1-AS1 (SAPCD1 antisense RNA 1)
- KIF6 (kinesin family member 6)

- ANKRD66 (ankyrin repeat domain 66)

- LRP11 (LDL receptor related protein 11)
- RAET1E-AS1 (RAET1E antisense RNA 1)
- RSPH3 (radial spoke head 3)
- AGR2 (anterior gradient 2, protein disulphide isomerase
- ZNF273 (zinc finger protein 273)
- ELN-AS1 (ELN antisense RNA 1)
- DDX3ILA1 (DDX3 interacting lncRNA 1)
- PCOLCE-AS1 (PCOLCE antisense RNA 1)
- MUC12 (mucin 12, cell surface associated)
- EMSLR (E2F1 mRNA stabilizing lncRNA)
- EFCAB10 (EF-hand calcium binding domain 10)
- LRGUK (leucine rich repeats and guanylate kinase domain
- AGBL3 (AGBL carboxypeptidase 3)
- WDR86-AS1 (WDR86 antisense RNA 1)
- NAT1 (N-acetyltransferase 1)
- LINC00589 (long intergenic non-protein coding RNA 589)
- GSR (glutathione-disulfide reductase)
- RP1 (RP1 axonemal microtubule associated)
- IL7 (interleukin 7)
- TMEM67 (transmembrane protein 67)
- NDUFB9 (NADH:ubiquinone oxidoreductase subunit B9)
- RHPN1-AS1 (RHPN1 antisense RNA 1 (head to head))
- IQANK1 (IQ motif and ankyrin repeat containing 1)
- GLIS3-AS1 (GLIS3 antisense RNA 1)
- NTRK2 (neurotrophic receptor tyrosine kinase 2)

Up

- SPATA21 (spermatogenesis associated 21)
- NECAP2 (NECAP endocytosis associated 2)
- EMC1-AS1 (EMC1 antisense RNA 1)
- STPG1 (sperm tail PG-rich repeat containing 1)
- CD52 (CD52 molecule)
- LAPTM5 (lysosomal protein transmembrane 5)
- KIAA0319L (KIAA0319 like)
- CSF3R (colony stimulating factor 3 receptor)
- BTBD19 (BTB domain containing 19)
- MKNK1-AS1 (MKNK1 antisense RNA 1)
- SPATA1 (spermatogenesis associated 1)
- BCL10 (BCL10 immune signaling adaptor)
- CCN1 (cellular communication network factor 1)
- GBP5 (guanylate binding protein 5)
- CCDC18-AS1 (CCDC18 antisense RNA 1)
- TMEM167B-DT (TMEM167B divergent transcript)
- GNAT2 (G protein subunit alpha transducin 2)
- DENND2D (DENN domain containing 2D)
- TRIM33 (tripartite motif containing 33)
- CD2 (CD2 molecule)
- LINC00623 (long intergenic non-protein coding RNA 623)
- ADAMTSL4-AS2 (ADAMTSL4 antisense RNA 2)
- ADAMTSL4-AS1 (ADAMTSL4 antisense RNA 1)
- MLLT11 (MLLT11 transcription factor 7 cofactor)
- ADAR (adenosine deaminase RNA specific)
- MTX1 (metaxin 1)
- FDPS (farnesyl diphosphate synthase)
- ARHGEF2 (Rho/Rac guanine nucleotide exchange factor 2)
- MNDA (myeloid cell nuclear differentiation antigen)
- NIT1 (nitrilase 1)
- NR1I3 (nuclear receptor subfamily 1 group I member 3)
- TOR1AIP2 (torsin 1A interacting protein 2)
- GLUL (glutamate-ammonia ligase)
- IVNS1ABP (influenza virus NS1A binding protein)
- RAB29 (RAB29, member RAS oncogene family)
- IL19 (interleukin 19)
- CD55 (CD55 molecule (Cromer blood group))
- FAM177B (family with sequence similarity 177 member B)
- TMEM63A (transmembrane protein 63A)
- TSNAX (translin associated factor X)
- COX20 (cytochrome c oxidase assembly factor COX20)
- LINC00707 (long intergenic non-protein coding RNA 707)
- USP6NL-AS1 (USP6NL antisense RNA 1)
- CDC123 (cell division cycle 123)
- RNU6-6P (RNA, U6 small nuclear 6, pseudogene)
- VIM-AS1 (VIM antisense RNA 1)
- MAP3K8 (mitogen-activated protein kinase kinase kinase 8)
- EPC1 (enhancer of polycomb homolog 1)
- PRKG1-AS1 (PRKG1 antisense RNA 1)
- JMJD1C (jumonji domain containing 1C)
- SHLD2 (shieldin complex subunit 2)
- IFIT3 (interferon induced protein with tetratricopeptide
- HPS1 (HPS1 biogenesis of lysosomal organelles complex 3
- PSD (pleckstrin and Sec7 domain containing)
- SH3PXD2A-AS1 (SH3PXD2A antisense RNA 1)
- CFAP58-DT (CFAP58 divergent transcript)
- PDCD4-AS1 (PDCD4 antisense RNA 1)
- ZDHHC6 (zinc finger DHHC-type palmitoyltransferase 6)
- TMEM80 (transmembrane protein 80)
- TOLLIP (toll interacting protein)
- PRR33 (proline rich 33)
- RHOG (ras homolog family member G)
- TRIM22 (tripartite motif containing 22)
- DENND5A (DENN domain containing 5A)
- MICAL2 (microtubule associated monooxygenase, calponin
- C11orf58 (chromosome 11 open reading frame 58)
- SAA2 (serum amyloid A2)
- CD44 (CD44 molecule (Indian blood group))
- CD44-AS1 (CD44 antisense RNA 1)
- DGKZ (diacylglycerol kinase zeta)
- TMEM258 (transmembrane protein 258)
- PPP1R14B (protein phosphatase 1 regulatory inhibitor
- NEAT1 (nuclear paraspeckle assembly transcript 1)
- MALAT1 (metastasis associated lung adenocarcinoma
- MAP3K11 (mitogen-activated protein kinase kinase kinase
- SNX32 (sorting nexin 32)
- TCIRG1 (T cell immune regulator 1, ATPase H+ transporting
- ANO1-AS1 (ANO1 antisense RNA 1)
- ARAP1-AS1 (ARAP1 antisense RNA 1)
- ATG16L2 (autophagy related 16 like 2)
- CASP4 (caspase 4)
- PCSK7 (proprotein convertase subtilisin/kexin type 7)
- SORL1 (sortilin related receptor 1)
- HSPA8 (heat shock protein family A (Hsp70) member 8)
- GSEC (G-quadruplex forming sequence containing lncRNA)
- CCDC77 (coiled-coil domain containing 77)
- CD69 (CD69 molecule)
- KLRK1-AS1 (KLRK1 antisense RNA 1)
- CDKN1B (cyclin dependent kinase inhibitor 1B)
- FGFR1OP2 (FGFR1 oncogene partner 2)
- LINC02422 (long intergenic non-protein coding RNA 2422)
- IRAK4 (interleukin 1 receptor associated kinase 4)
- SCAF11 (SR-related CTD associated factor 11)
- TUBA1B (tubulin alpha 1b)
- DAZAP2 (DAZ associated protein 2)
- NR4A1AS (NR4A1 antisense RNA)
- MYL6 (myosin light chain 6)
- STAT6 (signal transducer and activator of transcription 6)
- MARS1 (methionyl-tRNA synthetase 1)
- LINC02448 (long intergenic non-protein coding RNA 2448)
- USP15 (ubiquitin specific peptidase 15)
- IRAK3 (interleukin 1 receptor associated kinase 3)
- PRANCR (progenitor renewal associated non-coding RNA)
- THAP2 (THAP domain containing 2)
- PPP1R12A-AS1 (PPP1R12A antisense RNA 1)
- ATP2B1-AS1 (ATP2B1 antisense RNA 1)
- LTA4H (leukotriene A4 hydrolase)
- USP30-AS1 (USP30 antisense RNA 1)
- TCHP (trichoplein keratin filament binding)
- GIT2 (GIT ArfGAP 2)
- ATXN2 (ataxin 2)
- RFC5 (replication factor C subunit 5)
- PXN-AS1 (PXN antisense RNA 1)
- LINC02361 (long intergenic non-protein coding RNA 2361)
- TPT1-AS1 (TPT1 antisense RNA 1)
- CPB2-AS1 (CPB2 antisense RNA 1)
- SCEL-AS1 (SCEL antisense RNA 1)
- LINC00641 (long intergenic non-protein coding RNA 641)
- SUPT16H (SPT16 homolog, facilitates chromatin remodeling
- TRDC (T cell receptor delta constant)
- TRAC (T cell receptor alpha constant)
- FAM177A1 (family with sequence similarity 177 member
- LINC01588 (long intergenic non-protein coding RNA 1588)
- PSMA3 (proteasome 20S subunit alpha 3)
- PSMA3-AS1 (PSMA3 antisense RNA 1)
- HIF1A-AS1 (HIF1A antisense RNA 1)
- ZBTB25 (zinc finger and BTB domain containing 25)
- SIPA1L1 (signal induced proliferation associated 1 like 1)
- FOS (Fos proto-oncogene, AP-1 transcription factor subunit)
- GPATCH2L (G-patch domain containing 2 like)
- DGLUCY (D-glutamate cyclase)
- LINC00239 (long intergenic non-protein coding RNA 239)
- KATNBL1 (katanin regulatory subunit B1 like 1)
- DNAJC17 (DnaJ heat shock protein family (Hsp40) member
- B2M (beta-2-microglobulin)
- GABPB1-AS1 (GABPB1 antisense RNA 1)
- GCOM1 (GCOM1, MYZAP-POLR2M combined locus)
- ANXA2 (annexin A2)
- OAZ2 (ornithine decarboxylase antizyme 2)
- ADPGK (ADP dependent glucokinase)
- ADPGK-AS1 (ADPGK antisense RNA 1)
- NPTN-IT1 (NPTN intronic transcript 1)
- PPCDC (phosphopantothenoylcysteine decarboxylase)
- C15orf39 (chromosome 15 open reading frame 39)
- DNAJA4 (DnaJ heat shock protein family (Hsp40) member
- ST20-AS1 (ST20 antisense RNA 1)
- HDGFL3 (HDGF like 3)
- PRC1-AS1 (PRC1 antisense RNA 1)
- CHASERR (CHD2 adjacent suppressive regulatory RNA)
- MCTP2 (multiple C2 and transmembrane domain
- EME2 (essential meiotic structure-specific endonuclease
- LINC00921 (long intergenic non-protein coding RNA 921)
- SOCS1 (suppressor of cytokine signaling 1)
- METTL9 (methyltransferase like 9)
- IL4R (interleukin 4 receptor)
- STX4 (syntaxin 4)
- FUS (FUS RNA binding protein)
- ITGAX (integrin subunit alpha X)
- CYLD-AS1 (CYLD antisense RNA 1)
- KIFC3 (kinesin family member C3)
- ATP6V0D1 (ATPase H+ transporting V0 subunit d1)
- NFAT5 (nuclear factor of activated T cells 5)
- KARS1 (lysyl-tRNA synthetase 1)
- KLHL36 (kelch like family member 36)
- SPG7 (SPG7 matrix AAA peptidase subunit, paraplegin)
- VPS9D1-AS1 (VPS9D1 antisense RNA 1)
- ZNF276 (zinc finger protein 276)
- ENO3 (enolase 3)
- PRAL (p53 regulation associated lncRNA)
- ALOX12-AS1 (ALOX12 antisense RNA 1)
- FGF11 (fibroblast growth factor 11)
- LINC00324 (long intergenic non-protein coding RNA 324)
- NDEL1 (nudE neurodevelopment protein 1 like 1)
- PIK3R5-DT (PIK3R5 divergent transcript)
- TMEM238L (transmembrane protein 238 like)
- FLCN (folliculin)
- WSB1 (WD repeat and SOCS box containing 1)
- RSKR (ribosomal protein S6 kinase related)

- NF1 (neurofibromin 1)

- ZNF207 (zinc finger protein 207)
- CCL3-AS1 (CCL3 antisense RNA 1)
- ZNHIT3 (zinc finger HIT-type containing 3)
- RARA-AS1 (RARA antisense RNA 1)
- KRT13 (keratin 13)
- EIF1 (eukaryotic translation initiation factor 1)
- STAT3 (signal transducer and activator of transcription 3)
- MAP3K14-AS1 (MAP3K14 antisense RNA 1)
- TSPOAP1-AS1 (TSPOAP1, SUPT4H1 and RNF43 antisense RNA
- YPEL2 (yippee like 2)
- PTRH2 (peptidyl-tRNA hydrolase 2)
- VMP1 (vacuole membrane protein 1)
- SOCS3 (suppressor of cytokine signaling 3)
- PGS1 (phosphatidylglycerophosphate synthase 1)
- SGSH (N-sulfoglucosamine sulfohydrolase)
- RNF213 (ring finger protein 213)
- RNF213-AS1 (RNF213 antisense RNA 1)
- CSNK1D (casein kinase 1 delta)
- MYL12A (myosin light chain 12A)
- VAPA (VAMP associated protein A)
- ABHD3 (abhydrolase domain containing 3, phospholipase)
- NPC1 (NPC intracellular cholesterol transporter 1)
- PWWP3A (PWWP domain containing 3A, DNA repair factor)
- JSRP1 (junctional sarcoplasmic reticulum protein 1)
- GNA11 (G protein subunit alpha 11)

Subgroup C

Down

- CD52 (CD52 molecule)
- LAPTM5 (lysosomal protein transmembrane 5)
- CSF3R (colony stimulating factor 3 receptor)
- GBP5 (guanylate binding protein 5)
- CD2 (CD2 molecule)
- ADAMTSL4-AS2 (ADAMTSL4 antisense RNA 2)
- ADAMTSL4-AS1 (ADAMTSL4 antisense RNA 1)
- MNDA (myeloid cell nuclear differentiation antigen)
- IVNS1ABP (influenza virus NS1A binding protein)
- FAM177B (family with sequence similarity 177 member B)
- VIM-AS1 (VIM antisense RNA 1)
- CFAP58-DT (CFAP58 divergent transcript)
- PRR33 (proline rich 33)
- DENND5A (DENN domain containing 5A)
- CD44 (CD44 molecule (Indian blood group))
- CD44-AS1 (CD44 antisense RNA 1)
- NEAT1 (nuclear paraspeckle assembly transcript 1)
- CD69 (CD69 molecule)
- MARS1 (methionyl-tRNA synthetase 1)
- LINC02448 (long intergenic non-protein coding RNA 2448)
- USP15 (ubiquitin specific peptidase 15)
- IRAK3 (interleukin 1 receptor associated kinase 3)
- CPB2-AS1 (CPB2 antisense RNA 1)
- SCEL-AS1 (SCEL antisense RNA 1)
- FOS (Fos proto-oncogene, AP-1 transcription factor subunit)
- THBS1-AS1 (THBS1 antisense RNA 1)
- B2M (beta-2-microglobulin)
- DUOX2 (dual oxidase 2)
- GCOM1 (GCOM1, MYZAP-POLR2M combined locus)
- ALDH1A2 (aldehyde dehydrogenase 1 family member A2)
- METTL9 (methyltransferase like 9)
- IL4R (interleukin 4 receptor)
- ITGAX (integrin subunit alpha X)
- KIFC3 (kinesin family member C3)
- ATP6V0D1 (ATPase H+ transporting V0 subunit d1)
- CCL3-AS1 (CCL3 antisense RNA 1)
- STAT3 (signal transducer and activator of transcription 3)
- SOCS3 (suppressor of cytokine signaling 3)
- GNA15-DT (GNA15 divergent transcript)
- STXBP2 (syntaxin binding protein 2)
- HOOK2 (hook microtubule tethering protein 2)
- JUNB (JunB proto-oncogene, AP-1 transcription factor
- LINC01764 (long intergenic non-protein coding RNA 1764)
- IFI30 (IFI30 lysosomal thiol reductase)
- MAP4K1-AS1 (MAP4K1 antisense RNA 1)
- LIPE-AS1 (LIPE antisense RNA 1)
- CEACAM1 (CEA cell adhesion molecule 1)
- CD177 (CD177 molecule)
- SIGLEC10-AS1 (SIGLEC10 antisense RNA 1)
- LINC01890 (long intergenic non-protein coding RNA 1890)
- PCBP1-AS1 (PCBP1 antisense RNA 1)
- LINC01943 (long intergenic non-protein coding RNA 1943)
- IGKC (immunoglobulin kappa constant)
- NABP1 (nucleic acid binding protein 1)
- CXCR2 (C-X-C motif chemokine receptor 2)
- SLC12A5-AS1 (SLC12A5 and MMP9 antisense RNA 1)
- SLCO4A1-AS1 (SLCO4A1 antisense RNA 1)
- MX2 (MX dynamin like GTPase 2)
- LINC00528 (long intergenic non-protein coding RNA 528)
- NCF4-AS1 (NCF4 antisense RNA 1)
- PLA2G6 (phospholipase A2 group VI)
- TYMP (thymidine phosphorylase)
- CCR1 (C-C motif chemokine receptor 1)
- CCR5AS (CCR5 antisense RNA)
- CXCL5 (C-X-C motif chemokine ligand 5)
- FNIP1 (folliculin interacting protein 1)
- EGR1 (early growth response 1)
- C5orf58 (chromosome 5 open reading frame 58)
- EDN1 (endothelin 1)
- IER3-AS1 (IER3 antisense RNA 1)
- HCG20 (HLA complex group 20 (non-protein coding))
- PSORS1C1 (psoriasis susceptibility 1 candidate 1)
- CDSN (corneodesmosin)
- TNF (tumor necrosis factor)
- WAKMAR2 (wound and keratinocyte migration associated
- SOD2 (superoxide dismutase 2)
- NAMPT (nicotinamide phosphoribosyltransferase)
- LINC-PINT (long intergenic non-protein coding RNA, p53
- CYREN (cell cycle regulator of NHEJ)
- TBXAS1 (thromboxane A synthase 1)
- TRBC2 (T cell receptor beta constant 2)
- SDCBP (syndecan binding protein)
- FTH1P1 (ferritin heavy chain 1 pseudogene 1)
- FCGR1CP (Fc fragment of IgG receptor Ic, pseudogene)
- SPRR2C (small proline rich protein 2C (pseudogene))
- FTH1P2 (ferritin heavy chain 1 pseudogene 2)
- NAMPTP1 (nicotinamide phosphoribosyltransferase
- FTH1P16 (ferritin heavy chain 1 pseudogene 16)
- FTH1P7 (ferritin heavy chain 1 pseudogene 7)
- KRT17P6 (keratin 17 pseudogene 6)
- FTH1P3 (ferritin heavy chain 1 pseudogene 3)
- FTH1P20 (ferritin heavy chain 1 pseudogene 20)
- IMPDH1P10 (inosine monophosphate dehydrogenase 1
- FTH1P23 (ferritin heavy chain 1 pseudogene 23)
- FTH1P4 (ferritin heavy chain 1 pseudogene 4)
- IFITM3P1 (IFITM3 pseudogene 1)
- FTH1P10 (ferritin heavy chain 1 pseudogene 10)
- FTH1P5 (ferritin heavy chain 1 pseudogene 5)
- NCF1C (neutrophil cytosolic factor 1C pseudogene)
- FTH1P11 (ferritin heavy chain 1 pseudogene 11)
- FTH1P12 (ferritin heavy chain 1 pseudogene 12)
- SDCBPP3 (syndecan binding protein pseudogene 3)
- GK4P (glycerol kinase 4 pseudogene)
- FTH1P8 (ferritin heavy chain 1 pseudogene 8)

Up

- TTLL10-AS1 (TTLL10 antisense RNA 1)
- TTLL10 (tubulin tyrosine ligase like 10)
- MORN1 (MORN repeat containing 1)
- PLCH2 (phospholipase C eta 2)
- TTC34 (tetratricopeptide repeat domain 34)
- RERE (arginine-glutamic acid dipeptide repeats)
- EXOSC10 (exosome component 10)
- FHAD1 (forkhead associated phosphopeptide binding
- FHAD1-AS1 (FHAD1 antisense RNA 1)
- NBPF1 (NBPF member 1)
- CDC42-AS1 (CDC42 antisense RNA 1)
- NCMAP-DT (NCMAP divergent transcript)
- TMEM50A (transmembrane protein 50A)
- HMGN2 (high mobility group nucleosomal binding domain
- COL16A1 (collagen type XVI alpha 1 chain)
- TEKT2 (tektin 2)
- GNL2 (G protein nucleolar 2)
- EBNA1BP2 (EBNA1 binding protein 2)
- DMAP1 (DNA methyltransferase 1 associated protein 1)
- LINC01144 (long intergenic non-protein coding RNA 1144)
- NASP (nuclear autoantigenic sperm protein)
- NSUN4 (NOP2/Sun RNA methyltransferase 4)
- ATPAF1 (ATP synthase mitochondrial F1 complex assembly
- CDKN2C (cyclin dependent kinase inhibitor 2C)
- HSPB11 (heat shock protein family B (small) member 11)
- MIGA1 (mitoguardin 1)
- F3 (coagulation factor III, tissue factor)
- SLC35A3 (solute carrier family 35 member A3)
- EXTL2 (exostosin like glycosyltransferase 2)
- SARS1 (seryl-tRNA synthetase 1)
- ATP5PB (ATP synthase peripheral stalk-membrane subunit
- LINC01765 (long intergenic non-protein coding RNA 1765)
- WARS2-AS1 (WARS2 antisense RNA 1)
- NBPF10 (NBPF member 10)
- C2CD4D-AS1 (C2CD4D and THEM5 antisense RNA 1)
- CHTOP (chromatin target of PRMT1)
- DCST1-AS1 (DCST1 antisense RNA 1)
- LINC01133 (long intergenic non-protein coding RNA 1133)
- DCAF6 (DDB1 and CUL4 associated factor 6)
- VAMP4 (vesicle associated membrane protein 4)
- PRDX6 (peroxiredoxin 6)
- RO60 (Ro60, Y RNA binding protein)
- LINC00467 (long intergenic non-protein coding RNA 467)
- ESRRG (estrogen related receptor gamma)
- SPATA17-AS1 (SPATA17 antisense RNA 1)
- CAPN2 (calpain 2)
- EPHX1 (epoxide hydrolase 1)
- ACBD3-AS1 (ACBD3 antisense RNA 1)
- AGT (angiotensinogen)
- TARBP1 (TAR (HIV-1) RNA binding protein 1)
- LINC01132 (long intergenic non-protein coding RNA 1132)
- TBCE (tubulin folding cofactor E)
- SDCCAG8 (SHH signaling and ciliogenesis regulator
- CALML3-AS1 (CALML3 antisense RNA 1)
- BEND7 (BEN domain containing 7)
- SUV39H2-DT (SUV39H2 divergent transcript)
- RPP38-DT (RPP38 divergent transcript)
- NMT2 (N-myristoyltransferase 2)
- ENKUR (enkurin, TRPC channel interacting protein)
- ZNF248 (zinc finger protein 248)
- ZNF32-AS1 (ZNF32 antisense RNA 1)
- ZNF22-AS1 (ZNF22 antisense RNA 1)
- TIMM23B (translocase of inner mitochondrial membrane 23
- SGMS1-AS1 (SGMS1 antisense RNA 1)
- REEP3 (receptor accessory protein 3)
- DNAJC9-AS1 (DNAJC9 antisense RNA 1)
- CFAP70 (cilia and flagella associated protein 70)
- PPP3CB (protein phosphatase 3 catalytic subunit beta)
- NUTM2B-AS1 (NUTM2B antisense RNA 1)
- ANXA11 (annexin A11)
- PDLIM1 (PDZ and LIM domain 1)
- DNMBP (dynamin binding protein)
- DNMBP-AS1 (DNMBP antisense RNA 1)
- C10orf95 (chromosome 10 open reading frame 95)
- CASP7 (caspase 7)
- PLEKHS1 (pleckstrin homology domain containing S1)
- HSPA12A (heat shock protein family A (Hsp70) member
- CASC2 (cancer susceptibility 2)
- WDR11 (WD repeat domain 11)
- TACC2 (transforming acidic coiled-coil containing protein 2)
- LHPP (phospholysine phosphohistidine inorganic
- MUC5B-AS1 (MUC5B antisense RNA 1)
- STIM1-AS1 (STIM1 antisense RNA 1)
- TMEM9B-AS1 (TMEM9B antisense RNA 1)
- WEE1 (WEE1 G2 checkpoint kinase)
- ZBED5 (zinc finger BED-type containing 5)
- MICAL2 (microtubule associated monooxygenase, calponin
- TEAD1 (TEA domain transcription factor 1)
- NUCB2 (nucleobindin 2)
- CSRP3-AS1 (CSRP3 and E2F8 antisense RNA 1)
- NAV2-AS1 (NAV2 antisense RNA 1)
- GAS2 (growth arrest specific 2)
- ANO3 (anoctamin 3)
- LINC00294 (long intergenic non-protein coding RNA 294)
- CD59 (CD59 molecule (CD59 blood group))
- PRDM11 (PR/SET domain 11)
- CD6 (CD6 molecule)
- DDB1 (damage specific DNA binding protein 1)
- CYB561A3 (cytochrome b561 family member A3)
- FADS1 (fatty acid desaturase 1)
- ASRGL1 (asparaginase and isoaspartyl peptidase 1)
- RBM4B (RNA binding motif protein 4B)
- RAD9A (RAD9 checkpoint clamp component A)
- ALDH3B1 (aldehyde dehydrogenase 3 family member B1)
- PRSS23 (serine protease 23)
- EXPH5 (exophilin 5)
- COLCA1 (colorectal cancer associated 1)
- BACE1-AS (BACE1 antisense RNA)
- TRIM29 (tripartite motif containing 29)
- SIAE (sialic acid acetylesterase)
- AKAP3 (A-kinase anchoring protein 3)
- MIR200CHG (MIR200C and MIR141 host gene)
- YBX3 (Y-box binding protein 3)
- H2AJ (H2A.J histone)
- PKP2 (plakophilin 2)
- FKBP11 (FKBP prolyl isomerase 11)
- TUBA1C (tubulin alpha 1c)
- KRT8 (keratin 8)
- MYG1-AS1 (MYG1 antisense RNA 1)
- PCBP2 (poly(rC) binding protein 2)
- CBX5 (chromobox 5)
- PTGES3 (prostaglandin E synthase 3)
- RXYLT1-AS1 (RXYLT1 antisense RNA 1)
- CAPS2 (calcyphosine 2)
- LRRIQ1 (leucine rich repeats and IQ motif containing 1)
- UBE2N (ubiquitin conjugating enzyme E2 N)
- TMPO-AS1 (TMPO antisense RNA 1)
- IGF1 (insulin like growth factor 1)
- MVK (mevalonate kinase)
- C12orf76 (chromosome 12 open reading frame 76)
- IQCD (IQ motif containing D)
- CCDC60 (coiled-coil domain containing 60)
- ANAPC5 (anaphase promoting complex subunit 5)
- SNRNP35 (small nuclear ribonucleoprotein U11/U12
- DNAH10OS (dynein axonemal heavy chain 10 opposite
- LINC00332 (long intergenic non-protein coding RNA 332)
- NEK5 (NIMA related kinase 5)
- FARP1 (FERM, ARH/RhoGEF and pleckstrin domain protein
- ARGLU1 (arginine and glutamate rich 1)
- PRECSIT (p53 regulated carcinoma associated Stat3
- MCF2L-AS1 (MCF2L antisense RNA 1)
- GRTP1 (growth hormone regulated TBC protein 1)
- UPF3A (UPF3A regulator of nonsense mediated mRNA
- ANG (angiogenin)
- EGILA (EGFR interacting lncRNA)
- HNRNPC (heterogeneous nuclear ribonucleoprotein C)
- OXA1L (OXA1L mitochondrial inner membrane protein)
- ACIN1 (apoptotic chromatin condensation inducer 1)
- ZFHX2 (zinc finger homeobox 2)
- DHRS4-AS1 (DHRS4 antisense RNA 1)
- LINC02300 (long intergenic non-protein coding RNA 2300)
- HEATR5A (HEAT repeat containing 5A)
- SLC25A21-AS1 (SLC25A21 antisense RNA 1)
- KLHL28 (kelch like family member 28)
- SAV1 (salvador family WW domain containing protein 1)
- PCNX4 (pecanex 4)
- SALRNA1 (senescence associated long non-coding RNA 1)
- FUT8-AS1 (FUT8 antisense RNA 1)
- EIF2S1 (eukaryotic translation initiation factor 2 subunit
- PTGR2 (prostaglandin reductase 2)
- YLPM1 (YLP motif containing 1)
- FOXN3-AS1 (FOXN3 antisense RNA 1)
- GLRX5 (glutaredoxin 5)
- PAPOLA-DT (PAPOLA divergent transcript)
- PPP2R5C (protein phosphatase 2 regulatory subunit
- AMN (amnion associated transmembrane protein)
- SIVA1 (SIVA1 apoptosis inducing factor)
- CLBA1 (clathrin binding box of aftiphilin containing 1)
- NDN (necdin, MAGE family member)
- SNHG14 (small nucleolar RNA host gene 14)
- KLF13 (Kruppel like factor 13)
- LINC02345 (long intergenic non-protein coding RNA 2345)
- OIP5-AS1 (OIP5 antisense RNA 1)
- MAP1A (microtubule associated protein 1A)
- PDIA3 (protein disulfide isomerase family A member 3)
- DUOX1 (dual oxidase 1)
- ATP8B4 (ATPase phospholipid transporting 8B4 (putative))
- TEX9 (testis expressed 9)

- MYO1E (myosin IE)

- LINC02568 (long intergenic non-protein coding RNA 2568)
- MAP2K5-DT (MAP2K5 divergent transcript)
- CCDC33 (coiled-coil domain containing 33)
- CLK3 (CDC like kinase 3)
- GOLGA6L9 (golgin A6 family like 9)
- HOMER2 (homer scaffold protein 2)
- WDR93 (WD repeat domain 93)
- ZNF710-AS1 (ZNF710 antisense RNA 1)
- NGRN (neugrin, neurite outgrowth associated)
- SNHG19 (small nucleolar RNA host gene 19)
- ABAT (4-aminobutyrate aminotransferase)
- LINC02185 (long intergenic non-protein coding RNA 2185)
- TMC5 (transmembrane channel like 5)
- GDE1 (glycerophosphodiester phosphodiesterase 1)
- DNAH3 (dynein axonemal heavy chain 3)
- RN7SKP23 (RN7SK pseudogene 23)
- SULT1A1 (sulfotransferase family 1A member 1)
- CD2BP2-DT (CD2BP2 divergent transcript)
- ZNF771 (zinc finger protein 771)
- RNF40 (ring finger protein 40)
- BCL7C (BAF chromatin remodeling complex subunit BCL7C)
- ZNF720 (zinc finger protein 720)
- ITFG1 (integrin alpha FG-GAP repeat containing 1)
- FTO (FTO alpha-ketoglutarate dependent dioxygenase)
- LINC00920 (long intergenic non-protein coding RNA 920)

Subgroup D

Down

- TTLL10-AS1 (TTLL10 antisense RNA 1)
- TTLL10 (tubulin tyrosine ligase like 10)
- INTS11 (integrator complex subunit 11)
- CPTP (ceramide-1-phosphate transfer protein)
- FAAP20 (FA core complex associated protein 20)
- MORN1 (MORN repeat containing 1)
- PLCH2 (phospholipase C eta 2)
- TNFRSF14-AS1 (TNFRSF14 antisense RNA 1)
- TTC34 (tetratricopeptide repeat domain 34)
- VAMP3 (vesicle associated membrane protein 3)
- UTS2 (urotensin 2)
- RERE (arginine-glutamic acid dipeptide repeats)
- EXOSC10 (exosome component 10)
- C1orf167 (chromosome 1 open reading frame 167)
- MTHFR (methylenetetrahydrofolate reductase)
- FHAD1 (forkhead associated phosphopeptide binding
- FHAD1-AS1 (FHAD1 antisense RNA 1)
- ARHGEF19-AS1 (ARHGEF19 antisense RNA 1)
- NECAP2 (NECAP endocytosis associated 2)
- NBPF1 (NBPF member 1)
- EMC1-AS1 (EMC1 antisense RNA 1)
- CDC42-AS1 (CDC42 antisense RNA 1)
- KDM1A (lysine demethylase 1A)
- SRSF10 (serine and arginine rich splicing factor 10)
- TMEM50A (transmembrane protein 50A)
- ZNF593OS (ZNF593 opposite strand)
- CD52 (CD52 molecule)
- HMGN2 (high mobility group nucleosomal binding domain
- NUDC (nuclear distribution C, dynein complex regulator)
- SRSF4 (serine and arginine rich splicing factor 4)
- LAPTM5 (lysosomal protein transmembrane 5)
- COL16A1 (collagen type XVI alpha 1 chain)
- EIF3I (eukaryotic translation initiation factor 3 subunit I)
- ZBTB8OS (zinc finger and BTB domain containing 8 opposite
- ZMYM4-AS1 (ZMYM4 antisense RNA 1)
- KIAA0319L (KIAA0319 like)
- TEKT2 (tektin 2)
- CSF3R (colony stimulating factor 3 receptor)
- LINC01137 (long intergenic non-protein coding RNA 1137)
- GNL2 (G protein nucleolar 2)
- NDUFS5 (NADH:ubiquinone oxidoreductase subunit S5)
- NFYC-AS1 (NFYC antisense RNA 1)
- SLFNL1-AS1 (SLFNL1 antisense RNA 1)
- TMEM269 (transmembrane protein 269)
- EBNA1BP2 (EBNA1 binding protein 2)
- DMAP1 (DNA methyltransferase 1 associated protein 1)
- RNF220 (ring finger protein 220)
- BTBD19 (BTB domain containing 19)
- LINC01144 (long intergenic non-protein coding RNA 1144)
- AKR1A1 (aldo-keto reductase family 1 member A1)
- NASP (nuclear autoantigenic sperm protein)
- POMGNT1 (protein O-linked mannose N-
- NSUN4 (NOP2/Sun RNA methyltransferase 4)
- MKNK1-AS1 (MKNK1 antisense RNA 1)
- ATPAF1 (ATP synthase mitochondrial F1 complex assembly
- FOXD2-AS1 (FOXD2 adjacent opposite strand RNA 1)
- CDKN2C (cyclin dependent kinase inhibitor 2C)
- TTC39A-AS1 (TTC39A antisense RNA 1)
- HSPB11 (heat shock protein family B (small) member 11)
- NFIA (nuclear factor I A)
- TM2D1 (TM2 domain containing 1)
- ITGB3BP (integrin subunit beta 3 binding protein)
- SPATA1 (spermatogenesis associated 1)
- CCN1 (cellular communication network factor 1)
- GBP5 (guanylate binding protein 5)
- F3 (coagulation factor III, tissue factor)
- DPYD-AS1 (DPYD antisense RNA 1)
- SLC35A3 (solute carrier family 35 member A3)
- EXTL2 (exostosin like glycosyltransferase 2)
- TMEM167B-DT (TMEM167B divergent transcript)
- SARS1 (seryl-tRNA synthetase 1)
- GNAT2 (G protein subunit alpha transducin 2)
- CEPT1 (choline/ethanolamine phosphotransferase 1)
- DENND2D (DENN domain containing 2D)
- ATP5PB (ATP synthase peripheral stalk-membrane subunit
- TRIM33 (tripartite motif containing 33)
- NRAS (NRAS proto-oncogene, GTPase)
- LINC01765 (long intergenic non-protein coding RNA 1765)
- ATP1A1 (ATPase Na+/K+ transporting subunit alpha 1)
- ATP1A1-AS1 (ATP1A1 antisense RNA 1)
- CD2 (CD2 molecule)
- WARS2-AS1 (WARS2 antisense RNA 1)
- LINC00623 (long intergenic non-protein coding RNA 623)
- LIX1L-AS1 (LIX1L antisense RNA 1)
- NBPF10 (NBPF member 10)
- FMO5 (flavin containing dimethylaniline monoxygenase 5)
- LINC00624 (long intergenic non-protein coding RNA 624)
- CERS2 (ceramide synthase 2)
- OAZ3 (ornithine decarboxylase antizyme 3)
- C2CD4D-AS1 (C2CD4D and THEM5 antisense RNA 1)
- S100A11 (S100 calcium binding protein A11)
- CHTOP (chromatin target of PRMT1)
- ADAR (adenosine deaminase RNA specific)
- PMVK (phosphomevalonate kinase)
- DCST1-AS1 (DCST1 antisense RNA 1)
- MUC1 (mucin 1, cell surface associated)
- FDPS (farnesyl diphosphate synthase)
- RUSC1-AS1 (RUSC1 antisense RNA 1)
- ASH1L-AS1 (ASH1L antisense RNA 1)
- ARHGEF2 (Rho/Rac guanine nucleotide exchange factor 2)
- GPATCH4 (G-patch domain containing 4)
- MNDA (myeloid cell nuclear differentiation antigen)
- IFI16 (interferon gamma inducible protein 16)
- LINC01133 (long intergenic non-protein coding RNA 1133)
- DCAF8 (DDB1 and CUL4 associated factor 8)
- PPOX (protoporphyrinogen oxidase)
- NR1I3 (nuclear receptor subfamily 1 group I member 3)
- MPC2 (mitochondrial pyruvate carrier 2)
- DCAF6 (DDB1 and CUL4 associated factor 6)
- C1orf112 (chromosome 1 open reading frame 112)
- VAMP4 (vesicle associated membrane protein 4)
- PIGC (phosphatidylinositol glycan anchor biosynthesis class
- PRDX6 (peroxiredoxin 6)
- GAS5-AS1 (GAS5 antisense RNA 1)
- GLUL (glutamate-ammonia ligase)
- IVNS1ABP (influenza virus NS1A binding protein)
- TPR (translocated promoter region, nuclear basket protein)
- UCHL5 (ubiquitin C-terminal hydrolase L5)
- RO60 (Ro60, Y RNA binding protein)
- BLACAT1 (bladder cancer associated transcript 1)
- RAB29 (RAB29, member RAS oncogene family)
- MIR29B2CHG (MIR29B2 and MIR29C host gene)
- LINC02767 (long intergenic non-protein coding RNA 2767)
- MIR205HG (MIR205 host gene)
- LINC00467 (long intergenic non-protein coding RNA 467)
- ANGEL2 (angel homolog 2)
- ESRRG (estrogen related receptor gamma)
- SPATA17-AS1 (SPATA17 antisense RNA 1)
- AIDA (axin interactor, dorsalization associated)
- BROX (BRO1 domain and CAAX motif containing)
- FAM177B (family with sequence similarity 177 member B)
- CAPN2 (calpain 2)
- TMEM63A (transmembrane protein 63A)
- TRIM11 (tripartite motif containing 11)
- COA6-AS1 (COA6 antisense RNA 1)
- IRF2BP2 (interferon regulatory factor 2 binding protein 2)
- LINC01348 (long intergenic non-protein coding RNA 1348)
- SDCCAG8 (SHH signaling and ciliogenesis regulator
- USP6NL-AS1 (USP6NL antisense RNA 1)
- SUV39H2-DT (SUV39H2 divergent transcript)
- RPP38-DT (RPP38 divergent transcript)
- NMT2 (N-myristoyltransferase 2)
- VIM-AS1 (VIM antisense RNA 1)
- ENKUR (enkurin, TRPC channel interacting protein)
- YME1L1 (YME1 like 1 ATPase)
- WAC-AS1 (WAC antisense RNA 1 (head to head))
- MAP3K8 (mitogen-activated protein kinase kinase kinase 8)
- KIF5B (kinesin family member 5B)
- CCDC7 (coiled-coil domain containing 7)
- ZNF248 (zinc finger protein 248)
- ZNF33A (zinc finger protein 33A)
- ZNF33B (zinc finger protein 33B)
- ZNF32-AS1 (ZNF32 antisense RNA 1)
- ZNF32-AS2 (ZNF32 antisense RNA 2)
- RASSF4 (Ras association domain family member 4)
- ZNF22-AS1 (ZNF22 antisense RNA 1)
- TIMM23B (translocase of inner mitochondrial membrane 23
- SGMS1-AS1 (SGMS1 antisense RNA 1)
- JMJD1C (jumonji domain containing 1C)
- PPA1 (inorganic pyrophosphatase 1)
- ASCC1 (activating signal cointegrator 1 complex subunit 1)
- DNAJC9-AS1 (DNAJC9 antisense RNA 1)
- CFAP70 (cilia and flagella associated protein 70)
- PPP3CB (protein phosphatase 3 catalytic subunit beta)
- FUT11 (fucosyltransferase 11)
- NUTM2B-AS1 (NUTM2B antisense RNA 1)
- ANXA11 (annexin A11)
- CERNA2 (competing endogenous lncRNA 2 for microRNA let-
- SHLD2 (shieldin complex subunit 2)
- ACTA2-AS1 (ACTA2 antisense RNA 1)
- IFIT3 (interferon induced protein with tetratricopeptide
- PCGF5 (polycomb group ring finger 5)
- BTAF1 (B-TFIID TATA-box binding protein associated factor
- HELLS (helicase, lymphoid specific)
- PDLIM1 (PDZ and LIM domain 1)
- PGAM1 (phosphoglycerate mutase 1)
- HPS1 (HPS1 biogenesis of lysosomal organelles complex 3
- DNMBP (dynamin binding protein)

- DNMBP-AS1 (DNMBP antisense RNA 1)

- NDUFB8 (NADH:ubiquinone oxidoreductase subunit B8)
- HIF1AN (hypoxia inducible factor 1 subunit alpha inhibitor)
- TWNK (twinkle mtDNA helicase)
- DPCD (deleted in primary ciliary dyskinesia homolog
- PSD (pleckstrin and Sec7 domain containing)
- C10orf95 (chromosome 10 open reading frame 95)
- CFAP58-DT (CFAP58 divergent transcript)
- RNU4-5P (RNA, U4 small nuclear 5, pseudogene)
- BBIP1 (BBSome interacting protein 1)
- ZDHHC6 (zinc finger DHHC-type palmitoyltransferase 6)
- VTI1A (vesicle transport through interaction with t-SNAREs
- CASP7 (caspase 7)
- PLEKHS1 (pleckstrin homology domain containing S1)
- HSPA12A (heat shock protein family A (Hsp70) member
- CASC2 (cancer susceptibility 2)
- LINC00867 (long intergenic non-protein coding RNA 867)
- WDR11 (WD repeat domain 11)
- PSTK (phosphoseryl-tRNA kinase)
- LHPP (phospholysine phosphohistidine inorganic
- TUBGCP2 (tubulin gamma complex associated protein 2)
- TMEM80 (transmembrane protein 80)
- CD151 (CD151 molecule (Raph blood group))
- MUC2 (mucin 2, oligomeric mucus/gel-forming)
- MUC5B-AS1 (MUC5B antisense RNA 1)
- PRR33 (proline rich 33)

Up

- CLCA4-AS1 (CLCA4 antisense RNA 1)
- PRKG1-AS1 (PRKG1 antisense RNA 1)
- IL18 (interleukin 18)
- LINC02448 (long intergenic non-protein coding RNA 2448)
- SCEL-AS1 (SCEL antisense RNA 1)
- DNAJC17 (DnaJ heat shock protein family (Hsp40) member
- GCNT3 (glucosaminyl (N-acetyl) transferase 3, mucin type)
- KRT13 (keratin 13)
- LIPE-AS1 (LIPE antisense RNA 1)
- XPO1 (exportin 1)
- TNK2 (tyrosine kinase non receptor 2)
- SRD5A3-AS1 (SRD5A3 antisense RNA 1)
- CDKN2B-AS1 (CDKN2B antisense RNA 1)

MARS1 (methionyl-tRNA synthetase 1)
LINC02448 (long intergenic non-protein coding RNA 2448)
USP15 (ubiquitin specific peptidase 15)
RXYLT1-AS1 (RXYLT1 antisense RNA 1)
IRAK3 (interleukin 1 receptor associated kinase 3)
PRANCR (progenitor renewal associated non-coding RNA)
THAP2 (THAP domain containing 2)
CAPS2 (calcyphosine 2)
PPP1R12A-AS1 (PPP1R12A antisense RNA 1)
LRRIQ1 (leucine rich repeats and IQ motif containing 1)
ATP2B1-AS1 (ATP2B1 antisense RNA 1)
PLEKHG7 (pleckstrin homology and RhoGEF domain
UBE2N (ubiquitin conjugating enzyme E2 N)
LTA4H (leukotriene A4 hydrolase)
CHPT1 (choline phosphotransferase 1)
IGF1 (insulin like growth factor 1)
USP30-AS1 (USP30 antisense RNA 1)
TCHP (trichoplein keratin filament binding)
GIT2 (GIT ArfGAP 2)
C12orf76 (chromosome 12 open reading frame 76)
ATXN2 (ataxin 2)
OAS1 (2'-5'-oligoadenylate synthetase 1)
IQCD (IQ motif containing D)
RFC5 (replication factor C subunit 5)
CCDC60 (coiled-coil domain containing 60)
PXN-AS1 (PXN antisense RNA 1)
SRSF9 (serine and arginine rich splicing factor 9)
NRAV (negative regulator of antiviral response)
ORAI1 (ORAI calcium release-activated calcium modulator
CFAP251 (cilia and flagella associated protein 251)
SNRNP35 (small nuclear ribonucleoprotein U11/U12
TMED2 (transmembrane p24 trafficking protein 2)
DNAH10 (dynein axonemal heavy chain 10)
DNAH10OS (dynein axonemal heavy chain 10 opposite
LINC02361 (long intergenic non-protein coding RNA 2361)
ZNF26 (zinc finger protein 26)
LINC00332 (long intergenic non-protein coding RNA 332)
VWA8-AS1 (VWA8 antisense RNA 1 (head to head))
CPB2-AS1 (CPB2 antisense RNA 1)
NEK5 (NIMA related kinase 5)
LMO7-AS1 (LMO7 antisense RNA 1)
FBXL3 (F-box and leucine rich repeat protein 3)
SCEL-AS1 (SCEL antisense RNA 1)
RBM26 (RNA binding motif protein 26)
SOX21-AS1 (SOX21 antisense divergent transcript 1)
FARP1-AS1 (FARP1 antisense RNA 1)
PCCA-DT (PCCA divergent transcript)
FGF14-AS2 (FGF14 antisense RNA 2)
ARGLU1 (arginine and glutamate rich 1)
MYO16-AS1 (MYO16 antisense RNA 1)
PRECSIT (p53 regulated carcinoma associated Stat3
ANKRD10-IT1 (ANKRD10 intronic transcript 1)
MCF2L-AS1 (MCF2L antisense RNA 1)
PCID2 (PCI domain containing 2)
UPF3A (UPF3A regulator of nonsense mediated mRNA
ANG (angiogenin)
LINC00641 (long intergenic non-protein coding RNA 641)
HNRNPC (heterogeneous nuclear ribonucleoprotein C)
SUPT16H (SPT16 homolog, facilitates chromatin remodeling
TRDC (T cell receptor delta constant)
OXA1L (OXA1L mitochondrial inner membrane protein)
ACIN1 (apoptotic chromatin condensation inducer 1)
BCL2L2 (BCL2 like 2)
ZFHX2 (zinc finger homeobox 2)
NEDD8 (NEDD8 ubiquitin like modifier)
SDR39U1 (short chain dehydrogenase/reductase family 39U
HEATR5A (HEAT repeat containing 5A)
ARHGAP5-AS1 (ARHGAP5 antisense RNA 1 (head to head))
FAM177A1 (family with sequence similarity 177 member
PAX9 (paired box 9)
SLC25A21-AS1 (SLC25A21 antisense RNA 1)
KLHL28 (kelch like family member 28)
RN7SL1 (RNA component of signal recognition particle 7SL1)
SAV1 (salvador family WW domain containing protein 1)
PYGL (glycogen phosphorylase L)
LGALS3 (galectin 3)
AP5M1 (adaptor related protein complex 5 subunit mu 1)
PSMA3-AS1 (PSMA3 antisense RNA 1)
CCDC175 (coiled-coil domain containing 175)
PCNX4 (pecanex 4)
HIF1A-AS1 (HIF1A antisense RNA 1)
ZBTB25 (zinc finger and BTB domain containing 25)
FUT8-AS1 (FUT8 antisense RNA 1)
EIF2S1 (eukaryotic translation initiation factor 2 subunit
ACTN1 (actinin alpha 1)
SLC39A9 (solute carrier family 39 member 9)
SIPA1L1 (signal induced proliferation associated 1 like 1)
RBM25 (RNA binding motif protein 25)
PTGR2 (prostaglandin reductase 2)
ENTPD5 (ectonucleoside triphosphate diphosphohydrolase
NPC2 (NPC intracellular cholesterol transporter 2)
YLPM1 (YLP motif containing 1)
FOS (Fos proto-oncogene, AP-1 transcription factor subunit)
GPATCH2L (G-patch domain containing 2 like)
VASH1-AS1 (VASH1 antisense RNA 1)
SURP (SRA stem-loop interacting RNA binding protein)
FOXN3-AS1 (FOXN3 antisense RNA 1)
DGLUCY (D-glutamate cyclase)
IFI27 (interferon alpha inducible protein 27)
GLRX5 (glutaredoxin 5)
PAPOLA-DT (PAPOLA divergent transcript)
EVL (Enah/Vasp-like)
LINC00239 (long intergenic non-protein coding RNA 239)
PPP2R5C (protein phosphatase 2 regulatory subunit
AMN (amnion associated transmembrane protein)
MARK3 (microtubule affinity regulating kinase 3)
CLBA1 (clathrin binding box of aftiphilin containing 1)
NDN (necdin, MAGE family member)
SNHG14 (small nucleolar RNA host gene 14)
KLF13 (Kruppel like factor 13)
LINC02345 (long intergenic non-protein coding RNA 2345)
THBS1-AS1 (THBS1 antisense RNA 1)
DNAJC17 (DnaJ heat shock protein family (Hsp40) member
OIP5-AS1 (OIP5 antisense RNA 1)
GANC (glucosidase alpha, neutral C)
MAP1A (microtubule associated protein 1A)
PDIA3 (protein disulfide isomerase family A member 3)
MIR1282 (microRNA 1282)
EIF3J (eukaryotic translation initiation factor 3 subunit J)
PATL2 (PAT1 homolog 2)
B2M (beta-2-microglobulin)
TRIM69 (tripartite motif containing 69)
DUOX1 (dual oxidase 1)
GABPB1-AS1 (GABPB1 antisense RNA 1)
FAM214A (family with sequence similarity 214 member A)
TEX9 (testis expressed 9)
GCOM1 (GCOM1, MYZAP-POLR2M combined locus)
ALDH1A2 (aldehyde dehydrogenase 1 family member A2)
MYO1E (myosin IE)
BNIP2 (BCL2 interacting protein 2)
ANXA2 (annexin A2)
RORA (RAR related orphan receptor A)
LINC02568 (long intergenic non-protein coding RNA 2568)
USP3-AS1 (USP3 antisense RNA 1)
OAZ2 (ornithine decarboxylase antizyme 2)
MAP2K5-DT (MAP2K5 divergent transcript)
GLCE (glucuronic acid epimerase)
ADPGK (ADP dependent glucokinase)
ADPGK-AS1 (ADPGK antisense RNA 1)
NPTN-IT1 (NPTN intronic transcript 1)
PML (PML nuclear body scaffold)
CCDC33 (coiled-coil domain containing 33)
CLK3 (CDC like kinase 3)
PPCDC (phosphopantothenoylcysteine decarboxylase)
C15orf39 (chromosome 15 open reading frame 39)
DNAJA4 (DnaJ heat shock protein family (Hsp40) member
ST20-AS1 (ST20 antisense RNA 1)
GOLGA6L9 (golgin A6 family like 9)
HOMER2 (homer scaffold protein 2)
WDR93 (WD repeat domain 93)
ZNF710-AS1 (ZNF710 antisense RNA 1)
NGRN (neugrin, neurite outgrowth associated)
CHASERR (CHD2 adjacent suppressive regulatory RNA)
MCTP2 (multiple C2 and transmembrane domain
SNRNP25 (small nuclear ribonucleoprotein U11/U12
SNHG19 (small nucleolar RNA host gene 19)
LINC00921 (long intergenic non-protein coding RNA 921)
ABAT (4-aminobutyrate aminotransferase)
TMEM186 (transmembrane protein 186)
C16orf72 (chromosome 16 open reading frame 72)
CIITA (class II major histocompatibility complex
LINC02185 (long intergenic non-protein coding RNA 2185)
NDE1 (nudE neurodevelopment protein 1)
TMC5 (transmembrane channel like 5)
GDE1 (glycerophosphodiester phosphodiesterase 1)
DNAH3 (dynein axonemal heavy chain 3)
METTL9 (methyltransferase like 9)
SULT1A1 (sulfotransferase family 1A member 1)
CD2BP2-DT (CD2BP2 divergent transcript)
RNF40 (ring finger protein 40)
BCL7C (BAF chromatin remodeling complex subunit BCL7C)
FBXL19-AS1 (FBXL19 antisense RNA 1)
FUS (FUS RNA binding protein)
PYCARD (PYD and CARD domain containing)
ITGAX (integrin subunit alpha X)
ZNF720 (zinc finger protein 720)
ITFG1 (integrin alpha FG-GAP repeat containing 1)
CYLD-AS1 (CYLD antisense RNA 1)
FTO (FTO alpha-ketoglutarate dependent dioxygenase)
POLR2C (RNA polymerase II subunit C)
KIFC3 (kinesin family member C3)
SETD6 (SET domain containing 6, protein lysine
LINC00920 (long intergenic non-protein coding RNA 920)
CMTM4 (CKLF like MARVEL transmembrane domain
DYNC1LI2 (dynein cytoplasmic 1 light intermediate chain 2)
ATP6V0D1 (ATPase H+ transporting V0 subunit d1)
TERF2 (telomeric repeat binding factor 2)
VAC14 (VAC14 component of PIKFYVE complex)
CMTR2 (cap methyltransferase 2)
ZNF821 (zinc finger protein 821)
PMFBP1 (polyamine modulated factor 1 binding protein 1)
WDR59 (WD repeat domain 59)
ADAT1 (adenosine deaminase tRNA specific 1)
DNAAF1 (dynein axonemal assembly factor 1)
ATP2C2-AS1 (ATP2C2 antisense RNA 1)
USP10 (ubiquitin specific peptidase 10)
CIBAR2 (CBY1 interacting BAR domain containing 2)
LINC02188 (long intergenic non-protein coding RNA 2188)
LINC02166 (long intergenic non-protein coding RNA 2166)
VPS9D1-AS1 (VPS9D1 antisense RNA 1)
PITPNA-AS1 (PITPNA antisense RNA 1)
PRAL (p53 regulation associated lncRNA)
ALOX12-AS1 (ALOX12 antisense RNA 1)
FGF11 (fibroblast growth factor 11)
NAA38 (N-alpha-acetyltransferase 38, NatC auxiliary
CHD3 (chromodomain helicase DNA binding protein 3)
PIK3R5-DT (PIK3R5 divergent transcript)
TMEM238L (transmembrane protein 238 like)
DNAH9 (dynein axonemal heavy chain 9)
PIGL (phosphatidylinositol glycan anchor biosynthesis class

AOPEP (aminopeptidase O (putative))
PTRH1 (peptidyl-tRNA hydrolase 1 homolog)
COQ4 (coenzyme Q4)
ODF2-AS1 (ODF2 antisense RNA 1)
LINC00963 (long intergenic non-protein coding RNA 963)
ABO (ABO, alpha 1-3-N-acetylgalactosaminyltransferase and
CCDC187 (coiled-coil domain containing 187)
FAM166A (family with sequence similarity 166 member A)
MST1P2 (macrophage stimulating 1 pseudogene 2)
BCAS2P2 (BCAS2 pseudogene 2)
HYDIN2 (HYDIN axonemal central pair apparatus protein 2
FMO6P (flavin containing dimethylaniline monoxygenase 6,
ALG1L8P (ALG1 like 8, pseudogene)
OR7E36P (olfactory receptor family 7 subfamily E member
SORD2P (sorbitol dehydrogenase 2, pseudogene)
ABCC6P2 (ATP binding cassette subfamily C member 6
ABCC6P1 (ATP binding cassette subfamily C member 6
CES1P1 (carboxylesterase 1 pseudogene 1)
URAHP (urate (hydroxyiso-) hydrolase, pseudogene)
MEIS3P2 (Meis homeobox 3 pseudogene 2)
LRRC37A7P (leucine rich repeat containing 37 member A7,
CYP2F2P (cytochrome P450 family 2 subfamily F member 2,
CYP2G2P (cytochrome P450 family 2 subfamily G member 2,
MED15P9 (mediator complex subunit 15 pseudogene 9)
OR7E122P (olfactory receptor family 7 subfamily E member
TPRXL (tetrapeptide repeat homeobox like (pseudogene))
AADACP1 (arylacetamide deacetylase pseudogene 1)
KRT8P32 (keratin 8 pseudogene 32)
CMAHP (cytidine monophospho-N-acetylneuraminic acid
ZNF204P (zinc finger protein 204, pseudogene)
ALG1L5P (ALG1 like 5, pseudogene)
STAG3L3 (stromal antigen 3-like 3 (pseudogene))
LRRC37A5P (leucine rich repeat containing 37 member A5,

GNA15-DT (GNA15 divergent transcript)
LRG1 (leucine rich alpha-2-glycoprotein 1)
KDM4B (lysine demethylase 4B)
TINCR (TINCR ubiquitin domain containing)
SH2D3A (SH2 domain containing 3A)
STXBP2 (syntaxin binding protein 2)
HNRNPM (heterogeneous nuclear ribonucleoprotein M)
DNM2 (dynamin 2)
LDLR (low density lipoprotein receptor)
SPC24 (SPC24 component of NDC80 kinetochore complex)
HOOK2 (hook microtubule tethering protein 2)
JUNB (JunB proto-oncogene, AP-1 transcription factor
MIR23AHG (miR-23a/27a/24-2 cluster host gene)
DNAJB1 (DnaJ heat shock protein family (Hsp40) member
EPS15L1 (epidermal growth factor receptor pathway
MAST3-AS1 (MAST3 antisense RNA 1)
IFI30 (IFI30 lysosomal thiol reductase)
CRLF1 (cytokine receptor like factor 1)
ZNF493 (zinc finger protein 493)
NFKBID (NFKB inhibitor delta)
MAP4K1-AS1 (MAP4K1 antisense RNA 1)
HNRNPL (heterogeneous nuclear ribonucleoprotein L)
SAMD4B (sterile alpha motif domain containing 4B)
FCGBP (Fc fragment of IgG binding protein)
COQ8B (coenzyme Q8B)
TGFB1 (transforming growth factor beta 1)
LIPE-AS1 (LIPE antisense RNA 1)
CEACAM1 (CEA cell adhesion molecule 1)
CD177 (CD177 molecule)
NAPA-AS1 (NAPA antisense RNA 1)
NOP53-AS1 (NOP53 antisense RNA 1)
CARD8-AS1 (CARD8 antisense RNA 1)
PPP1R15A (protein phosphatase 1 regulatory subunit 15A)
NUCB1-AS1 (NUCB1 antisense RNA 1)
NOSIP (nitric oxide synthase interacting protein)
SIGLEC10-AS1 (SIGLEC10 antisense RNA 1)
FPR3 (formyl peptide receptor 3)
MBOAT7 (membrane bound O-acyltransferase domain
LENG9 (leukocyte receptor cluster member 9)
ID2 (inhibitor of DNA binding 2)
IAH1 (isoamyl acetate hydrolyzing esterase 1 (putative))
GRHL1 (grainyhead like transcription factor 1)
HPCAL1 (hippocalcin like 1)
ITSN2 (intersectin 2)
BABAM2 (BRISC and BRCA1 A complex member 2)
EML4-AS1 (EML4 antisense RNA 1)
LINC01890 (long intergenic non-protein coding RNA 1890)
PCBP1-AS1 (PCBP1 antisense RNA 1)
LINC01816 (long intergenic non-protein coding RNA 1816)
NAGK (N-acetylglucosamine kinase)
VAMP5 (vesicle associated membrane protein 5)
RNF103-CHMP3 (RNF103-CHMP3 readthrough)
CHMP3 (charged multivesicular body protein 3)
LINC01943 (long intergenic non-protein coding RNA 1943)
IGKC (immunoglobulin kappa constant)
LINC00342 (long intergenic non-protein coding RNA 342)
UBXN4 (UBX domain protein 4)
MBD5 (methyl-CpG binding domain protein 5)
MAP3K20-AS1 (MAP3K20 antisense RNA 1)
NABP1 (nucleic acid binding protein 1)
STK17B (serine/threonine kinase 17b)
ANKRD44 (ankyrin repeat domain 44)
BZW1-AS1 (BZW1 antisense RNA 1)
CFLAR (CASP8 and FADD like apoptosis regulator)
CASP8 (caspase 8)
CREB1 (cAMP responsive element binding protein 1)
CXCR2 (C-X-C motif chemokine receptor 2)
ARPC2 (actin related protein 2/3 complex subunit 2)
CTDSP1 (CTD small phosphatase 1)
SP110 (SP110 nuclear body protein)
SDCBP2 (syndecan binding protein 2)
RRBP1 (ribosome binding protein 1)
RALGAPA2 (Ral GTPase activating protein catalytic subunit
PLCG1-AS1 (PLCG1 antisense RNA 1)
STK4 (serine/threonine kinase 4)
SLC12A5-AS1 (SLC12A5 and MMP9 antisense RNA 1)
LINC01271 (long intergenic non-protein coding RNA 1271)
BCAS4 (breast carcinoma amplified sequence 4)
SLCO4A1-AS1 (SLCO4A1 antisense RNA 1)
IL10RB-DT (IL10RB divergent transcript)
DONSON (DNA replication fork stabilization factor DONSON)
KCNE1 (potassium voltage-gated channel subfamily E
CBR3 (carbonyl reductase 3)
MX2 (MX dynamin like GTPase 2)
ITGB2-AS1 (ITGB2 antisense RNA 1)
PRMT2 (protein arginine methyltransferase 2)
LINC00528 (long intergenic non-protein coding RNA 528)
PI4KA (phosphatidylinositol 4-kinase alpha)
MIF-AS1 (MIF antisense RNA 1)
ASCC2 (activating signal cointegrator 1 complex subunit 2)
TUG1 (taurine up-regulated 1)
HMOX1 (heme oxygenase 1)
NCF4-AS1 (NCF4 antisense RNA 1)
GGA1 (golgi associated, gamma adaptin ear containing, ARF
PDXP-DT (PDXP divergent transcript)
PLA2G6 (phospholipase A2 group VI)
CSNK1E (casein kinase 1 epsilon)
GTPBP1 (GTP binding protein 1)
APOBEC3B-AS1 (APOBEC3B antisense RNA 1)
GRAP2 (GRB2 related adaptor protein 2)
TYMP (thymidine phosphorylase)
KLHDC7B-DT (KLHDC7B divergent transcript)
BHLHE40-AS1 (BHLHE40 antisense RNA 1)
RAF1 (Raf-1 proto-oncogene, serine/threonine kinase)
SH3BP5-AS1 (SH3BP5 antisense RNA 1)
CMTM7 (CKLF like MARVEL transmembrane domain
LRRFIP2 (LRR binding FLII interacting protein 2)
EIF1B (eukaryotic translation initiation factor 1B)
LINC02158 (long intergenic non-protein coding RNA 2158)
SNRK-AS1 (SNRK antisense RNA 1)
CCR1 (C-C motif chemokine receptor 1)
CCR5AS (CCR5 antisense RNA)
CCR5 (C-C motif chemokine receptor 5)
ARIH2 (ariadne RBR E3 ubiquitin protein ligase 2)
SEMA3F-AS1 (SEMA3F antisense RNA 1)
RASSF1-AS1 (RASSF1 antisense RNA 1)
PSMD6-AS2 (PSMD6 antisense RNA 2)
PSMD6 (proteasome 26S subunit, non-ATPase 6)
FOXP1-IT1 (FOXP1 intronic transcript 1)
CBLB (Cbl proto-oncogene B)
SLC35A5 (solute carrier family 35 member A5)
HSPBAP1 (HSPB1 associated protein 1)
MGLL (monoglyceride lipase)
RUVBL1 (RuvB like AAA ATPase 1)
PLSCR1 (phospholipid scramblase 1)
MBNL1 (muscleblind like splicing regulator 1)
DHX36 (DEAH-box helicase 36)
LINC02067 (long intergenic non-protein coding RNA 2067)
B3GNT5 (UDP-GlcNAc:betaGal beta-1,3-N-
BCL6 (BCL6 transcription repressor)
LPP (LIM domain containing preferred translocation partner
UTS2B (urotensin 2B)
MUC4 (mucin 4, cell surface associated)
SLC51A (solute carrier family 51 subunit alpha)
GAK (cyclin G associated kinase)
BOD1L1 (biorientation of chromosomes in cell division 1
RHOH (ras homolog family member H)
RBM47 (RNA binding motif protein 47)
UBA6 (ubiquitin like modifier activating enzyme 6)
CXCL5 (C-X-C motif chemokine ligand 5)
RASGEF1B (RasGEF domain family member 1B)
UBE2D3 (ubiquitin conjugating enzyme E2 D3)
ALPK1 (alpha kinase 1)
NAA15 (N-alpha-acetyltransferase 15, NatA auxiliary
RAB33B-AS1 (RAB33B antisense RNA 1)
SPCS3-AS1 (SPCS3 antisense RNA 1)
IRF2-DT (IRF2 divergent transcript)
PDCD6 (programmed cell death 6)
GPBP1 (GC-rich promoter binding protein 1)
SREK1 (splicing regulatory glutamic acid and lysine rich
VCAN-AS1 (VCAN antisense RNA 1)
CCNH (cyclin H)
SRP19 (signal recognition particle 19)
FNIP1 (folliculin interacting protein 1)
IRF1-AS1 (IRF1 antisense RNA 1)
IRF1 (interferon regulatory factor 1)
MIR3661 (microRNA 3661)
MACROH2A1 (macroH2A.1 histone)
EGR1 (early growth response 1)
ARHGAP26 (Rho GTPase activating protein 26)
ADRB2 (adrenoceptor beta 2)
GRPEL2-AS1 (GRPEL2 antisense RNA 1)
TNIP1 (TNFAIP3 interacting protein 1)
FAXDC2 (fatty acid hydroxylase domain containing 2)
C5orf58 (chromosome 5 open reading frame 58)
PHYKPL (5-phosphohydroxy-L-lysine phospho-lyase)
MRNIP (MRN complex interacting protein)
LINC00847 (long intergenic non-protein coding RNA 847)
SERPINB1 (serpin family B member 1)
RREB1 (ras responsive element binding protein 1)
EDN1 (endothelin 1)
NOL7 (nucleolar protein 7)
ATXN1 (ataxin 1)
HLA-F (major histocompatibility complex, class I, F)
HLA-A (major histocompatibility complex, class I, A)
HCG18 (HLA complex group 18)
IER3-AS1 (IER3 antisense RNA 1)
HCG20 (HLA complex group 20 (non-protein coding))
TNF (tumor necrosis factor)
HLA-DQB1-AS1 (HLA-DQB1 antisense RNA 1)
ILRUN-AS1 (ILRUN antisense RNA 1)
FKBP1C (FKBP prolyl isomerase family member 1C)
PNRC1 (proline rich nuclear receptor coactivator 1)
PNISR (PNN interacting serine and arginine rich protein)
CALHM6-AS1 (CALHM6 antisense RNA 1)
WAKMAR2 (wound and keratinocyte migration associated
TNFAIP3 (TNF alpha induced protein 3)
SOD2 (superoxide dismutase 2)
IGF2R (insulin like growth factor 2 receptor)
AGPAT4 (1-acylglycerol-3-phosphate O-acyltransferase 4)
PSMB1 (proteasome 20S subunit beta 1)
C7orf50 (chromosome 7 open reading frame 50)
SNX13 (sorting nexin 13)
TRA2A (transformer 2 alpha homolog)
TAX1BP1 (Tax1 binding protein 1)
GARS1-DT (GARS1 divergent transcript)
AVL9 (AVL9 cell migration associated)
SEPTIN7 (septin 7)
TRGC2 (T cell receptor gamma constant 2)
CDK13 (cyclin dependent kinase 13)
DBNL (drebrin like)
POLM (DNA polymerase mu)
ERV3-1 (endogenous retrovirus group 3 member 1,
TYW1 (tRNA-yW synthesizing protein 1 homolog)
DTX2P1-UPK3BP1-PMS2P11 (DTX2P1-UPK3BP1-PMS2P11
STEAP4 (STEAP4 metalloreductase)
PDAP1 (PDGFA associated protein 1)
LAMTOR4 (late endosomal/lysosomal adaptor, MAPK and
POLR2J3 (RNA polymerase II subunit J3)
POLR2J2 (RNA polymerase II subunit J2)

CMTM4 (CKLF like MARVEL transmembrane domain
VAC14 (VAC14 component of PIKFYVE complex)
ADAT1 (adenosine deaminase tRNA specific 1)
DNAAF1 (dynein axonemal assembly factor 1)
ATP2C2-AS1 (ATP2C2 antisense RNA 1)
USP10 (ubiquitin specific peptidase 10)
CIBAR2 (CBY1 interacting BAR domain containing 2)
LINC02188 (long intergenic non-protein coding RNA 2188)
LINC02166 (long intergenic non-protein coding RNA 2166)
PITPNA-AS1 (PITPNA antisense RNA 1)
NAA38 (N-alpha-acetyltransferase 38, NatC auxiliary
DNAH9 (dynein axonemal heavy chain 9)
PIGL (phosphatidylinositol glycan anchor biosynthesis class
DRC3 (dynein regulatory complex subunit 3)
ATPAF2 (ATP synthase mitochondrial F1 complex assembly
B9D1 (B9 domain containing 1)
CCDC144NL-AS1 (CCDC144NL antisense RNA 1)
ERVE-1 (endogenous retrovirus group E member 1)
NF1 (neurofibromin 1)
OMG (oligodendrocyte myelin glycoprotein)
TADA2A (transcriptional adaptor 2A)
THRA (thyroid hormone receptor alpha)
SMARCE1 (SWI/SNF related, matrix associated, actin
NBR2 (neighbor of BRCA1 lncRNA 2)
CBX1 (chromobox 1)
ZNF652 (zinc finger protein 652)
MRPL27 (mitochondrial ribosomal protein L27)
ACSF2 (acyl-CoA synthetase family member 2)
ANKFN1 (ankyrin repeat and fibronectin type III domain
AKAP1-DT (AKAP1 divergent transcript)
LINC02875 (long intergenic non-protein coding RNA 2875)
TLK2 (tousled like kinase 2)
MARCHF10 (membrane associated ring-CH-type finger 10)
PRR29-AS1 (PRR29 antisense RNA 1)
ICAM2 (intercellular adhesion molecule 2)
LINC00511 (long intergenic non-protein coding RNA 511)
RECQL5 (RecQ like helicase 5)
BAIAP2-DT (BAIAP2 divergent transcript)
BAIAP2 (BAR/IMD domain containing adaptor protein 2)
MAFG-DT (MAFG divergent transcript)
CCDC57 (coiled-coil domain containing 57)
ENOSF1 (enolase superfamily member 1)
LINC00526 (long intergenic non-protein coding RNA 526)
TTC39C-AS1 (TTC39C antisense RNA 1)
KCTD1 (potassium channel tetramerization domain
AQP4-AS1 (AQP4 antisense RNA 1)
LINC01908 (long intergenic non-protein coding RNA 1908)
COSMOC (cell fate and sterol metabolism associated
TPGS2 (tubulin polyglutamylase complex subunit 2)
ZBTB7C (zinc finger and BTB domain containing 7C)
ELAC1 (elaC ribonuclease Z 1)
TXNL1 (thioredoxin like 1)
NEDD4L (NEDD4 like E3 ubiquitin protein ligase)
KDSR-DT (KDSR divergent transcript)
LINC00683 (long intergenic non-protein coding RNA 683)
PARD6G-AS1 (PARD6G antisense RNA 1)
PLPP2 (phospholipid phosphatase 2)
PSPN (persephin)
KHSRP (KH-type splicing regulatory protein)
C3 (complement C3)
ILF3-DT (ILF3 divergent transcript)
ZNF440 (zinc finger protein 440)
NFIX (nuclear factor I X)
NWD1 (NACHT and WD repeat domain containing 1)
UBA52 (ubiquitin A-52 residue ribosomal protein fusion
ZNF429 (zinc finger protein 429)
DPY19L3-DT (DPY19L3 divergent transcript)
CEP89 (centrosomal protein 89)
TBCB (tubulin folding cofactor B)
ZNF529-AS1 (ZNF529 antisense RNA 1)
ZNF529 (zinc finger protein 529)
SAMD4B (sterile alpha motif domain containing 4B)
ZNF780A (zinc finger protein 780A)
CYP2F1 (cytochrome P450 family 2 subfamily F member 1)
CEACAM6 (CEA cell adhesion molecule 6)
ZNF226 (zinc finger protein 226)
ERCC1 (ERCC excision repair 1, endonuclease non-catalytic
C5AR1 (complement C5a receptor 1)
MED25 (mediator complex subunit 25)
ZNF473 (zinc finger protein 473)
ZNF880 (zinc finger protein 880)
ZNF528-AS1 (ZNF528 antisense RNA 1)
ZNF677 (zinc finger protein 677)
DNAAF3-AS1 (DNAAF3 antisense RNA 1)
TRAPPC2B (trafficking protein particle complex subunit 2B)
ZNF135 (zinc finger protein 135)
SLC27A5 (solute carrier family 27 member 5)
MZF1-AS1 (MZF1 antisense RNA 1)
MZF1 (myeloid zinc finger 1)
FAM166C (family with sequence similarity 166 member C)
SLC5A6 (solute carrier family 5 member 6)
PPM1G (protein phosphatase, Mg2+/Mn2+ dependent 1G)
CAPN13 (calpain 13)
COX7A2L (cytochrome c oxidase subunit 7A2 like)
LINC01833 (long intergenic non-protein coding RNA 1833)
SIX3-AS1 (SIX3 antisense RNA 1)
RHOQ (ras homolog family member Q)
CALM2 (calmodulin 2)
PPP1R21-DT (PPP1R21 divergent transcript)
BCL11A (BAF chromatin remodeling complex subunit
PUS10 (pseudouridine synthase 10)
OTX1 (orthodenticle homeobox 1)
ANXA4 (annexin A4)
PARTICL (promoter of MAT2A antisense radiation-induced
EIF2AK3-DT (EIF2AK3 divergent transcript)
EIF2AK3 (eukaryotic translation initiation factor 2 alpha
ZNF514 (zinc finger protein 514)
LINC00342 (long intergenic non-protein coding RNA 342)
NEURL3 (neuralized E3 ubiquitin protein ligase 3)
CRACDL (CRACD like)
TSGA10 (testis specific 10)
TBC1D8-AS1 (TBC1D8 antisense RNA 1)
C2orf49-DT (C2orf49 divergent transcript)
CCDC138 (coiled-coil domain containing 138)
NPHP1 (nephrocystin 1)
LY75 (lymphocyte antigen 75)
TTC21B-AS1 (TTC21B antisense RNA 1)
METTL5 (methyltransferase 5, N6-adenosine)
ATP5MC3 (ATP synthase membrane subunit c locus 3)
AGPS (alkylglycerone phosphate synthase)
ANKRD44-AS1 (ANKRD44 antisense RNA 1)
HSPE1 (heat shock protein family E (Hsp10) member 1)
ALS2 (alsin Rho guanine nucleotide exchange factor ALS2)
CYP20A1 (cytochrome P450 family 20 subfamily A member
RAPH1 (Ras association (RalGDS/AF-6) and pleckstrin
LANCL1 (LanC like 1)
LINC01963 (long intergenic non-protein coding RNA 1963)
IGFBP2 (insulin like growth factor binding protein 2)
SH3BP4 (SH3 domain binding protein 4)
MLPH (melanophilin)
NDUFA10 (NADH:ubiquinone oxidoreductase subunit A10)
ANKMY1 (ankyrin repeat and MYND domain containing 1)
FARP2 (FERM, ARH/RhoGEF and pleckstrin domain protein
ING5 (inhibitor of growth family member 5)
DTD1 (D-aminoacyl-tRNA deacylase 1)
LINC00653 (long intergenic non-protein coding RNA 653)
BCL2L1 (BCL2 like 1)
BPIFB1 (BPI fold containing family B member 1)
UQCC1 (ubiquinol-cytochrome c reductase complex
CNBD2 (cyclic nucleotide binding domain containing 2)
NORAD (non-coding RNA activated by DNA damage)
BLCAP (BLCAP apoptosis inducing factor)
SLC2A10 (solute carrier family 2 member 10)
VAPB (VAMP associated protein B and C)
UCKL1-AS1 (UCKL1 antisense RNA 1)
URB1-AS1 (URB1 antisense RNA 1 (head to head))
LINC01436 (long intergenic non-protein coding RNA 1436)
LCA5L (lebercilin LCA5 like)
PLAC4 (placenta enriched 4)
UMODL1-AS1 (UMODL1 antisense RNA 1)
LINC01671 (long intergenic non-protein coding RNA 1671)
NDUFV3 (NADH:ubiquinone oxidoreductase subunit V3)
AATBC (apoptosis associated transcript in bladder cancer)
LINC00205 (long intergenic non-protein coding RNA 205)
UFD1 (ubiquitin recognition factor in ER associated
LINC01637 (long intergenic non-protein coding RNA 1637)
MAPK1 (mitogen-activated protein kinase 1)
C22orf15 (chromosome 22 open reading frame 15)
KIAA1671-AS1 (KIAA1671 antisense RNA 1)
TFIP11-DT (TFIP11 divergent transcript)
CCDC157 (coiled-coil domain containing 157)
RNF215 (ring finger protein 215)
SLC35E4 (solute carrier family 35 member E4)
DUSP18 (dual specificity phosphatase 18)
IFT27 (intraflagellar transport 27)
ANKRD54 (ankyrin repeat domain 54)
DDX17 (DEAD-box helicase 17)
NPTXR (neuronal pentraxin receptor)
XPNPEP3 (X-prolyl aminopeptidase 3)
EP300-AS1 (EP300 antisense RNA 1)
TCF20 (transcription factor 20)
TTLL1-AS1 (TTLL1 antisense RNA 1)
PRR34 (PRR34 long non-coding RNA)
RABL2B (RAB, member of RAS oncogene family like 2B)
CHL1-AS2 (CHL1 antisense RNA 2)
CHL1 (cell adhesion molecule L1 like)
CHL1-AS1 (CHL1 antisense RNA 1)
IL5RA (interleukin 5 receptor subunit alpha)
SRGAP3 (SLIT-ROBO Rho GTPase activating protein 3)
SYN2 (synapsin II)
ANKRD28 (ankyrin repeat domain 28)
NEK10 (NIMA related kinase 10)
CNOT10-AS1 (CNOT10 antisense RNA 1)
ACAA1 (acetyl-CoA acyltransferase 1)
ENTPD3-AS1 (ENTPD3 antisense RNA 1)
KIF9-AS1 (KIF9 antisense RNA 1)
ARIH2 (ariadne RBR E3 ubiquitin protein ligase 2)
RHOA (ras homolog family member A)
ZMYND10-AS1 (ZMYND10 antisense RNA 1)
GLT8D1 (glycosyltransferase 8 domain containing 1)
DNAH12 (dynein axonemal heavy chain 12)
FAM107A (family with sequence similarity 107 member A)
ZNF717 (zinc finger protein 717)
DCBLD2 (discoidin, CUB and LCCL domain containing 2)
NIT2 (nitrilase family member 2)
TBILA (TGF-beta induced lncRNA)
CD200R1L-AS1 (CD200R1L antisense RNA 1)
QTRT2 (queuine tRNA-ribosyltransferase accessory subunit
GSK3B (glycogen synthase kinase 3 beta)
ALDH1L1-AS1 (ALDH1L1 antisense RNA 1)
H1-10-AS1 (H1-10 antisense RNA 1)
SNORA7B (small nucleolar RNA, H/ACA box 7B)
EFCAB12 (EF-hand calcium binding domain 12)
COPB2 (COPI coat complex subunit beta 2)
HLTF-AS1 (HLTF antisense RNA 1)
ANKUB1 (ankyrin repeat and ubiquitin domain containing
SERP1 (stress associated endoplasmic reticulum protein 1)
TMEM212 (transmembrane protein 212)
LINC02031 (long intergenic non-protein coding RNA 2031)
ABCC5 (ATP binding cassette subfamily C member 5)

KCNQ1OT1 (KCNQ1 opposite strand/antisense transcript 1)
NAP1L4 (nucleosome assembly protein 1 like 4)
RHOG (ras homolog family member G)
STIM1-AS1 (STIM1 antisense RNA 1)
RRM1-AS1 (RRM1 antisense RNA 1)
FHIP1B (FHF complex subunit HOOK interacting protein 1B)
DENND2B (DENN domain containing 2B)
DENND5A (DENN domain containing 5A)
WEE1 (WEE1 G2 checkpoint kinase)
ZBED5 (zinc finger BED-type containing 5)
MICAL2 (microtubule associated monooxygenase, calponin
CYP2R1 (cytochrome P450 family 2 subfamily R member 1)
C11orf58 (chromosome 11 open reading frame 58)
NUCB2 (nucleobindin 2)
SAA2 (serum amyloid A2)
CSRP3-AS1 (CSRP3 and E2F8 antisense RNA 1)
NAV2-AS1 (NAV2 antisense RNA 1)
GAS2 (growth arrest specific 2)
RCN1 (reticulocalbin 1)
LINC00294 (long intergenic non-protein coding RNA 294)
CD59 (CD59 molecule (CD59 blood group))
CD44 (CD44 molecule (Indian blood group))
CD44-AS1 (CD44 antisense RNA 1)
PRDM11 (PR/SET domain 11)
PHF21A (PHD finger protein 21A)
DGKZ (diacylglycerol kinase zeta)
MDK (midkine)
FNBP4 (formin binding protein 4)
FAM111A-DT (FAM111A divergent transcript)
CD6 (CD6 molecule)
DDB1 (damage specific DNA binding protein 1)
TKFC (triokinase and FMN cyclase)
CYB561A3 (cytochrome b561 family member A3)
FEN1 (flap structure-specific endonuclease 1)
FADS1 (fatty acid desaturase 1)
ASRGL1 (asparaginase and isoaspartyl peptidase 1)
LBHD1 (LBH domain containing 1)
TMEM223 (transmembrane protein 223)
COX8A (cytochrome c oxidase subunit 8A)
PPP1R14B (protein phosphatase 1 regulatory inhibitor
GPR137 (G protein-coupled receptor 137)
TMEM262 (transmembrane protein 262)
MRPL49 (mitochondrial ribosomal protein L49)
NEAT1 (nuclear paraspeckle assembly transcript 1)
MAP3K11 (mitogen-activated protein kinase kinase kinase
RNASEH2C (ribonuclease H2 subunit C)
RBM4B (RNA binding motif protein 4B)
ANKRD13D (ankyrin repeat domain 13D)
RAD9A (RAD9 checkpoint clamp component A)
ALDH3B1 (aldehyde dehydrogenase 3 family member B1)
NDUFS8 (NADH:ubiquinone oxidoreductase core subunit
TCIRG1 (T cell immune regulator 1, ATPase H+ transporting
CCND1 (cyclin D1)
IL18BP (interleukin 18 binding protein)
ARAP1-AS1 (ARAP1 antisense RNA 1)
ATG16L2 (autophagy related 16 like 2)
P4HA3 (prolyl 4-hydroxylase subunit alpha 3)
PCF11-AS1 (PCF11 antisense RNA 1)
PRSS23 (serine protease 23)
ARHGAP42-AS1 (ARHGAP42 antisense RNA 1)
EXPH5 (exophilin 5)
COLCA1 (colorectal cancer associated 1)
PCSK7 (proprotein convertase subtilisin/kexin type 7)
BACE1-AS (BACE1 antisense RNA)
TMPRSS4 (transmembrane serine protease 4)
KMT2A (lysine methyltransferase 2A)
CENATAC-DT (CENATAC divergent transcript)
TRAPPC4 (trafficking protein particle complex subunit 4)
SORL1 (sortilin related receptor 1)
SIAE (sialic acid acetylesterase)
GSEC (G-quadruplex forming sequence containing lncRNA)
CRACR2A (calcium release activated channel regulator 2A)
AKAP3 (A-kinase anchoring protein 3)
SCNN1A (sodium channel epithelial 1 subunit alpha)
LTBR (lymphotoxin beta receptor)
TAPBPL (TAP binding protein like)
NCAPD2 (non-SMC condensin I complex subunit D2)
MLF2 (myeloid leukemia factor 2)
SPSB2 (splA/ryanodine receptor domain and SOCS box
MIR200CHG (MIR200C and MIR141 host gene)
C1RL-AS1 (C1RL antisense RNA 1)
MIR1244-3 (microRNA 1244-3)
LINC00987 (long intergenic non-protein coding RNA 987)
CD69 (CD69 molecule)
KLRK1-AS1 (KLRK1 antisense RNA 1)
MIR1244-4 (microRNA 1244-4)
HEBP1 (heme binding protein 1)
PLBD1-AS1 (PLBD1 antisense RNA 1)
H2AJ (H2A.J histone)
PLEKHA5 (pleckstrin homology domain containing A5)
FGFR1OP2 (FGFR1 oncogene partner 2)
OVCH1-AS1 (OVCH1 antisense RNA 1)
LINC02422 (long intergenic non-protein coding RNA 2422)
PKP2 (plakophilin 2)
SCAF11 (SR-related CTD associated factor 11)
HDAC7 (histone deacetylase 7)
FKBP11 (FKBP prolyl isomerase 11)
TUBA1B (tubulin alpha 1b)
TUBA1C (tubulin alpha 1c)
DAZAP2 (DAZ associated protein 2)
SMAGP (small cell adhesion glycoprotein)
KRT7 (keratin 7)
KRT8 (keratin 8)
KRT18 (keratin 18)
MYG1-AS1 (MYG1 antisense RNA 1)
PCBP2 (poly(rC) binding protein 2)
CBX5 (chromobox 5)
PMEL (premelanosome protein)
MYL6 (myosin light chain 6)
SMARCC2 (SWI/SNF related, matrix associated, actin
PTGES3 (prostaglandin E synthase 3)
STAT6 (signal transducer and activator of transcription 6)
MARS1 (methionyl-tRNA synthetase 1)
AGAP2-AS1 (AGAP2 antisense RNA 1)
RXYLT1-AS1 (RXYLT1 antisense RNA 1)
LLPH-DT (LLPH divergent transcript)
IRAK3 (interleukin 1 receptor associated kinase 3)
PRANCR (progenitor renewal associated non-coding RNA)
THAP2 (THAP domain containing 2)
CAPS2 (calcyphosine 2)
LRRIQ1 (leucine rich repeats and IQ motif containing 1)
ATP2B1-AS1 (ATP2B1 antisense RNA 1)
BTG1 (BTG anti-proliferation factor 1)
UBE2N (ubiquitin conjugating enzyme E2 N)
LTA4H (leukotriene A4 hydrolase)
TMPO-AS1 (TMPO antisense RNA 1)
IGF1 (insulin like growth factor 1)
C12orf73 (chromosome 12 open reading frame 73)
USP30-AS1 (USP30 antisense RNA 1)
MVK (mevalonate kinase)
TCHP (trichoplein keratin filament binding)
C12orf76 (chromosome 12 open reading frame 76)
ATXN2 (ataxin 2)
IQCD (IQ motif containing D)
CCDC60 (coiled-coil domain containing 60)
PXN-AS1 (PXN antisense RNA 1)
NRAV (negative regulator of antiviral response)
ANAPC5 (anaphase promoting complex subunit 5)
RNF34 (ring finger protein 34)
ORAI1 (ORAI calcium release-activated calcium modulator
CFAP251 (cilia and flagella associated protein 251)
SBNO1-AS1 (SBNO1 antisense RNA 1)
SNRNP35 (small nuclear ribonucleoprotein U11/U12
TMED2 (transmembrane p24 trafficking protein 2)
DNAH10 (dynein axonemal heavy chain 10)
DNAH10OS (dynein axonemal heavy chain 10 opposite
LINC02361 (long intergenic non-protein coding RNA 2361)
FBRSL1 (fibrosin like 1)
ZNF26 (zinc finger protein 26)
NHLRC3 (NHL repeat containing 3)
LINC00332 (long intergenic non-protein coding RNA 332)
ELF1 (E74 like ETS transcription factor 1)
VWA8-AS1 (VWA8 antisense RNA 1 (head to head))
DNAJC15 (DnaJ heat shock protein family (Hsp40) member
TPT1-AS1 (TPT1 antisense RNA 1)
CPB2-AS1 (CPB2 antisense RNA 1)
MED4-AS1 (MED4 antisense RNA 1)
DLEU2 (deleted in lymphocytic leukemia 2)
ALG11 (ALG11 alpha-1,2-mannosyltransferase)
NEK5 (NIMA related kinase 5)
LMO7-AS1 (LMO7 antisense RNA 1)
CLN5 (CLN5 intracellular trafficking protein)
FBXL3 (F-box and leucine rich repeat protein 3)
RBM26 (RNA binding motif protein 26)
SOX21-AS1 (SOX21 antisense divergent transcript 1)
FARP1 (FERM, ARH/RhoGEF and pleckstrin domain protein
FARP1-AS1 (FARP1 antisense RNA 1)
LINC00449 (long intergenic non-protein coding RNA 449)
PCCA-DT (PCCA divergent transcript)
FGF14-AS2 (FGF14 antisense RNA 2)
ARGLU1 (arginine and glutamate rich 1)
MYO16-AS1 (MYO16 antisense RNA 1)
COL4A2-AS1 (COL4A2 antisense RNA 1)
PRECSIT (p53 regulated carcinoma associated Stat3
ANKRD10-IT1 (ANKRD10 intronic transcript 1)
MCF2L-AS1 (MCF2L antisense RNA 1)
PCID2 (PCI domain containing 2)
GRTP1 (growth hormone regulated TBC protein 1)
GAS6-AS1 (GAS6 antisense RNA 1)
UPF3A (UPF3A regulator of nonsense mediated mRNA
ANG (angiogenin)
EGILA (EGFR interacting lncRNA)
LINC00641 (long intergenic non-protein coding RNA 641)
HNRNPC (heterogeneous nuclear ribonucleoprotein C)
TRDC (T cell receptor delta constant)
TRAC (T cell receptor alpha constant)
OXA1L (OXA1L mitochondrial inner membrane protein)
PRMT5-AS1 (PRMT5 antisense RNA 1)
ACIN1 (apoptotic chromatin condensation inducer 1)
BCL2L2 (BCL2 like 2)
ZFHX2 (zinc finger homeobox 2)
DHRS4-AS1 (DHRS4 antisense RNA 1)
TM9SF1 (transmembrane 9 superfamily member 1)
LINC02300 (long intergenic non-protein coding RNA 2300)
HEATR5A (HEAT repeat containing 5A)
FAM177A1 (family with sequence similarity 177 member
PRORP (protein only RNase P catalytic subunit)
PAX9 (paired box 9)
SLC25A21-AS1 (SLC25A21 antisense RNA 1)
TTC6 (tetratricopeptide repeat domain 6)
KLHL28 (kelch like family member 28)
RN7SL1 (RNA component of signal recognition particle 7SL1)
SAV1 (salvador family WW domain containing protein 1)
FRMD6-AS1 (FRMD6 antisense RNA 1)
AP5M1 (adaptor related protein complex 5 subunit mu 1)
PSMA3 (proteasome 20S subunit alpha 3)
PSMA3-AS1 (PSMA3 antisense RNA 1)
CCDC175 (coiled-coil domain containing 175)
PCNX4 (pecanex 4)
HIF1A-AS1 (HIF1A antisense RNA 1)

DRC3 (dynein regulatory complex subunit 3)
ATPAF2 (ATP synthase mitochondrial F1 complex assembly
B9D1 (B9 domain containing 1)
CCDC144NL-AS1 (CCDC144NL antisense RNA 1)
WSB1 (WD repeat and SOCS box containing 1)
RSKR (ribosomal protein S6 kinase related)
SSH2 (slingshot protein phosphatase 2)
NF1 (neurofibromin 1)
OMG (oligodendrocyte myelin glycoprotein)
ZNF207 (zinc finger protein 207)
CCL3-AS1 (CCL3 antisense RNA 1)
TADA2A (transcriptional adaptor 2A)
DUSP14 (dual specificity phosphatase 14)
ERBB2 (erb-b2 receptor tyrosine kinase 2)
PSMD3 (proteasome 26S subunit, non-ATPase 3)
THRA (thyroid hormone receptor alpha)
MSL1 (MSL complex subunit 1)
SMARCE1 (SWI/SNF related, matrix associated, actin
STAT3 (signal transducer and activator of transcription 3)
NBR2 (neighbor of BRCA1 lncRNA 2)
KPNB1-DT (KPNB1 divergent transcript)
CBX1 (chromobox 1)
ZNF652 (zinc finger protein 652)
TOB1-AS1 (TOB1 antisense RNA 1)
ANKFN1 (ankyrin repeat and fibronectin type III domain
MSI2 (musashi RNA binding protein 2)
YPEL2 (yippee like 2)
VMP1 (vacuole membrane protein 1)
LINC02875 (long intergenic non-protein coding RNA 2875)
TLK2 (tousled like kinase 2)
MARCHF10 (membrane associated ring-CH-type finger 10)
PRR29-AS1 (PRR29 antisense RNA 1)
ICAM2 (intercellular adhesion molecule 2)
KCNJ2-AS1 (KCNJ2 antisense RNA 1)
LINC00511 (long intergenic non-protein coding RNA 511)
GPRC5C (G protein-coupled receptor class C group 5
KCTD2 (potassium channel tetramerization domain
SUMO2 (small ubiquitin like modifier 2)
MIF4GD-DT (MIF4GD divergent transcript)
RECQL5 (RecQ like helicase 5)
H3-3B (H3.3 histone B)
ST6GALNAC1 (ST6 N-acetylgalactosaminide alpha-2,6-
SOCS3 (suppressor of cytokine signaling 3)
PGS1 (phosphatidylglycerophosphate synthase 1)
RNF213 (ring finger protein 213)
RNF213-AS1 (RNF213 antisense RNA 1)
BAIAP2-DT (BAIAP2 divergent transcript)
BAIAP2 (BAR/IMD domain containing adaptor protein 2)
AATK (apoptosis associated tyrosine kinase)
ALYREF (Aly/REF export factor)
MAFG-DT (MAFG divergent transcript)
CCDC57 (coiled-coil domain containing 57)
CSNK1D (casein kinase 1 delta)
ENOSF1 (enolase superfamily member 1)
MYL12A (myosin light chain 12A)
VAPA (VAMP associated protein A)
NPC1 (NPC intracellular cholesterol transporter 1)
TTC39C-AS1 (TTC39C antisense RNA 1)
KCTD1 (potassium channel tetramerization domain
AQP4-AS1 (AQP4 antisense RNA 1)
LINC01908 (long intergenic non-protein coding RNA 1908)
ZSCAN30 (zinc finger and SCAN domain containing 30)
TPGS2 (tubulin polyglutamylase complex subunit 2)
SNHG22 (small nucleolar RNA host gene 22)
ELAC1 (elaC ribonuclease Z 1)
TXNL1 (thioredoxin like 1)
NEDD4L (NEDD4 like E3 ubiquitin protein ligase)
KDSR-DT (KDSR divergent transcript)
CNDP2 (carnosine dipeptidase 2)
LINC00683 (long intergenic non-protein coding RNA 683)
PARD6G-AS1 (PARD6G antisense RNA 1)
PLPP2 (phospholipid phosphatase 2)
PTBP1 (polypyrimidine tract binding protein 1)
CIRBP-AS1 (CIRBP antisense RNA 1)
PWWP3A (PWWP domain containing 3A, DNA repair factor)
JSRP1 (junctional sarcoplasmic reticulum protein 1)
TINCR (TINCR ubiquitin domain containing)
FUT3 (fucosyltransferase 3 (Lewis blood group))
KHSRP (KH-type splicing regulatory protein)
C3 (complement C3)
STXBP2 (syntaxin binding protein 2)
SLC44A2 (solute carrier family 44 member 2)
ILF3-DT (ILF3 divergent transcript)
DNM2 (dynamin 2)
LDLR (low density lipoprotein receptor)
PRKCSH (protein kinase C substrate 80K-H)
ZNF440 (zinc finger protein 440)
HOOK2 (hook microtubule tethering protein 2)
JUNB (JunB proto-oncogene, AP-1 transcription factor
NFIX (nuclear factor I X)
SAMD1 (sterile alpha motif domain containing 1)
DNAJB1 (DnaJ heat shock protein family (Hsp40) member
C19orf44 (chromosome 19 open reading frame 44)
NWD1 (NACHT and WD repeat domain containing 1)
MAST3-AS1 (MAST3 antisense RNA 1)
IFI30 (IFI30 lysosomal thiol reductase)
ZNF506 (zinc finger protein 506)
ZNF429 (zinc finger protein 429)
DPY19L3-DT (DPY19L3 divergent transcript)
CEP89 (centrosomal protein 89)
TBCB (tubulin folding cofactor B)
ZNF529-AS1 (ZNF529 antisense RNA 1)
ZNF529 (zinc finger protein 529)
HNRNPL (heterogeneous nuclear ribonucleoprotein L)
SAMD4B (sterile alpha motif domain containing 4B)
FCGBP (Fc fragment of IgG binding protein)
ZNF780A (zinc finger protein 780A)
CYP2F1 (cytochrome P450 family 2 subfamily F member 1)
LIPE-AS1 (LIPE antisense RNA 1)
CD177 (CD177 molecule)
ZNF224 (zinc finger protein 224)
ZNF235 (zinc finger protein 235)
ERCC1 (ERCC excision repair 1, endonuclease non-catalytic
C5AR1 (complement C5a receptor 1)
CARD8-AS1 (CARD8 antisense RNA 1)
PPP1R15A (protein phosphatase 1 regulatory subunit 15A)
SNRNP70 (small nuclear ribonucleoprotein U1 subunit 70)
TRPM4 (transient receptor potential cation channel
MED25 (mediator complex subunit 25)
PTOV1 (PTOV1 extended AT-hook containing adaptor
ZNF473 (zinc finger protein 473)
SIGLEC10-AS1 (SIGLEC10 antisense RNA 1)
FPR3 (formyl peptide receptor 3)
ZNF880 (zinc finger protein 880)
ZNF528-AS1 (ZNF528 antisense RNA 1)
ZNF677 (zinc finger protein 677)
MBOAT7 (membrane bound O-acyltransferase domain
DNAAF3-AS1 (DNAAF3 antisense RNA 1)
TRAPPC2B (trafficking protein particle complex subunit 2B)
ZNF552 (zinc finger protein 552)
ZNF135 (zinc finger protein 135)
SLC27A5 (solute carrier family 27 member 5)
MZF1-AS1 (MZF1 antisense RNA 1)
MZF1 (myeloid zinc finger 1)
ID2 (inhibitor of DNA binding 2)
IAH1 (isoamyl acetate hydrolyzing esterase 1 (putative))
HPCAL1 (hippocalcin like 1)
LRATD1 (LRAT domain containing 1)
TTC32 (tetratricopeptide repeat domain 32)
LAPTM4A (lysosomal protein transmembrane 4 alpha)
ITSN2 (intersectin 2)
FAM166C (family with sequence similarity 166 member C)
SLC5A6 (solute carrier family 5 member 6)
PPM1G (protein phosphatase, Mg2+/Mn2+ dependent 1G)
BABAM2 (BRISC and BRCA1 A complex member 2)
PPP1CB (protein phosphatase 1 catalytic subunit beta)
CAPN13 (calpain 13)
EML4-AS1 (EML4 antisense RNA 1)
COX7A2L (cytochrome c oxidase subunit 7A2 like)
LINC01833 (long intergenic non-protein coding RNA 1833)
SIX3-AS1 (SIX3 antisense RNA 1)
RHOQ (ras homolog family member Q)
CALM2 (calmodulin 2)
FBXO11 (F-box protein 11)
PPP1R21-DT (PPP1R21 divergent transcript)
PPP4R3B (protein phosphatase 4 regulatory subunit 3B)
PPP4R3B-DT (PPP4R3B divergent transcript)
BCL11A (BAF chromatin remodeling complex subunit
XPO1 (exportin 1)
UGP2 (UDP-glucose pyrophosphorylase 2)
LINC01890 (long intergenic non-protein coding RNA 1890)
ANXA4 (annexin A4)
PCBP1-AS1 (PCBP1 antisense RNA 1)
PCBP1 (poly(rC) binding protein 1)
NAGK (N-acetylglucosamine kinase)
RNF103-CHMP3 (RNF103-CHMP3 readthrough)
LINC01943 (long intergenic non-protein coding RNA 1943)
EIF2AK3-DT (EIF2AK3 divergent transcript)
IGKC (immunoglobulin kappa constant)
ZNF514 (zinc finger protein 514)
LINC00342 (long intergenic non-protein coding RNA 342)
CRACDL (CRACD like)
TSGA10 (testis specific 10)
TBC1D8-AS1 (TBC1D8 antisense RNA 1)
C2orf49-DT (C2orf49 divergent transcript)
GCC2-AS1 (GCC2 antisense RNA 1)
CCDC138 (coiled-coil domain containing 138)
NPHP1 (nephrocystin 1)
UBXN4 (UBX domain protein 4)
MBD5 (methyl-CpG binding domain protein 5)
BAZ2B (bromodomain adjacent to zinc finger domain 2B)
LY75 (lymphocyte antigen 75)
TTC21B-AS1 (TTC21B antisense RNA 1)
METTL5 (methyltransferase 5, N6-adenosine)
MAP3K20-AS1 (MAP3K20 antisense RNA 1)
AGPS (alkylglycerone phosphate synthase)
ORMDL1 (ORMDL sphingolipid biosynthesis regulator 1)
NABP1 (nucleic acid binding protein 1)
STK17B (serine/threonine kinase 17b)
ANKRD44 (ankyrin repeat domain 44)
ANKRD44-AS1 (ANKRD44 antisense RNA 1)
HSPE1 (heat shock protein family E (Hsp10) member 1)
CFLAR (CASP8 and FADD like apoptosis regulator)
CASP8 (caspase 8)
CYP20A1 (cytochrome P450 family 20 subfamily A member
EEF1B2 (eukaryotic translation elongation factor 1 beta 2)
CREB1 (cAMP responsive element binding protein 1)
LANCL1 (LanC like 1)
LINC01963 (long intergenic non-protein coding RNA 1963)
IGFBP2 (insulin like growth factor binding protein 2)
CXCR2 (C-X-C motif chemokine receptor 2)
ARPC2 (actin related protein 2/3 complex subunit 2)
SP110 (SP110 nuclear body protein)
PDE6D (phosphodiesterase 6D)
SH3BP4 (SH3 domain binding protein 4)
MLPH (melanophilin)
NDUFA10 (NADH:ubiquinone oxidoreductase subunit A10)
ANKMY1 (ankyrin repeat and MYND domain containing 1)
FARP2 (FERM, ARH/RhoGEF and pleckstrin domain protein
ING5 (inhibitor of growth family member 5)

KMT2E-AS1 (KMT2E antisense RNA 1)
NAMPT (nicotinamide phosphoribosyltransferase)
SND1-IT1 (SND1 intronic transcript 1)
KCP (kielin cysteine rich BMP regulator)
CPA4 (carboxypeptidase A4)
LINC-PINT (long intergenic non-protein coding RNA, p53
CYREN (cell cycle regulator of NHEJ)
TBXAS1 (thromboxane A synthase 1)
TRBC1 (T cell receptor beta constant 1)
TRBC2 (T cell receptor beta constant 2)
EPHA1 (EPH receptor A1)
DNAJB6 (DnaJ heat shock protein family (Hsp40) member
SLC25A37 (solute carrier family 25 member 37)
PTK2B (protein tyrosine kinase 2 beta)
PLEKHA2 (pleckstrin homology domain containing A2)
SDCBP (syndecan binding protein)
ASPH (aspartate beta-hydroxylase)
LINC02605 (long intergenic non-protein coding RNA 2605)
CA2 (carbonic anhydrase 2)
YWHAZ (tyrosine 3-monooxygenase/tryptophan 5-
ENY2 (ENY2 transcription and export complex 2 subunit)
RAD21-AS1 (RAD21 antisense RNA 1)
WASHC5-AS1 (WASHC5 antisense RNA 1)
CASC11 (cancer susceptibility 11)
PHF20L1 (PHD finger protein 20 like 1)
EEF1D (eukaryotic translation elongation factor 1 delta)
PARP10 (poly(ADP-ribose) polymerase family member 10)
SHARPIN (SHANK associated RH domain interactor)
ZNF7 (zinc finger protein 7)
CDKN2B-AS1 (CDKN2B antisense RNA 1)
GLIPR2 (GLI pathogenesis related 2)
LINC01504 (long intergenic non-protein coding RNA 1504)
HSD17B3-AS1 (HSD17B3 antisense RNA 1)
SLC31A2 (solute carrier family 31 member 2)
PRPF4 (pre-mRNA processing factor 4)
B3GALT9 (beta-1,3-galactosyltransferase 9)
LCN2 (lipocalin 2)
FNBP1 (formin binding protein 1)
RXRA (retinoid X receptor alpha)
ASMTL-AS1 (ASMTL antisense RNA 1)
GK-AS1 (GK antisense RNA 1)
TAB3 (TGF-beta activated kinase 1 (MAP3K7) binding
MED14OS (MED14 opposite strand)
DDX3X (DEAD-box helicase 3 X-linked)
LINC02595 (long intergenic non-protein coding RNA 2595)
MSN (moesin)
PGK1 (phosphoglycerate kinase 1)
FTH1P1 (ferritin heavy chain 1 pseudogene 1)
TPI1P1 (triosephosphate isomerase 1 pseudogene 1)
NSRP1P1 (nuclear speckle splicing regulatory protein 1
GBP1P1 (guanylate binding protein 1 pseudogene 1)
NRBF2P3 (nuclear receptor binding factor 2 pseudogene 3)
NAP1L4P1 (nucleosome assembly protein 1 like 4
FCGR1CP (Fc fragment of IgG receptor Ic, pseudogene)
SPRR2C (small proline rich protein 2C (pseudogene))
PFN1P1 (profilin 1 pseudogene 1)
EIF1P3 (eukaryotic translation initiation factor 1
ACTBP11 (ACTB pseudogene 11)
FTH1P2 (ferritin heavy chain 1 pseudogene 2)
NAMPTP1 (nicotinamide phosphoribosyltransferase
ZNF37BP (zinc finger protein 37B, pseudogene)
CAP1P2 (CAP1 pseudogene 2)
PPIAP19 (peptidylprolyl isomerase A pseudogene 19)
LDHAP5 (lactate dehydrogenase A pseudogene 5)
MORF4L1P3 (mortality factor 4 like 1 pseudogene 3)
IFITM9P (interferon induced transmembrane protein 9
FTH1P16 (ferritin heavy chain 1 pseudogene 16)
ARPC3P3 (actin related protein 2/3 complex subunit 3
IFITM3P2 (IFITM3 pseudogene 2)
ATP6V1E1P3 (ATPase H+ transporting V1 subunit E1
RBMS1P1 (RNA binding motif single stranded interacting
CLIC1P1 (chloride intracellular channel 1 pseudogene 1)
FTH1P7 (ferritin heavy chain 1 pseudogene 7)
EEF1A1P3 (eukaryotic translation elongation factor 1 alpha
H3P36 (H3 histone pseudogene 36)
HMGN2P46 (high mobility group nucleosomal binding
RRN3P3 (RRN3 pseudogene 3)
SMG1P2 (SMG1 pseudogene 2)
TXNP4 (thioredoxin pseudogene 4)
KRT17P6 (keratin 17 pseudogene 6)
EIF1P5 (eukaryotic translation initiation factor 1
PTP4A2P1 (PTP4A2 pseudogene 1)
PLEKHM1P1 (pleckstrin homology and RUN domain
ATF4P3 (activating transcription factor 4 pseudogene 3)
PGK1P2 (phosphoglycerate kinase 1, pseudogene 2)
PRR13P5 (proline rich 13 pseudogene 5)
GAPDHP38 (glyceraldehyde 3 phosphate dehydrogenase
TMSB4XP2 (TMSB4X pseudogene 2)
EIF1P7 (eukaryotic translation initiation factor 1
FTH1P3 (ferritin heavy chain 1 pseudogene 3)
ARL14EPP1 (ARL14EP pseudogene 1)
PRELID1P6 (PRELID1 pseudogene 6)
LDHAP7 (lactate dehydrogenase A pseudogene 7)
FAM133DP (family with sequence similarity 133, member A
FTH1P20 (ferritin heavy chain 1 pseudogene 20)
DNAJB1P1 (DnaJ heat shock protein family (Hsp40) member
IMPDH1P10 (inosine monophosphate dehydrogenase 1
TPM3P8 (tropomyosin 3 pseudogene 8)
FTLP3 (ferritin light chain pseudogene 3)
UBE2D3P1 (ubiquitin conjugating enzyme E2 D3
ARPC3P1 (actin related protein 2/3 complex subunit 3
TMSB4XP6 (TMSB4X pseudogene 6)
CCDC74BP1 (coiled-coil domain containing 74B pseudogene
CRYBB2P1 (crystallin beta B2 pseudogene 1)
TPTEP2 (TPTE pseudogene 2)
RRP7BP (ribosomal RNA processing 7 homolog B,
FTH1P23 (ferritin heavy chain 1 pseudogene 23)
CAP1P1 (CAP1 pseudogene 1)
FTH1P4 (ferritin heavy chain 1 pseudogene 4)
BZW1P1 (basic leucine zipper and W2 domains 1
LRRFIP1P1 (LRR binding FLII interacting protein 1
RNA5SP151 (RNA, 5S ribosomal pseudogene 151)
IFITM3P1 (IFITM3 pseudogene 1)
GAPDHP60 (glyceraldehyde-3-phosphate dehydrogenase
TMSB4XP8 (TMSB4X pseudogene 8)
H3P14 (H3 histone pseudogene 14)
CEP170P1 (centrosomal protein 170 pseudogene 1)
SEPTIN7P14 (septin 7 pseudogene 14)
H3P16 (H3 histone pseudogene 16)
FTH1P10 (ferritin heavy chain 1 pseudogene 10)
NAIPP2 (NAIP pseudogene 2)
RAP1BL (RAP1B like (pseudogene))
ALDH7A1P1 (aldehyde dehydrogenase 7 family member A1
ACTBP2 (ACTB pseudogene 2)
MTRNR2L2 (MT-RNR2 like 2)
HNRNPKP1 (heterogeneous nuclear ribonucleoprotein K
GAPDHP40 (glyceraldehyde 3 phosphate dehydrogenase
HTATSF1P2 (HIV-1 Tat specific factor 1 pseudogene 2)
ZNRD1ASP (zinc ribbon domain containing 1 antisense,
HLA-L (major histocompatibility complex, class I, L
FTH1P5 (ferritin heavy chain 1 pseudogene 5)
FTH1P15 (ferritin heavy chain 1 pseudogene 15)
LYPLA1P3 (LYPLA1 pseudogene 3)
GAPDHP63 (glyceraldehyde 3 phosphate dehydrogenase
TPT1P6 (tumor protein, translationally-controlled 1
TPI1P3 (triosephosphate isomerase 1 pseudogene 3)
TPT1P4 (tumor protein, translationally-controlled 1
S100A11P2 (S100A11 pseudogene 2)
IFITM3P4 (IFITM3 pseudogene 4)
GTF2IRD1P1 (GTF2I repeat domain containing 1
NCF1C (neutrophil cytosolic factor 1C pseudogene)
FTH1P11 (ferritin heavy chain 1 pseudogene 11)
SMPD5 (sphingomyelin phosphodiesterase 5 (pseudogene))
CLCN3P1 (chloride voltage-gated channel 3 pseudogene 1)
FTH1P12 (ferritin heavy chain 1 pseudogene 12)
GLULP4 (glutamate-ammonia ligase pseudogene 4)
EEF1DP2 (eukaryotic translation elongation factor 1 delta
TPT1P9 (tumor protein, translationally-controlled 1
TMSB4XP4 (TMSB4X pseudogene 4)
FTLP2 (ferritin light chain pseudogene 2)
GAPDHP1 (glyceraldehyde-3-phosphate dehydrogenase
SDCBPP3 (syndecan binding protein pseudogene 3)
GAPDHP65 (glyceraldehyde 3 phosphate dehydrogenase
H3P44 (H3 histone pseudogene 44)
PGK1P1 (phosphoglycerate kinase 1 pseudogene 1)
GK4P (glycerol kinase 4 pseudogene)
H3P47 (H3 histone pseudogene 47)
FTH1P8 (ferritin heavy chain 1 pseudogene 8)
IKBKGP1 (inhibitor of nuclear factor kappa B kinase subunit

TMEM41A (transmembrane protein 41A)
EIF4A2 (eukaryotic translation initiation factor 4A2)
TPRG1-AS1 (TPRG1 antisense RNA 1)
MUC20-OT1 (MUC20 overlapping transcript)
MUC20 (mucin 20, cell surface associated)
PCYT1A (phosphate cytidylyltransferase 1A, choline)
PIGX (phosphatidylinositol glycan anchor biosynthesis class
LMLN-AS1 (LMLN antisense RNA 1)
SPON2 (spondin 2)
CTBP1-DT (CTBP1 divergent transcript)
MXD4 (MAX dimerization protein 4)
LINC02481 (long intergenic non-protein coding RNA 2481)
MRFAP1L1 (Morf4 family associated protein 1 like 1)
TADA2B (transcriptional adaptor 2B)
TAPT1-AS1 (TAPT1 antisense RNA 1 (head to head))
SEPSECS-AS1 (SEPSECS antisense RNA 1 (head to head))
STIM2 (stromal interaction molecule 2)
PCDH7 (protocadherin 7)
DTHD1 (death domain containing 1)
UGDH (UDP-glucose 6-dehydrogenase)
UGDH-AS1 (UGDH antisense RNA 1)
LINC02265 (long intergenic non-protein coding RNA 2265)
SGCB (sarcoglycan beta)
SULT1E1 (sulfotransferase family 1E member 1)
SHROOM3-AS1 (SHROOM3 antisense RNA 1)
BMP2K (BMP2 inducible kinase)
PLAC8 (placenta associated 8)
MRPS18C (mitochondrial ribosomal protein S18C)
MAPK10 (mitogen-activated protein kinase 10)
TIGD2 (tigger transposable element derived 2)
BMPR1B-DT (BMPR1B divergent transcript)
ADH5 (alcohol dehydrogenase 5 (class III), chi polypeptide)
TRMT10A (tRNA methyltransferase 10A)
CXXC4 (CXXC finger protein 4)
SNHG8 (small nucleolar RNA host gene 8)
SETD7 (SET domain containing 7, histone lysine
MIR4453HG (MIR4453 host gene)
CEP72 (centrosomal protein 72)
TPPP (tubulin polymerization promoting protein)
TENT4A (terminal nucleotidyltransferase 4A)
SEMA5A (semaphorin 5A)
LINC01513 (long intergenic non-protein coding RNA 1513)
GOLPH3-DT (GOLPH3 divergent transcript)
SUB1 (SUB1 regulator of transcription)
TTC23L-AS1 (TTC23L antisense RNA 1)
NIPBL-DT (NIPBL divergent transcript)
LIFR (LIF receptor subunit alpha)
NNT-AS1 (NNT antisense RNA 1)
MRPS30 (mitochondrial ribosomal protein S30)
MAST4-AS1 (MAST4 antisense RNA 1)
MARVELD2 (MARVEL domain containing 2)
BTF3-DT (BTF3 divergent transcript)
ARHGEF28 (Rho guanine nucleotide exchange factor 28)
LINC02122 (long intergenic non-protein coding RNA 2122)
NSA2 (NSA2 ribosome biogenesis factor)
ARRDC3 (arrestin domain containing 3)
LINC01023 (long intergenic non-protein coding RNA 1023)
FER (FER tyrosine kinase)
P4HA2-AS1 (P4HA2 antisense RNA 1)
PPP2CA-DT (PPP2CA divergent transcript)
LINC01843 (long intergenic non-protein coding RNA 1843)
PKD2L2 (polycystin 2 like 2, transient receptor potential
SNHG4 (small nucleolar RNA host gene 4)
IK (IK cytokine)
WDR55 (WD repeat domain 55)
DELE1 (DAP3 binding cell death enhancer 1)
ATOX1 (antioxidant 1 copper chaperone)
G3BP1 (G3BP stress granule assembly factor 1)
FABP6-AS1 (FABP6 antisense RNA 1)
ZNF354A (zinc finger protein 354A)
RNF130 (ring finger protein 130)
FOXC1 (forkhead box C1)
SLC22A23 (solute carrier family 22 member 23)
H2AC4 (H2A clustered histone 4)
HCG11 (HLA complex group 11)
ZNF165 (zinc finger protein 165)
HCG14 (HLA complex group 14)
TRIM31-AS1 (TRIM31 antisense RNA 1)
DHX16 (DEAH-box helicase 16)
SAPCD1-AS1 (SAPCD1 antisense RNA 1)
SLC44A4 (solute carrier family 44 member 4)
HLA-DOA (major histocompatibility complex, class II, DO
PFDN6 (prefoldin subunit 6)
KIF6 (kinesin family member 6)
GNMT (glycine N-methyltransferase)
ANKRD66 (ankyrin repeat domain 66)
CD2AP-DT (CD2AP divergent transcript)
GCLC (glutamate-cysteine ligase catalytic subunit)
DST (dystonin)
SCAT8 (S-phase cancer associated transcript 8)
HMGN3 (high mobility group nucleosomal binding domain
HMGN3-AS1 (HMGN3 antisense RNA 1)
DOP1A (DOP1 leucine zipper like protein A)
MANEA-DT (MANEA divergent transcript)
PEX7 (peroxisomal biogenesis factor 7)
UTRN (utrophin)
LRP11 (LDL receptor related protein 11)
RAET1E-AS1 (RAET1E antisense RNA 1)
MIR3918 (microRNA 3918)
RSPH3 (radial spoke head 3)
RSPH10B (radial spoke head 10 homolog B)
MIOS-DT (MIOS divergent transcript)
TSPAN13 (tetraspanin 13)
AGR2 (anterior gradient 2, protein disulphide isomerase
LINC02888 (long intergenic non-protein coding RNA 2888)
SP8 (Sp8 transcription factor)
PRR15 (proline rich 15)
LINC00997 (long intergenic non-protein coding RNA 997)
HERPUD2-AS1 (HERPUD2 antisense RNA 1)
POLR2J4 (RNA polymerase II subunit J4, pseudogene)
PPIA (peptidylprolyl isomerase A)
ABCA13 (ATP binding cassette subfamily A member 13)
ZNF273 (zinc finger protein 273)
LINC00174 (long intergenic non-protein coding RNA 174)
LINC02604 (long intergenic non-protein coding RNA 2604)
AUTS2 (activator of transcription and developmental
BUD23 (BUD23 rRNA methyltransferase and ribosome
CLDN3 (claudin 3)
ELN-AS1 (ELN antisense RNA 1)
GTF2IRD1 (GTF2I repeat domain containing 1)
MDH2 (malate dehydrogenase 2)
DDX3ILA1 (DDX3 interacting lncRNA 1)
SRI-AS1 (SRI antisense RNA 1)
AKAP9 (A-kinase anchoring protein 9)
GNGT1 (G protein subunit gamma transducin 1)
GNG11 (G protein subunit gamma 11)
SPDYE3 (speedy/RINGO cell cycle regulator family member
PCOLCE-AS1 (PCOLCE antisense RNA 1)
MUC12 (mucin 12, cell surface associated)
EMSLR (E2F1 mRNA stabilizing lncRNA)
EFCAB10 (EF-hand calcium binding domain 10)
FEZF1-AS1 (FEZF1 antisense RNA 1)
FEZF1 (FEZ family zinc finger 1)
CHCHD3 (coiled-coil-helix-coiled-coil-helix domain
LRGUK (leucine rich repeats and guanylate kinase domain
AGBL3 (AGBL carboxypeptidase 3)
KDM7A-DT (KDM7A divergent transcript)
MGAM (maltase-glucoamylase)
REPIN1-AS1 (REPIN1 antisense RNA 1)
WDR86-AS1 (WDR86 antisense RNA 1)
PRKAG2-AS1 (PRKAG2 antisense RNA 1)
LINC01003 (long intergenic non-protein coding RNA 1003)
PAXIP1-DT (PAXIP1 divergent transcript)
NAT1 (N-acetyltransferase 1)
BNIP3L (BCL2 interacting protein 3 like)
HMBOX1 (homeobox containing 1)
LINC00589 (long intergenic non-protein coding RNA 589)
NSD3 (nuclear receptor binding SET domain protein 3)
RP1 (RP1 axonemal microtubule associated)
YTHDF3-DT (YTHDF3 divergent transcript)
IL7 (interleukin 7)
TMEM67 (transmembrane protein 67)
LINC02894 (long intergenic non-protein coding RNA 2894)
EIF3H (eukaryotic translation initiation factor 3 subunit H)
SAMD12 (sterile alpha motif domain containing 12)
NDUFB9 (NADH:ubiquinone oxidoreductase subunit B9)
LY6E-DT (LY6E divergent transcript)
RHPN1-AS1 (RHPN1 antisense RNA 1 (head to head))
IQANK1 (IQ motif and ankyrin repeat containing 1)
RFX3-AS1 (RFX3 antisense RNA 1)
GLIS3-AS1 (GLIS3 antisense RNA 1)
ACO1 (aconitase 1)
BAG1 (BAG cochaperone 1)
ZBTB5 (zinc finger and BTB domain containing 5)
C9orf85 (chromosome 9 open reading frame 85)
ALDH1A1 (aldehyde dehydrogenase 1 family member A1)
ANXA1 (annexin A1)
NTRK2 (neurotrophic receptor tyrosine kinase 2)
AOPEP (aminopeptidase O (putative))
MFSD14C (major facilitator superfamily domain containing
SLC31A1 (solute carrier family 31 member 1)
STRBP (spermatid perinuclear RNA binding protein)
PTRH1 (peptidyl-tRNA hydrolase 1 homolog)
AK1 (adenylate kinase 1)
COQ4 (coenzyme Q4)
ODF2-AS1 (ODF2 antisense RNA 1)
LINC00963 (long intergenic non-protein coding RNA 963)
ABO (ABO, alpha 1-3-N-acetylgalactosaminyltransferase and
CCDC187 (coiled-coil domain containing 187)
CCDC183-AS1 (CCDC183 antisense RNA 1)
MAN1B1-DT (MAN1B1 divergent transcript)
FAM166A (family with sequence similarity 166 member A)
ARSD-AS1 (ARSD antisense RNA 1)
INE2 (inactivation escape 2)
CASK-AS1 (CASK antisense RNA 1)
USP27X-DT (USP27X divergent transcript)
LINC01278 (long intergenic non-protein coding RNA 1278)
OGT (O-linked N-acetylglucosamine (GlcNAc) transferase)
JPX (JPX transcript, XIST activator)
SH3BGRL (SH3 domain binding glutamate rich protein like)
NDUFA1 (NADH:ubiquinone oxidoreductase subunit A1)
MTMR1 (myotubularin related protein 1)
MTND2P28 (MT-ND2 pseudogene 28)
MST1P2 (macrophage stimulating 1 pseudogene 2)
KRT8P47 (keratin 8 pseudogene 47)
CTBP2P8 (CTBP2 pseudogene 8)
SEC63P1 (SEC63 homolog, protein translocation regulator
BCAS2P2 (BCAS2 pseudogene 2)
PDE4DIPP2 (PDE4DIP pseudogene 2)
EMBP1 (embigin pseudogene 1)
HYDIN2 (HYDIN axonemal central pair apparatus protein 2
H2BC19P (H2B clustered histone 19, pseudogene)
H2BC20P (H2B clustered histone 20, pseudogene)
KRT8P45 (keratin 8 pseudogene 45)
FMO6P (flavin containing dimethylaniline monoxygenase 6,
CRYZL2P (crystallin zeta like 2, pseudogene)
MORF4L1P1 (mortality factor 4 like 1 pseudogene 1)
GTF2IP20 (general transcription factor IIi pseudogene 20)
ZNF731P (zinc finger protein 731, pseudogene)
KRT8P37 (keratin 8 pseudogene 37)

ZBTB25 (zinc finger and BTB domain containing 25)
FUT8-AS1 (FUT8 antisense RNA 1)
FAM71D (family with sequence similarity 71 member D)
EIF2S1 (eukaryotic translation initiation factor 2 subunit
ACTN1 (actinin alpha 1)
SLC39A9 (solute carrier family 39 member 9)
RBM25 (RNA binding motif protein 25)
PTGR2 (prostaglandin reductase 2)
ENTPD5 (ectonucleoside triphosphate diphosphohydrolase
YLPM1 (YLP motif containing 1)
FOS (Fos proto-oncogene, AP-1 transcription factor subunit)
GPATCH2L (G-patch domain containing 2 like)
VASH1-AS1 (VASH1 antisense RNA 1)
SURP (SRA stem-loop interacting RNA binding protein)
FOXN3-AS1 (FOXN3 antisense RNA 1)
DGLUCY (D-glutamate cyclase)
PPP4R3A (protein phosphatase 4 regulatory subunit 3A)
GLRX5 (glutaredoxin 5)
PAPOLA-DT (PAPOLA divergent transcript)
EVL (Enah/Vasp-like)
LINC00239 (long intergenic non-protein coding RNA 239)
PPP2R5C (protein phosphatase 2 regulatory subunit
AMN (amnion associated transmembrane protein)
MARK3 (microtubule affinity regulating kinase 3)
SIVA1 (SIVA1 apoptosis inducing factor)
CLBA1 (clathrin binding box of aftiphilin containing 1)
NDN (necdin, MAGE family member)
SNHG14 (small nucleolar RNA host gene 14)
KLF13 (Kruppel like factor 13)
LINC02256 (long intergenic non-protein coding RNA 2256)
LINC02345 (long intergenic non-protein coding RNA 2345)
THBS1-AS1 (THBS1 antisense RNA 1)
OIP5-AS1 (OIP5 antisense RNA 1)
GANC (glucosidase alpha, neutral C)
MAP1A (microtubule associated protein 1A)
PDIA3 (protein disulfide isomerase family A member 3)
MIR1282 (microRNA 1282)
PATL2 (PAT1 homolog 2)
B2M (beta-2-microglobulin)
TRIM69 (tripartite motif containing 69)
DUOX1 (dual oxidase 1)
ATP8B4 (ATPase phospholipid transporting 8B4 (putative))
GABPB1-IT1 (GABPB1 intronic transcript)
GABPB1-AS1 (GABPB1 antisense RNA 1)
USP50 (ubiquitin specific peptidase 50)
FAM214A (family with sequence similarity 214 member A)
PIGB (phosphatidylinositol glycan anchor biosynthesis class
TEX9 (testis expressed 9)
ALDH1A2 (aldehyde dehydrogenase 1 family member A2)
MYO1E (myosin IE)
ANXA2 (annexin A2)
LINC02568 (long intergenic non-protein coding RNA 2568)
OAZ2 (ornithine decarboxylase antizyme 2)
GLCE (glucuronic acid epimerase)
PKM (pyruvate kinase M1/2)
ADPGK (ADP dependent glucokinase)
ADPGK-AS1 (ADPGK antisense RNA 1)
NPTN-IT1 (NPTN intronic transcript 1)
CCDC33 (coiled-coil domain containing 33)
CLK3 (CDC like kinase 3)
C15orf39 (chromosome 15 open reading frame 39)
DNAJA4 (DnaJ heat shock protein family (Hsp40) member
ST20-AS1 (ST20 antisense RNA 1)
GOLGA6L9 (golgin A6 family like 9)
HOMER2 (homer scaffold protein 2)
C15orf40 (chromosome 15 open reading frame 40)
HDGFL3 (HDGF like 3)
SEC11A (SEC11 homolog A, signal peptidase complex
WDR93 (WD repeat domain 93)
AP3S2 (adaptor related protein complex 3 subunit sigma 2)
ZNF710-AS1 (ZNF710 antisense RNA 1)
NGRN (neugrin, neurite outgrowth associated)
UNC45A (unc-45 myosin chaperone A)
RCCD1-AS1 (RCCD1 and UNC45A antisense RNA 1)
PRC1-AS1 (PRC1 antisense RNA 1)
CHASERR (CHD2 adjacent suppressive regulatory RNA)
SNRNP25 (small nuclear ribonucleoprotein U11/U12
SNHG19 (small nucleolar RNA host gene 19)
SRRM2 (serine/arginine repetitive matrix 2)
ZG16B (zymogen granule protein 16B)
PKMYT1 (protein kinase, membrane associated
MMP25-AS1 (MMP25 antisense RNA 1)
LINC00921 (long intergenic non-protein coding RNA 921)
ZNF263 (zinc finger protein 263)
DNASE1 (deoxyribonuclease 1)
UBN1 (ubinuclein 1)
ABAT (4-aminobutyrate aminotransferase)
PMM2 (phosphomannomutase 2)
C16orf72 (chromosome 16 open reading frame 72)
CIITA (class II major histocompatibility complex
SOCS1 (suppressor of cytokine signaling 1)
LINC02185 (long intergenic non-protein coding RNA 2185)
MRTFB (myocardin related transcription factor B)
NDE1 (nudE neurodevelopment protein 1)
COQ7 (coenzyme Q7, hydroxylase)
TMC5 (transmembrane channel like 5)
DNAH3 (dynein axonemal heavy chain 3)
METTL9 (methyltransferase like 9)
POLR3E (RNA polymerase III subunit E)
RN7SKP23 (RN7SK pseudogene 23)
ERN2 (endoplasmic reticulum to nucleus signaling 2)
IL4R (interleukin 4 receptor)
SULT1A1 (sulfotransferase family 1A member 1)
MVP (major vault protein)
INO80E (INO80 complex subunit E)
CD2BP2-DT (CD2BP2 divergent transcript)
ZNF771 (zinc finger protein 771)
RNF40 (ring finger protein 40)
BCL7C (BAF chromatin remodeling complex subunit BCL7C)
FUS (FUS RNA binding protein)
PYCARD-AS1 (PYCARD antisense RNA 1)
PYCARD (PYD and CARD domain containing)
ITGAX (integrin subunit alpha X)
ARMC5 (armadillo repeat containing 5)
SLC5A2 (solute carrier family 5 member 2)
ZNF720 (zinc finger protein 720)
ITFG1 (integrin alpha FG-GAP repeat containing 1)
ABCC11 (ATP binding cassette subfamily C member 11)
BRD7 (bromodomain containing 7)
CYLD-AS1 (CYLD antisense RNA 1)
HNRNPA1P48 (heterogeneous nuclear ribonucleoprotein A1
CHD9 (chromodomain helicase DNA binding protein 9)
FTO (FTO alpha-ketoglutarate dependent dioxygenase)
MT1F (metallothionein 1F)
PLLP (plasmolipin)
POLR2C (RNA polymerase II subunit C)
LINC00920 (long intergenic non-protein coding RNA 920)
CMTM4 (CKLF like MARVEL transmembrane domain
DYNC1LI2 (dynein cytoplasmic 1 light intermediate chain 2)
ATP6V0D1 (ATPase H+ transporting V0 subunit d1)
TSNAXIP1 (translin associated factor X interacting protein 1)
CTRL (chymotrypsin like)
COG8 (component of oligomeric golgi complex 8)
NIP7 (nucleolar pre-rRNA processing protein NIP7)
TERF2 (telomeric repeat binding factor 2)
NFAT5 (nuclear factor of activated T cells 5)
NQO1-DT (NQO1 divergent transcript)
VAC14 (VAC14 component of PIKFYVE complex)
ZNF821 (zinc finger protein 821)
PMFBP1 (polyamine modulated factor 1 binding protein 1)
ADAT1 (adenosine deaminase tRNA specific 1)
DNAAF1 (dynein axonemal assembly factor 1)
ATP2C2-AS1 (ATP2C2 antisense RNA 1)
KLHL36 (kelch like family member 36)
USP10 (ubiquitin specific peptidase 10)
CIBAR2 (CBY1 interacting BAR domain containing 2)
COX4I1 (cytochrome c oxidase subunit 4I1)
LINC02188 (long intergenic non-protein coding RNA 2188)
C16orf95-DT (C16orf95 divergent transcript)
ACSF3 (acyl-CoA synthetase family member 3)
ANKRD11 (ankyrin repeat domain 11)
SPG7 (SPG7 matrix AAA peptidase subunit, paraplegin)
LINC02166 (long intergenic non-protein coding RNA 2166)
VPS9D1-AS1 (VPS9D1 antisense RNA 1)
ZNF276 (zinc finger protein 276)
GLOD4 (glyoxalase domain containing 4)
PITPNA-AS1 (PITPNA antisense RNA 1)
PITPNA (phosphatidylinositol transfer protein alpha)
P2RX5-TAX1BP3 (P2RX5-TAX1BP3 readthrough (NMD
ENO3 (enolase 3)
NUP88 (nucleoporin 88)
DERL2 (derlin 2)
PRAL (p53 regulation associated lncRNA)
MIR497HG (mir-497-195 cluster host gene)
GPS2 (G protein pathway suppressor 2)
FGF11 (fibroblast growth factor 11)
EIF4A1 (eukaryotic translation initiation factor 4A1)
NAA38 (N-alpha-acetyltransferase 38, NatC auxiliary
CHD3 (chromodomain helicase DNA binding protein 3)
LINC00324 (long intergenic non-protein coding RNA 324)
NDEL1 (nudE neurodevelopment protein 1 like 1)
PIK3R5-DT (PIK3R5 divergent transcript)
TMEM238L (transmembrane protein 238 like)
DNAH9 (dynein axonemal heavy chain 9)
NCOR1 (nuclear receptor corepressor 1)
PIGL (phosphatidylinositol glycan anchor biosynthesis class
FLCN (folliculin)
DRC3 (dynein regulatory complex subunit 3)
ATPAF2 (ATP synthase mitochondrial F1 complex assembly
B9D1 (B9 domain containing 1)
CCDC144NL-AS1 (CCDC144NL antisense RNA 1)
DHRS7B (dehydrogenase/reductase 7B)
WSB1 (WD repeat and SOCS box containing 1)
ERVE-1 (endogenous retrovirus group E member 1)
RSKR (ribosomal protein S6 kinase related)
FAM222B (family with sequence similarity 222 member B)
SSH2 (slingshot protein phosphatase 2)
NF1 (neurofibromin 1)
OMG (oligodendrocyte myelin glycoprotein)
LIG3 (DNA ligase 3)
CCL3-AS1 (CCL3 antisense RNA 1)
ZNHIT3 (zinc finger HIT-type containing 3)
GGNBP2 (gametogenetin binding protein 2)
TADA2A (transcriptional adaptor 2A)
PSMD3 (proteasome 26S subunit, non-ATPase 3)
THRA (thyroid hormone receptor alpha)
MSL1 (MSL complex subunit 1)
RARA-AS1 (RARA antisense RNA 1)
SMARCE1 (SWI/SNF related, matrix associated, actin
EIF1 (eukaryotic translation initiation factor 1)
STAT3 (signal transducer and activator of transcription 3)
HSD17B1-AS1 (HSD17B1 antisense RNA 1)
RETREG3 (reticulophagy regulator family member 3)
NBR2 (neighbor of BRCA1 lncRNA 2)
MEIOC (meiosis specific with coiled-coil domain)
CCDC103 (coiled-coil domain containing 103)
MAP3K14-AS1 (MAP3K14 antisense RNA 1)
LRRC37A2 (leucine rich repeat containing 37 member A2)
FAM215B (family with sequence similarity 215 member B)
SP2-DT (SP2 divergent transcript)

UBOX5-AS1 (UBOX5 antisense RNA 1)
SNX5 (sorting nexin 5)
DTD1 (D-aminoacyl-tRNA deacylase 1)
LINC00653 (long intergenic non-protein coding RNA 653)
RALGAPA2 (Ral GTPase activating protein catalytic subunit
ZNF337-AS1 (ZNF337 antisense RNA 1)
BCL2L1 (BCL2 like 1)
BPIFB1 (BPI fold containing family B member 1)
EIF2S2 (eukaryotic translation initiation factor 2 subunit
UQCC1 (ubiquinol-cytochrome c reductase complex
PHF20 (PHD finger protein 20)
CNBD2 (cyclic nucleotide binding domain containing 2)
NORAD (non-coding RNA activated by DNA damage)
BLCAP (BLCAP apoptosis inducing factor)
YWHAB (tyrosine 3-monooxygenase/tryptophan 5-
STK4 (serine/threonine kinase 4)
SLC2A10 (solute carrier family 2 member 10)
BCAS4 (breast carcinoma amplified sequence 4)
BCAS1 (brain enriched myelin associated protein 1)
VAPB (VAMP associated protein B and C)
MTG2 (mitochondrial ribosome associated GTPase 2)
UCKL1-AS1 (UCKL1 antisense RNA 1)
URB1-AS1 (URB1 antisense RNA 1 (head to head))
IL10RB-DT (IL10RB divergent transcript)
DONSON (DNA replication fork stabilization factor DONSON)
LINC00649 (long intergenic non-protein coding RNA 649)
KCNE1 (potassium voltage-gated channel subfamily E
LINC01436 (long intergenic non-protein coding RNA 1436)
CBR3-AS1 (CBR3 antisense RNA 1)
TTC3-AS1 (TTC3 antisense RNA 1)
LCA5L (lebercilin LCA5 like)
PLAC4 (placenta enriched 4)
MX2 (MX dynamin like GTPase 2)
UMODL1-AS1 (UMODL1 antisense RNA 1)
LINC01671 (long intergenic non-protein coding RNA 1671)
NDUFV3 (NADH:ubiquinone oxidoreductase subunit V3)
AATBC (apoptosis associated transcript in bladder cancer)
CFAP410 (cilia and flagella associated protein 410)
ITGB2-AS1 (ITGB2 antisense RNA 1)
LINC00205 (long intergenic non-protein coding RNA 205)
MCM3AP-AS1 (MCM3AP antisense RNA 1)
PRMT2 (protein arginine methyltransferase 2)
LINC00528 (long intergenic non-protein coding RNA 528)
UFD1 (ubiquitin recognition factor in ER associated
PI4KA (phosphatidylinositol 4-kinase alpha)
LINC01637 (long intergenic non-protein coding RNA 1637)
MAPK1 (mitogen-activated protein kinase 1)
C22orf15 (chromosome 22 open reading frame 15)
KIAA1671-AS1 (KIAA1671 antisense RNA 1)
TFIP11-DT (TFIP11 divergent transcript)
MIATNB (MIAT neighbor)
ASCC2 (activating signal cointegrator 1 complex subunit 2)
CCDC157 (coiled-coil domain containing 157)
RNF215 (ring finger protein 215)
SLC35E4 (solute carrier family 35 member E4)
DUSP18 (dual specificity phosphatase 18)
TUG1 (taurine up-regulated 1)
HMOX1 (heme oxygenase 1)
IFT27 (intraflagellar transport 27)
NCF4-AS1 (NCF4 antisense RNA 1)
ANKRD54 (ankyrin repeat domain 54)
EIF3L (eukaryotic translation initiation factor 3 subunit L)
PLA2G6 (phospholipase A2 group VI)
TPTEP2-CSNK1E (TPTEP2-CSNK1E readthrough)
CSNK1E (casein kinase 1 epsilon)
DDX17 (DEAD-box helicase 17)
NPTXR (neuronal pentraxin receptor)
XPNPEP3 (X-prolyl aminopeptidase 3)
EP300-AS1 (EP300 antisense RNA 1)
CSDC2 (cold shock domain containing C2)
SREBF2-AS1 (SREBF2 antisense RNA 1)
WBP2NL (WBP2 N-terminal like)
A4GALT (alpha 1,4-galactosyltransferase (P blood group))
TTLL1-AS1 (TTLL1 antisense RNA 1)
FAM118A (family with sequence similarity 118 member A)
TYMP (thymidine phosphorylase)
KLHDC7B-DT (KLHDC7B divergent transcript)
RABL2B (RAB, member of RAS oncogene family like 2B)
CHL1 (cell adhesion molecule L1 like)
IL5RA (interleukin 5 receptor subunit alpha)
BHLHE40 (basic helix-loop-helix family member e40)
VGLL4 (vestigial like family member 4)
SYN2 (synapsin II)
RAF1 (Raf-1 proto-oncogene, serine/threonine kinase)
XPC (XPC complex subunit, DNA damage recognition and
XPC-AS1 (XPC antisense RNA 1)
MRPS25 (mitochondrial ribosomal protein S25)
SH3BP5-AS1 (SH3BP5 antisense RNA 1)
ANKRD28 (ankyrin repeat domain 28)
NEK10 (NIMA related kinase 10)
CMTM7 (CKLF like MARVEL transmembrane domain
PDCD6IP-DT (PDCD6IP divergent transcript)
LRRFIP2 (LRR binding FLII interacting protein 2)
ACAA1 (acetyl-CoA acyltransferase 1)
EIF1B (eukaryotic translation initiation factor 1B)
ENTPD3-AS1 (ENTPD3 antisense RNA 1)
LINC02158 (long intergenic non-protein coding RNA 2158)
SNRK-AS1 (SNRK antisense RNA 1)
CCR1 (C-C motif chemokine receptor 1)
CCR5AS (CCR5 antisense RNA)
CCR5 (C-C motif chemokine receptor 5)
KIF9-AS1 (KIF9 antisense RNA 1)
ARIH2 (ariadne RBR E3 ubiquitin protein ligase 2)
RBM6 (RNA binding motif protein 6)
SEMA3F-AS1 (SEMA3F antisense RNA 1)
ZMYND10-AS1 (ZMYND10 antisense RNA 1)
ALAS1 (5'-aminolevulinate synthase 1)
GLT8D1 (glycosyltransferase 8 domain containing 1)
DNAH12 (dynein axonemal heavy chain 12)
FAM107A (family with sequence similarity 107 member A)
PSMD6-AS2 (PSMD6 antisense RNA 2)
PSMD6 (proteasome 26S subunit, non-ATPase 6)
FOXP1-IT1 (FOXP1 intronic transcript 1)
ZNF717 (zinc finger protein 717)
DCBLD2 (discoidin, CUB and LCCL domain containing 2)
NIT2 (nitrilase family member 2)
TFG (trafficking from ER to golgi regulator)
CBLB (Cbl proto-oncogene B)
TBILA (TGF-beta induced lncRNA)
SLC35A5 (solute carrier family 35 member A5)
CD200R1L-AS1 (CD200R1L antisense RNA 1)
QTRT2 (queuine tRNA-ribosyltransferase accessory subunit
GSK3B (glycogen synthase kinase 3 beta)
WDR5B-DT (WDR5B divergent transcript)
HSPBAP1 (HSPB1 associated protein 1)
LINC02035 (long intergenic non-protein coding RNA 2035)
ALDH1L1-AS1 (ALDH1L1 antisense RNA 1)
MGLL (monoglyceride lipase)
SNORA7B (small nucleolar RNA, H/ACA box 7B)
EFCAB12 (EF-hand calcium binding domain 12)
SLC35G2 (solute carrier family 35 member G2)
COPB2 (COPI coat complex subunit beta 2)
PLSCR1 (phospholipid scramblase 1)
HLTF-AS1 (HLTF antisense RNA 1)
ANKUB1 (ankyrin repeat and ubiquitin domain containing
SERP1 (stress associated endoplasmic reticulum protein 1)
DHX36 (DEAH-box helicase 36)
TRIM59 (tripartite motif containing 59)
TMEM212 (transmembrane protein 212)
CCDC39 (coiled-coil domain containing 39)
FXR1 (FMR1 autosomal homolog 1)
LINC02031 (long intergenic non-protein coding RNA 2031)
MCCC1-AS1 (MCCC1 antisense RNA 1)
B3GNT5 (UDP-GlcNAc:betaGal beta-1,3-N-
ABCC5 (ATP binding cassette subfamily C member 5)
MAP3K13 (mitogen-activated protein kinase kinase kinase
TMEM41A (transmembrane protein 41A)
EIF4A2 (eukaryotic translation initiation factor 4A2)
RFC4 (replication factor C subunit 4)
BCL6 (BCL6 transcription repressor)
UTS2B (urotensin 2B)
TMEM44-AS1 (TMEM44 antisense RNA 1)
TMEM44 (transmembrane protein 44)
MUC20-OT1 (MUC20 overlapping transcript)
MUC20 (mucin 20, cell surface associated)
MUC4 (mucin 4, cell surface associated)
TNK2 (tyrosine kinase non receptor 2)
PCYT1A (phosphate cytidylyltransferase 1A, choline)
SLC51A (solute carrier family 51 subunit alpha)
PIGX (phosphatidylinositol glycan anchor biosynthesis class
LMLN-AS1 (LMLN antisense RNA 1)
GAK (cyclin G associated kinase)
SPON2 (spondin 2)
CTBP1-DT (CTBP1 divergent transcript)
MXD4 (MAX dimerization protein 4)
ADD1 (adducin 1)
LINC02481 (long intergenic non-protein coding RNA 2481)
TADA2B (transcriptional adaptor 2B)
BOD1L1 (biorientation of chromosomes in cell division 1
DHX15 (DEAH-box helicase 15)
SEPSECS-AS1 (SEPSECS antisense RNA 1 (head to head))
STIM2 (stromal interaction molecule 2)
PCDH7 (protocadherin 7)
DTHD1 (death domain containing 1)
UGDH-AS1 (UGDH antisense RNA 1)
RHOH (ras homolog family member H)
LINC02265 (long intergenic non-protein coding RNA 2265)
SGCB (sarcoglycan beta)
USP46-DT (USP46 divergent transcript)
SRD5A3-AS1 (SRD5A3 antisense RNA 1)
TMEM165 (transmembrane protein 165)
CXCL5 (C-X-C motif chemokine ligand 5)
THAP6 (THAP domain containing 6)
SHROOM3-AS1 (SHROOM3 antisense RNA 1)
BMP2K (BMP2 inducible kinase)
HNRNPD (heterogeneous nuclear ribonucleoprotein D)
PLAC8 (placenta associated 8)
MRPS18C (mitochondrial ribosomal protein S18C)
WDFY3 (WD repeat and FYVE domain containing 3)
TIGD2 (tigger transposable element derived 2)
ADH5 (alcohol dehydrogenase 5 (class III), chi polypeptide)
TRMT10A (tRNA methyltransferase 10A)
UBE2D3 (ubiquitin conjugating enzyme E2 D3)
UBE2D3-AS1 (UBE2D3 antisense RNA 1)
CXXC4 (CXXC finger protein 4)
ARHGEF38 (Rho guanine nucleotide exchange factor 38)
ALPK1 (alpha kinase 1)
SNHG8 (small nucleolar RNA host gene 8)
NAA15 (N-alpha-acetyltransferase 15, NatA auxiliary
RAB33B-AS1 (RAB33B antisense RNA 1)
SETD7 (SET domain containing 7, histone lysine
MIR4453HG (MIR4453 host gene)
LINC02363 (long intergenic non-protein coding RNA 2363)
IRF2-DT (IRF2 divergent transcript)
ANKRD37 (ankyrin repeat domain 37)
PDCD6 (programmed cell death 6)
TPPP (tubulin polymerization promoting protein)
TENT4A (terminal nucleotidyltransferase 4A)
SEMA5A (semaphorin 5A)
CCT5 (chaperonin containing TCP1 subunit 5)

PARGP1 (poly(ADP-ribose) glycohydrolase pseudogene 1)
BMS1P1 (BMS1 pseudogene 1)
SHQ1P1 (SHQ1, H/ACA ribonucleoprotein assembly factor
CALM2P2 (calmodulin 2 pseudogene 2)
KRT8P49 (keratin 8 pseudogene 49)
HSP90AA2P (heat shock protein 90 alpha family class A
ALG1L8P (ALG1 like 8, pseudogene)
OTUD4P1 (OTUD4 pseudogene 1)
HSPA8P5 (heat shock protein family A (Hsp70) member 8
EEF1A1P4 (eukaryotic translation elongation factor 1 alpha
GOLGA2P5 (GOLGA2 pseudogene 5)
NUS1P2 (NUS1 pseudogene 2)
CALM2P3 (calmodulin 2 pseudogene 3)
OR7E36P (olfactory receptor family 7 subfamily E member
MRPS31P4 (mitochondrial ribosomal protein S31
DYNLL1P1 (dynein light chain LC8-type 1 pseudogene 1)
DYNLL1P2 (dynein light chain LC8-type 1 pseudogene 2)
HERC2P2 (hect domain and RLD 2 pseudogene 2)
HERC2P9 (hect domain and RLD 2 pseudogene 9)
HMGN2P5 (high mobility group nucleosomal binding
SORD2P (sorbitol dehydrogenase 2, pseudogene)
SCAND2P (SCAN domain containing 2 pseudogene)
ABCC6P2 (ATP binding cassette subfamily C member 6
ABCC6P1 (ATP binding cassette subfamily C member 6
CES1P1 (carboxylesterase 1 pseudogene 1)
URAHP (urate (hydroxyiso-) hydrolase, pseudogene)
MEIS3P1 (Meis homeobox 3 pseudogene 1)
UPF3AP1 (UPF3A pseudogene 1)
MEIS3P2 (Meis homeobox 3 pseudogene 2)
LRRC37BP1 (leucine rich repeat containing 37B pseudogene
LRRC37A11P (leucine rich repeat containing 37 member
LRRC37A4P (leucine rich repeat containing 37 member A4,
LINC02210 (long intergenic non-protein coding RNA 2210)
LRRC37A17P (leucine rich repeat containing 37 member
LRRC37A7P (leucine rich repeat containing 37 member A7,
ZNF271P (zinc finger protein 271, pseudogene)
ZNF788P (zinc finger family member 788, pseudogene)
CYP4F23P (cytochrome P450 family 4 subfamily F member
IPO5P1 (importin 5 pseudogene 1)
CYP2T1P (cytochrome P450 family 2 subfamily T member 1,
CYP2F2P (cytochrome P450 family 2 subfamily F member 2,
CYP2B7P (cytochrome P450 family 2 subfamily B member 7,
CYP2G2P (cytochrome P450 family 2 subfamily G member 2,
ZNF321P (zinc finger protein 321, pseudogene)
CENPBD1P1 (CENPB DNA-binding domains containing 1
FNBP1P1 (formin binding protein 1 pseudogene 1)
PEBP1P2 (phosphatidylethanolamine binding protein 1
MED15P9 (mediator complex subunit 15 pseudogene 9)
YWHAEP5 (tyrosine 3-monooxygenase/tryptophan 5-
NUDCP2 (nuclear distribution C pseudogene 2)
KRT8P10 (keratin 8 pseudogene 10)
TSPY26P (testis specific protein Y-linked 26, pseudogene)
OR7E122P (olfactory receptor family 7 subfamily E member
EMC3-AS1 (EMC3 antisense RNA 1)
TPRXL (tetrapeptide repeat homeobox like (pseudogene))
RNF7P1 (ring finger protein 7 pseudogene 1)
ALG1L6P (ALG1 like 6, pseudogene)
PDCL3P4 (PDCL3 pseudogene 4)
KRT8P36 (keratin 8 pseudogene 36)
ST13P15 (ST13, Hsp70 interacting protein pseudogene 15)
SNRPCP3 (small nuclear ribonucleoprotein polypeptide C
AADACP1 (arylacetamide deacetylase pseudogene 1)
ALG1L15P (ALG1 like 15, pseudogene)
KRT8P12 (keratin 8 pseudogene 12)
KRT8P13 (keratin 8 pseudogene 13)
FAM86EP (family with sequence similarity 86, member A
HSP90AB3P (heat shock protein 90 alpha family class B
GTF2IP12 (general transcription factor IIi pseudogene 12)
SDHAP3 (succinate dehydrogenase complex flavoprotein
EEF1A1P19 (eukaryotic translation elongation factor 1
KRT8P32 (keratin 8 pseudogene 32)
CTBP2P4 (CTBP2 pseudogene 4)
KRT8P33 (keratin 8 pseudogene 33)
KRT8P48 (keratin 8 pseudogene 48)
CMAHP (cytidine monophospho-N-acetylneuraminic acid
ZNF204P (zinc finger protein 204, pseudogene)
HLA-H (major histocompatibility complex, class I, H
HLA-K (major histocompatibility complex, class I, K
ZNRD1ASP (zinc ribbon domain containing 1 antisense,
ADH5P4 (ADH5 pseudogene 4)
BDH2P1 (3-hydroxybutyrate dehydrogenase 2, pseudogene
ALG1L5P (ALG1 like 5, pseudogene)
HSPA8P8 (heat shock protein family A (Hsp70) member 8
RWDD4P2 (RWD domain containing 4 pseudogene 2)
GTF2IP13 (general transcription factor IIi pseudogene 13)
FKBP9P1 (FKBP prolyl isomerase 9 pseudogene 1)
PSPHP1 (phosphoserine phosphatase pseudogene 1)
STAG3L3 (stromal antigen 3-like 3 (pseudogene))
ABHD11-AS1 (ABHD11 antisense RNA 1 (tail to tail))
KRT8P51 (keratin 8 pseudogene 51)
KRT8P3 (keratin 8 pseudogene 3)
ZNF252P (zinc finger protein 252, pseudogene)
PRDX1P1 (peroxiredoxin 1 pseudogene 1)
FAM27B (family with sequence similarity 27 member B)
LRRC37A5P (leucine rich repeat containing 37 member A5,
CUTALP (cutA divalent cation tolerance like, pseudogene)
TTC3P1 (tetratricopeptide repeat domain 3 pseudogene 1)
CD24P4 (CD24 molecule pseudogene 4)

CBX1 (chromobox 1)
SNF8 (SNF8 subunit of ESCRT-II)
ZNF652 (zinc finger protein 652)
FAM117A (family with sequence similarity 117 member A)
MRPL27 (mitochondrial ribosomal protein L27)
ACSF2 (acyl-CoA synthetase family member 2)
TOB1-AS1 (TOB1 antisense RNA 1)
ANKFN1 (ankyrin repeat and fibronectin type III domain
AKAP1-DT (AKAP1 divergent transcript)
MSI2 (musashi RNA binding protein 2)
LINC02875 (long intergenic non-protein coding RNA 2875)
TLK2 (tousled like kinase 2)
MARCHF10 (membrane associated ring-CH-type finger 10)
FTSJ3 (FtsJ RNA 2'-O-methyltransferase 3)
PRR29-AS1 (PRR29 antisense RNA 1)
ICAM2 (intercellular adhesion molecule 2)
HELZ-AS1 (HELZ antisense RNA 1)
KCNJ2-AS1 (KCNJ2 antisense RNA 1)
LINC00511 (long intergenic non-protein coding RNA 511)
COG1 (component of oligomeric golgi complex 1)
GPRC5C (G protein-coupled receptor class C group 5
SLC9A3R1-AS1 (SLC9A3R1 antisense RNA 1)
MRPS7 (mitochondrial ribosomal protein S7)
MIF4GD-DT (MIF4GD divergent transcript)
RECQL5 (RecQ like helicase 5)
H3-3B (H3.3 histone B)
ST6GALNAC1 (ST6 N-acetylgalactosaminide alpha-2,6-
SOCS3 (suppressor of cytokine signaling 3)
PGS1 (phosphatidylglycerophosphate synthase 1)
ENGASE (endo-beta-N-acetylglucosaminidase)
GAA (alpha glucosidase)
SGSH (N-sulfoglucosamine sulfohydrolase)
RNF213 (ring finger protein 213)
RNF213-AS1 (RNF213 antisense RNA 1)
ENDOV (endonuclease V)
BAIAP2-DT (BAIAP2 divergent transcript)
BAIAP2 (BAR/IMD domain containing adaptor protein 2)
OXLD1 (oxidoreductase like domain containing 1)
HGS (hepatocyte growth factor-regulated tyrosine kinase
ALYREF (Aly/REF export factor)
MAFG-DT (MAFG divergent transcript)
CCDC57 (coiled-coil domain containing 57)
CSNK1D (casein kinase 1 delta)
ENOSF1 (enolase superfamily member 1)
MYL12A (myosin light chain 12A)
LINC00526 (long intergenic non-protein coding RNA 526)
TUBB6 (tubulin beta 6 class V)
CEP192-DT (CEP192 divergent transcript)
ABHD3 (abhydrolase domain containing 3, phospholipase)
TTC39C-AS1 (TTC39C antisense RNA 1)
KCTD1 (potassium channel tetramerization domain
AQP4-AS1 (AQP4 antisense RNA 1)
LINC01908 (long intergenic non-protein coding RNA 1908)
COSMOC (cell fate and sterol metabolism associated
TPGS2 (tubulin polyglutamylase complex subunit 2)
KATNAL2 (katanin catalytic subunit A1 like 2)
SMAD2 (SMAD family member 2)
ZBTB7C (zinc finger and BTB domain containing 7C)
ELAC1 (elaC ribonuclease Z 1)
TXNL1 (thioredoxin like 1)
NEDD4L (NEDD4 like E3 ubiquitin protein ligase)
KDSR-DT (KDSR divergent transcript)
SERPINB11 (serpin family B member 11)
CNDP2 (carnosine dipeptidase 2)
LINC00683 (long intergenic non-protein coding RNA 683)
ATP9B (ATPase phospholipid transporting 9B (putative))
PARD6G-AS1 (PARD6G antisense RNA 1)
PLPP2 (phospholipid phosphatase 2)
PTBP1 (polypyrimidine tract binding protein 1)
CIRBP-AS1 (CIRBP antisense RNA 1)
PWWP3A (PWWP domain containing 3A, DNA repair factor)
ADAT3 (adenosine deaminase tRNA specific 3)
JSRP1 (junctional sarcoplasmic reticulum protein 1)
LMNB2 (lamin B2)
TLE5 (TLE family member 5, transcriptional modulator)
GNA15-DT (GNA15 divergent transcript)
LRG1 (leucine rich alpha-2-glycoprotein 1)
PSPN (persephin)
KHSRP (KH-type splicing regulatory protein)
C3 (complement C3)
SH2D3A (SH2 domain containing 3A)
STXBP2 (syntaxin binding protein 2)
RAB11B-AS1 (RAB11B antisense RNA 1)
HNRNPM (heterogeneous nuclear ribonucleoprotein M)
ILF3-DT (ILF3 divergent transcript)
DNM2 (dynamin 2)
LDLR (low density lipoprotein receptor)
SPC24 (SPC24 component of NDC80 kinetochore complex)
ODAD3 (outer dynein arm docking complex subunit 3)
PRKCSH (protein kinase C substrate 80K-H)
ZNF440 (zinc finger protein 440)
HOOK2 (hook microtubule tethering protein 2)
JUNB (JunB proto-oncogene, AP-1 transcription factor
FARSA-AS1 (FARSA antisense RNA 1)
NFIX (nuclear factor I X)
SAMD1 (sterile alpha motif domain containing 1)
EPS15L1 (epidermal growth factor receptor pathway
C19orf44 (chromosome 19 open reading frame 44)
NWD1 (NACHT and WD repeat domain containing 1)
PGLS-DT (PGLS divergent transcript)
MAST3-AS1 (MAST3 antisense RNA 1)
IFI30 (IFI30 lysosomal thiol reductase)
UBA52 (ubiquitin A-52 residue ribosomal protein fusion
ZNF506 (zinc finger protein 506)
ZNF493 (zinc finger protein 493)
ZNF429 (zinc finger protein 429)
DPY19L3-DT (DPY19L3 divergent transcript)
CEP89 (centrosomal protein 89)
TMEM147-AS1 (TMEM147 antisense RNA 1)
NFKBID (NFKB inhibitor delta)
TBCB (tubulin folding cofactor B)
LINC00665 (long intergenic non-protein coding RNA 665)
ZNF529-AS1 (ZNF529 antisense RNA 1)
ZNF529 (zinc finger protein 529)
ZNF875 (zinc finger protein 875)
ZNF793 (zinc finger protein 793)
SPINT2 (serine peptidase inhibitor, Kunitz type 2)
MAP4K1-AS1 (MAP4K1 antisense RNA 1)
HNRNPL (heterogeneous nuclear ribonucleoprotein L)
SAMD4B (sterile alpha motif domain containing 4B)
FCGBP (Fc fragment of IgG binding protein)
ZNF780A (zinc finger protein 780A)
C19orf54 (chromosome 19 open reading frame 54)
CYP2F1 (cytochrome P450 family 2 subfamily F member 1)
TGFB1 (transforming growth factor beta 1)
CEACAM6 (CEA cell adhesion molecule 6)
CD177 (CD177 molecule)
ZNF575 (zinc finger protein 575)
ZNF224 (zinc finger protein 224)
ZNF226 (zinc finger protein 226)
ZNF235 (zinc finger protein 235)
ERCC1 (ERCC excision repair 1, endonuclease non-catalytic
FKRP (fukutin related protein)
C5AR1 (complement C5a receptor 1)
NOP53-AS1 (NOP53 antisense RNA 1)
CARD8-AS1 (CARD8 antisense RNA 1)
FUT2 (fucosyltransferase 2)
FUT1 (fucosyltransferase 1 (H blood group))
PPP1R15A (protein phosphatase 1 regulatory subunit 15A)
NUCB1-AS1 (NUCB1 antisense RNA 1)
SNRNP70 (small nuclear ribonucleoprotein U1 subunit 70)
TRPM4 (transient receptor potential cation channel
MED25 (mediator complex subunit 25)
PTOV1-AS1 (PTOV1 antisense RNA 1)
PTOV1 (PTOV1 extended AT-hook containing adaptor
PTOV1-AS2 (PTOV1 antisense RNA 2)
PNKP (polynucleotide kinase 3'-phosphatase)
ZNF473 (zinc finger protein 473)
SIGLEC10-AS1 (SIGLEC10 antisense RNA 1)
FPR3 (formyl peptide receptor 3)
ZNF880 (zinc finger protein 880)
ZNF528-AS1 (ZNF528 antisense RNA 1)
ZNF677 (zinc finger protein 677)
MBOAT7 (membrane bound O-acyltransferase domain
LENG9 (leukocyte receptor cluster member 9)
DNAAF3-AS1 (DNAAF3 antisense RNA 1)
FIZ1 (FLT3 interacting zinc finger 1)
TRAPPC2B (trafficking protein particle complex subunit 2B)
ZNF552 (zinc finger protein 552)
ZNF814 (zinc finger protein 814)
ZNF135 (zinc finger protein 135)
ZNF544 (zinc finger protein 544)
ZNF8-DT (ZNF8 divergent transcript)
ERVK3-1 (endogenous retrovirus group K3 member 1)
SLC27A5 (solute carrier family 27 member 5)
MZF1-AS1 (MZF1 antisense RNA 1)
MZF1 (myeloid zinc finger 1)
TMEM18-DT (TMEM18 divergent transcript)
TRAPPC12 (trafficking protein particle complex subunit 12)
ID2 (inhibitor of DNA binding 2)
IAH1 (isoamyl acetate hydrolyzing esterase 1 (putative))
HPCAL1 (hippocalcin like 1)
GEN1 (GEN1 Holliday junction 5' flap endonuclease)
TTC32 (tetratricopeptide repeat domain 32)
DTNB-AS1 (DTNB antisense RNA 1)
FAM166C (family with sequence similarity 166 member C)
SLC5A6 (solute carrier family 5 member 6)
PPM1G (protein phosphatase, Mg2+/Mn2+ dependent 1G)
BABAM2 (BRISC and BRCA1 A complex member 2)
CAPN13 (calpain 13)
CEBPZOS (CEBPZ opposite strand)
MAP4K3-DT (MAP4K3 divergent transcript)
EML4-AS1 (EML4 antisense RNA 1)
COX7A2L (cytochrome c oxidase subunit 7A2 like)
LINC01833 (long intergenic non-protein coding RNA 1833)
SIX3-AS1 (SIX3 antisense RNA 1)
RHOQ (ras homolog family member Q)
PIGF (phosphatidylinositol glycan anchor biosynthesis class
CALM2 (calmodulin 2)
FBXO11 (F-box protein 11)
PPP1R21-DT (PPP1R21 divergent transcript)
PPP4R3B (protein phosphatase 4 regulatory subunit 3B)
PPP4R3B-DT (PPP4R3B divergent transcript)
BCL11A (BAF chromatin remodeling complex subunit
CCT4 (chaperonin containing TCP1 subunit 4)
OTX1 (orthodenticle homeobox 1)
LINC01890 (long intergenic non-protein coding RNA 1890)
ANXA4 (annexin A4)
PCBP1-AS1 (PCBP1 antisense RNA 1)
NAGK (N-acetylglucosamine kinase)
DCTN1-AS1 (DCTN1 antisense RNA 1)
LOXL3 (lysyl oxidase like 3)
GCFC2 (GC-rich sequence DNA-binding factor 2)
PARTICL (promoter of MAT2A antisense radiation-induced
RNF103-CHMP3 (RNF103-CHMP3 readthrough)
CHMP3 (charged multivesicular body protein 3)
LINC01943 (long intergenic non-protein coding RNA 1943)
EIF2AK3-DT (EIF2AK3 divergent transcript)
IGKC (immunoglobulin kappa constant)
ZNF514 (zinc finger protein 514)

LINC01513 (long intergenic non-protein coding RNA 1513)
GOLPH3-DT (GOLPH3 divergent transcript)
SUB1 (SUB1 regulator of transcription)
TTC23L-AS1 (TTC23L antisense RNA 1)
NIPBL-DT (NIPBL divergent transcript)
NNT-AS1 (NNT antisense RNA 1)
MRPS30 (mitochondrial ribosomal protein S30)
GPBP1 (GC-rich promoter binding protein 1)
MARVELD2 (MARVEL domain containing 2)
FCHO2-DT (FCHO2 divergent transcript)
BTF3-DT (BTF3 divergent transcript)
ARHGEF28 (Rho guanine nucleotide exchange factor 28)
LINC02122 (long intergenic non-protein coding RNA 2122)
NSA2 (NSA2 ribosome biogenesis factor)
FER (FER tyrosine kinase)
SRP19 (signal recognition particle 19)
MIR1244-2 (microRNA 1244-2)
SLC12A2 (solute carrier family 12 member 2)
FNIP1 (folliculin interacting protein 1)
P4HA2-AS1 (P4HA2 antisense RNA 1)
IRF1-AS1 (IRF1 antisense RNA 1)
IRF1 (interferon regulatory factor 1)
LINC01843 (long intergenic non-protein coding RNA 1843)
MACROH2A1 (macroH2A.1 histone)
EGR1 (early growth response 1)
SNHG4 (small nucleolar RNA host gene 4)
PAIP2 (poly(A) binding protein interacting protein 2)
IK (IK cytokine)
WDR55 (WD repeat domain 55)
DELE1 (DAP3 binding cell death enhancer 1)
ARHGAP26 (Rho GTPase activating protein 26)
ARHGEF37 (Rho guanine nucleotide exchange factor 37)
TNIP1 (TNFAIP3 interacting protein 1)
ATOX1 (antioxidant 1 copper chaperone)
G3BP1 (G3BP stress granule assembly factor 1)
FAXDC2 (fatty acid hydroxylase domain containing 2)
MED7 (mediator complex subunit 7)
FABP6-AS1 (FABP6 antisense RNA 1)
C5orf58 (chromosome 5 open reading frame 58)
ERGIC1 (endoplasmic reticulum-golgi intermediate
PHYKPL (5-phosphohydroxy-L-lysine phospho-lyase)
ZNF354A (zinc finger protein 354A)
RUFY1-AS1 (RUFY1 antisense RNA 1)
MRNIP (MRN complex interacting protein)
RNF130 (ring finger protein 130)
FOXC1 (forkhead box C1)
SERPINB1 (serpin family B member 1)
PSMG4 (proteasome assembly chaperone 4)
SLC22A23 (solute carrier family 22 member 23)
EDN1 (endothelin 1)
NOL7 (nucleolar protein 7)
ATXN1 (ataxin 1)
H2AC4 (H2A clustered histone 4)
HCG11 (HLA complex group 11)
ZNF165 (zinc finger protein 165)
ZSCAN31 (zinc finger and SCAN domain containing 31)
HCG14 (HLA complex group 14)
TRIM31-AS1 (TRIM31 antisense RNA 1)
DHX16 (DEAH-box helicase 16)
IER3-AS1 (IER3 antisense RNA 1)
HCG20 (HLA complex group 20 (non-protein coding))
DDX39B (DExD-box helicase 39B)
TNF (tumor necrosis factor)
CLIC1 (chloride intracellular channel 1)
SAPCD1-AS1 (SAPCD1 antisense RNA 1)
SLC44A4 (solute carrier family 44 member 4)
HLA-DRB1 (major histocompatibility complex, class II, DR
HLA-DOA (major histocompatibility complex, class II, DO
PFDN6 (prefoldin subunit 6)
PANDAR (promoter of CDKN1A antisense DNA damage
MTCH1 (mitochondrial carrier 1)
KIF6 (kinesin family member 6)
GNMT (glycine N-methyltransferase)
KLC4-AS1 (KLC4 antisense RNA 1)
ANKRD66 (ankyrin repeat domain 66)
GCLC (glutamate-cysteine ligase catalytic subunit)
SCAT8 (S-phase cancer associated transcript 8)
PHIP (pleckstrin homology domain interacting protein)
HMGN3 (high mobility group nucleosomal binding domain
HMGN3-AS1 (HMGN3 antisense RNA 1)
PNRC1 (proline rich nuclear receptor coactivator 1)
MANEA-DT (MANEA divergent transcript)
PNISR (PNN interacting serine and arginine rich protein)
CALHM6-AS1 (CALHM6 antisense RNA 1)
MCM9 (minichromosome maintenance 9 homologous
VNN3 (vanin 3)
PEX7 (peroxisomal biogenesis factor 7)
WAKMAR2 (wound and keratinocyte migration associated
TNFAIP3 (TNF alpha induced protein 3)
LRP11 (LDL receptor related protein 11)
RAET1E-AS1 (RAET1E antisense RNA 1)
ARID1B (AT-rich interaction domain 1B)
DYNLT1 (dynein light chain Tctex-type 1)
MIR3918 (microRNA 3918)
RSPH3 (radial spoke head 3)
SOD2 (superoxide dismutase 2)
IGF2R (insulin like growth factor 2 receptor)
AGPAT4 (1-acylglycerol-3-phosphate O-acyltransferase 4)
PSMB1 (proteasome 20S subunit beta 1)
SUN1 (Sad1 and UNC84 domain containing 1)
RSPH10B (radial spoke head 10 homolog B)
RSPH10B2 (radial spoke head 10 homolog B2)
MIOS-DT (MIOS divergent transcript)
TSPAN13 (tetraspanin 13)
AGR2 (anterior gradient 2, protein disulphide isomerase
LINC02888 (long intergenic non-protein coding RNA 2888)
SP8 (Sp8 transcription factor)
TRA2A (transformer 2 alpha homolog)
TAX1BP1-AS1 (TAX1BP1 antisense RNA 1)
TAX1BP1 (Tax1 binding protein 1)
PRR15 (proline rich 15)
GARS1-DT (GARS1 divergent transcript)
HERPUD2-AS1 (HERPUD2 antisense RNA 1)
SEPTIN7 (septin 7)
DBNL (drebrin like)
DDX56 (DEAD-box helicase 56)
PPIA (peptidylprolyl isomerase A)
ABCA13 (ATP binding cassette subfamily A member 13)
ZNF273 (zinc finger protein 273)
ERV3-1 (endogenous retrovirus group 3 member 1,
LINC00174 (long intergenic non-protein coding RNA 174)
LINC02604 (long intergenic non-protein coding RNA 2604)
AUTS2 (activator of transcription and developmental
BUD23 (BUD23 rRNA methyltransferase and ribosome
CLDN3 (claudin 3)
ELN-AS1 (ELN antisense RNA 1)
MDH2 (malate dehydrogenase 2)
DDX3ILA1 (DDX3 interacting lncRNA 1)
SRI-AS1 (SRI antisense RNA 1)
AKAP9 (A-kinase anchoring protein 9)
BRI3 (brain protein I3)
LAMTOR4 (late endosomal/lysosomal adaptor, MAPK and
PILRB (paired immunoglobin like type 2 receptor beta)
PCOLCE-AS1 (PCOLCE antisense RNA 1)
MUC12 (mucin 12, cell surface associated)
EMSLR (E2F1 mRNA stabilizing lncRNA)
KMT2E-AS1 (KMT2E antisense RNA 1)
EFCAB10 (EF-hand calcium binding domain 10)
NAMPT (nicotinamide phosphoribosyltransferase)
HBP1 (HMG-box transcription factor 1)
WASL-DT (WASL divergent transcript)
CHCHD3 (coiled-coil-helix-coiled-coil-helix domain
LRGUK (leucine rich repeats and guanylate kinase domain
AGBL3 (AGBL carboxypeptidase 3)
CYREN (cell cycle regulator of NHEJ)
MTPN (myotrophin)
TBXAS1 (thromboxane A synthase 1)
BRAF (B-Raf proto-oncogene, serine/threonine kinase)
MGAM (maltase-glucoamylase)
TRBC1 (T cell receptor beta constant 1)
TRBC2 (T cell receptor beta constant 2)
EPHA1 (EPH receptor A1)
WDR86 (WD repeat domain 86)
WDR86-AS1 (WDR86 antisense RNA 1)
PRKAG2-AS1 (PRKAG2 antisense RNA 1)
PAXIP1-DT (PAXIP1 divergent transcript)
DNAJB6 (DnaJ heat shock protein family (Hsp40) member
NAT1 (N-acetyltransferase 1)
SLC25A37 (solute carrier family 25 member 37)
BNIP3L (BCL2 interacting protein 3 like)
PTK2B (protein tyrosine kinase 2 beta)
HMBOX1 (homeobox containing 1)
LINC00589 (long intergenic non-protein coding RNA 589)
DCTN6-DT (DCTN6 divergent transcript)
GSR (glutathione-disulfide reductase)
NSD3 (nuclear receptor binding SET domain protein 3)
PLEKHA2 (pleckstrin homology domain containing A2)
SLC20A2 (solute carrier family 20 member 2)
RP1 (RP1 axonemal microtubule associated)
SDCBP (syndecan binding protein)
ASPH (aspartate beta-hydroxylase)
YTHDF3-DT (YTHDF3 divergent transcript)
ARFGEF1 (ADP ribosylation factor guanine nucleotide
IL7 (interleukin 7)
LINC02605 (long intergenic non-protein coding RNA 2605)
TMEM67 (transmembrane protein 67)
LINC02894 (long intergenic non-protein coding RNA 2894)
EIF3H (eukaryotic translation initiation factor 3 subunit H)
SAMD12 (sterile alpha motif domain containing 12)
PCAT1 (prostate cancer associated transcript 1)
PHF20L1 (PHD finger protein 20 like 1)
LY6E-DT (LY6E divergent transcript)
RHPN1-AS1 (RHPN1 antisense RNA 1 (head to head))
EEF1D (eukaryotic translation elongation factor 1 delta)
IQANK1 (IQ motif and ankyrin repeat containing 1)
PARP10 (poly(ADP-ribose) polymerase family member 10)
CYHR1 (cysteine and histidine rich 1)
SMARCA2 (SWI/SNF related, matrix associated, actin
GLIS3-AS1 (GLIS3 antisense RNA 1)
DENND4C (DENN domain containing 4C)
CDKN2B-AS1 (CDKN2B antisense RNA 1)
ACO1 (aconitase 1)
BAG1 (BAG cochaperone 1)
ZBTB5 (zinc finger and BTB domain containing 5)
PTAR1 (protein prenyltransferase alpha subunit repeat
C9orf85 (chromosome 9 open reading frame 85)
ALDH1A1 (aldehyde dehydrogenase 1 family member A1)
ANXA1 (annexin A1)
NMRK1 (nicotinamide riboside kinase 1)
NTRK2 (neurotrophic receptor tyrosine kinase 2)
SECISBP2 (SECIS binding protein 2)
AOPEP (aminopeptidase O (putative))
MFSD14C (major facilitator superfamily domain containing
NANS (N-acetylneuraminate synthase)
SLC31A2 (solute carrier family 31 member 2)
SLC31A1 (solute carrier family 31 member 1)
PRPF4 (pre-mRNA processing factor 4)
STRBP (spermatid perinuclear RNA binding protein)
PTRH1 (peptidyl-tRNA hydrolase 1 homolog)
AK1 (adenylate kinase 1)
LINC00342 (long intergenic non-protein coding RNA 342)
NEURL3 (neuralized E3 ubiquitin protein ligase 3)
ANKRD23 (ankyrin repeat domain 23)
CRACDL (CRACD like)
TSGA10 (testis specific 10)
MITD1 (microtubule interacting and trafficking domain
TBC1D8-AS1 (TBC1D8 antisense RNA 1)
MRPS9-AS1 (MRPS9 antisense RNA 1)
C2orf49-DT (C2orf49 divergent transcript)
FHL2 (four and a half LIM domains 2)
CCDC138 (coiled-coil domain containing 138)
NPHP1 (nephrocystin 1)
IMP4 (IMP U3 small nucleolar ribonucleoprotein 4)
UBXN4 (UBX domain protein 4)
MBD5 (methyl-CpG binding domain protein 5)
PKP4-AS1 (PKP4 antisense RNA 1)
LY75 (lymphocyte antigen 75)
TTC21B-AS1 (TTC21B antisense RNA 1)
METTL5 (methyltransferase 5, N6-adenosine)
MAP3K20-AS1 (MAP3K20 antisense RNA 1)
ATP5MC3 (ATP synthase membrane subunit c locus 3)
AGPS (alkylglycerone phosphate synthase)
ORMDL1 (ORMDL sphingolipid biosynthesis regulator 1)
INPP1 (inositol polyphosphate-1-phosphatase)
STK17B (serine/threonine kinase 17b)
ANKRD44 (ankyrin repeat domain 44)
ANKRD44-AS1 (ANKRD44 antisense RNA 1)
HSPE1 (heat shock protein family E (Hsp10) member 1)
CASP8 (caspase 8)
STRADB (STE20 related adaptor beta)
ALS2 (alsin Rho guanine nucleotide exchange factor ALS2)
CYP20A1 (cytochrome P450 family 20 subfamily A member
ABI2 (abl interactor 2)
RAPH1 (Ras association (RalGDS/AF-6) and pleckstrin
EEF1B2 (eukaryotic translation elongation factor 1 beta 2)
CREB1 (cAMP responsive element binding protein 1)
LANCL1 (LanC like 1)
LINC01963 (long intergenic non-protein coding RNA 1963)
IGFBP2 (insulin like growth factor binding protein 2)
CXCR2 (C-X-C motif chemokine receptor 2)
ARPC2 (actin related protein 2/3 complex subunit 2)
CTDSP1 (CTD small phosphatase 1)
PTMA (prothymosin alpha)
PDE6D (phosphodiesterase 6D)
UGT1A6 (UDP glucuronosyltransferase family 1 member A6)
SH3BP4 (SH3 domain binding protein 4)
PER2 (period circadian regulator 2)
NDUFA10 (NADH:ubiquinone oxidoreductase subunit A10)
CAPN10-DT (CAPN10 divergent transcript)
FARP2 (FERM, ARH/RhoGEF and pleckstrin domain protein
ING5 (inhibitor of growth family member 5)
SDCBP2 (syndecan binding protein 2)
SNRPB (small nuclear ribonucleoprotein polypeptides B and
UBOX5-AS1 (UBOX5 antisense RNA 1)
PANK2-AS1 (PANK2 antisense RNA 1)
PANK2 (pantothenate kinase 2)
PRNP (prion protein)
SHLD1 (shieldin complex subunit 1)
RRBP1 (ribosome binding protein 1)
SNX5 (sorting nexin 5)
DTD1 (D-aminoacyl-tRNA deacylase 1)
LINC00653 (long intergenic non-protein coding RNA 653)
RALGAPA2 (Ral GTPase activating protein catalytic subunit
ZNF337-AS1 (ZNF337 antisense RNA 1)
HM13 (histocompatibility minor 13)
BCL2L1 (BCL2 like 1)
BPIFB1 (BPI fold containing family B member 1)
EIF2S2 (eukaryotic translation initiation factor 2 subunit
UQCC1 (ubiquinol-cytochrome c reductase complex
PHF20 (PHD finger protein 20)
CNBD2 (cyclic nucleotide binding domain containing 2)
NORAD (non-coding RNA activated by DNA damage)
DLGAP4 (DLG associated protein 4)
DLGAP4-AS1 (DLGAP4 antisense RNA 1)
BLCAP (BLCAP apoptosis inducing factor)
YWHAB (tyrosine 3-monooxygenase/tryptophan 5-
STK4 (serine/threonine kinase 4)
SLC12A5-AS1 (SLC12A5 and MMP9 antisense RNA 1)
SLC2A10 (solute carrier family 2 member 10)
LINC01271 (long intergenic non-protein coding RNA 1271)
VAPB (VAMP associated protein B and C)
MTG2 (mitochondrial ribosome associated GTPase 2)
LAMA5 (laminin subunit alpha 5)
UCKL1-AS1 (UCKL1 antisense RNA 1)
BTG3-AS1 (BTG3 antisense RNA 1)
ATP5PF (ATP synthase peripheral stalk subunit F6)
URB1-AS1 (URB1 antisense RNA 1 (head to head))
IL10RB-DT (IL10RB divergent transcript)
DONSON (DNA replication fork stabilization factor DONSON)
LINC00649 (long intergenic non-protein coding RNA 649)
KCNE1 (potassium voltage-gated channel subfamily E
LINC01436 (long intergenic non-protein coding RNA 1436)
SETD4-AS1 (SETD4 antisense RNA 1)
CBR3 (carbonyl reductase 3)
TTC3-AS1 (TTC3 antisense RNA 1)
LCA5L (lebercilin LCA5 like)
PLAC4 (placenta enriched 4)
MX2 (MX dynamin like GTPase 2)
UMODL1-AS1 (UMODL1 antisense RNA 1)
LINC01671 (long intergenic non-protein coding RNA 1671)
NDUFV3 (NADH:ubiquinone oxidoreductase subunit V3)
CSTB (cystatin B)
AATBC (apoptosis associated transcript in bladder cancer)
CFAP410 (cilia and flagella associated protein 410)
ITGB2-AS1 (ITGB2 antisense RNA 1)
LINC00205 (long intergenic non-protein coding RNA 205)
MCM3AP-AS1 (MCM3AP antisense RNA 1)
LINC00528 (long intergenic non-protein coding RNA 528)
MICAL3 (microtubule associated monooxygenase, calponin
DGCR11 (DiGeorge syndrome critical region gene 11)
UFD1 (ubiquitin recognition factor in ER associated
LINC01637 (long intergenic non-protein coding RNA 1637)
MAPK1 (mitogen-activated protein kinase 1)
C22orf15 (chromosome 22 open reading frame 15)
SLC2A11 (solute carrier family 2 member 11)
MIF-AS1 (MIF antisense RNA 1)
KIAA1671-AS1 (KIAA1671 antisense RNA 1)
TFIP11-DT (TFIP11 divergent transcript)
MIATNB (MIAT neighbor)
RHBDD3 (rhomboid domain containing 3)
CCDC157 (coiled-coil domain containing 157)
RNF215 (ring finger protein 215)
SLC35E4 (solute carrier family 35 member E4)
DUSP18 (dual specificity phosphatase 18)
IFT27 (intraflagellar transport 27)
NCF4-AS1 (NCF4 antisense RNA 1)
GGA1 (golgi associated, gamma adaptin ear containing, ARF
PDXP-DT (PDXP divergent transcript)
ANKRD54 (ankyrin repeat domain 54)
EIF3L (eukaryotic translation initiation factor 3 subunit L)
PLA2G6 (phospholipase A2 group VI)
DDX17 (DEAD-box helicase 17)
GTPBP1 (GTP binding protein 1)
NPTXR (neuronal pentraxin receptor)
GRAP2 (GRB2 related adaptor protein 2)
XPNPEP3 (X-prolyl aminopeptidase 3)
EP300-AS1 (EP300 antisense RNA 1)
CSDC2 (cold shock domain containing C2)
WBP2NL (WBP2 N-terminal like)
TCF20 (transcription factor 20)
A4GALT (alpha 1,4-galactosyltransferase (P blood group))
TTLL1-AS1 (TTLL1 antisense RNA 1)
PRR34 (PRR34 long non-coding RNA)
LINC01644 (long intergenic non-protein coding RNA 1644)
CRELD2 (cysteine rich with EGF like domains 2)
TYMP (thymidine phosphorylase)
KLHDC7B-DT (KLHDC7B divergent transcript)
RABL2B (RAB, member of RAS oncogene family like 2B)
CHL1-AS2 (CHL1 antisense RNA 2)
CHL1 (cell adhesion molecule L1 like)
CHL1-AS1 (CHL1 antisense RNA 1)
IL5RA (interleukin 5 receptor subunit alpha)
CRBN (cereblon)
SUMF1 (sulfatase modifying factor 1)
BHLHE40-AS1 (BHLHE40 antisense RNA 1)
BHLHE40 (basic helix-loop-helix family member e40)
SRGAP3 (SLIT-ROBO Rho GTPase activating protein 3)
THUMPD3-AS1 (THUMPD3 antisense RNA 1)
OGG1 (8-oxoguanine DNA glycosylase)
PRRT3-AS1 (PRRT3 antisense RNA 1)
VGLL4 (vestigial like family member 4)
SYN2 (synapsin II)
XPC (XPC complex subunit, DNA damage recognition and
XPC-AS1 (XPC antisense RNA 1)
NR2C2 (nuclear receptor subfamily 2 group C member 2)
MRPS25 (mitochondrial ribosomal protein S25)
SH3BP5-AS1 (SH3BP5 antisense RNA 1)
HACL1 (2-hydroxyacyl-CoA lyase 1)
ANKRD28 (ankyrin repeat domain 28)
NEK10 (NIMA related kinase 10)
CMTM7 (CKLF like MARVEL transmembrane domain
CNOT10-AS1 (CNOT10 antisense RNA 1)
LRRFIP2 (LRR binding FLII interacting protein 2)
GOLGA4-AS1 (GOLGA4 antisense RNA 1)
ITGA9-AS1 (ITGA9 antisense RNA 1)
ACAA1 (acetyl-CoA acyltransferase 1)
EIF1B (eukaryotic translation initiation factor 1B)
ENTPD3-AS1 (ENTPD3 antisense RNA 1)
CCK (cholecystokinin)
LINC02158 (long intergenic non-protein coding RNA 2158)
SNRK-AS1 (SNRK antisense RNA 1)
UMD1-AS1 (UMD1 antisense RNA 1)
CCR1 (C-C motif chemokine receptor 1)
CCR5AS (CCR5 antisense RNA)
CCR5 (C-C motif chemokine receptor 5)
KIF9-AS1 (KIF9 antisense RNA 1)
PRKAR2A-AS1 (PRKAR2A antisense RNA 1)
ARIH2 (ariadne RBR E3 ubiquitin protein ligase 2)
RHOA (ras homolog family member A)
RASSF1-AS1 (RASSF1 antisense RNA 1)
ZMYND10-AS1 (ZMYND10 antisense RNA 1)
ABHD14A (abhydrolase domain containing 14A)
ALAS1 (5'-aminolevulinate synthase 1)
SMIM4 (small integral membrane protein 4)
GLT8D1 (glycosyltransferase 8 domain containing 1)
TKT (transketolase)
DNAH12 (dynein axonemal heavy chain 12)
FAM107A (family with sequence similarity 107 member A)
PSMD6-AS2 (PSMD6 antisense RNA 2)
SLC25A26 (solute carrier family 25 member 26)
FOXP1-IT1 (FOXP1 intronic transcript 1)
ZNF717 (zinc finger protein 717)
DCBLD2 (discoidin, CUB and LCCL domain containing 2)
NIT2 (nitrilase family member 2)
ZBTB11-AS1 (ZBTB11 antisense RNA 1)
CBLB (Cbl proto-oncogene B)
C3orf52 (chromosome 3 open reading frame 52)
TBILA (TGF-beta induced lncRNA)
SLC35A5 (solute carrier family 35 member A5)
CD200R1L-AS1 (CD200R1L antisense RNA 1)

SLC25A25-AS1 (SLC25A25 antisense RNA 1)
COQ4 (coenzyme Q4)
ODF2-AS1 (ODF2 antisense RNA 1)
LINC00963 (long intergenic non-protein coding RNA 963)
FNBP1 (formin binding protein 1)
SURF1 (SURF1 cytochrome c oxidase assembly factor)
RXRA (retinoid X receptor alpha)
CCDC187 (coiled-coil domain containing 187)
CCDC183-AS1 (CCDC183 antisense RNA 1)
MAN1B1-DT (MAN1B1 divergent transcript)
FAM166A (family with sequence similarity 166 member A)
ASMTL-AS1 (ASMTL antisense RNA 1)
ARSD-AS1 (ARSD antisense RNA 1)
INE2 (inactivation escape 2)
SAT1 (spermidine/spermine N1-acetyltransferase 1)
KLHL15 (kelch like family member 15)
GK-AS1 (GK antisense RNA 1)
TAB3 (TGF-beta activated kinase 1 (MAP3K7) binding
DDX3X (DEAD-box helicase 3 X-linked)
CASK-AS1 (CASK antisense RNA 1)
SLC9A7 (solute carrier family 9 member A7)
INE1 (inactivation escape 1)
USP27X-DT (USP27X divergent transcript)
LINC01278 (long intergenic non-protein coding RNA 1278)
MSN (moesin)
OGT (O-linked N-acetylglucosamine (GlcNAc) transferase)
TSIX (TSIX transcript, XIST antisense RNA)
FTX (FTX transcript, XIST regulator)
MAGT1 (magnesium transporter 1)
PGK1 (phosphoglycerate kinase 1)
SH3BGRL (SH3 domain binding glutamate rich protein like)
SLC25A53 (solute carrier family 25 member 53)
NDUFA1 (NADH:ubiquinone oxidoreductase subunit A1)
MTMR1 (myotubularin related protein 1)
SLC6A8 (solute carrier family 6 member 8)
PRKY (protein kinase Y-linked (pseudogene))
MTND2P28 (MT-ND2 pseudogene 28)
CHCHD2P6 (coiled-coil-helix-coiled-coil-helix domain
CROCCP3 (CROCC pseudogene 3)
FTH1P1 (ferritin heavy chain 1 pseudogene 1)
CTBP2P8 (CTBP2 pseudogene 8)
NSRP1P1 (nuclear speckle splicing regulatory protein 1
PTGES3P1 (prostaglandin E synthase 3 pseudogene 1)
GBP1P1 (guanylate binding protein 1 pseudogene 1)
EEF1A1P11 (eukaryotic translation elongation factor 1
SEC63P1 (SEC63 homolog, protein translocation regulator
BCAS2P2 (BCAS2 pseudogene 2)
NDUFA4P1 (NDUFA4 pseudogene 1)
NRBF2P3 (nuclear receptor binding factor 2 pseudogene 3)
PDE4DIPP2 (PDE4DIP pseudogene 2)
EMBP1 (embigin pseudogene 1)
FCGR1CP (Fc fragment of IgG receptor Ic, pseudogene)
HYDIN2 (HYDIN axonemal central pair apparatus protein 2
SSBL4P (small RNA binding exonuclease protection factor La
CCT8P1 (chaperonin containing TCP1 subunit 8 pseudogene
H2BC19P (H2B clustered histone 19, pseudogene)
H2BC20P (H2B clustered histone 20, pseudogene)
MSTO2P (misato family member 2, pseudogene)
HSP90AA3P (heat shock protein 90 alpha family class A
SUMO1P3 (SUMO1 pseudogene 3)
SLAMF6P1 (SLAM family member 6 pseudogene 1)
PFN1P1 (profilin 1 pseudogene 1)
CRYZL2P (crystallin zeta like 2, pseudogene)
EIF1P3 (eukaryotic translation initiation factor 1
SNRPGP10 (small nuclear ribonucleoprotein polypeptide G
MORF4L1P1 (mortality factor 4 like 1 pseudogene 1)
GTF2IP20 (general transcription factor IIi pseudogene 20)
FTH1P2 (ferritin heavy chain 1 pseudogene 2)
ZNF731P (zinc finger protein 731, pseudogene)
NAMPTP1 (nicotinamide phosphoribosyltransferase
ZNF37BP (zinc finger protein 37B, pseudogene)
CAP1P2 (CAP1 pseudogene 2)
PARGP1 (poly(ADP-ribose) glycohydrolase pseudogene 1)
BMS1P1 (BMS1 pseudogene 1)
CALM2P2 (calmodulin 2 pseudogene 2)
SRP9P1 (signal recognition particle 9 pseudogene 1)
EIF2S2P3 (eukaryotic translation initiation factor 2 subunit
NPM1P25 (nucleophosmin 1 pseudogene 25)
KRT8P49 (keratin 8 pseudogene 49)
MORF4L1P3 (mortality factor 4 like 1 pseudogene 3)
ST13P5 (ST13, Hsp70 interacting protein pseudogene 5)
HSP90AA2P (heat shock protein 90 alpha family class A
SEC14L1P1 (SEC14 like 1 pseudogene 1)
SLC25A47P1 (solute carrier family 25 member 47
ALG1L8P (ALG1 like 8, pseudogene)
IFITM9P (interferon induced transmembrane protein 9
FTH1P16 (ferritin heavy chain 1 pseudogene 16)
SRP14P2 (signal recognition particle 14 pseudogene 2)
ARPC3P3 (actin related protein 2/3 complex subunit 3
DNAJB6P1 (DNAJB6 pseudogene 1)
WASH8P (WAS protein family homolog 8, pseudogene)
OTUD4P1 (OTUD4 pseudogene 1)
HSPA8P5 (heat shock protein family A (Hsp70) member 8
SRP14P1 (signal recognition particle 14 pseudogene 1)
HTR7P1 (5-hydroxytryptamine receptor 7 pseudogene 1)
EEF1A1P4 (eukaryotic translation elongation factor 1 alpha
TCP1P3 (t-complex 1 pseudogene 3)
IFITM3P2 (IFITM3 pseudogene 2)
HSPD1P4 (heat shock protein family D (Hsp60) member 1
PABPC1P4 (poly(A) binding protein cytoplasmic 1
RBMS1P1 (RNA binding motif single stranded interacting
ATP5PDP4 (ATP synthase peripheral stalk subunit d
GOLGA2P5 (GOLGA2 pseudogene 5)
HSPE1P4 (heat shock protein family E (Hsp10) member 1
CLIC1P1 (chloride intracellular channel 1 pseudogene 1)
EP400P1 (EP400 pseudogene 1)
FTH1P7 (ferritin heavy chain 1 pseudogene 7)
NUS1P2 (NUS1 pseudogene 2)
ATP5F1EP2 (ATP synthase F1 subunit epsilon pseudogene 2)
CALM2P3 (calmodulin 2 pseudogene 3)
OR7E36P (olfactory receptor family 7 subfamily E member
RCN1P2 (reticulocalbin 1 pseudogene 2)
COX17P1 (COX17 pseudogene 1)
PSME2P2 (proteasome activator subunit 2 pseudogene 2)
H3P36 (H3 histone pseudogene 36)
ATP5F1CP1 (ATP synthase F1 subunit gamma pseudogene 1)
HSPE1P2 (heat shock protein family E (Hsp10) member 1
DYNLL1P1 (dynein light chain LC8-type 1 pseudogene 1)
DYNLL1P2 (dynein light chain LC8-type 1 pseudogene 2)
HERC2P2 (hect domain and RLD 2 pseudogene 2)
HERC2P9 (hect domain and RLD 2 pseudogene 9)
HMGN2P5 (high mobility group nucleosomal binding
SORD2P (sorbitol dehydrogenase 2, pseudogene)
EEF1B2P1 (eukaryotic translation elongation factor 1 beta 2
SCAND2P (SCAN domain containing 2 pseudogene)
HSPE1P3 (heat shock protein family E (Hsp10) member 1
HSP90B2P (heat shock protein 90 beta family member 2,
SUB1P3 (SUB1 pseudogene 3)
ABCC6P2 (ATP binding cassette subfamily C member 6
ABCC6P1 (ATP binding cassette subfamily C member 6
GAPDHP35 (glyceraldehyde 3 phosphate dehydrogenase
SMG1P2 (SMG1 pseudogene 2)
BCLAF1P2 (BCL2 associated transcription factor 1
VPS35P1 (VPS35 pseudogene 1)
CES1P1 (carboxylesterase 1 pseudogene 1)
URAHP (urate (hydroxyiso-) hydrolase, pseudogene)
TXNP4 (thioredoxin pseudogene 4)
MEIS3P1 (Meis homeobox 3 pseudogene 1)
UPF3AP1 (UPF3A pseudogene 1)
MEIS3P2 (Meis homeobox 3 pseudogene 2)
TWF1P1 (twinfilin 1 pseudogene 1)
LRRC37BP1 (leucine rich repeat containing 37B pseudogene
UBL5P2 (ubiquitin like 5 pseudogene 2)
LRRC37A11P (leucine rich repeat containing 37 member
LRRC37A4P (leucine rich repeat containing 37 member A4,
LINC02210 (long intergenic non-protein coding RNA 2210)
MICOS10P2 (MICOS10 pseudogene 2)
PLEKHM1P1 (pleckstrin homology and RUN domain
LINC00674 (long intergenic non-protein coding RNA 674)
ATF4P3 (activating transcription factor 4 pseudogene 3)
LRRC37A7P (leucine rich repeat containing 37 member A7,
ZNF271P (zinc finger protein 271, pseudogene)
MTCO2P2 (MT-CO2 pseudogene 2)
SNRPGP2 (small nuclear ribonucleoprotein polypeptide G
ZNF788P (zinc finger family member 788, pseudogene)
ITGB1P1 (integrin subunit beta 1 pseudogene 1)
IPO5P1 (importin 5 pseudogene 1)
EEF1A1P7 (eukaryotic translation elongation factor 1 alpha
PRR13P5 (proline rich 13 pseudogene 5)
CYP2F2P (cytochrome P450 family 2 subfamily F member 2,
CYP2B7P (cytochrome P450 family 2 subfamily B member 7,
CYP2G2P (cytochrome P450 family 2 subfamily G member 2,
ZNF321P (zinc finger protein 321, pseudogene)
CENPBD1P1 (CENPB DNA-binding domains containing 1
TMSB4XP2 (TMSB4X pseudogene 2)
EIF1P7 (eukaryotic translation initiation factor 1
FTH1P3 (ferritin heavy chain 1 pseudogene 3)
KRTCAP2P1 (KRTCAP2 pseudogene 1)
AHSA2P (activator of HSP90 ATPase homolog 2,
PRELID1P6 (PRELID1 pseudogene 6)
MRPL36P1 (mitochondrial ribosomal protein L36
FNBP1P1 (formin binding protein 1 pseudogene 1)
EEF1A1P12 (eukaryotic translation elongation factor 1
MED15P9 (mediator complex subunit 15 pseudogene 9)
UBBP1 (ubiquitin B pseudogene 1)
YWHAEP5 (tyrosine 3-monooxygenase/tryptophan 5-
AHCTF1P1 (AT-hook containing transcription factor 1
H3P6 (H3 histone pseudogene 6)
NUDCP2 (nuclear distribution C pseudogene 2)
FTH1P20 (ferritin heavy chain 1 pseudogene 20)
CACYBPP2 (calcyclin binding protein pseudogene 2)
DNAJB1P1 (DnaJ heat shock protein family (Hsp40) member
IMPDH1P10 (inosine monophosphate dehydrogenase 1
DAZAP2P1 (DAZ associated protein 2 pseudogene 1)
HSPA9P1 (heat shock protein family A (Hsp70) member 9
BANF1P3 (BANF1 pseudogene 3)
FTLP3 (ferritin light chain pseudogene 3)
UBE2D3P1 (ubiquitin conjugating enzyme E2 D3
FRG1BP (FSHD region gene 1 family member B, pseudogene)
TSPY26P (testis specific protein Y-linked 26, pseudogene)
ARPC3P1 (actin related protein 2/3 complex subunit 3
TMSB4XP6 (TMSB4X pseudogene 6)
CCDC74BP1 (coiled-coil domain containing 74B pseudogene
CRYBB2P1 (crystallin beta B2 pseudogene 1)
TPTEP2 (TPTE pseudogene 2)
SERHL (serine hydrolase like (pseudogene))
RRP7BP (ribosomal RNA processing 7 homolog B,
OR7E122P (olfactory receptor family 7 subfamily E member
EMC3-AS1 (EMC3 antisense RNA 1)
TPRXL (tetrapeptide repeat homeobox like (pseudogene))
EEF1A1P24 (eukaryotic translation elongation factor 1
RNF7P1 (ring finger protein 7 pseudogene 1)
FTH1P23 (ferritin heavy chain 1 pseudogene 23)
ALG1L6P (ALG1 like 6, pseudogene)
HNRNPKP4 (heterogeneous nuclear ribonucleoprotein K
PDCL3P4 (PDCL3 pseudogene 4)
FTH1P4 (ferritin heavy chain 1 pseudogene 4)
HSPA8P9 (heat shock protein family A (Hsp70) member 8
ST13P15 (ST13, Hsp70 interacting protein pseudogene 15)
SNRPCP3 (small nuclear ribonucleoprotein polypeptide C

QTRT2 (queuine tRNA-ribosyltransferase accessory subunit
GSK3B (glycogen synthase kinase 3 beta)
HSPBAP1 (HSPB1 associated protein 1)
LINC02035 (long intergenic non-protein coding RNA 2035)
ALDH1L1-AS1 (ALDH1L1 antisense RNA 1)
RUVBL1 (RuvB like AAA ATPase 1)
H1-10-AS1 (H1-10 antisense RNA 1)
SNORA7B (small nucleolar RNA, H/ACA box 7B)
EFCAB12 (EF-hand calcium binding domain 12)
SLC35G2 (solute carrier family 35 member G2)
CHST2 (carbohydrate sulfotransferase 2)
HLTF-AS1 (HLTF antisense RNA 1)
ANKUB1 (ankyrin repeat and ubiquitin domain containing
SERP1 (stress associated endoplasmic reticulum protein 1)
SELENOT (selenoprotein T)
DHX36 (DEAH-box helicase 36)
MFSD1 (major facilitator superfamily domain containing 1)
IFT80 (intraflagellar transport 80)
LINC02067 (long intergenic non-protein coding RNA 2067)
TMEM212 (transmembrane protein 212)
CCDC39 (coiled-coil domain containing 39)
FXR1 (FMR1 autosomal homolog 1)
LINC02031 (long intergenic non-protein coding RNA 2031)
ABCC5 (ATP binding cassette subfamily C member 5)
AP2M1 (adaptor related protein complex 2 subunit mu 1)
MAP3K13 (mitogen-activated protein kinase kinase kinase
TRA2B (transformer 2 beta homolog)
EIF4A2 (eukaryotic translation initiation factor 4A2)
BCL6 (BCL6 transcription repressor)
LPP-AS2 (LPP antisense RNA 2)
TPRG1-AS1 (TPRG1 antisense RNA 1)
TMEM44-AS1 (TMEM44 antisense RNA 1)
TMEM44 (transmembrane protein 44)
MUC20-OT1 (MUC20 overlapping transcript)
MUC4 (mucin 4, cell surface associated)
PCYT1A (phosphate cytidylyltransferase 1A, choline)
SLC51A (solute carrier family 51 subunit alpha)
PIGX (phosphatidylinositol glycan anchor biosynthesis class
NCBP2-AS1 (NCBP2 antisense RNA 1)
DLG1 (discs large MAGUK scaffold protein 1)
LMLN-AS1 (LMLN antisense RNA 1)
GAK (cyclin G associated kinase)
SPON2 (spondin 2)
CTBP1-AS (CTBP1 antisense RNA)
CTBP1-DT (CTBP1 divergent transcript)
MXD4 (MAX dimerization protein 4)
ADD1 (adducin 1)
NOP14-AS1 (NOP14 antisense RNA 1)
GRK4 (G protein-coupled receptor kinase 4)
RGS12 (regulator of G protein signaling 12)
MRFAP1L1 (Morf4 family associated protein 1 like 1)
TADA2B (transcriptional adaptor 2B)
HS3ST1 (heparan sulfate-glucosamine 3-sulfotransferase 1)
BOD1L1 (biorientation of chromosomes in cell division 1
TAPT1-AS1 (TAPT1 antisense RNA 1 (head to head))
CCDC149 (coiled-coil domain containing 149)
SEPSECS-AS1 (SEPSECS antisense RNA 1 (head to head))
STIM2 (stromal interaction molecule 2)
PCDH7 (protocadherin 7)
DTHD1 (death domain containing 1)
KLF3-AS1 (KLF3 antisense RNA 1)
UGDH (UDP-glucose 6-dehydrogenase)
UGDH-AS1 (UGDH antisense RNA 1)
RHOH (ras homolog family member H)
LINC02265 (long intergenic non-protein coding RNA 2265)
RBM47 (RNA binding motif protein 47)
YIPF7 (Yip1 domain family member 7)
SGCB (sarcoglycan beta)
SULT1E1 (sulfotransferase family 1E member 1)
CXCL5 (C-X-C motif chemokine ligand 5)
SHROOM3-AS1 (SHROOM3 antisense RNA 1)
BMP2K (BMP2 inducible kinase)
THAP9-AS1 (THAP9 antisense RNA 1)
PLAC8 (placenta associated 8)
WDFY3 (WD repeat and FYVE domain containing 3)
ARHGAP24 (Rho GTPase activating protein 24)
MAPK10 (mitogen-activated protein kinase 10)
TIGD2 (tigger transposable element derived 2)
BMPR1B-DT (BMPR1B divergent transcript)
ADH5 (alcohol dehydrogenase 5 (class III), chi polypeptide)
TRMT10A (tRNA methyltransferase 10A)
H2AZ1-DT (H2AZ1 divergent transcript)
UBE2D3 (ubiquitin conjugating enzyme E2 D3)
CXXC4 (CXXC finger protein 4)
ALPK1 (alpha kinase 1)
MIR302CHG (miR-302/367 cluster host gene)
SNHG8 (small nucleolar RNA host gene 8)
USP53 (ubiquitin specific peptidase 53)
HSPA4L (heat shock protein family A (Hsp70) member 4 like)
NAA15 (N-alpha-acetyltransferase 15, NatA auxiliary
RAB33B-AS1 (RAB33B antisense RNA 1)
SETD7 (SET domain containing 7, histone lysine
SMARCA5-AS1 (SMARCA5 antisense RNA 1)
OTUD4 (OTU deubiquitinase 4)
SMAD1-AS1 (SMAD1 antisense RNA 1)
SH3D19 (SH3 domain containing 19)
MIR4453HG (MIR4453 host gene)
ETFDH (electron transfer flavoprotein dehydrogenase)
ING2 (inhibitor of growth family member 2)
LINC02363 (long intergenic non-protein coding RNA 2363)
IRF2-DT (IRF2 divergent transcript)
ANKRD37 (ankyrin repeat domain 37)
CEP72 (centrosomal protein 72)
TPPP (tubulin polymerization promoting protein)
NDUFS6 (NADH:ubiquinone oxidoreductase subunit S6)
TENT4A (terminal nucleotidyltransferase 4A)
SEMA5A (semaphorin 5A)
SNHG18 (small nucleolar RNA host gene 18)
CCT5 (chaperonin containing TCP1 subunit 5)
LINC01513 (long intergenic non-protein coding RNA 1513)
GOLPH3-DT (GOLPH3 divergent transcript)
SUB1 (SUB1 regulator of transcription)
TTC23L-AS1 (TTC23L antisense RNA 1)
RAD1 (RAD1 checkpoint DNA exonuclease)
NIPBL-DT (NIPBL divergent transcript)
LIFR (LIF receptor subunit alpha)
NNT-AS1 (NNT antisense RNA 1)
MRPS30 (mitochondrial ribosomal protein S30)
SMIM15-AS1 (SMIM15 antisense RNA 1)
DIMT1 (DIM1 rRNA methyltransferase and ribosome
SREK1 (splicing regulatory glutamic acid and lysine rich
MAST4-AS1 (MAST4 antisense RNA 1)
MARVELD2 (MARVEL domain containing 2)
BTF3-DT (BTF3 divergent transcript)
LINC02122 (long intergenic non-protein coding RNA 2122)
ENC1 (ectodermal-neural cortex 1)
HEXB (hexosaminidase subunit beta)
NSA2 (NSA2 ribosome biogenesis factor)
ATG10 (autophagy related 10)
VCAN-AS1 (VCAN antisense RNA 1)
ADGRV1 (adhesion G protein-coupled receptor V1)
ARRDC3 (arrestin domain containing 3)
LNPEP (leucyl and cystinyl aminopeptidase)
LIX1-AS1 (LIX1 and RIOK2 antisense RNA 1)
LINC01023 (long intergenic non-protein coding RNA 1023)
FER (FER tyrosine kinase)
STARD4-AS1 (STARD4 antisense RNA 1)
DCP2 (decapping mRNA 2)
LINC00992 (long intergenic non-protein coding RNA 992)
MIR1244-2 (microRNA 1244-2)
PPIC-AS1 (PPIC antisense RNA 1)
P4HA2-AS1 (P4HA2 antisense RNA 1)
SLC22A5 (solute carrier family 22 member 5)
IRF1-AS1 (IRF1 antisense RNA 1)
IRF1 (interferon regulatory factor 1)
MIR3661 (microRNA 3661)
PPP2CA-DT (PPP2CA divergent transcript)
LINC01843 (long intergenic non-protein coding RNA 1843)
SAR1B (secretion associated Ras related GTPase 1B)
PITX1 (paired like homeodomain 1)
MACROH2A1 (macroH2A.1 histone)
PKD2L2 (polycystin 2 like 2, transient receptor potential
EGR1 (early growth response 1)
SNHG4 (small nucleolar RNA host gene 4)
CYSTM1 (cysteine rich transmembrane module containing
SRA1 (steroid receptor RNA activator 1)
IK (IK cytokine)
WDR55 (WD repeat domain 55)
HDAC3 (histone deacetylase 3)
DELE1 (DAP3 binding cell death enhancer 1)
ARHGAP26 (Rho GTPase activating protein 26)
ADRB2 (adrenoceptor beta 2)
GRPEL2-AS1 (GRPEL2 antisense RNA 1)
ARHGEF37 (Rho guanine nucleotide exchange factor 37)
TNIP1 (TNFAIP3 interacting protein 1)
ATOX1 (antioxidant 1 copper chaperone)
G3BP1 (G3BP stress granule assembly factor 1)
MED7 (mediator complex subunit 7)
FABP6-AS1 (FABP6 antisense RNA 1)
C5orf58 (chromosome 5 open reading frame 58)
ERGIC1 (endoplasmic reticulum-golgi intermediate
GRK6 (G protein-coupled receptor kinase 6)
PHYKPL (5-phosphohydroxy-L-lysine phospho-lyase)
ZNF354A (zinc finger protein 354A)
RUFY1-AS1 (RUFY1 antisense RNA 1)
MRNIP (MRN complex interacting protein)
RNF130 (ring finger protein 130)
FOXC1 (forkhead box C1)
SERPINB1 (serpin family B member 1)
SLC22A23 (solute carrier family 22 member 23)
C6orf201 (chromosome 6 open reading frame 201)
LYRM4 (LYR motif containing 4)
EDN1 (endothelin 1)
ATXN1 (ataxin 1)
NUP153-AS1 (NUP153 antisense RNA 1)
GPLD1 (glycosylphosphatidylinositol specific phospholipase
HCG11 (HLA complex group 11)
ZNF165 (zinc finger protein 165)
ZSCAN16-AS1 (ZSCAN16 antisense RNA 1)
HCG14 (HLA complex group 14)
HLA-F (major histocompatibility complex, class I, F)
HLA-A (major histocompatibility complex, class I, A)
TRIM31-AS1 (TRIM31 antisense RNA 1)
HCG18 (HLA complex group 18)
DHX16 (DEAH-box helicase 16)
TUBB (tubulin beta class I)
MICA (MHC class I polypeptide-related sequence A)
DDX39B (DExD-box helicase 39B)
TNF (tumor necrosis factor)
C6orf47-AS1 (C6orf47 antisense RNA 1)
CLIC1 (chloride intracellular channel 1)
SAPCD1-AS1 (SAPCD1 antisense RNA 1)
EHMT2-AS1 (EHMT2 and SLC44A4 antisense RNA 1)
STK19 (serine/threonine kinase 19)
PPT2-EGFL8 (PPT2-EGFL8 readthrough (NMD candidate))
HLA-DRB1 (major histocompatibility complex, class II, DR
HLA-DQB1-AS1 (HLA-DQB1 antisense RNA 1)
HLA-DQA2 (major histocompatibility complex, class II, DQ
HLA-DOA (major histocompatibility complex, class II, DO
ILRUN-AS1 (ILRUN antisense RNA 1)

AADACP1 (arylacetamide deacetylase pseudogene 1)
BRD7P2 (bromodomain containing 7 pseudogene 2)
KRT8P12 (keratin 8 pseudogene 12)
LRRFIP1P1 (LRR binding FLII interacting protein 1
RNA5SP151 (RNA, 5S ribosomal pseudogene 151)
EEF1A1P8 (eukaryotic translation elongation factor 1 alpha
FAM86EP (family with sequence similarity 86, member A
CDC42P6 (cell division cycle 42 pseudogene 6)
ARL4AP2 (ADP ribosylation factor like GTPase 4A
EXOC5P1 (exocyst complex component 5 pseudogene 1)
IFITM3P1 (IFITM3 pseudogene 1)
HSP90AB3P (heat shock protein 90 alpha family class B
TMSB4XP8 (TMSB4X pseudogene 8)
EEF1A1P9 (eukaryotic translation elongation factor 1 alpha
H3P14 (H3 histone pseudogene 14)
CEP170P1 (centrosomal protein 170 pseudogene 1)
GTF2IP12 (general transcription factor IIi pseudogene 12)
SEPTIN7P14 (septin 7 pseudogene 14)
H3P16 (H3 histone pseudogene 16)
MORF4 (mortality factor 4 (pseudogene))
MYL12BP2 (MYL12B pseudogene 2)
EEF1A1P13 (eukaryotic translation elongation factor 1
FTH1P10 (ferritin heavy chain 1 pseudogene 10)
HSPD1P1 (heat shock protein family D (Hsp60) member 1
EEF1A1P19 (eukaryotic translation elongation factor 1
NAIPP2 (NAIP pseudogene 2)
RAP1BL (RAP1B like (pseudogene))
CTBP2P4 (CTBP2 pseudogene 4)
HNRNPKP1 (heterogeneous nuclear ribonucleoprotein K
MTND5P11 (MT-ND5 pseudogene 11)
GAPDHP40 (glyceraldehyde 3 phosphate dehydrogenase
ASS1P1 (argininosuccinate synthetase 1 pseudogene 1)
CMAHP (cytidine monophospho-N-acetylneuraminic acid
ZNF204P (zinc finger protein 204, pseudogene)
SUMO2P1 (SUMO2 pseudogene 1)
HCG4 (HLA complex group 4)
HLA-H (major histocompatibility complex, class I, H
HLA-K (major histocompatibility complex, class I, K
ZNRD1ASP (zinc ribbon domain containing 1 antisense,
LAP3P2 (leucine aminopeptidase 3 pseudogene 2)
COX6A1P2 (cytochrome c oxidase subunit 6A1 pseudogene
FTH1P5 (ferritin heavy chain 1 pseudogene 5)
FTH1P15 (ferritin heavy chain 1 pseudogene 15)
ADH5P4 (ADH5 pseudogene 4)
TPT1P6 (tumor protein, translationally-controlled 1
BDH2P1 (3-hydroxybutyrate dehydrogenase 2, pseudogene
ATP5MFP2 (ATP synthase membrane subunit f pseudogene
TPT1P4 (tumor protein, translationally-controlled 1
PDCL3P5 (PDCL3 pseudogene 5)
ALG1L5P (ALG1 like 5, pseudogene)
HSPA8P8 (heat shock protein family A (Hsp70) member 8
EEF1A1P6 (eukaryotic translation elongation factor 1 alpha
S100A11P2 (S100A11 pseudogene 2)
RWDD4P2 (RWD domain containing 4 pseudogene 2)
TCP1P1 (t-complex 1 pseudogene 1)
GTF2IP13 (general transcription factor IIi pseudogene 13)
FKBP9P1 (FKBP prolyl isomerase 9 pseudogene 1)
PSPHP1 (phosphoserine phosphatase pseudogene 1)
IFITM3P4 (IFITM3 pseudogene 4)
YWHAEP1 (tyrosine 3-monooxygenase/tryptophan 5-
GTF2IRD1P1 (GTF2I repeat domain containing 1
STAG3L4 (stromal antigen 3-like 4 (pseudogene))
STAG3L3 (stromal antigen 3-like 3 (pseudogene))
ABHD11-AS1 (ABHD11 antisense RNA 1 (tail to tail))
NCF1C (neutrophil cytosolic factor 1C pseudogene)
EIF4BP6 (eukaryotic translation initiation factor 4B
YBX1P2 (Y-box binding protein 1 pseudogene 2)
ST13P7 (ST13, Hsp70 interacting protein pseudogene 7)
PCBP2P2 (poly(rC) binding protein 2 pseudogene 2)
FTH1P11 (ferritin heavy chain 1 pseudogene 11)
CPP (ceruloplasmin pseudogene)
TAGLN2P1 (transgelin 2 pseudogene 1)
ST13P6 (ST13, Hsp70 interacting protein pseudogene 6)
SMPD5 (sphingomyelin phosphodiesterase 5 (pseudogene))
ZNF252P (zinc finger protein 252, pseudogene)
FTH1P12 (ferritin heavy chain 1 pseudogene 12)
GLULP4 (glutamate-ammonia ligase pseudogene 4)
FAM27B (family with sequence similarity 27 member B)
EEF1DP2 (eukaryotic translation elongation factor 1 delta
EIF4BP3 (eukaryotic translation initiation factor 4B
LRRC37A5P (leucine rich repeat containing 37 member A5,
TPT1P9 (tumor protein, translationally-controlled 1
CUTALP (cutA divalent cation tolerance like, pseudogene)
FTLP2 (ferritin light chain pseudogene 2)
TBCAP1 (tubulin folding cofactor A pseudogene 1)
SDCBPP3 (syndecan binding protein pseudogene 3)
RBM39P1 (RBM39 pseudogene 1)
CHMP5P1 (CHMP5 pseudogene 1)
EIF4A2P4 (eukaryotic translation initiation factor 4A2
PGK1P1 (phosphoglycerate kinase 1 pseudogene 1)
SHISA5P2 (SHISA5 pseudogene 2)
TTC3P1 (tetratricopeptide repeat domain 3 pseudogene 1)
STIP1P3 (stress induced phosphoprotein 1 pseudogene 3)
ST13P18 (ST13, Hsp70 interacting protein pseudogene 18)
GK4P (glycerol kinase 4 pseudogene)
DNAJA1P3 (DnaJ heat shock protein family (Hsp40) member
EIF4BP7 (eukaryotic translation initiation factor 4B
RHOXF1P3 (Rhox homeobox family member 1 pseudogene
HSPA8P1 (heat shock protein family A (Hsp70) member 8
H3P47 (H3 histone pseudogene 47)
FTH1P8 (ferritin heavy chain 1 pseudogene 8)
IKBKGP1 (inhibitor of nuclear factor kappa B kinase subunit
CD24P4 (CD24 molecule pseudogene 4)

LHFPL5 (LHFPL tetraspan subfamily member 5)
PANDAR (promoter of CDKN1A antisense DNA damage
MTCH1 (mitochondrial carrier 1)
GLO1 (glyoxalase I)
KIF6 (kinesin family member 6)
CNPY3 (canopy FGF signaling regulator 3)
GNMT (glycine N-methyltransferase)
RRP36 (ribosomal RNA processing 36)
KLC4-AS1 (KLC4 antisense RNA 1)
POLR1C (RNA polymerase I and III subunit C)
ANKRD66 (ankyrin repeat domain 66)
CD2AP-DT (CD2AP divergent transcript)
GCLC (glutamate-cysteine ligase catalytic subunit)
DST (dystonin)
FKBP1C (FKBP prolyl isomerase family member 1C)
SCAT8 (S-phase cancer associated transcript 8)
EEF1A1 (eukaryotic translation elongation factor 1 alpha 1)
IRAK1BP1 (interleukin 1 receptor associated kinase 1
PHIP (pleckstrin homology domain interacting protein)
HMGN3 (high mobility group nucleosomal binding domain
HMGN3-AS1 (HMGN3 antisense RNA 1)
DOP1A (DOP1 leucine zipper like protein A)
PNRC1 (proline rich nuclear receptor coactivator 1)
MANEA-DT (MANEA divergent transcript)
TSTD3 (thiosulfate sulfurtransferase like domain containing
DSE (dermatan sulfate epimerase)
CALHM6-AS1 (CALHM6 antisense RNA 1)
MCM9 (minichromosome maintenance 9 homologous
HDDC2 (HD domain containing 2)
VNN3 (vanin 3)
LINC00271 (long intergenic non-protein coding RNA 271)
PEX7 (peroxisomal biogenesis factor 7)
WAKMAR2 (wound and keratinocyte migration associated
TNFAIP3 (TNF alpha induced protein 3)
UTRN (utrophin)
LRP11 (LDL receptor related protein 11)
RAET1E-AS1 (RAET1E antisense RNA 1)
MIR3918 (microRNA 3918)
EZR-AS1 (EZR antisense RNA 1)
RSPH3 (radial spoke head 3)
SOD2 (superoxide dismutase 2)
MRPL18 (mitochondrial ribosomal protein L18)
IGF2R (insulin like growth factor 2 receptor)
MAP3K4-AS1 (MAP3K4 antisense RNA 1)
AGPAT4 (1-acylglycerol-3-phosphate O-acyltransferase 4)
DYNLT2 (dynein light chain Tctex-type 2)
PSMB1 (proteasome 20S subunit beta 1)
SUN1 (Sad1 and UNC84 domain containing 1)
C7orf50 (chromosome 7 open reading frame 50)
MRM2 (mitochondrial rRNA methyltransferase 2)
MIOS-DT (MIOS divergent transcript)
TSPAN13 (tetraspanin 13)
AGR2 (anterior gradient 2, protein disulphide isomerase
LINC02888 (long intergenic non-protein coding RNA 2888)
SP8 (Sp8 transcription factor)
GPNMB (glycoprotein nmb)
TRA2A (transformer 2 alpha homolog)
PRR15 (proline rich 15)
GARS1-DT (GARS1 divergent transcript)
LINC00997 (long intergenic non-protein coding RNA 997)
HERPUD2-AS1 (HERPUD2 antisense RNA 1)
SEPTIN7 (septin 7)
TRGC2 (T cell receptor gamma constant 2)
CDK13 (cyclin dependent kinase 13)
POLR2J4 (RNA polymerase II subunit J4, pseudogene)
DBNL (drebrin like)
POLM (DNA polymerase mu)
DDX56 (DEAD-box helicase 56)
PPIA (peptidylprolyl isomerase A)
ABCA13 (ATP binding cassette subfamily A member 13)
EGFR-AS1 (EGFR antisense RNA 1)
ZNF273 (zinc finger protein 273)
ERV3-1 (endogenous retrovirus group 3 member 1,
LINC00174 (long intergenic non-protein coding RNA 174)
LINC02604 (long intergenic non-protein coding RNA 2604)
AUTS2 (activator of transcription and developmental
BUD23 (BUD23 rRNA methyltransferase and ribosome
CLDN3 (claudin 3)
ELN-AS1 (ELN antisense RNA 1)
GTF2IRD1 (GTF2I repeat domain containing 1)
MDH2 (malate dehydrogenase 2)
DTX2P1-UPK3BP1-PMS2P11 (DTX2P1-UPK3BP1-PMS2P11
DDX3ILA1 (DDX3 interacting lncRNA 1)
SRI-AS1 (SRI antisense RNA 1)
AKAP9 (A-kinase anchoring protein 9)
CYP51A1 (cytochrome P450 family 51 subfamily A member
GNGT1 (G protein subunit gamma transducin 1)
GNG11 (G protein subunit gamma 11)
PDAP1 (PDGFA associated protein 1)
ZNF394 (zinc finger protein 394)
FAM200A (family with sequence similarity 200 member A)
SPDYE3 (speedy/RINGO cell cycle regulator family member
PILRB (paired immunoglobin like type 2 receptor beta)
PCOLCE-AS1 (PCOLCE antisense RNA 1)
MUC12 (mucin 12, cell surface associated)
EMSLR (E2F1 mRNA stabilizing lncRNA)
POLR2J2 (RNA polymerase II subunit J2)
KMT2E-AS1 (KMT2E antisense RNA 1)
EFCAB10 (EF-hand calcium binding domain 10)
NAMPT (nicotinamide phosphoribosyltransferase)
CBLL1 (Cbl proto-oncogene like 1)
CAV2 (caveolin 2)
ST7-AS1 (ST7 antisense RNA 1)
ST7 (suppression of tumorigenicity 7)
ST7-AS2 (ST7 antisense RNA 2)
FEZF1-AS1 (FEZF1 antisense RNA 1)
FEZF1 (FEZ family zinc finger 1)
WASL-DT (WASL divergent transcript)
GCC1 (GRIP and coiled-coil domain containing 1)
SND1-IT1 (SND1 intronic transcript 1)
CALU (calumenin)
LINC-PINT (long intergenic non-protein coding RNA, p53
CHCHD3 (coiled-coil-helix-coiled-coil-helix domain
LRGUK (leucine rich repeats and guanylate kinase domain
AGBL3 (AGBL carboxypeptidase 3)
CYREN (cell cycle regulator of NHEJ)
MTPN (myotrophin)
TBXAS1 (thromboxane A synthase 1)
KDM7A-DT (KDM7A divergent transcript)
NDUFB2-AS1 (NDUFB2 antisense RNA 1)
BRAF (B-Raf proto-oncogene, serine/threonine kinase)
MGAM (maltase-glucoamylase)
TRBC1 (T cell receptor beta constant 1)
TRBC2 (T cell receptor beta constant 2)
GSTK1 (glutathione S-transferase kappa 1)
EPHA1 (EPH receptor A1)
GHET1 (gastric carcinoma proliferation enhancing transcript
ATP6V0E2-AS1 (ATP6V0E2 antisense RNA 1)
REPIN1-AS1 (REPIN1 antisense RNA 1)
WDR86-AS1 (WDR86 antisense RNA 1)
PRKAG2-AS1 (PRKAG2 antisense RNA 1)
LINC01003 (long intergenic non-protein coding RNA 1003)
PAXIP1-DT (PAXIP1 divergent transcript)
NAT1 (N-acetyltransferase 1)
R3HCC1 (R3H domain and coiled-coil containing 1)
SLC25A37 (solute carrier family 25 member 37)
BNIP3L (BCL2 interacting protein 3 like)
PTK2B (protein tyrosine kinase 2 beta)
HMBOX1 (homeobox containing 1)
LINC00589 (long intergenic non-protein coding RNA 589)
GSR (glutathione-disulfide reductase)
DDHD2 (DDHD domain containing 2)
PLPP5 (phospholipid phosphatase 5)
PLEKHA2 (pleckstrin homology domain containing A2)
SLC20A2 (solute carrier family 20 member 2)
RP1 (RP1 axonemal microtubule associated)
SDCBP (syndecan binding protein)
ASPH (aspartate beta-hydroxylase)
YTHDF3-DT (YTHDF3 divergent transcript)
ARFGEF1 (ADP ribosylation factor guanine nucleotide
LACTB2-AS1 (LACTB2 antisense RNA 1)
RDH10 (retinol dehydrogenase 10)
IL7 (interleukin 7)
LINC02605 (long intergenic non-protein coding RNA 2605)
FABP5 (fatty acid binding protein 5)
CA2 (carbonic anhydrase 2)
TMEM67 (transmembrane protein 67)
LINC02894 (long intergenic non-protein coding RNA 2894)
YWHAZ (tyrosine 3-monooxygenase/tryptophan 5-
DCAF13 (DDB1 and CUL4 associated factor 13)
EIF3H (eukaryotic translation initiation factor 3 subunit H)
SAMD12 (sterile alpha motif domain containing 12)
TBC1D31 (TBC1 domain family member 31)
TATDN1 (TatD DNase domain containing 1)
NDUFB9 (NADH:ubiquinone oxidoreductase subunit B9)
WASHC5-AS1 (WASHC5 antisense RNA 1)
PCAT1 (prostate cancer associated transcript 1)
CASC11 (cancer susceptibility 11)
LY6E-DT (LY6E divergent transcript)
RHPN1-AS1 (RHPN1 antisense RNA 1 (head to head))
EEF1D (eukaryotic translation elongation factor 1 delta)
IQANK1 (IQ motif and ankyrin repeat containing 1)
PARP10 (poly(ADP-ribose) polymerase family member 10)
CYHR1 (cysteine and histidine rich 1)
ZNF252P-AS1 (ZNF252P antisense RNA 1)
SMARCA2 (SWI/SNF related, matrix associated, actin
RFX3-AS1 (RFX3 antisense RNA 1)
GLIS3-AS1 (GLIS3 antisense RNA 1)
SPATA6L (spermatogenesis associated 6 like)
ACO1 (aconitase 1)
BAG1 (BAG cochaperone 1)
VCP (valosin containing protein)
GLIPR2 (GLI pathogenesis related 2)
ZBTB5 (zinc finger and BTB domain containing 5)
SLC25A51 (solute carrier family 25 member 51)
PTAR1 (protein prenyltransferase alpha subunit repeat
C9orf85 (chromosome 9 open reading frame 85)
LINC01504 (long intergenic non-protein coding RNA 1504)
ALDH1A1 (aldehyde dehydrogenase 1 family member A1)
ANXA1 (annexin A1)
NMRK1 (nicotinamide riboside kinase 1)
NTRK2 (neurotrophic receptor tyrosine kinase 2)
SECISBP2 (SECIS binding protein 2)
MIRLET7A1HG (miRlet-7a-1/let-7f-1/let-7d cluster host
AOPEP (aminopeptidase O (putative))
HSD17B3-AS1 (HSD17B3 antisense RNA 1)
MFSD14C (major facilitator superfamily domain containing
NCBP1 (nuclear cap binding protein subunit 1)
SEC61B (SEC61 translocon subunit beta)
TEX10 (testis expressed 10)
SLC31A1 (solute carrier family 31 member 1)
PRPF4 (pre-mRNA processing factor 4)
C9orf43 (chromosome 9 open reading frame 43)
RGS3 (regulator of G protein signaling 3)
GSN-AS1 (GSN antisense RNA 1)
GSN (gelsolin)
STRBP (spermatid perinuclear RNA binding protein)
HSPA5 (heat shock protein family A (Hsp70) member 5)
PTRH1 (peptidyl-tRNA hydrolase 1 homolog)
AK1 (adenylate kinase 1)

DPM2 (dolichyl-phosphate mannosyltransferase subunit 2,
SLC25A25-AS1 (SLC25A25 antisense RNA 1)
SWI5 (SWI5 homologous recombination repair protein)
COQ4 (coenzyme Q4)
ODF2-AS1 (ODF2 antisense RNA 1)
PTPA (protein phosphatase 2 phosphatase activator)
LINC00963 (long intergenic non-protein coding RNA 963)
USP20 (ubiquitin specific peptidase 20)
FNBP1 (formin binding protein 1)
FUBP3 (far upstream element binding protein 3)
ABO (ABO, alpha 1-3-N-acetylgalactosaminyltransferase and
SURF1 (SURF1 cytochrome c oxidase assembly factor)
RXRA (retinoid X receptor alpha)
CCDC187 (coiled-coil domain containing 187)
CCDC183-AS1 (CCDC183 antisense RNA 1)
PHPT1 (phosphohistidine phosphatase 1)
MAN1B1-DT (MAN1B1 divergent transcript)
FAM166A (family with sequence similarity 166 member A)
SLC25A6 (solute carrier family 25 member 6)
ASMTL-AS1 (ASMTL antisense RNA 1)
ARSD-AS1 (ARSD antisense RNA 1)
INE2 (inactivation escape 2)
PHKA2-AS1 (PHKA2 antisense RNA 1)
KLHL15 (kelch like family member 15)
GK-AS1 (GK antisense RNA 1)
MED14OS (MED14 opposite strand)
CASK-AS1 (CASK antisense RNA 1)
SLC9A7 (solute carrier family 9 member A7)
UBA1 (ubiquitin like modifier activating enzyme 1)
INE1 (inactivation escape 1)
UXT-AS1 (UXT antisense RNA 1)
USP27X-DT (USP27X divergent transcript)
KANTR (KDM5C adjacent transcript)
LINC01278 (long intergenic non-protein coding RNA 1278)
MSN (moesin)
OGT (O-linked N-acetylglucosamine (GlcNAc) transferase)
JPX (JPX transcript, XIST activator)
FTX (FTX transcript, XIST regulator)
MAGT1 (magnesium transporter 1)
PGK1 (phosphoglycerate kinase 1)
SH3BGRL (SH3 domain binding glutamate rich protein like)
ARMCX3-AS1 (ARMCX3 antisense RNA 1)
MORF4L2-AS1 (MORF4L2 antisense RNA 1)
SLC25A53 (solute carrier family 25 member 53)
SLC25A5-AS1 (SLC25A5 antisense RNA 1)
NDUFA1 (NADH:ubiquinone oxidoreductase subunit A1)
MBNL3 (muscleblind like splicing regulator 3)
MTMR1 (myotubularin related protein 1)
SLC6A8 (solute carrier family 6 member 8)
IKBKG (inhibitor of nuclear factor kappa B kinase regulatory
F8 (coagulation factor VIII)
PRKY (protein kinase Y-linked (pseudogene))
WASH7P (WASP family homolog 7, pseudogene)
WASH9P (WAS protein family homolog 9, pseudogene)
MTND1P23 (MT-ND1 pseudogene 23)
MTND2P28 (MT-ND2 pseudogene 28)
MTCO1P12 (MT-CO1 pseudogene 12)
MTCO2P12 (MT-CO2 pseudogene 12)
MTATP6P1 (MT-ATP6 pseudogene 1)
MTCO3P12 (MT-CO3 pseudogene 12)
HMGN2P17 (high mobility group nucleosomal binding
CHCHD2P6 (coiled-coil-helix-coiled-coil-helix domain
MT1XP1 (metallothionein 1X pseudogene 1)
MST1P2 (macrophage stimulating 1 pseudogene 2)
EIF1AXP1 (EIF1AX pseudogene 1)
NPM1P39 (nucleophosmin 1 pseudogene 39)
FTH1P1 (ferritin heavy chain 1 pseudogene 1)
HSPA5P1 (HSPA5 pseudogene 1)
KRT8P47 (keratin 8 pseudogene 47)
CTBP2P8 (CTBP2 pseudogene 8)
GDI2P2 (GDP dissociation inhibitor 2 pseudogene 2)
TPI1P1 (triosephosphate isomerase 1 pseudogene 1)
NEDD8P1 (NEDD8 pseudogene 1)
PTGES3P1 (prostaglandin E synthase 3 pseudogene 1)
GBP1P1 (guanylate binding protein 1 pseudogene 1)
EEF1A1P11 (eukaryotic translation elongation factor 1
SEC63P1 (SEC63 homolog, protein translocation regulator
BCAS2P2 (BCAS2 pseudogene 2)
PKMP1 (pyruvate kinase M1/2 pseudogene 1)
NAP1L4P1 (nucleosome assembly protein 1 like 4
PDE4DIPP2 (PDE4DIP pseudogene 2)
EMBP1 (embigin pseudogene 1)
FCGR1CP (Fc fragment of IgG receptor Ic, pseudogene)
SEC22B4P (SEC22 homolog B4, pseudogene)
HYDIN2 (HYDIN axonemal central pair apparatus protein 2
SSBL4P (small RNA binding exonuclease protection factor La
PDIA3P1 (protein disulfide isomerase family A member 3
CCT8P1 (chaperonin containing TCP1 subunit 8 pseudogene
H2BC19P (H2B clustered histone 19, pseudogene)
H2BC20P (H2B clustered histone 20, pseudogene)
MSTO2P (misato family member 2, pseudogene)
KRT8P45 (keratin 8 pseudogene 45)
ELL2P1 (elongation factor for RNA polymerase II 2
HSP90AA3P (heat shock protein 90 alpha family class A
FMO6P (flavin containing dimethylaniline monoxygenase 6,
PFN1P1 (profilin 1 pseudogene 1)
CRYZL2P (crystallin zeta like 2, pseudogene)
EIF1P3 (eukaryotic translation initiation factor 1
KRT18P28 (keratin 18 pseudogene 28)
SNRPGP10 (small nuclear ribonucleoprotein polypeptide G
MORF4L1P1 (mortality factor 4 like 1 pseudogene 1)
ACTBP11 (ACTB pseudogene 11)
FTH1P2 (ferritin heavy chain 1 pseudogene 2)
ZNF731P (zinc finger protein 731, pseudogene)
KRT8P37 (keratin 8 pseudogene 37)
HNRNPRP1 (heterogeneous nuclear ribonucleoprotein R
SVIL-AS1 (SVIL antisense RNA 1)
PPIAP31 (peptidylprolyl isomerase A pseudogene 31)
NAMPTP1 (nicotinamide phosphoribosyltransferase
EIF3LP3 (eukaryotic translation initiation factor 3 subunit L
ZNF37BP (zinc finger protein 37B, pseudogene)
EIF3LP2 (eukaryotic translation initiation factor 3 subunit L
HNRNPA3P1 (heterogeneous nuclear ribonucleoprotein A3
CEP164P1 (centrosomal protein 164 pseudogene 1)
PARGP1 (poly(ADP-ribose) glycohydrolase pseudogene 1)
SHLD2P1 (shieldin complex subunit 2 pseudogene 1)
BMS1P1 (BMS1 pseudogene 1)
DYNC1I2P1 (dynein cytoplasmic 1 intermediate chain 2
SHQ1P1 (SHQ1, H/ACA ribonucleoprotein assembly factor
DBF4P1 (DBF4 zinc finger pseudogene 1)
TMEM14DP (transmembrane protein 14D, pseudogene)
COX20P1 (COX20 Cox2 chaperone homolog (S. cerevisiae)
CALM2P2 (calmodulin 2 pseudogene 2)
NPM1P24 (nucleophosmin 1 pseudogene 24)
EIF5AP4 (eukaryotic translation initiation factor 5A
SRP9P1 (signal recognition particle 9 pseudogene 1)
NHP2P1 (NHP2 ribonucleoprotein pseudogene 1)
EIF2S2P3 (eukaryotic translation initiation factor 2 subunit
PPIAP19 (peptidylprolyl isomerase A pseudogene 19)
LDHAP5 (lactate dehydrogenase A pseudogene 5)
KRT8P49 (keratin 8 pseudogene 49)
ST13P5 (ST13, Hsp70 interacting protein pseudogene 5)
HSP90AA2P (heat shock protein 90 alpha family class A
FABP5P7 (fatty acid binding protein 5 pseudogene 7)
ALG1L8P (ALG1 like 8, pseudogene)
IFITM9P (interferon induced transmembrane protein 9
ALG1L9P (ALG1 like 9, pseudogene)
FTH1P16 (ferritin heavy chain 1 pseudogene 16)
HNRNPCP6 (heterogeneous nuclear ribonucleoprotein C
TUBAP2 (tubulin alpha pseudogene 2)
SRP14P2 (signal recognition particle 14 pseudogene 2)
WTAPP1 (Wilms tumor 1 associated protein pseudogene 1)
NAP1L1P1 (nucleosome assembly protein 1 like 1
WASH8P (WAS protein family homolog 8, pseudogene)
OTUD4P1 (OTUD4 pseudogene 1)
HSPA8P5 (heat shock protein family A (Hsp70) member 8
HADHAP2 (HADHA pseudogene 2)
PTMAP4 (prothymosin alpha pseudogene 4)
DDX12P (DEAD/H-box helicase 12, pseudogene)
PTMAP9 (prothymosin alpha pseudogene 9)
SUPT16HP1 (SPT16 homolog, facilitates chromatin
PSMC1P9 (proteasome 26S subunit, ATPase 1 pseudogene
EEF1A1P4 (eukaryotic translation elongation factor 1 alpha
TCP1P3 (t-complex 1 pseudogene 3)
IFITM3P2 (IFITM3 pseudogene 2)
EIF4A1P4 (eukaryotic translation initiation factor 4A1
HSPD1P4 (heat shock protein family D (Hsp60) member 1
PABPC1P4 (poly(A) binding protein cytoplasmic 1
CENPCP1 (centromere protein C pseudogene 1)
SUCLG2P2 (SUCLG2 pseudogene 2)
GOLGA2P5 (GOLGA2 pseudogene 5)
HSPE1P4 (heat shock protein family E (Hsp10) member 1
KRT18P20 (keratin 18 pseudogene 20)
NME2P1 (NME2 pseudogene 1)
CLIC1P1 (chloride intracellular channel 1 pseudogene 1)
EP400P1 (EP400 pseudogene 1)
FTH1P7 (ferritin heavy chain 1 pseudogene 7)
NUS1P2 (NUS1 pseudogene 2)
ATP5F1EP2 (ATP synthase F1 subunit epsilon pseudogene 2)
EEF1A1P3 (eukaryotic translation elongation factor 1 alpha
EIF4A1P7 (eukaryotic translation initiation factor 4A1
CYP51A1P2 (cytochrome P450 family 51 subfamily A
CALM2P3 (calmodulin 2 pseudogene 3)
TUBBP2 (tubulin beta pseudogene 2)
OR7E36P (olfactory receptor family 7 subfamily E member
RCN1P2 (reticulocalbin 1 pseudogene 2)
PSME2P2 (proteasome activator subunit 2 pseudogene 2)
COX7CP1 (cytochrome c oxidase subunit 7C pseudogene 1)
ST13P4 (ST13, Hsp70 interacting protein pseudogene 4)
CTAGE3P (CTAGE family member 3, pseudogene)
MRPS31P4 (mitochondrial ribosomal protein S31
LGMNP1 (legumain pseudogene 1)
HNRNPA3P5 (heterogeneous nuclear ribonucleoprotein A3
PTMAP5 (prothymosin alpha pseudogene 5)
H2AZP3 (H2A.Z histone pseudogene 3)
MTCO1P2 (MT-CO1 pseudogene 2)
ATP5F1CP1 (ATP synthase F1 subunit gamma pseudogene 1)
RBM8B (RNA binding motif protein 8B pseudogene)
HSPE1P2 (heat shock protein family E (Hsp10) member 1
YBX1P1 (Y-box binding protein 1 pseudogene 1)
DYNLL1P1 (dynein light chain LC8-type 1 pseudogene 1)
DYNLL1P2 (dynein light chain LC8-type 1 pseudogene 2)
ENSAP2 (endosulfine alpha pseudogene 2)
HERC2P2 (hect domain and RLD 2 pseudogene 2)
HERC2P9 (hect domain and RLD 2 pseudogene 9)
HMGN2P5 (high mobility group nucleosomal binding
SYNGR2P1 (synaptogyrin 2 pseudogene 1)
CATSPER2P1 (cation channel sperm associated 2
SORD2P (sorbitol dehydrogenase 2, pseudogene)
HMGN2P46 (high mobility group nucleosomal binding
EEF1B2P1 (eukaryotic translation elongation factor 1 beta 2
EEF1A1P22 (eukaryotic translation elongation factor 1
PIGHP1 (phosphatidylinositol glycan anchor biosynthesis
HMGB1P6 (high mobility group box 1 pseudogene 6)
HNRNPCP3 (heterogeneous nuclear ribonucleoprotein C
CSPG4P10 (chondroitin sulfate proteoglycan 4 pseudogene
SCAND2P (SCAN domain containing 2 pseudogene)
HSPE1P3 (heat shock protein family E (Hsp10) member 1
HMGN1P38 (high mobility group nucleosome binding
HSP90B2P (heat shock protein 90 beta family member 2,

ABCC6P2 (ATP binding cassette subfamily C member 6
ABCC6P1 (ATP binding cassette subfamily C member 6
RRN3P3 (RRN3 pseudogene 3)
HMGN2P3 (high mobility group nucleosomal binding
GAPDHP35 (glyceraldehyde 3 phosphate dehydrogenase
SMG1P2 (SMG1 pseudogene 2)
CLUHP3 (clustered mitochondria homolog pseudogene 3)
BCAP31P2 (B cell receptor associated protein 31
BCLAF1P2 (BCL2 associated transcription factor 1
VPS35P1 (VPS35 pseudogene 1)
NDUFA5P11 (NADH:ubiquinone oxidoreductase subunit A5
CES1P1 (carboxylesterase 1 pseudogene 1)
ATP5F1AP3 (ATP synthase F1 subunit alpha pseudogene 3)
ATP5PBP7 (ATP synthase peripheral stalk-membrane
URAHP (urate (hydroxyiso-) hydrolase, pseudogene)
TXNP4 (thioredoxin pseudogene 4)
ALOX12P2 (arachidonate 12-lipoxygenase pseudogene 2)
MEIS3P1 (Meis homeobox 3 pseudogene 1)
UPF3AP1 (UPF3A pseudogene 1)
ZNF286B (zinc finger protein 286B (pseudogene))
KRT17P6 (keratin 17 pseudogene 6)
MEIS3P2 (Meis homeobox 3 pseudogene 2)
LRRC37BP1 (leucine rich repeat containing 37B pseudogene
TLK2P1 (tousled like kinase 2 pseudogene 1)
HMGB1P24 (high mobility group box 1 pseudogene 24)
LRRC37A11P (leucine rich repeat containing 37 member
PTP4A2P1 (PTP4A2 pseudogene 1)
HMGN2P15 (high mobility group nucleosomal binding
LRRC37A4P (leucine rich repeat containing 37 member A4,
DND1P1 (DND microRNA-mediated repression inhibitor 1
LINC02210 (long intergenic non-protein coding RNA 2210)
LRRC37A17P (leucine rich repeat containing 37 member
MTCO1P40 (MT-CO1 pseudogene 40)
MICOS10P2 (MICOS10 pseudogene 2)
LRRC37A16P (leucine rich repeat containing 37 member
ATF4P3 (activating transcription factor 4 pseudogene 3)
KRT18P8 (keratin 18 pseudogene 8)
LRRC37A7P (leucine rich repeat containing 37 member A7,
HNRNPA1P7 (heterogeneous nuclear ribonucleoprotein A1
ZNF271P (zinc finger protein 271, pseudogene)
MTCO2P2 (MT-CO2 pseudogene 2)
SNRPGP2 (small nuclear ribonucleoprotein polypeptide G
SINHCAFP2 (SINHCAF pseudogene 2)
FAUP1 (FAU pseudogene 1)
SNRPEP4 (SNRPE pseudogene 4)
HNRNPA1P10 (heterogeneous nuclear ribonucleoprotein A1
ZNF788P (zinc finger family member 788, pseudogene)
PGK1P2 (phosphoglycerate kinase 1, pseudogene 2)
EEF1DP1 (eukaryotic translation elongation factor 1 delta
SNRPGP15 (small nuclear ribonucleoprotein polypeptide G
CYP4F23P (cytochrome P450 family 4 subfamily F member
IPO5P1 (importin 5 pseudogene 1)
SLC25A1P5 (solute carrier family 25 member 1 pseudogene
EEF1A1P7 (eukaryotic translation elongation factor 1 alpha
PRR13P5 (proline rich 13 pseudogene 5)
CYP2F2P (cytochrome P450 family 2 subfamily F member 2,
CYP2B7P (cytochrome P450 family 2 subfamily B member 7,
CYP2G2P (cytochrome P450 family 2 subfamily G member 2,
GAPDHP38 (glyceraldehyde 3 phosphate dehydrogenase
ZNF321P (zinc finger protein 321, pseudogene)
NDUFV2P1 (NADH:ubiquinone oxidoreductase core subunit
TPM3P9 (tropomyosin 3 pseudogene 9)
CENPBD1P1 (CENPB DNA-binding domains containing 1
EIF1P7 (eukaryotic translation initiation factor 1
DRG1P1 (DRG1 pseudogene 1)
NDUFAF2P1 (NDUFAF2 pseudogene 1)
FTH1P3 (ferritin heavy chain 1 pseudogene 3)
KRTCAP2P1 (KRTCAP2 pseudogene 1)
EIF3FP3 (eukaryotic translation initiation factor 3 subunit F
ATP1B3P1 (ATPase Na+/K+ transporting subunit beta 3
AHSA2P (activator of HSP90 ATPase homolog 2,
PRELID1P6 (PRELID1 pseudogene 6)
FNBP1P1 (formin binding protein 1 pseudogene 1)
LDHAP7 (lactate dehydrogenase A pseudogene 7)
PEBP1P2 (phosphatidylethanolamine binding protein 1
WBP1P1 (WBP1 pseudogene 1)
HMGN1P36 (high mobility group nucleosome binding
EEF1A1P12 (eukaryotic translation elongation factor 1
SNRPA1P1 (SNRPA1 pseudogene 1)
MED15P9 (mediator complex subunit 15 pseudogene 9)
NOC2LP1 (NOC2 like nucleolar associated transcriptional
SMPD4BP (sphingomyelin phosphodiesterase 4B,
HNRNPKP2 (heterogeneous nuclear ribonucleoprotein K
UBBP1 (ubiquitin B pseudogene 1)
YWHAEP5 (tyrosine 3-monooxygenase/tryptophan 5-
AHCTF1P1 (AT-hook containing transcription factor 1
H3P6 (H3 histone pseudogene 6)
NUDCP2 (nuclear distribution C pseudogene 2)
FTH1P20 (ferritin heavy chain 1 pseudogene 20)
KRT18P29 (keratin 18 pseudogene 29)
KRT8P10 (keratin 8 pseudogene 10)
CACYBPP2 (calcyclin binding protein pseudogene 2)
HNRNPCP2 (heterogeneous nuclear ribonucleoprotein C
NPM1P46 (nucleophosmin 1 pseudogene 46)
DAZAP2P1 (DAZ associated protein 2 pseudogene 1)
KRT18P39 (keratin 18 pseudogene 39)
HSPA9P1 (heat shock protein family A (Hsp70) member 9
TPM3P8 (tropomyosin 3 pseudogene 8)
BANF1P3 (BANF1 pseudogene 3)
FTLP3 (ferritin light chain pseudogene 3)
FRG1BP (FSHD region gene 1 family member B, pseudogene)
TSPY26P (testis specific protein Y-linked 26, pseudogene)
ERP29P1 (endoplasmic reticulum lumenal protein 29
HMGB1P1 (high mobility group box 1 pseudogene 1)
PPIAP22 (peptidylprolyl isomerase A pseudogene 22)
GPX1P2 (glutathione peroxidase pseudogene 2)
FABP5P11 (fatty acid binding protein 5 pseudogene 11)
KRT18P5 (keratin 18 pseudogene 5)
CCDC74BP1 (coiled-coil domain containing 74B pseudogene
FBXW4P1 (F-box and WD repeat domain containing 4
HMGB1P10 (high mobility group box 1 pseudogene 10)
NDUFA9P1 (NADH:ubiquinone oxidoreductase subunit A9
UQCRFS1P1 (ubiquinol-cytochrome c reductase, Rieske iron-
C22orf46 (CTA-216E10.6)
OLA1P1 (OLA1 pseudogene 1)
RRP7BP (ribosomal RNA processing 7 homolog B,
OR7E122P (olfactory receptor family 7 subfamily E member
EMC3-AS1 (EMC3 antisense RNA 1)
KRT18P17 (keratin 18 pseudogene 17)
TPRXL (tetrapeptide repeat homeobox like (pseudogene))
HMGB1P5 (high mobility group box 1 pseudogene 5)
KRT18P15 (keratin 18 pseudogene 15)
TCEA1P2 (transcription elongation factor A1 pseudogene 2)
EEF1A1P24 (eukaryotic translation elongation factor 1
RNF7P1 (ring finger protein 7 pseudogene 1)
PSMC1P1 (proteasome 26S subunit, ATPase 1 pseudogene
FTH1P23 (ferritin heavy chain 1 pseudogene 23)
HNRNPA3P6 (heterogeneous nuclear ribonucleoprotein A3
ALG1L6P (ALG1 like 6, pseudogene)
HNRNPKP4 (heterogeneous nuclear ribonucleoprotein K
PTMAP8 (prothymosin alpha pseudogene 8)
FTH1P4 (ferritin heavy chain 1 pseudogene 4)
HSPA8P9 (heat shock protein family A (Hsp70) member 8
KRT8P36 (keratin 8 pseudogene 36)
EEF1A1P25 (eukaryotic translation elongation factor 1
ST13P15 (ST13, Hsp70 interacting protein pseudogene 15)
SNRPCP3 (small nuclear ribonucleoprotein polypeptide C
AADACP1 (arylacetamide deacetylase pseudogene 1)
SETP14 (SET pseudogene 14)
ALG1L15P (ALG1 like 15, pseudogene)
PA2G4P4 (proliferation-associated 2G4 pseudogene 4)
KRT8P12 (keratin 8 pseudogene 12)
KRT8P13 (keratin 8 pseudogene 13)
SLC31A1P1 (solute carrier family 31 member 1 pseudogene
RNA5SP151 (RNA, 5S ribosomal pseudogene 151)
EEF1A1P8 (eukaryotic translation elongation factor 1 alpha
MUC20P1 (mucin 20, cell surface associated pseudogene 1)
FAM86EP (family with sequence similarity 86, member A
RNPS1P1 (RNA binding protein with serine rich domain 1
EXOC5P1 (exocyst complex component 5 pseudogene 1)
IFITM3P1 (IFITM3 pseudogene 1)
MT2P1 (metallothionein 2 pseudogene 1)
TXNP6 (thioredoxin pseudogene 6)
SERBP1P5 (SERPINE1 mRNA binding protein 1 pseudogene
TECRP1 (trans-2,3-enoyl-CoA reductase pseudogene 1)
GAPDHP60 (glyceraldehyde-3-phosphate dehydrogenase
HSP90AB3P (heat shock protein 90 alpha family class B
TMSB4XP8 (TMSB4X pseudogene 8)
EEF1A1P9 (eukaryotic translation elongation factor 1 alpha
CEP170P1 (centrosomal protein 170 pseudogene 1)
GTF2IP12 (general transcription factor IIi pseudogene 12)
SEPTIN7P14 (septin 7 pseudogene 14)
NACA3P (NACA family member 3, pseudogene)
MORF4 (mortality factor 4 (pseudogene))
SDHAP3 (succinate dehydrogenase complex flavoprotein
CCT6P2 (chaperonin containing TCP1 subunit 6 pseudogene
EEF1A1P13 (eukaryotic translation elongation factor 1
RBBP4P1 (RBBP4 pseudogene 1)
HSPD1P1 (heat shock protein family D (Hsp60) member 1
EEF1A1P19 (eukaryotic translation elongation factor 1
NAIPP2 (NAIP pseudogene 2)
FUNDC2P1 (FUN14 domain containing 2 pseudogene 1)
HMGN2P4 (high mobility group nucleosomal binding
ALDH7A1P1 (aldehyde dehydrogenase 7 family member A1
ACTBP2 (ACTB pseudogene 2)
MTRNR2L2 (MT-RNR2 like 2)
PPIAP11 (peptidylprolyl isomerase A pseudogene 11)
NPM1P27 (nucleophosmin 1 pseudogene 27)
PSME2P1 (proteasome activator subunit 2 pseudogene 1)
KRT8P32 (keratin 8 pseudogene 32)
CTBP2P4 (CTBP2 pseudogene 4)
MTCO2P22 (MT-CO2 pseudogene 22)
BCLAF1P1 (BCL2 associated transcription factor 1
PTMAP2 (prothymosin alpha pseudogene 2)
KRT8P33 (keratin 8 pseudogene 33)
KRT18P16 (keratin 18 pseudogene 16)
HNRNPKP1 (heterogeneous nuclear ribonucleoprotein K
MTCYBP18 (MT-CYB pseudogene 18)
MTND6P4 (MT-ND6 pseudogene 4)
MTND5P11 (MT-ND5 pseudogene 11)
MTND4P12 (MT-ND4 pseudogene 12)
MTND4LP30 (MT-ND4L pseudogene 30)
KRT8P48 (keratin 8 pseudogene 48)
GAPDHP40 (glyceraldehyde 3 phosphate dehydrogenase
KRT18P41 (keratin 18 pseudogene 41)
SUMO2P6 (SUMO2 pseudogene 6)
HTATSF1P2 (HIV-1 Tat specific factor 1 pseudogene 2)
KRT18P38 (keratin 18 pseudogene 38)
PPIAP29 (peptidylprolyl isomerase A pseudogene 29)
ASS1P1 (argininosuccinate synthetase 1 pseudogene 1)
CMAHP (cytidine monophospho-N-acetylneuraminic acid
ZNF204P (zinc finger protein 204, pseudogene)
KRT18P1 (keratin 18 pseudogene 1)
SUMO2P1 (SUMO2 pseudogene 1)
HLA-H (major histocompatibility complex, class I, H
HLA-K (major histocompatibility complex, class I, K
ZNRD1ASP (zinc ribbon domain containing 1 antisense,
RANP1 (RAN pseudogene 1)
GGNBP1 (gametogenetin binding protein 1 (pseudogene))
LAP3P2 (leucine aminopeptidase 3 pseudogene 2)

FTH1P5 (ferritin heavy chain 1 pseudogene 5)
FTH1P15 (ferritin heavy chain 1 pseudogene 15)
ADH5P4 (ADH5 pseudogene 4)
LYPLA1P3 (LYPLA1 pseudogene 3)
EIF3EP1 (eukaryotic translation initiation factor 3 subunit E
GAPDHP63 (glyceraldehyde 3 phosphate dehydrogenase
KRT18P64 (keratin 18 pseudogene 64)
ATP5MFP2 (ATP synthase membrane subunit f pseudogene
KRT18P65 (keratin 18 pseudogene 65)
TPI1P3 (triosephosphate isomerase 1 pseudogene 3)
TPT1P4 (tumor protein, translationally-controlled 1
PDCL3P5 (PDCL3 pseudogene 5)
ALG1L5P (ALG1 like 5, pseudogene)
HSPA8P8 (heat shock protein family A (Hsp70) member 8
EEF1A1P6 (eukaryotic translation elongation factor 1 alpha
EIF4HP1 (eukaryotic translation initiation factor 4H
DPY19L1P1 (DPY19L1 pseudogene 1)
RWDD4P2 (RWD domain containing 4 pseudogene 2)
TCP1P1 (t-complex 1 pseudogene 1)
GTF2IP13 (general transcription factor IIi pseudogene 13)
SLC25A5P3 (solute carrier family 25 member 5 pseudogene
FKBP9P1 (FKBP prolyl isomerase 9 pseudogene 1)
PSPHP1 (phosphoserine phosphatase pseudogene 1)
IFITM3P4 (IFITM3 pseudogene 4)
YWHAEP1 (tyrosine 3-monooxygenase/tryptophan 5-
STAG3L4 (stromal antigen 3-like 4 (pseudogene))
NSUN5P2 (NSUN5 pseudogene 2)
STAG3L3 (stromal antigen 3-like 3 (pseudogene))
PMS2P7 (PMS1 homolog 2, mismatch repair system
PMS2P5 (PMS1 homolog 2, mismatch repair system
NCF1C (neutrophil cytosolic factor 1C pseudogene)
PMS2P2 (PMS1 homolog 2, mismatch repair system
EIF4BP6 (eukaryotic translation initiation factor 4B
YBX1P2 (Y-box binding protein 1 pseudogene 2)
CICP14 (capicua transcriptional repressor pseudogene 14)
EEF1B2P6 (eukaryotic translation elongation factor 1 beta 2
ST13P7 (ST13, Hsp70 interacting protein pseudogene 7)
PSMC1P3 (proteasome 26S subunit, ATPase 1 pseudogene
KRT8P51 (keratin 8 pseudogene 51)
EEF1A1P10 (eukaryotic translation elongation factor 1
LSM12P1 (LSM12 pseudogene 1)
RNF5P1 (ring finger protein 5 pseudogene 1)
NPM1P6 (nucleophosmin 1 pseudogene 6)
KRT8P3 (keratin 8 pseudogene 3)
PCBP2P2 (poly(rC) binding protein 2 pseudogene 2)
PKMP4 (pyruvate kinase M1/2 pseudogene 4)
FTH1P11 (ferritin heavy chain 1 pseudogene 11)
HNRNPA1P4 (heterogeneous nuclear ribonucleoprotein A1
CPP (ceruloplasmin pseudogene)
TAGLN2P1 (transgelin 2 pseudogene 1)
ST13P6 (ST13, Hsp70 interacting protein pseudogene 6)
SMPD5 (sphingomyelin phosphodiesterase 5 (pseudogene))
ZNF252P (zinc finger protein 252, pseudogene)
SELENOTP1 (selenoprotein T pseudogene 1)
SNRPEP2 (SNRPE pseudogene 2)
PRDX1P1 (peroxiredoxin 1 pseudogene 1)
CLCN3P1 (chloride voltage-gated channel 3 pseudogene 1)
FTH1P12 (ferritin heavy chain 1 pseudogene 12)
RBMXP2 (RBMX pseudogene 2)
GLULP4 (glutamate-ammonia ligase pseudogene 4)
YBX1P10 (Y-box binding protein 1 pseudogene 10)
RAB1C (RAB1C, member RAS oncogene family pseudogene)
TMX2P1 (thioredoxin related transmembrane protein 2
PGM5P2 (phosphoglucomutase 5 pseudogene 2)
DUX4L50 (double homeobox 4 like 50 (pseudogene))
FRG1JP (FSHD region gene 1 family member J, pseudogene)
FAM27B (family with sequence similarity 27 member B)
EEF1DP2 (eukaryotic translation elongation factor 1 delta
PTMAP12 (prothymosin alpha pseudogene 12)
EIF4BP3 (eukaryotic translation initiation factor 4B
RALGAPA1P1 (RALGAPA1 pseudogene 1)
LRRC37A5P (leucine rich repeat containing 37 member A5,
TPT1P9 (tumor protein, translationally-controlled 1
CUTALP (cutA divalent cation tolerance like, pseudogene)
HMGB1P37 (high mobility group box 1 pseudogene 37)
EEF1A1P5 (eukaryotic translation elongation factor 1 alpha
ARF4P1 (ADP ribosylation factor 4 pseudogene 1)
EIF5P1 (eukaryotic translation initiation factor 5
GPX1P1 (glutathione peroxidase pseudogene 1)
UBE2E4P (ubiquitin conjugating enzyme E2 E4 pseudogene)
CA5BP1 (carbonic anhydrase 5B pseudogene 1)
EEF1B2P3 (eukaryotic translation elongation factor 1 beta 2
RANBP1P1 (RANBP1 pseudogene 1)
FTLP2 (ferritin light chain pseudogene 2)
GAPDHP1 (glyceraldehyde-3-phosphate dehydrogenase
SDCBPP3 (syndecan binding protein pseudogene 3)
CHTF8P1 (chromosome transmission fidelity factor 8
KRT18P68 (keratin 18 pseudogene 68)
GAPDHP65 (glyceraldehyde 3 phosphate dehydrogenase
VDAC1P2 (voltage dependent anion channel 1 pseudogene
H3P44 (H3 histone pseudogene 44)
RBMXP5 (RBMX pseudogene 5)
PGK1P1 (phosphoglycerate kinase 1 pseudogene 1)
SERBP1P1 (SERPINE1 mRNA binding protein 1 pseudogene
CNOT7P1 (CCR4-NOT transcription complex subunit 7
SHISA5P2 (SHISA5 pseudogene 2)
RAB11FIP1P1 (RAB11 family interacting protein 1
TTC3P1 (tetratricopeptide repeat domain 3 pseudogene 1)
LDHBP2 (lactate dehydrogenase B pseudogene 2)
VDAC1P1 (voltage dependent anion channel 1 pseudogene
STIP1P3 (stress induced phosphoprotein 1 pseudogene 3)
EIF4A1P10 (eukaryotic translation initiation factor 4A1
KRT18P11 (keratin 18 pseudogene 11)
ST13P18 (ST13, Hsp70 interacting protein pseudogene 18)
GK4P (glycerol kinase 4 pseudogene)
GTF3C6P2 (GTF3C6 pseudogene 2)
DNAJA1P3 (DnaJ heat shock protein family (Hsp40) member
EIF4BP7 (eukaryotic translation initiation factor 4B
EEF1GP5 (eukaryotic translation elongation factor 1 gamma
SUMO2P21 (SUMO2 pseudogene 21)
RHOXF1P3 (Rhox homeobox family member 1 pseudogene
HSPA8P1 (heat shock protein family A (Hsp70) member 8
MTND4P24 (MT-ND4 pseudogene 24)
PNKDP1 (PNKD pseudogene 1)
FTH1P8 (ferritin heavy chain 1 pseudogene 8)
XRCC6P2 (X-ray repair cross complementing 6 pseudogene
KRT8P8 (keratin 8 pseudogene 8)
IKBKGP1 (inhibitor of nuclear factor kappa B kinase subunit
HMGN1P37 (high mobility group nucleosome binding
EIF4A1P2 (eukaryotic translation initiation factor 4A1
KRT18P10 (keratin 18 pseudogene 10)
