# Supplementary material for: Multi-omic characterization of pediatric ARDS via nasal brushings
Source: Respir Res. 2022 Jul 9;23:181. doi: 10.1186/s12931-022-02098-3 (PMC9270778; doi:10.1186/s12931-022-02098-3)
Supplement: Supplementary file 17 — Additional file 17: Table S6. Differentially Expressed Genes. [file 12931_2022_2098_MOESM17_ESM.pdf]

Supplemental Table 6: Differentially Abundant Microbial Species Summary

	PARDS: Subgroup A	PARDS: Subgroup B	PARDS: Subgroup C	PARDS: Subgroup D
Control	0	0	5	0
PARDS: Subgroup A		0	0	0
PARDS: Subgroup B			0	0
PARDS: Subgroup C				2
PARDS: Subgroup D				
