# Supplementary material for: Multi-omic characterization of pediatric ARDS via nasal brushings
Source: Respir Res. 2022 Jul 9;23:181. doi: 10.1186/s12931-022-02098-3 (PMC9270778; doi:10.1186/s12931-022-02098-3)
Supplement: Supplementary file 18 — Additional file 18: Table S7. Differentially Abundant Microbial Species Summary. [file 12931_2022_2098_MOESM18_ESM.pdf]

Supplemental Table 7: Differentially Abundant Microbial Species

PARDS Subgroup C vs. Control	PARDS Subgroup C vs. D
Streptococcus sp.	*Gemella haemolysans*
Corynebacterium kefirresidentii	*Corynebacterium kefirresidentii*
Lactobacillales sp.	
Dolosigranulum pigrum	
Haemophilus sp.	
